# Thermodynamic *versus* kinetic control in substituent redistribution reactions of silylium ions steered by the counteranion†
†Electronic supplementary information (ESI) available: Experimental details, characterization, spectroscopic and crystallographic data, DFT calculation methods, energy data, and the coordinates of the calculated geometries. CCDC 1818576, 1818581, and 1818582. For ESI and crystallographic data in CIF or other electronic format see DOI: 10.1039/c8sc01833b


**DOI:** 10.1039/c8sc01833b

**Published:** 2018-05-21

**Authors:** Lukas Omann, Bimal Pudasaini, Elisabeth Irran, Hendrik F. T. Klare, Mu-Hyun Baik, Martin Oestreich

**Affiliations:** a Institut für Chemie , Technische Universität Berlin , Strasse des 17. Juni 115 , 10623 Berlin , Germany . Email: martin.oestreich@tu-berlin.de; b Center for Catalytic Hydrocarbon Functionalizations , Institute for Basic Science (IBS) , Daejeon , 34141 , Republic of Korea; c Department of Chemistry , Korea Advanced Institute of Science and Technology (KAIST) , Daejeon , 34141 , Republic of Korea . Email: mbaik2805@kaist.ac.kr

## Abstract

An in-depth experimental and theoretical study of the substituent exchange reaction of silylium ions is presented. Apart from the substitution pattern at the silicon atom, the selectivity of this process is predominantly influenced by the counteranion, which is introduced with the trityl salt in the silylium ion generation. In contrast to Müller's protocol for the synthesis of triarylsilylium ions under kinetic control, the use of Reed's carborane anions leads to contact ion pairs, allowing selective formation of trialkylsilylium ions under thermodynamic control. DFT calculations finally revealed an unexpected mechanism for the rate-determining alkyl exchange step, which is initiated by an unusual 1,2-silyl migration in the intermediate *ipso*-disilylated arenium ion. The resulting *ortho*-disilylated arenium ion can then undergo an alkyl transfer *via* a low-barrier five-centered transition state.

## Abstract 2

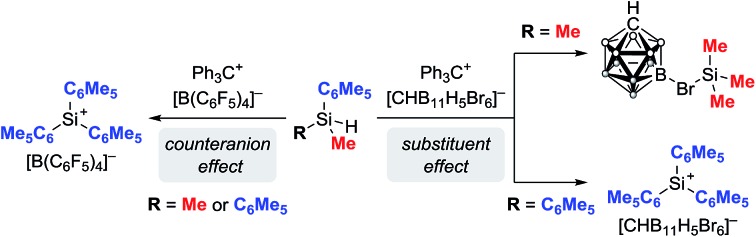
Substituent exchange reactions of silylium ions can be steered in opposite directions. The judicious choice of the hydrosilane and the counteranion enables the selective formation of either triaryl- or trialkylsilylium ions.

## Introduction

**Scheme 1:**
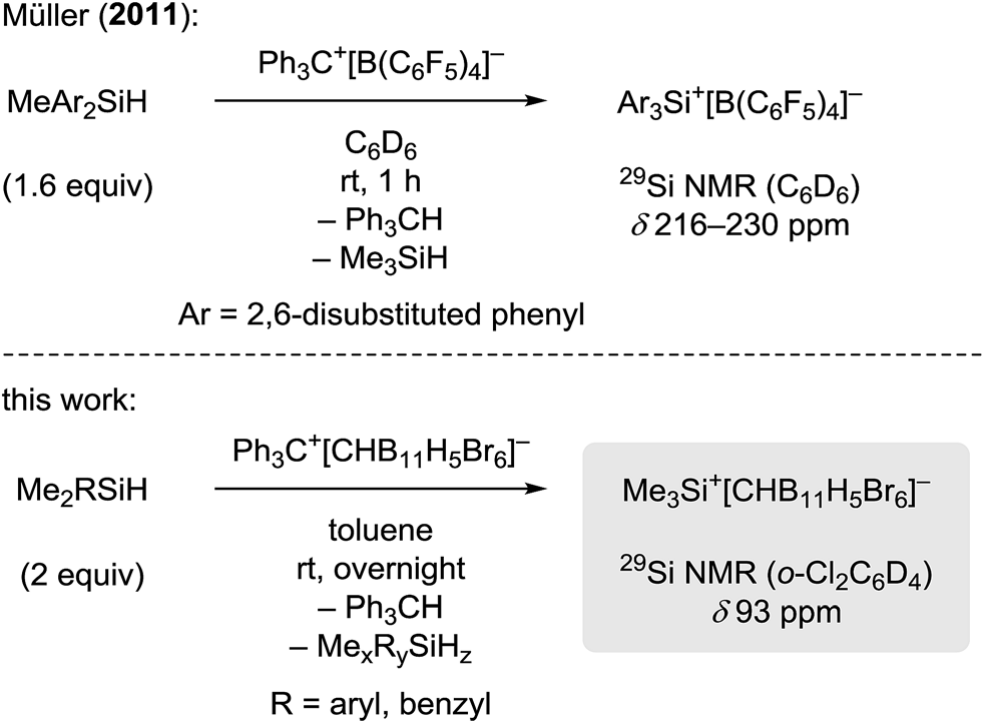
Divergence in the generation of silylium ions by substituent redistribution (*x* + *y* + *z* = 4).

Silylium ions (R_3_Si^+^) have recently emerged as useful and versatile catalysts for synthetically attractive transformations.^1^,^2^ The most commonly used approach to generate silylium ions is the Bartlett–Condon–Schneider reaction,^3^ that is the silicon-to-carbon hydride transfer from a hydrosilane to the trityl cation (Ph_3_C^+^) paired with a weakly coordinating counteranion.^4^,^5^ However, substituent redistribution of the hydrosilane starting material can occur under these highly Lewis acidic reaction conditions, leading to undesired mixtures of various silicon compounds.^6^–^8^ Hence, hydrosilanes containing three identical substituents, *e.g.* Et_3_SiH or iPr_3_SiH, are usually employed in this reaction.^9^ Conversely, Müller and co-workers have turned this unselective process into a useful synthetic route to triarylsilylium ions (Scheme 1, top).^10^ When sterically demanding methyl(diaryl)silanes MeAr_2_SiH are used in the hydride abstraction with Ph_3_C^+^[B(C_6_F_5_)_4_]^–^, the formation of otherwise difficult to prepare triarylsilylium ions Ar_3_Si^+^[B(C_6_F_5_)_4_]^–^ is observed.^11^ Notably, the useof less bulky hydrosilanes such as MePh_2_SiH or Me(*o*-Tol)_2_SiH does not give triarylsilylium ions but mixtures of different silicon cations.^12^

Herein, we report that treatment of hydrosilanes of type Me_2_RSiH (R = aryl, benzyl) with Reed's carborane-based trityl salt Ph_3_C^+^[CHB_11_H_5_Br_6_]^–^ (ref. 13) results in substituent exchange reactions selectively forming the elusive trimethylsilylium ion Me_3_Si^+^[CHB_11_H_5_Br_6_]^–^ (Scheme 1, bottom). This method thus complements Müller's approach and offers a practical route to Me_3_Si^+^, avoiding the use of gaseous and highly flammable Me_3_SiH.^14^ A systematic experimental and computational investigation was performed to gain a full mechanistic picture of this phenomenon. DFT calculations revealed an unexpected mechanism and suggested an active role of the carborane counteranion in the outcome of these reactions.

## Results and discussion

### Generation of the trimethylsilylium ion by substituent redistribution

**Fig. 1:**
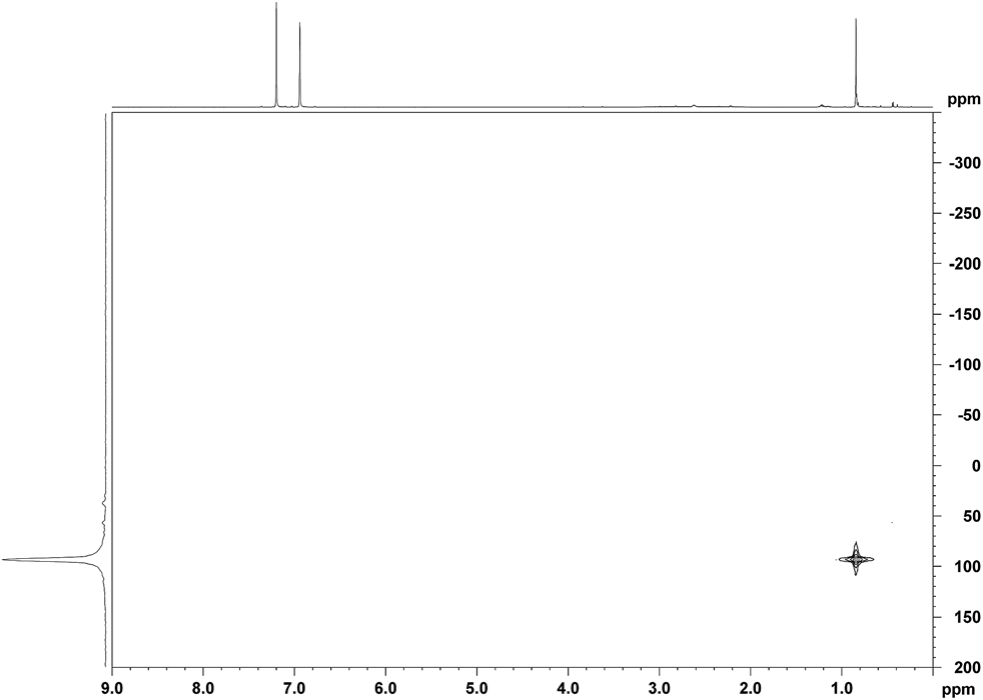
^1^H/^29^Si HMQC NMR spectrum (500/99 MHz, *o*-Cl_2_C_6_D_4_, 298 K, optimized for *J* = 7 Hz) of Me_3_Si^+^[CHB_11_H_5_Br_6_]^–^ from the reaction of Me_2_PhSiH with Ph_3_C^+^[CHB_11_H_5_Br_6_]^–^.

When a mixture of Me_2_PhSiH and Ph_3_C^+^[CHB_11_H_5_Br_6_]^–^ in toluene was stirred overnight at room temperature, a white suspension was obtained. The solid was collected by filtration, washed with *n*-pentane, and dissolved in *o*-Cl_2_C_6_D_4_ for NMR spectroscopic analysis. Unexpectedly, only a singlet at 0.83 ppm was detected in the ^1^H NMR spectrum, while no aromatic resonances except for those of the deuterated solvent were observed. The low-field ^29^Si NMR chemical shift of 93 ppm in the corresponding ^1^H/^29^Si HMQC spectrum, which is characteristic of trialkylsilylium ions, indicated clean formation of Me_3_Si^+^[CHB_11_H_5_Br_6_]^–^ (Fig. 1). The structural integrity of the carborane counteranion was confirmed by ^11^B NMR spectroscopy.

**Fig. 2:**
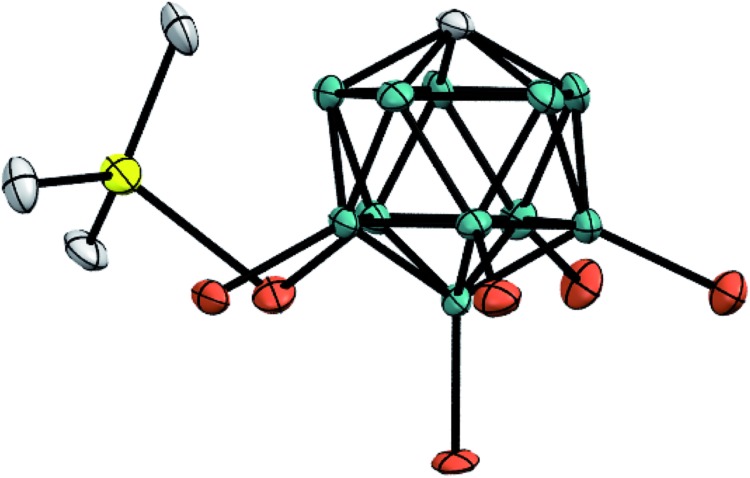
Molecular structure of Me_3_Si^+^[CHB_11_H_5_Br_6_]^–^ (thermal ellipsoids at the 50% probability level; H atoms omitted for clarity).

Unambiguous evidence for the structure of Me_3_Si^+^[CHB_11_H_5_Br_6_]^–^ was eventually provided by its crystallographic characterization (Fig. 2).^15^ Single crystals suitable for X-ray diffraction analysis were obtained by vapor diffusion with *n*-hexane from a solution of the silylium salt in *o*-F_2_C_6_H_4_ at room temperature. In accordance with reported molecular structures of silylium carboranes,^16^ one bromine atom at the pentagonal belt of the icosahedral anion is bound to the silicon cation. Both the Si–Br bond distance of 2.435(6) Å and the sum of all C–Si–C bond angles of 346.3(6)° are comparable to the larger Et_3_Si^+^[CHB_11_H_5_Br_6_]^–^.

In contrast to the clean formation of Me_3_Si^+^, the non-polar *n*-pentane filtrate contained several tri- and tetraorganosilanes, such as Ph_4_Si, MePh_3_Si, Ph_3_SiH, Me_2_Ph_2_Si, MePh_2_SiH, Me_3_PhSi, and Me_2_PhSiH, as verified by GC-MS analysis. Since silylium ions are known to promote substituent redistribution,^8^ this result did not come as a surprise but raised the question why Me_3_Si^+^ was selectively formed in this reaction mixture, whereas Müller's conditions cleanly afford sterically congested triarylsilylium ions.^10^

### Influence of the substituent pattern at the silicon atom on the selectivity of the substituent redistribution reaction

To understand the differences between Müller's protocol^10^ and our findings, we systematically studied the hydride transfer reaction of various hydrosilanes of type MeAr_2_SiH and Me_2_ArSiH using trityl salts Ph_3_C^+^[B(C_6_F_5_)_4_]^–^ and Ph_3_C^+^[CHB_11_H_5_Br_6_]^–^ (Table 1). Depending on the counteranion, slightly modified procedures were applied for the generation of the silicon cations (see the ESI† for details). For all reactions, an excess of hydrosilane (4 equiv.) was used, thereby excluding any influence of stoichiometry on the product formation. In accordance with Müller's report, bulky methyl(diaryl)silane Me(C_6_Me_5_)_2_SiH was converted to the corresponding triarylsilylium ion, regardless of which counteranion was used (entries 1 and 2). In contrast, hydride abstraction from sterically less hindered MePh_2_SiH with Ph_3_C^+^[B(C_6_F_5_)_4_]^–^ led to a complex reaction mixture as a result of anion decomposition (entry 3).^12^,^17^ The use of the carborane counteranion [CHB_11_H_5_Br_6_]^–^ furnished the unscrambled silylium ion MePh_2_Si^+^[CHB_11_H_5_Br_6_]^–^, as confirmed by X-ray diffraction analysis (entry 4; see the ESI† for the molecular structure of MePh_2_Si^+^[CHB_11_H_5_Br_6_]^–^).^15^ However, the formation of the MePh_2_Si^+^ cation was accompanied by a substantial amount of a second silylium ion, which was found to be the Me_2_PhSi^+^ cation.^18^ Notably, longer reaction times (7 days) or elevated temperatures (50 °C for 72 h) did not significantly change the product ratio of ∼79 : 21 (not shown). In all cases, the generation of Me_3_Si^+^ was not observed. We then turned our attention to dimethyl(aryl)silanes (entries 5–8). The reaction of Me_2_PhSiH with Ph_3_C^+^[B(C_6_F_5_)_4_]^–^ again resulted in decomposition of the borate counteranion (entry 5).^17^ Conversely, treatment of Me_2_PhSiH with trityl carborane Ph_3_C^+^[CHB_11_H_5_Br_6_]^–^ exclusively afforded silylium salt Me_3_Si^+^[CHB_11_H_5_Br_6_]^–^ without detectable formation of MePh_2_Si^+^ or Me_2_PhSi^+^ (entry 6). Strikingly, hydride abstraction from sterically more demanding Me_2_(C_6_Me_5_)SiH led to the corresponding triarylsilylium ion in the presence of the borate counteranion (entry 7), while substituent redistribution into the ‘opposite direction’ was induced by the carborane anion, now affording Me_3_Si^+^[CHB_11_H_5_Br_6_]^–^ (entry 8).^19^ However, heating of the reaction at 50 °C for 72 h was necessary.

**Table 1:** Silylium ion generation by substituent redistribution: effect of the hydrosilane and counteranion (Si = triorganosilyl)

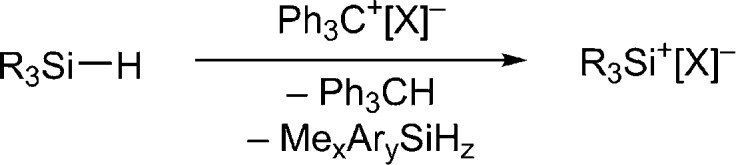				
Entry^*a*^	Si–H (4 equiv.)	[X]^–^	Si^+^	*δ*(^29^Si)^*b*^ [ppm]
1	Me(C_6_Me_5_)_2_SiH	[B(C_6_F_5_)_4_]^–^	(C_6_Me_5_)_3_Si^+^	217
2	Me(C_6_Me_5_)_2_SiH	[CHB_11_H_5_Br_6_]^–^	(C_6_Me_5_)_3_Si^+^	217
3	MePh_2_SiH	[B(C_6_F_5_)_4_]^–^	—^*c*^	—
4	MePh_2_SiH	[CHB_11_H_5_Br_6_]^–^	MePh_2_Si^+^/Me_2_PhSi^+^^*d*^	57/76
5	Me_2_PhSiH	[B(C_6_F_5_)_4_]^–^	—^*c*^	—
6	Me_2_PhSiH	[CHB_11_H_5_Br_6_]^–^	Me_3_Si^+^	93
7	Me_2_(C_6_Me_5_)SiH	[B(C_6_F_5_)_4_]^–^	(C_6_Me_5_)_3_Si^+^	217
8^*e*^	Me_2_(C_6_Me_5_)SiH	[CHB_11_H_5_Br_6_]^–^	Me_3_Si^+^	93

^*a*^All reactions were performed according to General Procedure (GP) 1 for X^–^ = [B(C_6_F_5_)_4_]^–^ (C_6_D_6_, room temperature, 60 min) or GP 2 for X^–^ = [CHB_11_H_5_Br_6_]^–^ (toluene, room temperature, 18–24 h). See the ESI for details.

^*b*^Measured in *o*-Cl_2_C_6_D_4_.

^*c*^A complex mixture was obtained as a result of counteranion decomposition.^17^

^*d*^Ratio of 79 : 21 determined by ^1^H NMR spectroscopy.

^*e*^Reaction performed at 50 °C for 72 h.

Overall, these results indicate that hydride abstraction from hydrosilanes of type Me_2_ArSiH with a carborane-based trityl salt tends to form the trimethylsilylium ion, whereas hydrosilanes of type MeAr_2_SiH with a bulky aryl substituent favor triarylsilylium ion generation.

### Mechanism of the substituent redistribution reaction with Me_2_PhSiH

**Fig. 3:**
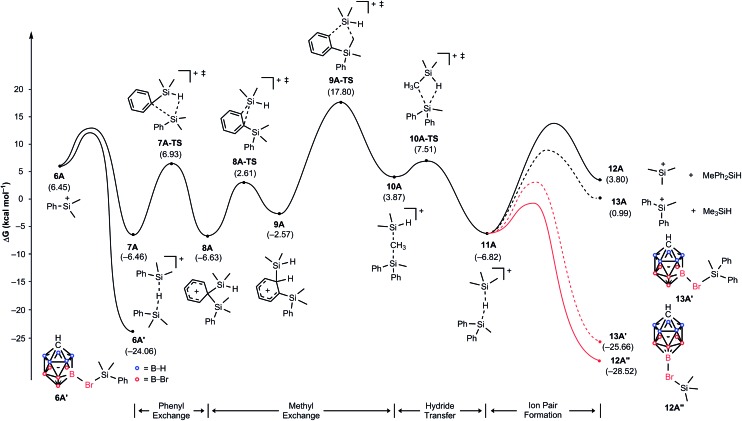
Energy (kcal mol^–1^) profile of the substituent redistribution in the reaction of Me_2_PhSiH (**1A**) with Ph_3_C^+^[CHB_11_H_5_Br_6_]^–^ (**2A**). The energies are relative to the starting materials **1A** and **2A**.

To gain insight into the reaction mechanism and to understand why the treatment of Me_2_PhSiH with Ph_3_C^+^[CHB_11_H_5_Br_6_]^–^ exclusively gives Me_3_Si^+^[CHB_11_H_5_Br_6_]^–^, we constructed a complete reaction energy profile using DFT calculations at the M06/cc-pVTZ(-f)//6-31G** level of theory (Fig. 3; see the ESI† for details of the computational method).^20^ The hydride abstraction from Me_2_PhSiH with Ph_3_C^+^[CHB_11_H_5_Br_6_]^–^ was found to have a barrier of 15.5 kcal mol^–1^ and is therefore expected to occur rapidly at room temperature (not shown). In the condensed phase, the resulting silylium ion Me_2_PhSi^+^ (**6A**), which is located at a relative free energy of 6.5 kcal mol^–1^, is stabilized through coordination by the solvent, another hydrosilane molecule, or by the counteranion (see the ESI† for a comparison of the association energies).8e,^21^ Coordination of one of the bromine atoms of the carborane counteranion to the silicon cation results in the highest binding energy, and the resulting ion pair **6A′** is predicted to be at a relative free energy of –24.1 kcal mol^–1^. Silylium ion **6A** can also interact with another equivalent of Me_2_PhSiH to form hydride-bridged adduct **7A**,^21^ located at –6.5 kcal mol^–1^. Note that these energies are not adjusted for the different concentrations of the components and assume normal conditions. Given that Me_2_PhSiH (**1A**) is present in excess, these normal energies suggest that adduct **7A** will be encountered easily in significant quantities.

Hydride-bridged adduct ion **7A** can undergo a phenyl group transfer to arrive at phenyl-bridged adduct **8A**7c,8b,^22^ *via* the four-centered transition state **7A-TS**, associated with a barrier of 13.4 kcal mol^–1^. Surprisingly, the subsequent methyl group transfer does not proceed *via* another typical four-membered transition state.^23^ Instead, our calculations suggest that 1,2-migration of the silicon group in **8A** occurs *via* the low barrier transition state **8A-TS**, leading to *ortho*-disilylated arenium ion **9A**. This seemingly unfavorable intermediate is only 4.1 kcal mol^–1^ higher in energy than arenium ion **8A**. Finally, **9A** facilitates the exchange of one methyl group, passing through five-centered transition state **9A-TS** with an overall barrier of 24.3 kcal mol^–1^ relative to **7A**. This energetically most demanding reaction step forms methonium ion **10A**, which is metastable and rapidly rearranges to hydride-bridged adduct **11A***via* low barrier transition state **10A-TS**. The hydrosilane-stabilized silylium ions **7A** and **11A** are almost isoenergetic (Δ*G* = 0.4 kcal mol^–1^), suggesting that both structures coexist in equilibrium. The formal dissociation of **11A** gives either Me_3_Si^+^ or MePh_2_Si^+^, the former being calculated to be 2.8 kcal mol^–1^ higher in energy. However, coordination by the carborane anion changes the energy landscape decisively, as ion pair formation reverses the energy ordering. Me_3_Si^+^[CHB_11_H_5_Br_6_]^–^ (**12A′′**), which is located at –28.5 kcal mol^–1^, is 2.9 kcal mol^–1^ lower in energy than MePh_2_Si^+^[CHB_11_H_5_Br_6_]^–^ (**13A′**) and also 4.5 kcal mol^–1^ more stable than Me_2_PhSi^+^[CHB_11_H_5_Br_6_]^–^ (**6A′**), thus predicting the silylium salt **12A′′** as the major product of the substituent redistribution reaction.

**Scheme 2:**
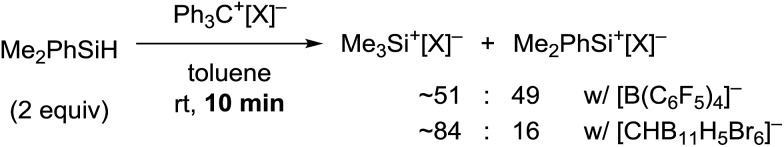
Influence of the counteranion on the selectivity of the trimethylsilylium ion formation.

It should be noted that silylium ions are significantly more stabilized by coordination of the carborane counteranion than by formation of solvent adducts such as R_3_Si(toluene)^+^[CHB_11_H_5_Br_6_]^–^. Moreover, the energy differences between these arenium ions are small, predicting a mixture of different silylium ions in the absence of the carborane counteranion (see the ESI† for details).^24^ This result was supported by independent control experiments (Scheme 2). The hydride abstraction from Me_2_PhSiH with borate-based trityl salt Ph_3_C^+^[B(C_6_F_5_)_4_]^–^ was repeated but stopped after stirring for 10 min in toluene (*cf.*Table 1, entry 5). NMR spectroscopic analysis of the polar phase in *o*-Cl_2_C_6_D_4_ revealed the formation of a mixture of Me_3_Si^+^[B(C_6_F_5_)_4_]^–^ and Me_2_PhSi^+^[B(C_6_F_5_)_4_]^–^ in a ratio of ∼51 : 49 along with small amounts of byproducts arising from counteranion decomposition. In contrast, stopping the reaction of Me_2_PhSiH with Ph_3_C^+^[CHB_11_H_5_Br_6_]^–^ after stirring for 10 min in toluene furnished Me_3_Si^+^[CHB_11_H_5_Br_6_]^–^ as the major product along with only small amounts of unscrambled Me_2_PhSi^+^[CHB_11_H_5_Br_6_]^–^ (ratio ∼84 : 16). In both reactions, full conversion of the trityl salt was observed.

**Fig. 4:**
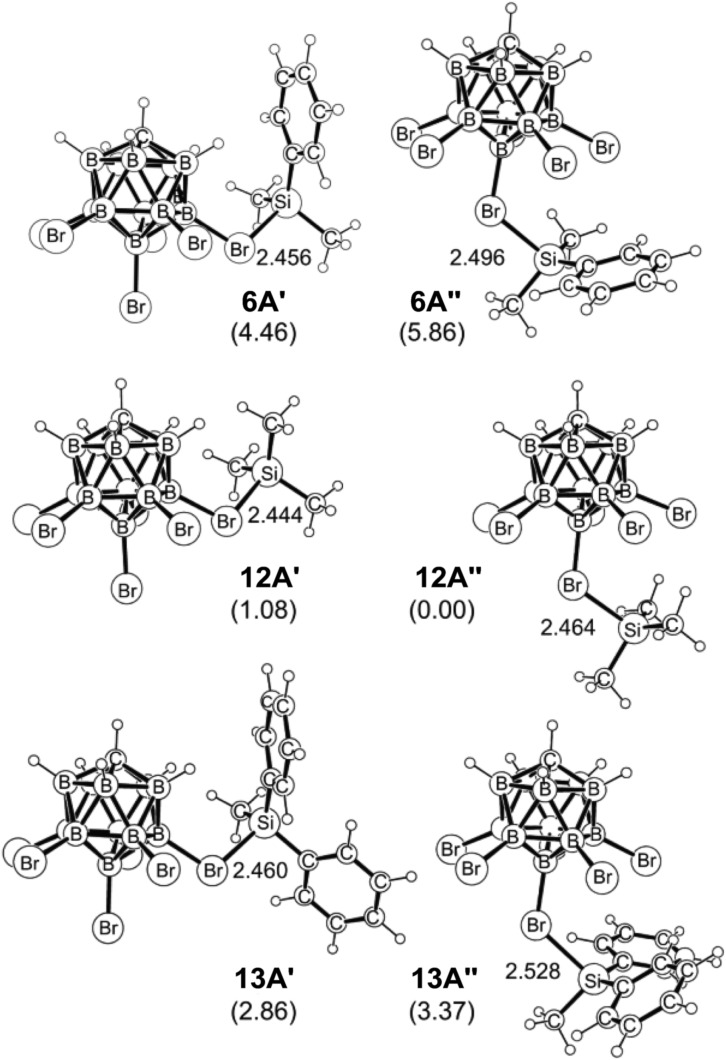
Computed apical and equatorial isomers of Me_2_PhSi^+^[CHB_11_H_5_Br_6_]^–^ (top), Me_3_Si^+^[CHB_11_H_5_Br_6_]^–^ (middle) and MePh_2_Si^+^[CHB_11_H_5_Br_6_]^–^ (bottom). Si–Br bond lengths are given in Å and relative free energy differences (kcal mol^–1^) are shown in parentheses.

As shown in Fig. 4, the silylium ions can be bound either to the apical or one of the equatorial bromine atoms of the carborane counteranion, with a slight preference of 1.1 kcal mol^–1^ for the apical position in Me_3_Si^+^[CHB_11_H_5_Br_6_]^–^ (**12A′′**). This result is in contrast to the molecular structure in the solid state, which shows the equatorial isomer (*cf.*Fig. 2). We speculate that either packing effects or a statistical preference for the equatorial isomer is the reason for this discrepancy. Notably, the equatorial isomer **12A′** is still 1.8 kcal mol^–1^ lower in energy than the equatorial isomer of MePh_2_Si^+^[CHB_11_H_5_Br_6_]^–^ (**13A′**). The higher ion pairing energy in **12A′** can be ascribed to the low steric demand of Me_3_Si^+^, leading to a closer carborane coordination and to attractive van der Waals interactions between the methyl moieties and the carborane anion. Especially in the apical position, the methyl functionality can interact with the highly polarizable bromine atoms. In contrast, the molecular fit of the sterically more demanding silylium ions Me_2_PhSi^+^ (**6A**) and MePh_2_Si^+^ (**13A**) with the carborane counteranion is less tight, and the ion pairing is therefore slightly less favorable. This trend is reflected in the corresponding Si–Br bond lengths of these silylium carborane salts, which were computed to be shortest in both isomers of Me_3_Si^+^[CHB_11_H_5_Br_6_]^–^ (**12A′** and **12A′′**). Hence, this ion pair is the most stable silylium salt despite the lack of stabilizing phenyl groups. Both isomers of Me_2_PhSi^+^[CHB_11_H_5_Br_6_]^–^ (**6A′** and **6A′′**) are higher in energy than the corresponding MePh_2_Si^+^[CHB_11_H_5_Br_6_]^–^ (**13A′** and **13A′′**), indicating that the stabilization of these silylium carborane salts is determined by a delicate balance of electronic and steric effects. It should also be noted here that the DFT optimized structures for Me_3_Si^+^[CHB_11_H_5_Br_6_]^–^ (**12A′**) and MePh_2_Si^+^[CHB_11_H_5_Br_6_]^–^ (**13A′**) are in good agreement with the corresponding molecular structures obtained by X-ray diffraction analysis (see the ESI† for details).

### Mechanism of the substituent redistribution reaction with MePh_2_SiH

**Fig. 5:**
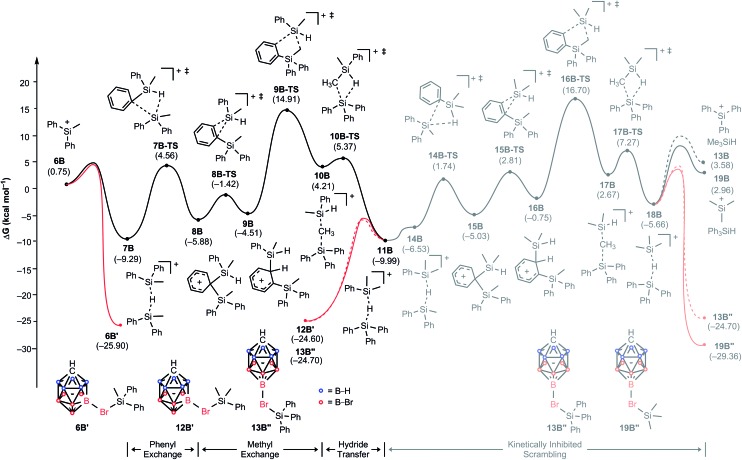
Energy (kcal mol^–1^) profile of the substituent redistribution in the reaction of MePh_2_SiH (**1A**) with Ph_3_C^+^[CHB_11_H_5_Br_6_]^–^ (**2B**). The energies are relative to the starting materials **1B** and **2B**.

To understand why the reaction of MePh_2_SiH with Ph_3_C^+^[CHB_11_H_5_Br_6_]^–^ does not furnish Me_3_Si^+^[CHB_11_H_5_Br_6_]^–^, we constructed again a complete energy profile employing DFT simulations (Fig. 5). The initial hydride transfer of the hydrosilane to the trityl cation has a calculated barrier of 14.3 kcal mol^–1^ (not shown), which is 1.2 kcal mol^–1^ lower in energy compared to the case of Me_2_PhSiH due to the slightly higher hydride donor strength of MePh_2_SiH (see Table S1 in the ESI† for details). The resulting silylium ion MePh_2_Si^+^ (**6B**) with a relative free energy of 0.8 kcal mol^–1^ is almost isoenergetic to the reactant state. Adduct formation with another equivalent of MePh_2_SiH affords hydrosilane-stabilized silylium ion **7B**, which undergoes a phenyl/methyl exchange reaction following a very similar reactivity pattern as described above, leading to scrambled hydride-bridged adduct **11B**. The transformation of **7B** to **11B***via* intermediates **8B**, **9B**, and **10B** is again reversible, since **7B** and **11B** have similar free energies (Δ*G* = 0.7 kcal mol^–1^). As before, the methyl group transfer *via* five-membered transition state **9B-TS** shows the highest barrier, which is 24.2 kcal mol^–1^ relative to **7B**. In this equilibrium, unscrambled MePh_2_Si^+^[CHB_11_H_5_Br_6_]^–^ (**6B′**) with a relative free energy of –25.9 kcal mol^–1^ is predicted to be the major species, followed by scrambled Me_2_PhSi^+^[CHB_11_H_5_Br_6_]^–^ (**12B′**) and Ph_3_Si^+^[CHB_11_H_5_Br_6_]^–^ (**13B′′**), which are basically isoenergetic at –24.6 kcal mol^–1^ and –24.7 kcal mol^–1^, respectively. This finding is in good agreement with the experimental observation of unscrambled MePh_2_Si^+^[CHB_11_H_5_Br_6_]^–^ being the main product of the reaction (*cf.*Table 1, entry 4).^25^

Our calculations suggest that a subsequent methyl exchange reaction leading to Me_3_Si^+^ is unlikely (**11B** → **18B**, gray energy profile in Fig. 5). The transition state for this methyl group transfer, **16B-TS**, is located 26.7 kcal mol^–1^ relative to **11B**, which is 1.8 kcal mol^–1^ higher in energy than the barrier of the backward reaction *via* transition state **9B-TS**. Consequently, the reaction of MePh_2_SiH with Ph_3_C^+^[CHB_11_H_5_Br_6_]^–^ stops at the above-mentioned mixture of silicon cations rather than undergoing exhaustive substituent redistribution to furnish low energy Me_3_Si^+^[CHB_11_H_5_Br_6_]^–^.

**Scheme 3:**
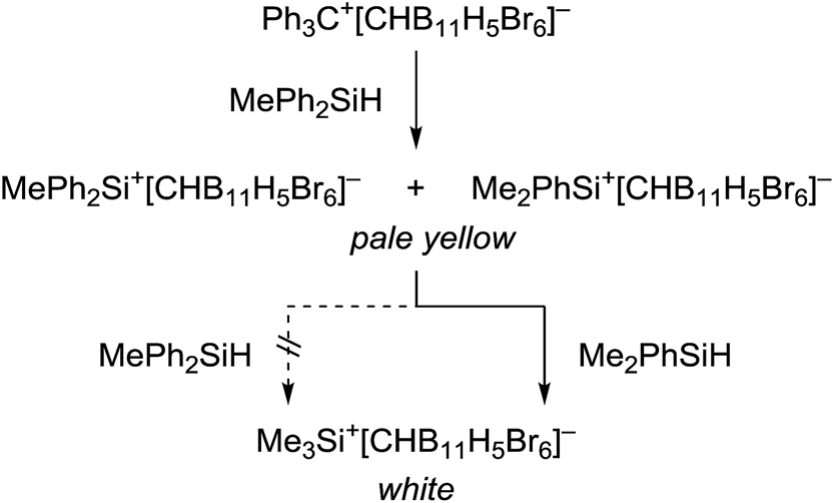
Probing the kinetic inhibition in the substituent redistribution reaction with MePh_2_SiH.

This kinetic inhibition was further proven by another mechanistic control experiment (Scheme 3). When a mixture of Ph_3_C^+^[CHB_11_H_5_Br_6_]^–^ and MePh_2_SiH in toluene was stirred overnight at room temperature, a pale yellow suspension was obtained, which is characteristic of silylium ions with aromatic substituents (*cf.*Table 1, entry 4). Addition of less bulky Me_2_PhSiH to this mixture resulted in a quick decolorization and formation of a white suspension. NMR spectroscopic analysis of the solid now confirmed exclusive formation of Me_3_Si^+^[CHB_11_H_5_Br_6_]^–^.

### Scope of the substituent redistribution reaction

The hydride abstraction from various dialkyl(phenyl)silanes with Ph_3_C^+^[CHB_11_H_5_Br_6_]^–^ finally revealed that the redistribution reaction is not restricted to methyl groups (Table 2). Although Et_2_PhSiH reacted much slower compared to Me_2_PhSiH, exclusive formation of trialkylsilylium ion Et_3_Si^+^[CHB_11_H_5_Br_6_]^–^ was observed (entries 1 and 2). Employing more bulky iPr_2_PhSiH led to clean generation of unscrambled dialkyl(aryl)silylium ion iPr_2_PhSi^+^[CHB_11_H_5_Br_6_]^–^, as verified by X-ray crystallography (entry 3; see the ESI† for the molecular structure of iPr_2_PhSi^+^[CHB_11_H_5_Br_6_]^–^).^15^ These results are in accordance with our calculations, predicting high energy barriers for the transfer of bulky alkyl groups. Sterically even more shielded *t*Bu_2_PhSiH then completely thwarted the hydride abstraction, and only the trityl salt was recovered from the reaction mixture (entry 4).

**Table 2:** Silylium ion generation from hydrosilanes of type R_2_PhSiH

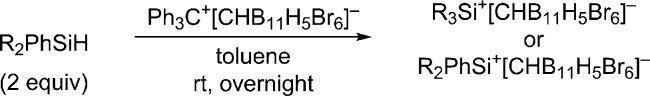			
Entry^*a*^	R	Si^+^	*δ*(^29^Si)^*b*^ [ppm]
1	Me	Me_3_Si^+^	93
2^*c*^	Et	Et_3_Si^+^	100
3	iPr	iPr_2_PhSi^+^	76
4	*t*Bu	—^*d*^	—

^*a*^All reactions were performed according to GP 2. See the ESI for details.

^*b*^Measured in *o*-Cl_2_C_6_D_4_.

^*c*^With 4 equiv. of Et_2_PhSiH and 7 days reaction time.

^*d*^No reaction; only Ph_3_C^+^[CHB_11_H_5_Br_6_]^–^ was recovered.

To investigate whether the phenyl group in Me_2_PhSiH can be replaced by other ‘leaving groups’, we also tested a benzyl and an alkyl substituent in Me_2_RSiH (Table 3). As in the case of Me_2_PhSiH (entry 1), clean formation of Me_3_Si^+^[CHB_11_H_5_Br_6_]^–^ was observed with Me_2_BnSiH (entry 2), showing that the phenyl group is not essential for the exchange process. In contrast, the bulky *tert*-butyl group in Me_2_*t*BuSiH completely prevented substituent redistribution, and silylium ion Me_2_*t*BuSi^+^[CHB_11_H_5_Br_6_]^–^ was formed as the only product (entry 3). This result again demonstrates that the intermolecular substituent exchange reaction is sensitive towards sterically demanding alkyl groups (*cf.* entry 3 in Table 2).

**Table 3:** Silylium ion generation from hydrosilanes of type Me_2_RSiH

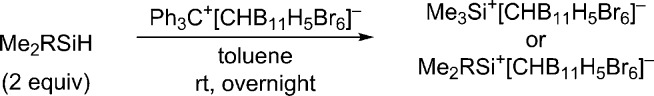			
Entry^*a*^	R	Si^+^	*δ*(^29^Si)^*b*^ [ppm]
1	Ph	Me_3_Si^+^	93
2	Bn	Me_3_Si^+^	93
3	*t*Bu	Me_2_*t*BuSi^+^	98

^*a*^All reactions were performed according to GP 2. See the ESI for details.

^*b*^Measured in *o*-Cl_2_C_6_D_4_.

## Conclusion

It has been known for decades that silylium ions can undergo redistribution reactions of their substituents.^8^ The present combined experimental and detailed computational study finally provides a full mechanistic picture of this phenomenon. The mechanism involves a series of phenyl and alkyl exchange reactions, the latter being calculated to be the energetically most demanding steps. While the transfer of phenyl groups proceeds *via* common four-centered transition states, the corresponding alkyl exchange was found to pass through unusual five-membered transition states. These are accessible after 1,2-silyl migration at the stage of the intermediate disilylated arenium ions.

Additionally, our DFT calculations revealed that the silicon cations are significantly more stabilized by ion pair formation with the carborane counteranion (R_3_Si^+^[CHB_11_H_5_Br_6_]^–^) than by formation of toluenium (R_3_Si(toluene)^+^[CHB_11_H_5_Br_6_]^–^) or hydrosilane-stabilized silylium ions ([R_3_Si–H–SiR_3_]^+^[CHB_11_H_5_Br_6_]^–^). More importantly, purely aliphatic silylium carboranes with small substituents, *i.e.*, methyl or ethyl groups, were found to be distinctly lower in energy than the corresponding mixed aliphatic/aromatic or purely aromatic silylium ion pairs as a result of stronger attractive interactions (Δ*G* ≥ 2.9 kcal mol^–1^ for R = Me). These energy differences account for the highly selective formation of Me_3_Si^+^[CHB_11_H_5_Br_6_]^–^ and Et_3_Si^+^[CHB_11_H_5_Br_6_]^–^ from the reaction of the corresponding hydrosilanes R_2_PhSiH (R = Me, Et) with Ph_3_C^+^[CHB_11_H_5_Br_6_]^–^ under thermodynamic control.

The phenyl group in Me_2_PhSiH turned out to be replaceable by other ‘leaving groups’, such as a benzyl or even a sterically demanding C_6_Me_5_ group. However, two alkyl groups must be preinstalled in the hydrosilane starting material to steer the reaction towards formation of Me_3_Si^+^[CHB_11_H_5_Br_6_]^–^. In contrast, hydride abstraction from MePh_2_SiH with only one alkyl substituent leads to a mixture of different silylium ions, as exhaustive scrambling to Me_3_Si^+^ is kinetically inhibited. Exchanging the phenyl groups in MePh_2_SiH by 2,6-disubstituted aryl groups (*e.g.* C_6_Me_5_) eventually provides access to sterically congested triarylsilylium ions, as previously demonstrated by Müller and co-workers.^10^

These general trends provide a solid foundation for the mechanistic understanding of the substituent redistribution of silylium ions, thereby enabling the prediction of the outcome of these exchange reactions. Thus, this process can be used as a reliable synthetic route not only to triaryl- but also to trialkylsilylium ions by deliberate choice of the hydrosilane and counteranion of the trityl salt.

## Conflicts of interest

There are no conflicts to declare.

## Supplementary Material

Supplementary information

Crystal structure data
