# Supplementary material for: Thermodynamic versus kinetic control in substituent redistribution reactions of silylium ions steered by the counteranion
Source: Chem Sci. 2018 May 21;9(25):5600–7. doi: 10.1039/c8sc01833b (PMC6048699; doi:10.1039/c8sc01833b)
Supplement: Supplementary file 1 [file SC-009-C8SC01833B-s001.pdf]

Electronic Supplementary Material (ESI) for Chemical Science.
This journal is © The Royal Society of Chemistry 2018

Thermodynamic versus kinetic control in substituent redistribution reactions of silylium ions steered by the counteranion

Lukas Omann,[a] Bimal Pudasaini,[b] Elisabeth Irran,[a] Hendrik F. T. Klare,[a] Mu-Hyun Baik*,[b,c] and Martin Oestreich*,[a]

[a]*Institut für Chemie, Technische Universität Berlin,*
Strasse des 17. Juni 115, 10623 Berlin, Germany
[b]*Center for Catalytic Hydrocarbon Functionalizations, Institute for Basic Science (IBS)*
Daejon, 34141, Republic of Korea
[c]*Department of Chemistry, Korea Advanced Institute of Science and Technology (KAIST),*
Daejon, 34141, Republic of Korea

mbaik2805@kaist.ac.kr
martin.oestreich@tu-berlin.de

Supporting Information

Table of Contents

**1**	**General Information**	**S4**
**2**	**Experimental Details for the Synthesis of Various Hydrosilanes**	**S5**
2.1	Dimethyl(2,3,4,5,6-pentamethylphenyl)silane	S5
2.2	Benzyl(dimethyl)silane	S6
2.3	Diisopropyl(phenyl)silane	S7
2.4	Di-*tert*-butyl(phenyl)silane	S7
**3**	**Experimental Details for the Synthesis of $Ph_3C^+[CHB_{11}H_5Br_6]^–$**	**S8**
3.1	$Ag^+[CHB_{11}H_5Br_6]^–$	S8
3.2	$Ph_3C^+[CHB_{11}H_5Br_6]^–$	S9
**4**	**General Procedures for the Generation of Silylium Ions**	**S10**
4.1	General Procedure for the Generation of Silylium Ions using $Ph_3C^+[B(C_6F_5)_4]^–$ (GP 1)	S10
4.2	General Procedure for the Generation of Silylium Ions using $Ph_3C^+[CHB_{11}H_5Br_6]^–$ (GP 2)	S10
**5**	**Experimental Details for the Hydride Abstraction of Various Hydrosilanes using $Ph_3C^+[B(C_6F_5)_4]^–$ and $Ph_3C^+[CHB_{11}H_5Br_6]^–$**	**S11**
5.1	Reaction of $Me(C_6Me_5)_2SiH$ with $Ph_3C^+[B(C_6F_5)_4]^–$: Generation of $(C_6Me_5)_3Si^+[B(C_6F_5)_4]^–$ (Table 1, Entry 1)	S11

5.2 Reaction of $Me(C_6Me_5)_2SiH$ with $Ph_3C^+[CHB_{11}H_5Br_6]^-$: Generation of $(C_6Me_5)_3Si^+[CHB_{11}H_5Br_6]^-$ (Table 1, Entry 2) S12

5.3 Reaction of $MePh_2SiH$ with $Ph_3C^+[B(C_6F_5)_4]^-$ (Table 1, Entry 3) S12

5.4 Reaction of $MePh_2SiH$ with $Ph_3C^+[CHB_{11}H_5Br_6]^-$: Generation of $MePh_2Si^+[CHB_{11}H_5Br_6]^-$ with Small Amounts of $Me_2PhSi^+[CHB_{11}H_5Br_6]^-$ (Table 1, Entry 4) S13

5.5 Reaction of Me_2PhSiH with $Ph_3C^+[B(C_6F_5)_4]^-$ (Table 1, Entry 5) S14

5.6 Reaction of Me_2PhSiH with $Ph_3C^+[CHB_{11}H_5Br_6]^-$: Generation of $Me_3Si^+[CHB_{11}H_5Br_6]^-$ (Table 1, Entry 6) S14

5.7 Reaction of $Me_2(C_6Me_5)SiH$ with $Ph_3C^+[B(C_6F_5)_4]^-$: Generation of $(C_6Me_5)_3Si^+[B(C_6F_5)_4]^-$ (Table 1, Entry 7) S15

5.8 Reaction of $Me_2(C_6Me_5)SiH$ with $Ph_3C^+[CHB_{11}H_5Br_6]^-$: Generation of $Me_3Si^+[CHB_{11}H_5Br_6]^-$ (Table 1, Entry 8) S16

5.9 Reaction of Et_2PhSiH with $Ph_3C^+[CHB_{11}H_5Br_6]^-$: Generation of $Et_3Si^+[CHB_{11}H_5Br_6]^-$ (Table 3, Entry 2) S16

5.10 Independent Synthesis of $Et_3Si^+[CHB_{11}H_5Br_6]^-$ from the Reaction of Et_3SiH with $Ph_3C^+[CHB_{11}H_5Br_6]^-$ S17

5.11 Reaction of *i*Pr_2PhSiH with $Ph_3C^+[CHB_{11}H_5Br_6]^-$: Generation of *i*$Pr_2PhSi^+[CHB_{11}H_5Br_6]^-$ (Table 3, Entry 3) S18

5.12 Reaction of *t*Bu_2PhSiH with $Ph_3C^+[CHB_{11}H_5Br_6]^-$ (Table 3, Entry 4) S18

5.13 Reaction of Me_2BnSiH with $Ph_3C^+[CHB_{11}H_5Br_6]^-$: Generation of $Me_3Si^+[CHB_{11}H_5Br_6]^-$ (Table 2, Entry 2) S19

5.14 Reaction of Me_2*t*BuSiH with $Ph_3C^+[CHB_{11}H_5Br_6]^-$: Generation of Me_2*t*$BuSi^+[CHB_{11}H_5Br_6]^-$ (Table 2, Entry 3) S19

6 Mechanistic Control Experiments S20

6.1 Influence of the Carborane Counteranion on the Selectivity of the Trimethylsilylium Ion Formation (Scheme 2) S20

6.2 Influence of the Borate Counteranion on the Selectivity of the Trimethylsilylium Ion Formation (Scheme 2) S20

6.3 Addition of Me_2PhSiH to a Mixture of $MePh_2Si^+[CHB_{11}H_5Br_6]^-$ and $Me_2PhSi^+[CHB_{11}H_5Br_6]^-$: Generation of $Me_3Si^+[CHB_{11}H_5Br_6]^-$ (Scheme 3) S21

7 NMR Spectra S22

8 Crystallographic Data S81

9 Computational Data S85

9.1 Evaluation of Silylium Ion Stability S85

9.2 Silylium Ion Binding to Various Nucleophiles S86

9.3 Evaluation of Methyl Transfer Mechanism S88

9.4 Geometries of the Bridged Structures S89
9.5 Energy Data S90
9.6 Coordinates of the Calculated Structures S93
9.7 Vibrational Frequencies (cm^{-1}) of the Calculated Structures S148
10 References S178

1 General Information

All reactions were performed in flame-dried glassware using an *MBraun* glovebox or conventional Schlenk techniques under a static pressure of argon (glovebox) or nitrogen unless otherwise stated. Solvents for chromatography and extraction were distilled prior to use. Et_2O and THF were dried over potassium/benzophenone and freshly distilled prior to use. Toluene was dried over sodium/benzophenone, distilled, degassed by three freeze-pump-thaw cycles, and stored in a glovebox over thermally activated 4 Å molecular sieves. Acetonitrile (99.9+%, extra dry, AcroSeal®) was purchased from *Acros Organics* and used as received. *n*-Pentane and *n*-hexane were obtained from an *MBraun* solvent system, degassed by three freeze-pump-thaw cycles and stored in a glovebox over thermally activated 4 Å molecular sieves. Hydrosilanes Me_2PhSiH, $MePh_2SiH$, and Me_2tBuSiH were dried over CaH_2, distilled, degassed by three freeze-pump-thaw cycles and stored in a glovebox over thermally activated 4 Å molecular sieves. Hydrosilanes $Me(C_6Me_5)_2SiH$[S1] and Et_2PhSiH[S2] were synthesized according to reported procedures. The concentration of *n*-butyllithium solutions was determined by titration using Suffert's reagent.[S3] *N*,*N*,*N'*,*N'*-Tetramethylethane-1,2-diamine (TMEDA) was dried over sodium, distilled, and stored under nitrogen. $Cs^+[CHB_{11}H_{11}]^-$ was purchased from *KatChem*, used as received, and converted into $Cs^+[CHB_{11}H_5Br_6]^-$ according to a reported procedure.[S4] Trityl bromide (Ph_3CBr) was purchased from *TCI*, stored in a glovebox, and used as received. $Ph_3C^+[B(C_6F_5)_4]^-$ was synthesized according to a reported procedure.[S5] Analytical thin-layer chromatography (TLC) was performed on silica gel 60 F254 glass plates. Flash column chromatography was performed on silica gel 60 (40–63 µm, 230–400 mesh, ASTM) by *Grace* using the indicated solvents. ^1H, ^{11}B, ^{13}C, ^{19}F, and ^{29}Si NMR spectra were recorded in CD_2Cl_2, C_6D_6, or *o*-$Cl_2C_6D_4$ on a *Bruker* AV500 or AV700 instrument using valve NMR tubes by *Norell*. *o*-$Cl_2C_6D_4$ was degassed by three freeze-pump-thaw cycles and stored in a glovebox over thermally activated 4 Å molecular sieves. Chemical shifts are reported in parts per million (ppm) and are referenced to the residual solvent resonance as the internal standard ($CDHCl_2$: δ 5.32 ppm for ^1H NMR and CD_2Cl_2: δ 53.84 ppm for ^{13}C NMR; C_6D_5H: δ 7.16 ppm for ^1H NMR and C_6D_6: δ 128.06 ppm for ^{13}C NMR; *o*-$Cl_2C_6D_3H$: δ 6.94 and 7.20 ppm for ^1H NMR and *o*-$Cl_2C_6D_4$: δ 127.1, 130.1, and 132.5 ppm for ^{13}C NMR). ^{11}B, ^{19}F, and ^{29}Si NMR spectra are referenced in compliance with the unified scale for NMR chemical shifts as recommended by the IUPAC stating the chemical shift relative to $BF_3{\cdot}Et_2O$, CCl_3F, and TMS, respectively.[S6] Data are reported as follows: chemical shift, multiplicity (s = singlet, d = doublet, t = triplet, q = quartet, sept = septet, m = multiplet, m_c = centrosymmetric multiplet, br = broad signal), coupling constants (Hz), and integration. Mass spectrometry (MS) data were measured with the GC-MS system 5975C from *Agilent Technologies* by electron ionization (EI). The GC-MS

was equipped with a HP-5MS capillary column (30 m × 0.32 mm, 0.25 µm film thickness) by *Agilent Technologies*. Analyses were performed using the following program: He carrier gas, injection temperature: 300 °C, detector temperature: 300 °C, flow rate: 0.8 mL·min^{-1}; temperature program: start temperature: 40 °C, heating rate 10 °C·min^{-1}, end temperature 280 °C for 10 min. Infrared (IR) spectra were recorded on a *Jasco* FT/IR-4100 spectrometer equipped with an ATR unit, and the signals are reported in wavenumbers (cm^{-1}). Melting points (m.p.) were determined with a *Stuart Scientific* SMP20 instrument and are not corrected. High resolution mass spectra (HRMS) were obtained from the *Analytical Facility* at the *Institut für Chemie, Technische Universität Berlin*.

2 Experimental Details for the Synthesis of Various Hydrosilanes

2.1 Dimethyl(2,3,4,5,6-pentamethylphenyl)silane

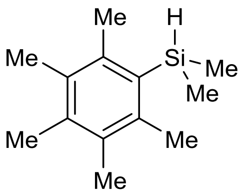

$C_{13}H_{22}Si$
M = 206.40 g/mol

To a solution of pentamethylphenyl bromide (1.14 g, 5.02 mmol, 1.00 equiv) and TMEDA (1.14 mL, 878 mg, 7.55 mmol, 1.51 equiv) in THF (100 mL), *n*-BuLi (2.68 M in hexanes, 2.80 mL, 7.50 mmol, 1.50 equiv) was added dropwise at –78 °C. After stirring for 1 h, chloro(dimethyl)silane (1.67 mL, 1.42 g, 15.0 mmol, 3.00 equiv) was added, and the mixture was allowed to warm to room temperature overnight. After heating for 1 h at 50 °C, H_2O (~30 mL) was added. The phases were separated, and the aqueous phase was extracted with *tert*-butyl methyl ether (~3 × 30 mL). The combined organic phases were dried over $MgSO_4$, and the solvents were removed under reduced pressure. Purification by flash column chromatography on silica gel using cyclohexane as eluent afforded the desired product (528 mg, 51%) as a white solid.

R_f = 0.67 (cyclohexane). **m.p.** = 51 °C. **IR** (ATR): ṽ = 3003, 2905, 2176 (Si–H), 1738, 1557, 1448, 1389, 1276, 1257, 1242, 1223, 1089, 1061, 1007, 948, 895, 831, 816, 763, 728, 694, 648 cm^{-1}. **^{1}H NMR** (500 MHz, C_6D_6, 300 K): δ 0.43 (d, ^3J(H,H) = 4.1 Hz, 6H, SiC*H*$_3$), 2.04 (s, 6H, *m*-C*H*$_3$), 2.08 (s, 3H, *p*-C*H*$_3$), 2.36 (s, 6H, *o*-C*H*$_3$), 5.07 (sept, ^3J(H,H) = 4.0 Hz, 1H, Si*H*) ppm. **^{13}C{^{1}H} NMR** (126 MHz, C_6D_6, 300 K): δ –1.3 (Si*C*H$_3$), 16.5 (*m*-*C*H$_3$), 16.9 (*p*-*C*H$_3$), 21.8 (*o*-*C*H$_3$), 132.3 (*m*-C$_{Ar}$), 132.7 (*i*-C$_{Ar}$), 136.2 (*p*-C$_{Ar}$), 139.5 (*o*-C$_{Ar}$) ppm. **^{29}Si DEPT NMR** (99

MHz, C_6D_6, 300 K, optimized for J = 200 Hz): δ –25.8 ppm. **HRMS** (EI): calculated for $C_{13}H_{22}Si^{•+}$ $[M]^{•+}$: 206.1485; found 206.1480.

2.2 Benzyl(dimethyl)silane

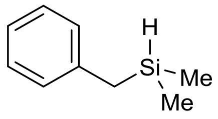

$C_9H_{14}Si$
M = 150.30 g/mol

To a suspension of magnesium turnings (401 mg, 16.5 mmol, 1.83 equiv) in THF (5 mL), a few drops of 1,2-dibromoethane and chloro(dimethyl)silane (1.00 mL, 852 mg, 9.00 mmol, 1.00 equiv) were added. While stirring at room temperature, a solution of benzyl bromide (1.39 mL, 2.00 g, 11.7 mmol, 1.30 equiv) in THF (35 mL) was added dropwise over a period of 4 h. The reaction was quenched with saturated aqueous NH_4Cl solution (~20 mL). The phases were separated, and the aqueous phase was extracted with *tert*-butyl methyl ether (~3 × 30 mL). The combined organic phases were dried over $MgSO_4$, and the solvents were removed under reduced pressure. Purification by Kugelrohr distillation (25 °C, 10^{-3} mbar) afforded the desired product (680 mg, 50%) as a colorless oil.

R_f = 0.63 (cyclohexane). **IR** (ATR): ṽ = 3081, 3061, 3024, 2958, 2895, 2113 (Si–H), 1600, 1493, 1452, 1409, 1249, 1206, 1155, 1056, 1031, 879, 836, 817, 781, 770, 839, 696, 619 cm^{-1}. **^{1}H NMR** (500 MHz, C_6D_6, 300 K): δ –0.06 (d, ^3J(H,H) = 3.6 Hz, 6H, SiCH_3), 1.97 (d, ^3J(H,H) = 3.2 Hz, 2H, SiCH_2Ph), 4.12 (m_c, 1H, Si*H*), 6.95–6.96 (m, 2H, *o*-H_{Ar}), 7.01 (t, ^3J(H,H) = 7.4 Hz, 1H, *p*-H_{Ar}), 7.13–7.16 (m, 2H, *m*-H_{Ar}) ppm. **^{13}C{^{1}H} NMR** (126 MHz, C_6D_6, 300 K): δ –4.7 (Si*C*H_3), 24.3 (Si*C*H_2Ph), 124.7 (*p*-C_{Ar}), 128.5 (*o*-C_{Ar}), 128.7 (*m*-C_{Ar}), 140.1 (*i*-C_{Ar}) ppm. **^{29}Si DEPT NMR** (99 MHz, C_6D_6, 300 K, optimized for J = 200 Hz): δ –12.3 ppm. **HRMS** (EI): calculated for $C_9H_{14}Si^{•+}$ $[M]^{•+}$: 150.0859; found 150.0852.

2.3 Diisopropyl(phenyl)silane

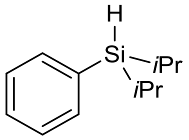

$C_{12}H_{20}Si$
M = 192.13 g/mol

To a solution of diisopropyl(chloro)silane (1.38 mL, 1.20 g, 7.98 mmol, 1.00 equiv) in Et_2O (32 mL), phenyllithium (~1.9 M in *n*-Bu_2O, 6.3 mL, ~12 mmol, ~1.5 equiv) was added dropwise at 0 °C. After stirring for 1 h, H_2O (~20 mL) was added. The phases were separated, and the aqueous phase was extracted with *tert*-butyl methyl ether (~3 × 30 mL). The combined organic phases were dried over $MgSO_4$, and the solvents were removed under reduced pressure. Purification by flash column chromatography on silica gel using cyclohexane as eluent afforded the desired product (1.08 g, 70%) as a colorless oil.

R_f = 0.75 (cyclohexane). **IR** (ATR): ṽ = 3069, 2940, 2890, 2863, 2099 (Si–H), 1461, 1428, 1383, 1365, 1238, 1111, 1065, 998, 918, 879, 800, 781, 732, 699, 657, 607 cm^{-1}. **^{1}H NMR** (500 MHz, C_6D_6, 298 K): δ 1.00 (d, ^3J(H,H) = 7.1 Hz, 6H, SiCHCH_3), 1.07 (d, ^3J(H,H) = 7.0 Hz, 6H, SiCHCH_3), 1.09–1.19 (m, 2H, SiC*H*CH_3), 4.21 (t, ^3J(H,H) = 3.0 Hz, 1H, Si*H*), 7.18–7.21 (m, 3H, *m*-H_{Ar}, *p*-H_{Ar}), 7.48–7.52 (m, 2H, *o*-H_{Ar}) ppm. **^{13}C{^{1}H} NMR** (126 MHz, C_6D_6, 298 K): δ 11.0 (Si*C*HCH_3), 18.7 (SiCH*C*H_3), 18.9 (SiCH*C*H_3), 128.1 (*p*-C_{Ar}), 129.5 (*m*-C_{Ar}), 134.2 (*i*-C_{Ar}), 135.8 (*o*-C_{Ar}) ppm. **^{29}Si DEPT NMR** (99 MHz, C_6D_6, 298 K, optimized for *J* = 200 Hz): δ 5.9 ppm. **HRMS** (EI): calculated for $C_{12}H_{20}Si^{\bullet+}$ $[M]^{\bullet+}$: 192.1329; found 192.1320.

2.4 Di-*tert*-butyl(phenyl)silane

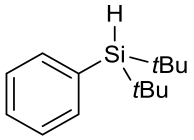

$C_{14}H_{24}Si$
M = 220.43 g/mol

To a solution of di-*tert*-butyl(chloro)silane (1.62 mL, 1.43 g, 8.01 mmol, 1.00 equiv) in THF (32 mL), phenyllithium (~1.9 M in *n*-Bu_2O, 6.3 mL, ~12 mmol, ~1.5 equiv) was added dropwise at 0 °C. After stirring for 1 h, H_2O (~20 mL) was added. The phases were separated, and the aqueous phase was extracted with *tert*-butyl methyl ether (~3×30 mL). The combined organic phases were dried over $MgSO_4$, and the solvents were removed

under reduced pressure. Purification by flash column chromatography on silica gel using cyclohexane as eluent afforded the desired product (1.32 g, 75%) as a colorless oil.

R*f* = 0.80 (cyclohexane). **IR** (ATR): ṽ = 3069, 2959, 2938, 2890, 2856, 2097 (Si–H), 1738, 1468, 1427, 1388, 1363, 1217, 1107, 1066, 1012, 935, 800, 734, 700, 619 cm^{-1}. **^{1}H NMR** (500 MHz, C_6D_6, 300 K): δ 1.07 (s, 18H, SiCC*H*$_3$), 4.07 (s, 1H, Si*H*), 7.17–7.20 (m, 3H, *m*-H_{Ar}, *p*-H_{Ar}), 7.59–7.61 (m, 2H, *o*-H_{Ar}) ppm. **^{13}C{^{1}H} NMR** (126 MHz, C_6D_6, 300 K): δ 19.1 (Si*C*CH$_3$), 29.1 (SiC*C*H$_3$), 128.0 (*p*-C_{Ar}), 129.4 (*m*-C_{Ar}), 135.6 (*i*-C_{Ar}), 136.1 (*o*-C_{Ar}) ppm. **^{29}Si DEPT NMR** (99 MHz, C_6D_6, 298 K, optimized for *J* = 200 Hz): δ 13.1 ppm. **HRMS** (EI): calculated for $C_{14}H_{24}Si^{\bullet+}$ $[M]^+$: 220.1642; found 220.1648.

3 Experimental Details for the Synthesis of $Ph_3C^+[CHB_{11}H_5Br_6]^-$

3.1 $Ag^+[CHB_{11}H_5Br_6]^-$

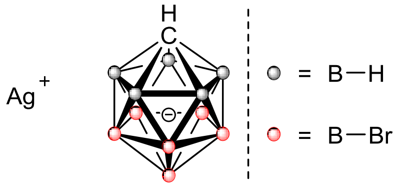

$CH_6B_{11}Br_6Ag$
M = 724.28 g/mol

According to a modified reported procedure,[S7] $Cs^+[CHB_{11}H_5Br_6]^-$ (2.50 g, 3.34 mmol, 1.00 equiv) was dissolved in boiling distilled water (100 mL). One drop of concentrated nitric acid was added to ensure a slightly acidic solution. Under exclusion of light, a solution of $AgNO_3$ (680 mg, 4.00 mmol, 1.20 equiv) in distilled water (2 mL) was added, resulting in formation of a white precipitate. After boiling for additional 15 min, the suspension was allowed to cool to room temperature. The solid was collected by filtration on a fine porosity glass frit, washed with distilled water (~10 mL) and dried for 3 days at 90 °C (10^{-3} mbar) to give the desired product (2.21 g, 91%) as a white powder, which was directly used in the next reaction step.

3.2 $Ph_3C^+[CHB_{11}H_5Br_6]^-$

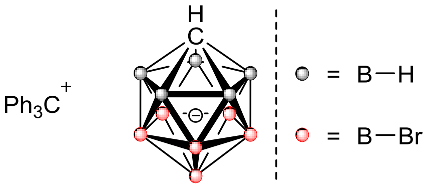

$C_{20}H_{21}B_{11}Br_6$
M = 859.74 g/mol

According to a modified reported procedure,[S8] $Ag^+[CHB_{11}H_5Br_6]^-$ (2.18 g, 3.01 mmol, 1.00 equiv) and Ph_3CBr (972 mg, 3.01 mmol, 1.00 equiv) were weighed into a Schlenk flask in a glovebox. Outside the glovebox, toluene (30 mL) and acetonitrile (50 mL) were added. After stirring for 2 h at room temperature under exclusion of light, the formed AgBr salt was removed by filtration under nitrogen and washed with small portions of toluene/acetonitrile (~10 mL; v:v = 4:1). The filtrate was concentrated under reduced pressure to a few mL, resulting in precipitation of an orange solid. In a glovebox, the solid was collected by filtration and washed with toluene (~2 mL) and *n*-pentane (~5 mL). After drying overnight at 80 °C (10^{-3} mbar), the desired product (2.44 g, 94%) was obtained as a yellow-orange solid.

^{1}H NMR (500 MHz, CD_2Cl_2, 300 K): δ ~1.6–3.1 (br m, 6H, $[CHB_{11}H_5Br_6]^-$), 7.69–7.72 (m, 6H, *o*-H_{Ar}), 7.91–7.95 (m, 6H, *m*-H_{Ar}), 8.30 (tt, 3H, ^3J(H,H) = 7.5 Hz, ^4J(H,H) = 1.2 Hz, *p*-H_{Ar}) ppm. **^{11}B NMR** (161 MHz, CD_2Cl_2, 300 K): δ –20.2 (d, ^1J(B,H) = 167 Hz), –9.9 (s), –1.8 (s) ppm. **^{13}C NMR** (126 MHz, CD_2Cl_2, 300 K): δ 41.6 ($[CHB_{11}H_5Br_6]^-$), 131.2 (*m*-C_{Ar}), 140.4 (*i*-C_{Ar}), 143.3 (*o*-C_{Ar}), 144.0 (*p*-C_{Ar}), 211.1 (CPh_3) ppm.

4 General Procedures for the Generation of Silylium Ions

4.1 General Procedure for the Generation of Silylium Ions using $Ph_3C^+[B(C_6F_5)_4]^-$ (GP 1)

In a glovebox, a solution of the corresponding hydrosilane (0.10–0.20 mmol, 4.0 equiv) in C_6D_6 (~10 drops) is added to a solution of $Ph_3C^+[B(C_6F_5)_4]^-$ (23–46 mg, 0.025–0.050 mmol, 1.0 equiv) in C_6D_6 (~10 drops), resulting in the formation of a biphasic mixture. After stirring for 60 min at room temperature, the upper phase is removed, and the oily bottom phase is washed with C_6D_6 (~3 × 10 drops). The residue is dissolved in *o*-$Cl_2C_6D_4$ (0.60 mL), transferred into a valve NMR tube, and directly subjected to NMR spectroscopic analysis.

4.2 General Procedure for the Generation of Silylium Ions using $Ph_3C^+[CHB_{11}H_5Br_6]^-$ (GP 2)

In a glovebox, a solution of the corresponding hydrosilane (0.046–0.23 mmol, 2.0 or 4.0 equiv) in toluene (~5–10 drops) is added to a suspension of $Ph_3C^+[CHB_{11}H_5Br_6]^-$ (20–50 mg, 0.023–0.058 mmol, 1.0 equiv) in toluene (~5–10 drops). After stirring the reaction mixture at the indicated temperature for a certain time, *n*-pentane (~1 mL) is added to ensure full precipitation of all ionic components. The solid is collected by filtration, washed with *n*-pentane (~3 × 0.5 mL) and then briefly dried under vacuum. Pale yellow solids are obtained in case of silylium ions with aromatic substituents, whereas those with purely aliphatic groups appear as white solids. *o*-$Cl_2C_6D_4$ (0.60 mL) is used to transfer the products into a valve NMR tube, which is directly subjected to NMR spectroscopic analysis.

5 Experimental Details for the Hydride Abstraction of Various Hydrosilanes using $Ph_3C^+[B(C_6F_5)_4]^-$ and $Ph_3C^+[CHB_{11}H_5Br_6]^-$

5.1 Reaction of $Me(C_6Me_5)_2SiH$ with $Ph_3C^+[B(C_6F_5)_4]^-$: Generation of $(C_6Me_5)_3Si^+[B(C_6F_5)_4]^-$ (Table 1, Entry 1)

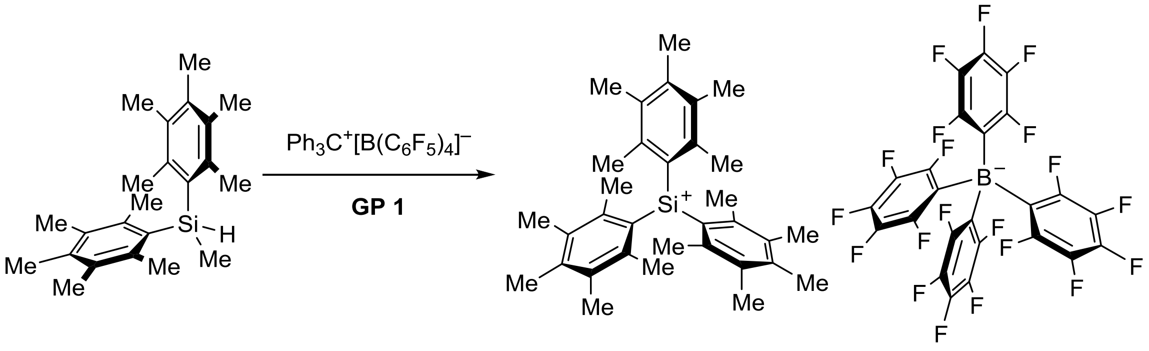

According to **GP 1**, $Ph_3C^+[B(C_6F_5)_4]^-$ (23 mg, 0.025 mmol, 1.0 equiv) was treated with $Me(C_6Me_5)_2SiH$ (34 mg, 0.10 mmol, 4.0 equiv). NMR spectroscopic analysis revealed clean formation of $(C_6Me_5)_3Si^+[B(C_6F_5)_4]^-$.

^{1}H NMR (500 MHz, *o*-$Cl_2C_6D_4$, 298 K): δ 1.99 (s, 18H, *m*-C*H*$_3$), 2.07 (s, 9H, *p*-C*H*$_3$), 2.37 (s, 18H, *o*-C*H*$_3$) ppm. **^{11}B{^{1}H} NMR** (161 MHz, *o*-$Cl_2C_6D_4$, 298 K): δ –16.1 (s) ppm. **^{13}C{^{1}H} NMR** (126 MHz, *o*-$Cl_2C_6D_4$, 298 K): δ 15.7 (*m*-CH_3), 17.4 (*p*-CH_3), 24.3 (*o*-CH_3), 134.2 (*i*-C_{Ar}), 135.9 (*m*-C_{Ar}), 138.3 (*o*-C_{Ar}), 146.1 (*p*-C_{Ar}) ppm. The signals of the $[B(C_6F_5)_4]^-$ counteranion were not detected. **^{19}F NMR** (471 MHz, *o*-$Cl_2C_6D_4$, 298 K): δ –166.2 (t, ^3J(F,F) = 17 Hz), –162.4 (t, ^3J(F,F) = 20 Hz), –131.8 (s) ppm. **^{1}H/^{29}Si HMQC NMR** (500/99 MHz, *o*-$Cl_2C_6D_4$, 298 K, optimized for J = 3 Hz): δ 1.99/217.0, 2.37/217.0 ppm.

5.2 Reaction of $Me(C_6Me_5)_2SiH$ with $Ph_3C^+[CHB_{11}H_5Br_6]^-$: Generation of $(C_6Me_5)_3Si^+[CHB_{11}H_5Br_6]^-$ (Table 1, Entry 2)

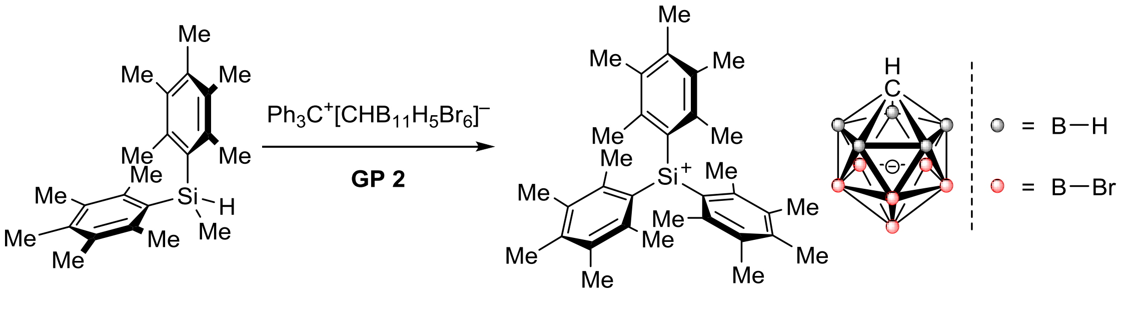

According to **GP 2**, $Ph_3C^+[CHB_{11}H_5Br_6]^-$ (20 mg, 0.023 mmol, 1.0 equiv) was treated with $Me(C_6Me_5)_2SiH$ (32 mg, 0.095 mmol, 4.1 equiv). The reaction was stopped after stirring for 22 h at room temperature. NMR spectroscopic analysis revealed clean formation of $(C_6Me_5)_3Si^+[CHB_{11}H_5Br_6]^-$.

^{1}H NMR (700 MHz, *o*-$Cl_2C_6D_4$, 298 K): δ 1.96 (s, 18H, *m*-CH_3), 2.02 (s, 9H, *p*-CH_3), ~2.1–3.2 (br m, 24H, *o*-CH_3, [C*H*B_{11}*H*$_5Br_6]^-$) ppm. **^{11}B NMR** (224 MHz, *o*-$Cl_2C_6D_4$, 298 K): δ –19.7 (d, ^1J(B,H) = 145 Hz), –9.1 (s), –0.8 (s) ppm. **^{13}C{^{1}H} NMR** (175 MHz, *o*-$Cl_2C_6D_4$, 298 K): δ 16.0 (*m*-CH_3), 17.6 (*p*-CH_3), 24.8 (*o*-CH_3), 41.4 ([$CHB_{11}H_5Br_6]^-$), 134.2 (*i*-C_{Ar}), 135.9 (*m*-C_{Ar}), 138.4 (*o*-C_{Ar}), 146.0 (*p*-C_{Ar}) ppm. **^{1}H/^{29}Si HMQC NMR** (700/139 MHz, *o*-$Cl_2C_6D_4$, 298 K, optimized for J = 3 Hz): δ 1.96/216.8, 2.37/216.8 ppm.

5.3 Reaction of $MePh_2SiH$ with $Ph_3C^+[B(C_6F_5)_4]^-$ (Table 1, Entry 3)

According to **GP 1**, $Ph_3C^+[B(C_6F_5)_4]^-$ (23 mg, 0.025 mmol, 1.0 equiv) was treated with $MePh_2SiH$ (20 mg, 0.10 mmol, 4.0 equiv). ^{19}F NMR spectroscopic analysis revealed formation of $B(C_6F_5)_3$ (cf. Figure S25), whereas multiple high-field signals in the ^{1}H/^{29}Si HMQC NMR spectrum indicated the presence of various organosilicon compounds. GC-MS analysis eventually revealed the existence of several silanes arising from substituent exchange reactions and anion decomposition, such as $MePh(C_6F_5)SiH$ (minor), $Me_2Ph(C_6F_5)Si$ (major), $Ph_2(C_6F_5)SiH$ (minor), and $MePh_2(C_6F_5)Si$ (major).

5.4 Reaction of $MePh_2SiH$ with $Ph_3C^+[CHB_{11}H_5Br_6]^-$: Generation of $MePh_2Si^+[CHB_{11}H_5Br_6]^-$ with Small Amounts of $Me_2PhSi^+[CHB_{11}H_5Br_6]^-$ (Table 1, Entry 4)

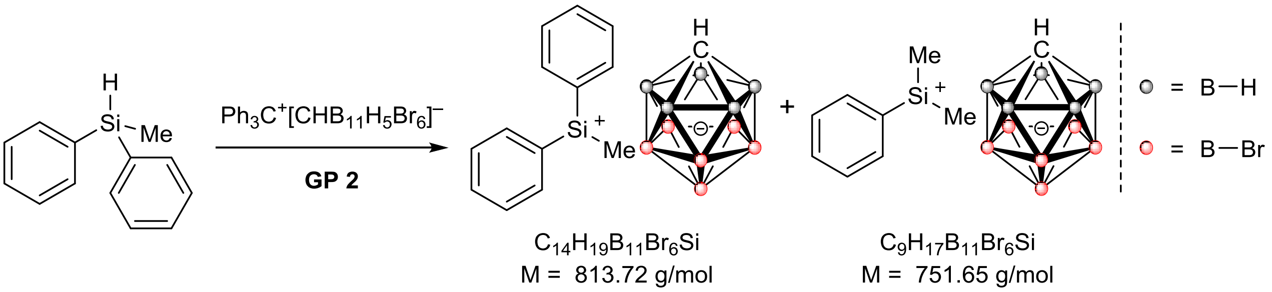

According to **GP 2**, $Ph_3C^+[CHB_{11}H_5Br_6]^-$ (20 mg, 0.023 mmol, 1.0 equiv) was treated with $MePh_2SiH$ (19 mg, 0.096 mmol, 4.2 equiv). The reaction was stopped after stirring for 24 h at room temperature. The NMR spectra showed the formation of $MePh_2Si^+[CHB_{11}H_5Br_6]^-$ along with small amounts of $Me_2PhSi^+[CHB_{11}H_5Br_6]^-$ (ratio ~79:21). No significant change in the NMR spectra was observed independent of reaction time, temperature, or stoichiometry of the hydrosilane (e.g., 7 days at room temperature, 3 days at 50 °C, or 2.0 equiv of $MePh_2SiH$).

Single crystals of $MePh_2Si^+[CHB_{11}H_5Br_6]^-$ suitable for X-ray diffraction were obtained from a solution in *o*-$Cl_2C_6D_4$ by vapor diffusion with *n*-hexane at room temperature (cf. Figure S60). CCDC 1818582 contains the supplementary crystallographic data. These data are provided free of charge by The Cambridge Crystallographic Data Centre.

^{1}H NMR (500 MHz, *o*-$Cl_2C_6D_4$, 300 K): δ 1.51 (s, 3H, $SiCH_3$), ~1.7–3.2 (br m, 6H, $[CHB_{11}H_5Br_6]^-$), 7.35 (dd, ^3J(H,H) = 7.7 Hz, ^3J(H,H) = 7.4 Hz, 4H, *m*-H_{Ar}), 7.47 (t, ^3J(H,H) = 7.5 Hz, 2H, *p*-H_{Ar}), 7.64 (d, ^3J(H,H) = 7.6 Hz, 4H, *o*-H_{Ar}) ppm. **^{11}B NMR** (161 MHz, *o*-$Cl_2C_6D_4$, 300 K): δ –20.8 (br s), –9.2 (s), –1.4 (s) ppm. **^{13}C{^{1}H} NMR** (126 MHz, *o*-$Cl_2C_6D_4$, 300 K): δ –0.6 ($SiCH_3$), 41.3 ($[CHB_{11}H_5Br_6]^-$), 126.0 (*i*-C_{Ar}), 129.5 (*m*-C_{Ar}), 134.5 (*p*-C_{Ar}), 135.6 (*o*-C_{Ar}) ppm. **^{1}H/^{29}Si HMQC NMR** (500/99 MHz, *o*-$Cl_2C_6D_4$, 300 K, optimized for *J* = 7 Hz): δ 1.51/57.4, 7.64/57.4 ppm.

Selected NMR spectroscopic data for **$Me_2PhSi^+[CHB_{11}H_5Br_6]^-$**:
^{1}H NMR (500 MHz, *o*-$Cl_2C_6D_4$, 300 K): δ 1.20 (s, 3H, $SiCH_3$) ppm. **^{1}H/^{29}Si HMQC NMR** (500/99 MHz, *o*-$Cl_2C_6D_4$, 300 K, optimized for *J* = 7 Hz): δ 1.20/76.0 ppm.

Trapping experiments with **$(C_6F_5)_3PF_2$**:
The reaction mixture was treated with $(C_6F_5)_3PF_2$[S9] (~13 mg, ~0.023 mmol, ~1.0 equiv) in order to convert the silicon cations into the corresponding fluorosilanes, thereby facilitating

product characterization by both NMR spectroscopic and GC-MS analysis. Upon addition of $(C_6F_5)_3PF_2$, immediate decolorization of the reaction mixture was observed. $MePh_2SiF$ and Me_2PhSiF were obtained as major compounds.

Selected NMR spectroscopic data for **$MePh_2SiF$**:
^{1}H NMR (500 MHz, *o*-$Cl_2C_6D_4$, 300 K): δ 0.65 (d, ^3J(H,F) = 7.2 Hz, 3H, SiCH_3) ppm. **^{1}H/^{29}Si HMQC NMR** (500/99 MHz, *o*-$Cl_2C_6D_4$, 300 K, optimized for J = 7 Hz): δ 0.65/8.2 ppm.

Selected NMR spectroscopic data for **Me_2PhSiF**:
^{1}H NMR (500 MHz, *o*-$Cl_2C_6D_4$, 300 K): δ 0.40 (d, ^3J(H,F) = 7.2 Hz, 6H, SiCH_3) ppm. **^{1}H/^{29}Si HMQC NMR** (500/99 MHz, *o*-$Cl_2C_6D_4$, 300 K, optimized for J = 7 Hz): δ 0.40/20.5 ppm.

5.5 Reaction of $MePh_2SiH$ with $Ph_3C^+[B(C_6F_5)_4]^-$ (Table 1, Entry 5)

According to **GP 1**, $Ph_3C^+[B(C_6F_5)_4]^-$ (23 mg, 0.025 mmol, 1.0 equiv) was treated with $MePh_2SiH$ (20 mg, 0.10 mmol, 4.0 equiv). ^{19}F NMR spectroscopic analysis revealed formation of $B(C_6F_5)_3$ (cf. Figure S25), whereas multiple high-field signals in the ^{1}H/^{29}Si HMQC NMR spectrum indicated the presence of various organosilicon compounds. GC-MS analysis eventually revealed the existence of several silanes arising from substituent exchange reactions and anion decomposition, such as $MePh(C_6F_5)SiH$ (minor), $Me_2Ph(C_6F_5)Si$ (major), $Ph_2(C_6F_5)SiH$ (minor), and $MePh_2(C_6F_5)Si$ (major).

5.6 Reaction of Me_2PhSiH with $Ph_3C^+[CHB_{11}H_5Br_6]^-$: Generation of $Me_3Si^+[CHB_{11}H_5Br_6]^-$ (Table 1, Entry 6)

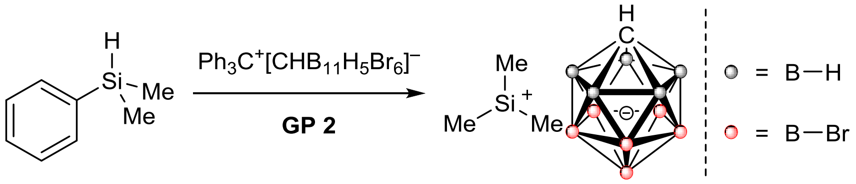

$C_4H_{15}B_{11}Br_6Si$
M = 689.58 g/mol

According to **GP 2**, $Ph_3C^+[CHB_{11}H_5Br_6]^-$ (50 mg, 0.058 mmol, 1.0 equiv) was treated with Me_2PhSiH (16 or 32 mg, 0.12 or 0.23 mmol, 2.0 or 4.0 equiv). The reaction was stopped after stirring for 18 h at room temperature. Clean formation of $Me_3Si^+[CHB_{11}H_5Br_6]^-$ was monitored by NMR spectroscopic analysis.

Single crystals of $Me_3Si^+[CHB_{11}H_5Br_6]^-$ suitable for X-ray diffraction were obtained from a solution in *o*-$F_2C_6D_4$ by vapor diffusion with *n*-hexane at room temperature (cf. Figure S61). CCDC 1818576 contains the supplementary crystallographic data. These data are provided free of charge by The Cambridge Crystallographic Data Centre.

^{1}H NMR (500 MHz, *o*-$Cl_2C_6D_4$, 300 K): δ 0.83 (s, 9H, $SiCH_3$), ~1.9–3.4 (br m, 6H, $[CHB_{11}H_5Br_6]^-$) ppm. **^{11}B NMR** (161 MHz, *o*-$Cl_2C_6D_4$, 300 K): δ –20.0 (d, ^1J(B,H) = 143 Hz), –9.3 (s), –1.4 (s) ppm. **^{13}C{^{1}H} DEPT NMR** (126 MHz, *o*-$Cl_2C_6D_4$, 300 K): δ 3.3 ($SiCH_3$), 41.0 ($[CHB_{11}H_5Br_6]^-$) ppm. **^{1}H/^{29}Si HMQC NMR** (500/99 MHz, *o*-$Cl_2C_6D_4$, 300 K, optimized for J = 7 Hz): δ 0.83/93.4 ppm.

5.7 Reaction of $Me_2(C_6Me_5)SiH$ with $Ph_3C^+[B(C_6F_5)_4]^-$: Generation of $(C_6Me_5)Si^+[B(C_6F_5)_4]^-$ (Table 1, Entry 7)

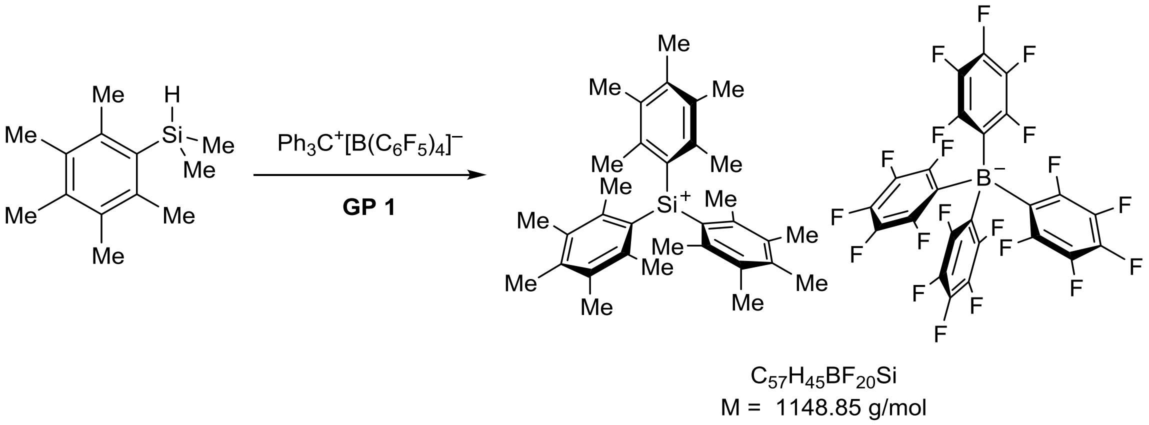

According to **GP 1**, $Ph_3C^+[B(C_6F_5)_4]^-$ (23 mg, 0.025 mmol, 1.0 equiv) was treated with $Me_2(C_6Me_5)SiH$ (21 mg, 0.10 mmol, 4.0 equiv). NMR spectroscopic analysis revealed formation of $(C_6Me_5)_3Si^+[B(C_6F_5)_4]^-$ along with minor impurities.

The NMR spectroscopic data are consistent with those reported in **5.1**.

5.8 Reaction of $Me_2(C_6Me_5)SiH$ with $Ph_3C^+[CHB_{11}H_5Br_6]^-$: Generation of $Me_3Si^+[CHB_{11}H_5Br_6]^-$ (Table 1, Entry 8)

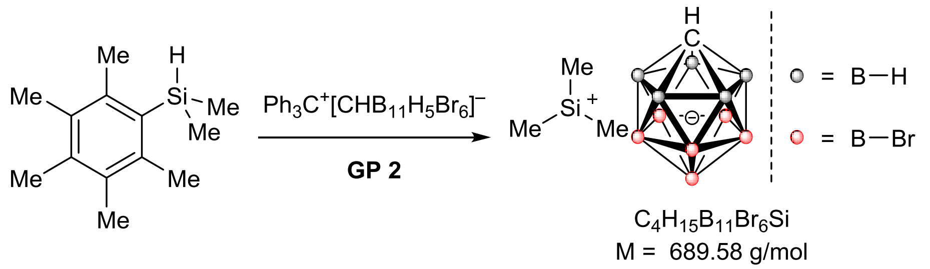

According to **GP 2**, $Ph_3C^+[CHB_{11}H_5Br_6]^-$ (20 mg, 0.023 mmol, 1.0 equiv) was treated with $Me_2(C_6Me_5)SiH$ (19 mg, 0.092 mmol, 4.0 equiv). The reaction was stopped after stirring for 72 h at 50 °C. The NMR spectra showed the formation of $Me_3Si^+[CHB_{11}H_5Br_6]^-$ along with minor amounts of $(C_6Me_5)_3Si^+[CHB_{11}H_5Br_6]^-$.

The NMR spectroscopic data are consistent with those reported in **5.6**.

5.9 Reaction of Et_2PhSiH with $Ph_3C^+[CHB_{11}H_5Br_6]^-$: Generation of $Et_3Si^+[CHB_{11}H_5Br_6]^-$ (Table 2, Entry 2)

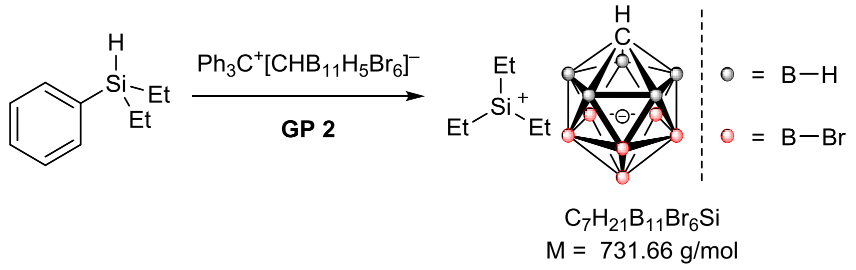

According to **GP 2**, $Ph_3C^+[CHB_{11}H_5Br_6]^-$ (50 mg, 0.058 mmol, 1.0 equiv) was treated with Et_2PhSiH (38 mg, 0.23 mmol, 4.0 equiv). The reaction was stopped after stirring for 7 days at room temperature. Clean formation of $Et_3Si^+[CHB_{11}H_5Br_6]^-$ was monitored by NMR spectroscopic analysis. Performing the reaction with less hydrosilane (2.0 equiv) for 18 h allowed for the detection of small amounts of a second silylium ion, which was identified as unscrambled $Et_2PhSi^+[CHB_{11}H_5Br_6]^-$ (ratio ~90:10).

^{1}H NMR (500 MHz, *o*-$Cl_2C_6D_4$, 298 K): δ 0.94 (t, ^3J(H,H) = 7.8 Hz, 9H, $SiCH_2CH_3$), 1.19 (q, ^3J(H,H) = 7.8 Hz, 6H, $SiCH_2CH_3$), ~1.9–3.4 (br m, 6H, $[CHB_{11}H_5Br_6]^-$) ppm. **^{11}B NMR** (161 MHz, *o*-$Cl_2C_6D_4$, 298 K): δ –19.9 (br s), –9.0 (s), –1.1 (s) ppm. **^{13}C{^{1}H} DEPT NMR** (126 MHz, *o*-$Cl_2C_6D_4$, 298 K): δ 5.9 ($SiCH_2CH_3$), 7.6 ($SiCH_2CH_3$), 41.0 ($[CHB_{11}H_5Br_6]^-$) ppm.

^{1}H/^{29}Si HMQC NMR (500/99 MHz, *o*-$Cl_2C_6D_4$, 298 K, optimized for *J* = 7 Hz): δ 0.94/101.1, 1.19/101.1 ppm.

Selected NMR spectroscopic data for **$Et_2PhSi^+[CHB_{11}H_5Br_6]^-$**:
^{1}H NMR (500 MHz, *o*-$Cl_2C_6D_4$, 298 K): δ 1.06 (t, ^3J(H,H) = 7.9 Hz, 6H, $SiCH_2CH_3$), 1.53 (q, ^3J(H,H) = 7.9 Hz, 4H, $SiCH_2CH_3$), 7.33 (dd, ^3J(H,H) = 7.6 Hz, ^3J(H,H) = 7.4 Hz, 2H, *m*-H_{Ar}), 7.44 (t, ^3J(H,H) = 7.6 Hz, 1H, *p*-H_{Ar}), 7.51 (d, ^3J(H,H) = 7.4 Hz, 2H, *o*-H_{Ar}) ppm. **^{1}H/^{29}Si HMQC NMR** (500/99 MHz, *o*-$Cl_2C_6D_4$, 298 K, optimized for *J* = 7 Hz): δ 1.06/76.0, 1.53/76.0, 7.51/76.0 ppm.

5.10 Independent Synthesis of $Et_3Si^+[CHB_{11}H_5Br_6]^-$ from the Reaction of Et_3SiH with $Ph_3C^+[CHB_{11}H_5Br_6]^-$

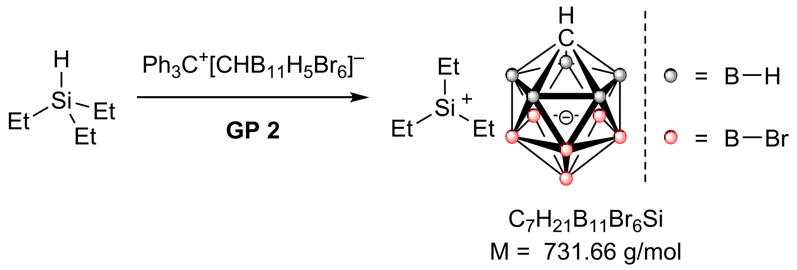

According to **GP 2**, $Ph_3C^+[CHB_{11}H_5Br_6]^-$ (20 mg, 0.023 mmol, 1.0 equiv) was treated with Et_3SiH (5.4 mg, 0.046 mmol, 2.0 equiv). The reaction was stopped after stirring for 18 h at room temperature. Clean formation of $Et_3Si^+[CHB_{11}H_5Br_6]^-$ was monitored by NMR spectroscopic analysis.

The NMR spectroscopic data are consistent with those reported in **5.9**.

5.11 Reaction of *i*Pr_2PhSiH with $Ph_3C^+[CHB_{11}H_5Br_6]^-$: Generation of *i*$Pr_2PhSi^+[CHB_{11}H_5Br_6]^-$ (Table 2, Entry 3)

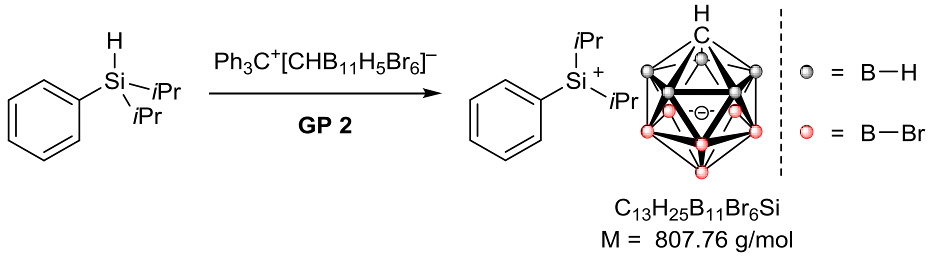

According to **GP 2**, $Ph_3C^+[CHB_{11}H_5Br_6]^-$ (20 mg, 0.023 mmol, 1.0 equiv) was treated with *i*Pr_2PhSiH (9.0 mg, 0.46 mmol, 2.0 equiv). The reaction was stopped after stirring for 24 h at room temperature. Clean formation of *i*$Pr_2PhSi^+[CHB_{11}H_5Br_6]^-$ was monitored by NMR spectroscopic analysis.

Single crystals of *i*$Pr_2PhSi^+[CHB_{11}H_5Br_6]^-$ suitable for X-ray diffraction were obtained from a solution in *o*-$Cl_2C_6D_4$ by vapor diffusion with *n*-hexane at room temperature (cf. Figure S62). CCDC 1818581 contains the supplementary crystallographic data. These data are provided free of charge by The Cambridge Crystallographic Data Centre.

^{1}H NMR (500 MHz, *o*-$Cl_2C_6D_4$, 298 K): δ 1.16 (d, ^3J(H,H) = 7.5 Hz, 12H, SiCHCH_3), 1.18 (sept, ^3J(H,H) = 7.5 Hz, 2H, SiC*H*CH_3), ~1.6–3.1 (br m, 6H, [C*H*$B_{11}H_5Br_6$]$^-$), 7.38 (dd, ^3J(H,H) = 7.6 Hz, ^3J(H,H) = 7.5 Hz, 2H, *m*-H_{Ar}), 7.46 (t, ^3J(H,H) = 7.4 Hz, 1H, *p*-H_{Ar}), 7.59 (d, ^3J(H,H) = 7.6 Hz, 2H, *o*-H_{Ar}) ppm. **^{11}B NMR** (161 MHz, *o*-$Cl_2C_6D_4$, 298 K): δ –20.8 (br s), –9.2 (s), –1.4 (s) ppm. **^{13}C{^{1}H} NMR** (126 MHz, *o*-$Cl_2C_6D_4$, 298 K): δ 15.9 (Si*C*HCH_3), 17.4 (SiCH*C*H_3), 41.0 ([*C*$HB_{11}H_5Br_6$]$^-$), 123.9 (*i*-C_{Ar}), 129.3 (*m*-C_{Ar}), 134.2 (*p*-C_{Ar}), 135.8 (*o*-C_{Ar}) ppm. **^{1}H/^{29}Si HMQC NMR** (500/99 MHz, *o*-$Cl_2C_6D_4$, 298 K, optimized for *J* = 7 Hz): δ 1.16/76.0, 1.18/ 76.0, 7.59/76.0 ppm.

5.12 Reaction of *t*Bu_2PhSiH with $Ph_3C^+[CHB_{11}H_5Br_6]^-$ (Table 2, Entry 4)

According to **GP 2**, $Ph_3C^+[CHB_{11}H_5Br_6]^-$ (20 mg, 0.023 mmol, 1.0 equiv) was treated with *t*Bu_2PhSiH (10 mg, 0.45 mmol, 2.0 equiv). The reaction was stopped after stirring for 48 h at room temperature. NMR spectroscopic analysis of the precipitate showed only trityl salt, and no silicon species was detected in the ^{1}H/^{29}Si HMQC NMR spectrum.

5.13 Reaction of Me_2BnSiH with $Ph_3C^+[CHB_{11}H_5B]^-$: Generation of $Me_3Si^+[CHB_{11}H_5Br_6]^-$ (Table 2, Entry 2)

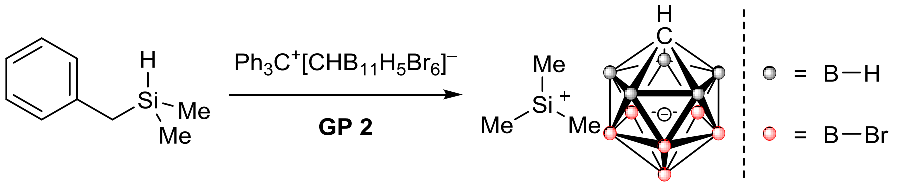

$C_4H_{15}B_{11}Br_6Si$
M = 689.58 g/mol

According to **GP 2**, $Ph_3C^+[CHB_{11}H_5Br_6]^-$ (20 mg, 0.023 mmol, 1.0 equiv) was treated with Me_2BnSiH (7.0 mg, 0.047 mmol, 2.0 equiv). The reaction was stopped after stirring for 18 h at room temperature. Clean formation of $Me_3Si^+[CHB_{11}H_5Br_6]^-$ was monitored by NMR spectroscopic analysis.

The NMR spectroscopic data are consistent with those reported in **5.6**.

5.14 Reaction of $Me_2\textit{t}BuSiH$ with $Ph_3C^+[CHB_{11}H_5Br_6]^-$: Generation of $Me_2\textit{t}BuSi^+[CHB_{11}H_5Br_6]^-$ (Table 2, Entry 3)

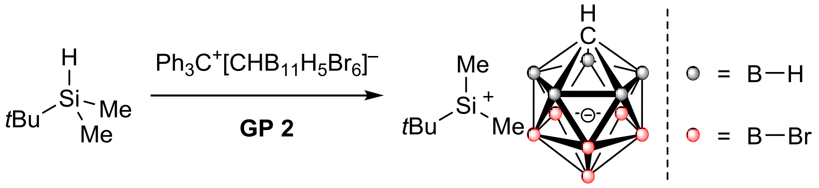

$C_7H_{21}B_{11}Br_6Si$
M = 731.66 g/mol

According to **GP 2**, $Ph_3C^+[CHB_{11}H_5Br_6]^-$ (20 mg, 0.023 mmol, 1.0 equiv) was treated with Me_2*t*BuSiH (5.4 mg, 0.046 mmol, 2.0 equiv). The reaction was stopped after stirring for 24 h at room temperature. Clean formation of Me_2*t*BuSi$^+[CHB_{11}H_5Br_6]^-$ was monitored by NMR spectroscopic analysis.

^{1}H NMR (500 MHz, *o*-$Cl_2C_6D_4$, 300 K): δ 0.83 (s, 9H, SiCCH_3), 0.88 (s, 3H, SiCH_3), ~1.9–3.5 (br m, 6H, $[CHB_{11}H_5Br_6]^-$) ppm. **^{11}B NMR** (161 MHz, *o*-$Cl_2C_6D_4$, 300 K): δ –20.5 (br s), –9.2 (s), –1.2 (s) ppm. **^{13}C{^{1}H} NMR** (126 MHz, *o*-$Cl_2C_6D_4$, 300 K): δ –0.9 (SiCH_3), 22.4 (SiCCH$_3$), 24.5 (SiCCH_3), 41.5 ($[CHB_{11}H_5Br_6]^-$) ppm. **^{1}H/^{29}Si HMQC NMR** (500/99 MHz, *o*-$Cl_2C_6D_4$, 300 K, optimized for J = 7 Hz): δ 0.83/98.2, 0.88/98.2 ppm.

6 Mechanistic Control Experiments

6.1 Influence of the Carborane Counteranion on the Selectivity of the Trimethylsilylium Ion Formation (Scheme 2)

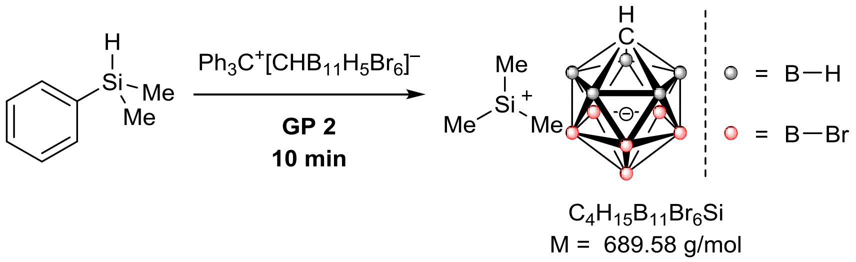

According to **GP 2**, $Ph_3C^+[CHB_{11}H_5Br_6]^-$ (50 mg, 0.058 mmol, 1.0 equiv) was treated with Me_2PhSiH (16 mg, 0.12 mmol, 2.0 equiv). The reaction was stopped after stirring for 10 min at room temperature. Full conversion of the trityl salt was observed, as verified by ^1H NMR spectroscopic analysis and indicated by the formation of a white suspension. NMR spectroscopic analysis revealed formation of $Me_3Si^+[CHB_{11}H_5Br_6]^-$ along with small amounts of unscrambled $Me_2PhSi^+[CHB_{11}H_5Br_6]^-$ (cf. **5.4**; ratio ~84:16).

6.2 Influence of the Borate Counteranion on the Selectivity of the Trimethylsilylium Ion Formation (Scheme 2)

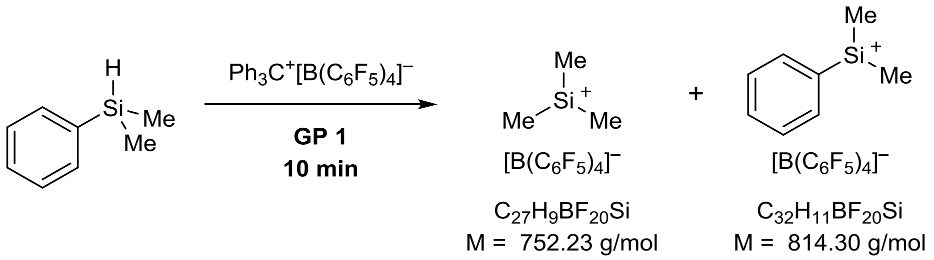

According to **GP 1**, $Ph_3C^+[B(C_6F_5)_4]^-$ (23 mg, 0.025 mmol, 1.0 equiv) was treated with Me_2PhSiH (6.8 mg, 0.050 mmol, 2.0 equiv). The reaction was stopped after stirring for 10 min at room temperature. Full conversion of the trityl salt was observed, as verified by ^1H NMR spectroscopic analysis. NMR spectroscopic analysis revealed formation of a mixture of $Me_3Si^+[B(C_6F_5)_4]^-$ with unscrambled $Me_2PhSi^+[B(C_6F_5)_4]^-$ (ratio ~51:49). Due to the low stabilitiy of these silylium ions, no $^{13}C\{^1H\}$ NMR spectrum was measured.

Selected NMR spectroscopic data for **$Me_3Si^+[B(C_6F_5)_4]^-$**:
^{1}H NMR (500 MHz, *o*-$Cl_2C_6D_4$, 300 K): δ 0.25 (s, 9H, SiCH_3) ppm. **^{1}H/^{29}Si HMQC NMR** (500/99 MHz, *o*-$Cl_2C_6D_4$, 300 K, optimized for J = 7 Hz): δ 0.25/90.0 ppm.

Selected NMR spectroscopic data for **$Me_2PhSi^+[B(C_6F_5)_4]^-$**:
^{1}H NMR (500 MHz, *o*-$Cl_2C_6D_4$, 300 K): δ 0.58 (s, 3H, SiCH_3), 7.03 (d, ^3J(H,H) = 6.9 Hz, 2H, *o*-H_{Ar}), 7.19–7.22 (m, 2H, *m*-H_{Ar}), 7.38 (t, ^3J(H,H) = 7.3 Hz, 1H, *p*-H_{Ar}) ppm. **^{1}H/^{29}Si HMQC NMR** (500/99 MHz, *o*-$Cl_2C_6D_4$, 300 K, optimized for J = 7 Hz): δ 0.58/69.9, 7.03/69.9 ppm.

6.3 Addition of Me_2PhSiH to a Mixture of $MePh_2Si^+[CHB_{11}H_5Br_6]^-$ and $Me_2PhSi^+[CHB_{11}H_5Br_6]^-$: Generation of $Me_3Si^+[CHB_{11}H_5Br_6]^-$ (Scheme 3)

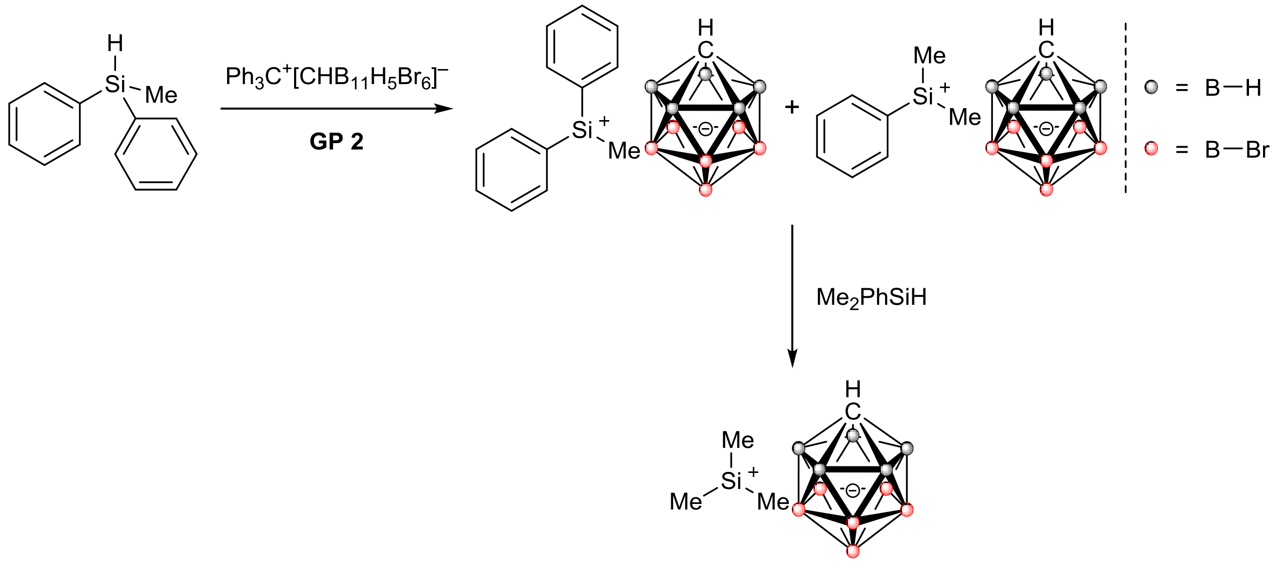

According to **GP 2**, $Ph_3C^+[CHB_{11}H_5Br_6]^-$ (20 mg, 0.023 mmol, 1.0 equiv) was treated with $MePh_2SiH$ (11 mg, 0.048 mmol, 2.1 equiv). After stirring for 22 h at room temperature, a pale yellow suspension was obtained. A solution of Me_2PhSiH (14 mg, 0.10 mmol, 4.4 equiv) in toluene (5 drops) was added, resulting in formation of a white suspension after stirring for 10 min. To ensure full conversion, the mixture was stirred for additional 22 h. *n*-Pentane (~1 mL) was added to allow full precipitation of all ionic components. The solid was collected by filtration, washed with *n*-pentane (~3 × 0.5 mL), briefly dried under vacuum, and dissolved in *o*-$Cl_2C_6D_4$ (0.60 mL). NMR spectroscopic analysis revealed exclusive formation of $Me_3Si^+[CHB_{11}H_5Br_6]^-$.

The NMR spectroscopic data are consistent with those reported in **5.6**.

7 NMR Spectra

Figure S1. ^1H NMR spectrum (500 MHz, C_6D_6, 300 K) of $Me_2(C_6Me_5)SiH$.

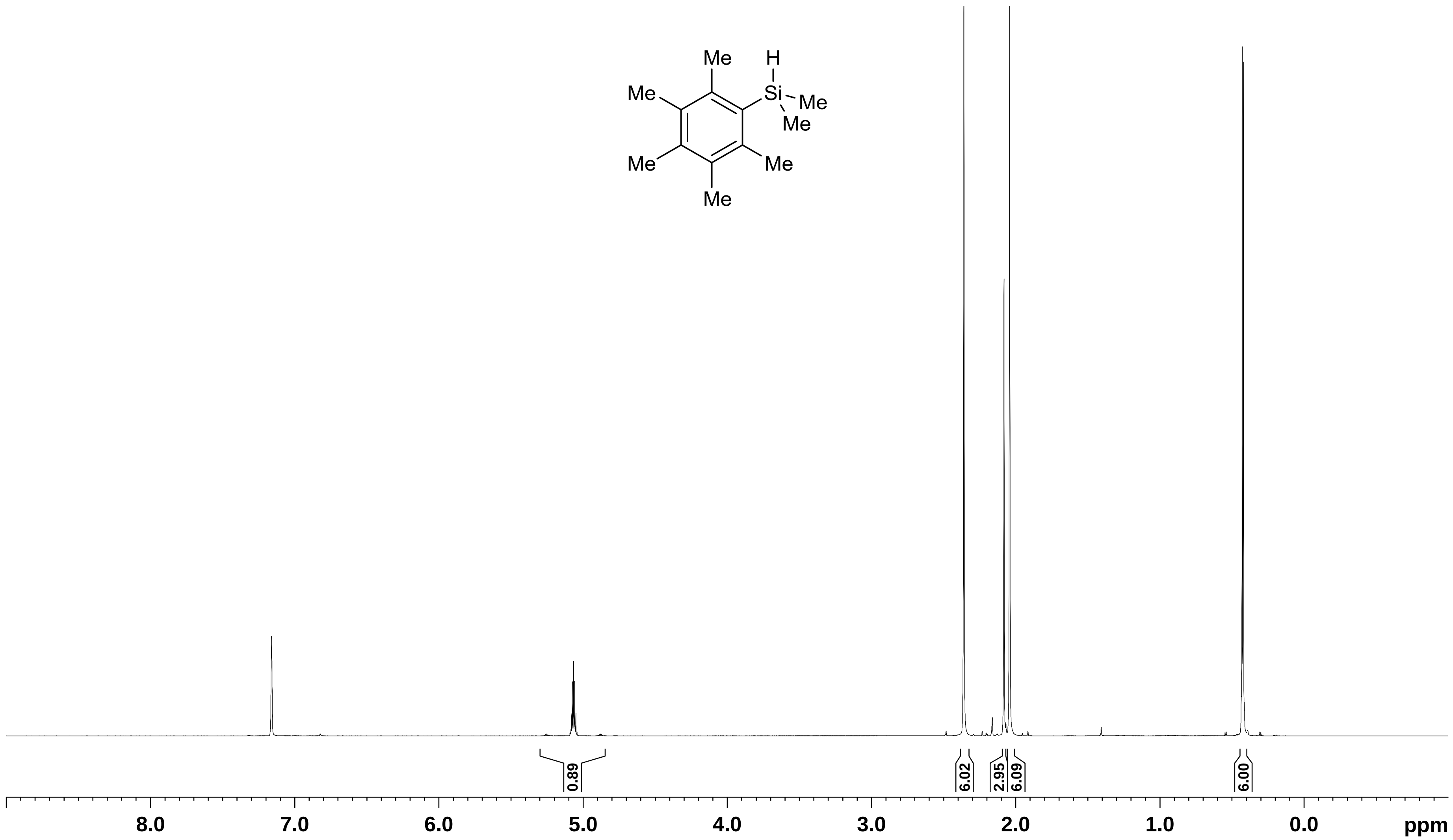

Figure S2. $^{13}C\{^{1}H\}$ NMR spectrum (126 MHz, C_6D_6, 300 K) of $Me_2(C_6Me_5)SiH$.

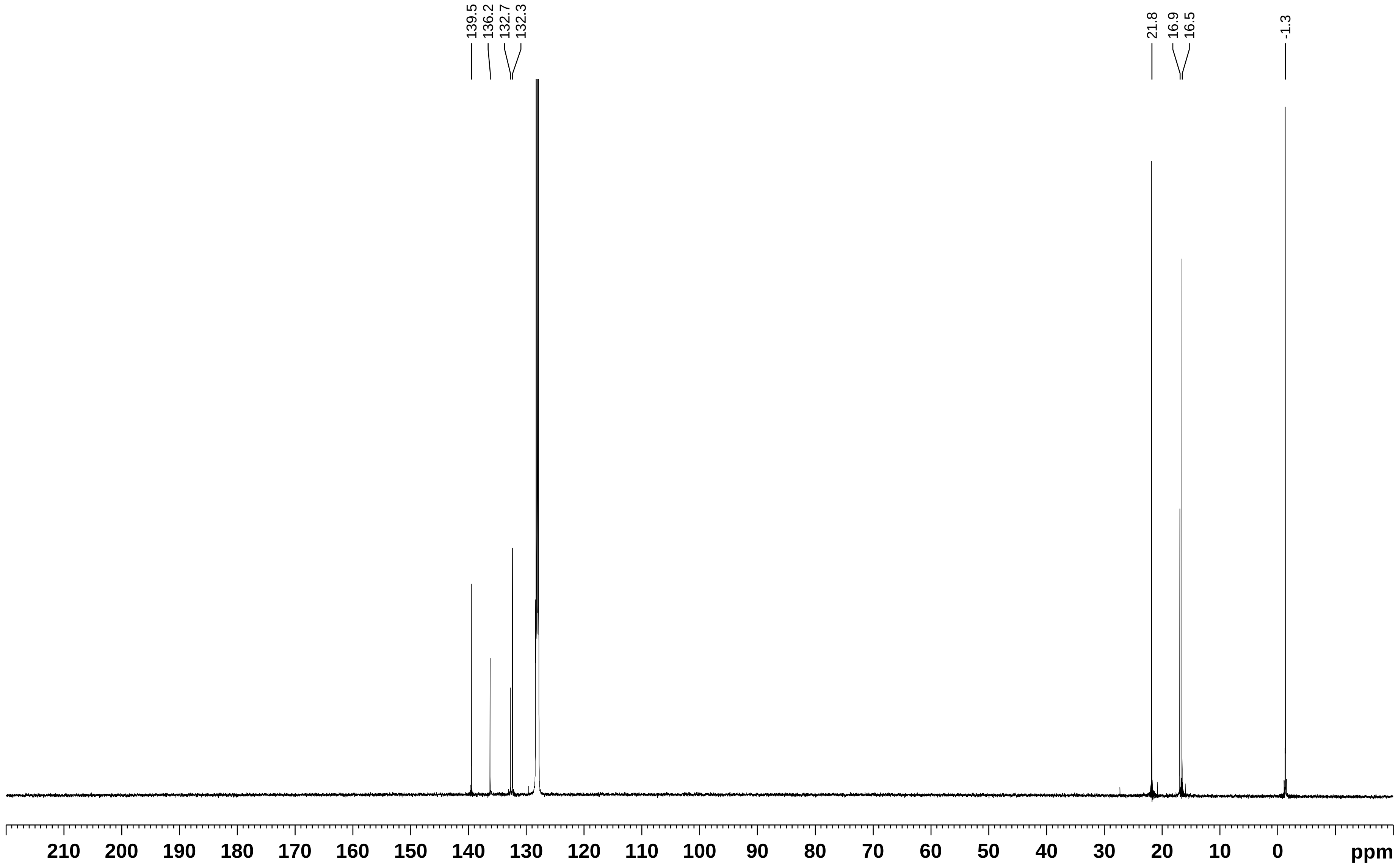

Figure S3. ^{29}Si DEPT NMR spectrum (99 MHz, C_6D_6, 300 K, optimized for *J* = 200 Hz) of $Me_2(C_6Me_5)SiH$.

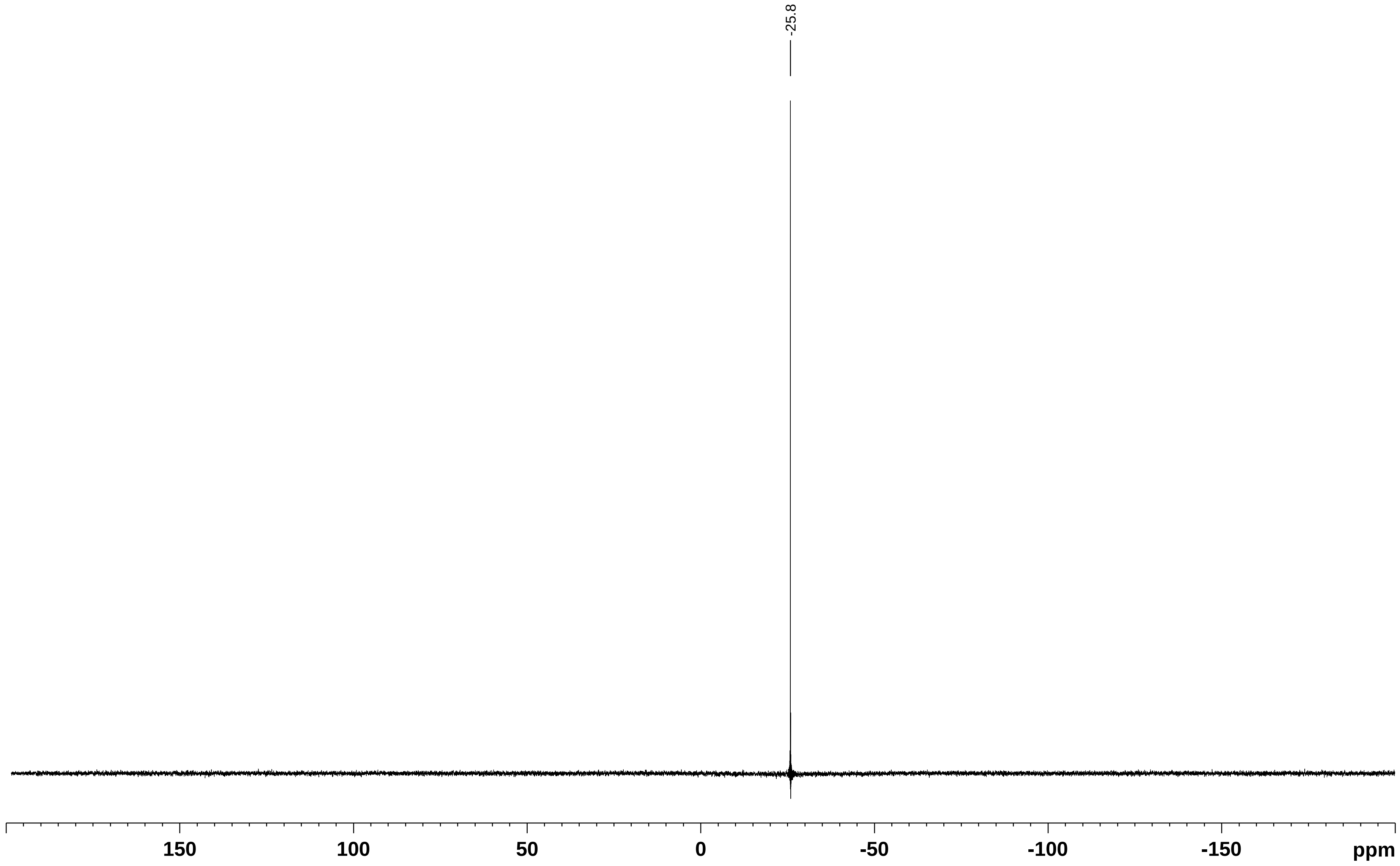

Figure S4. ^{1}H NMR spectrum (500 MHz, C_6D_6, 300 K) of Me_2BnSiH.

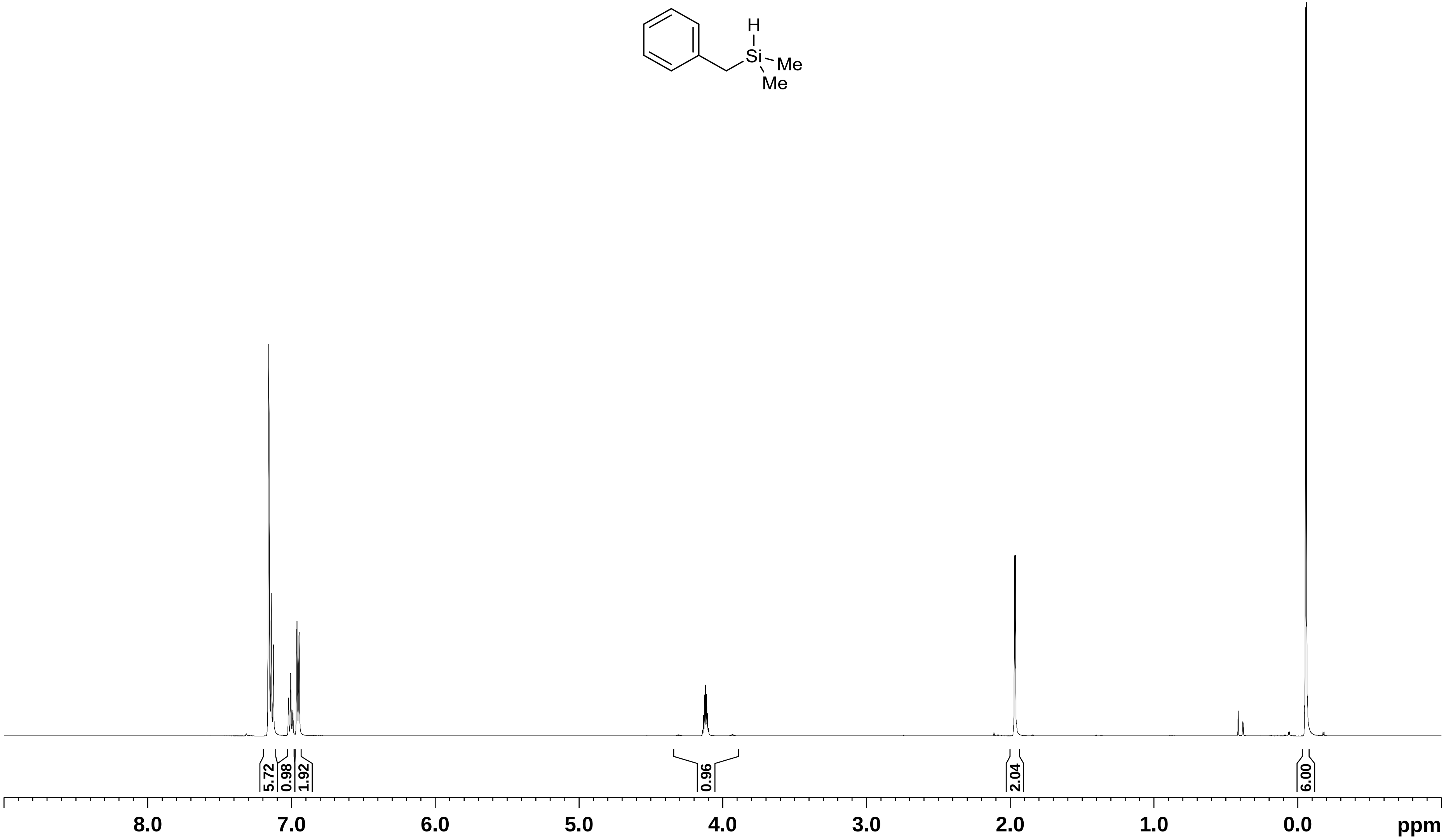

Figure S5. $^{13}C\{^{1}H\}$ NMR spectrum (126 MHz, C_6D_6, 300 K) of Me_2BnSiH.

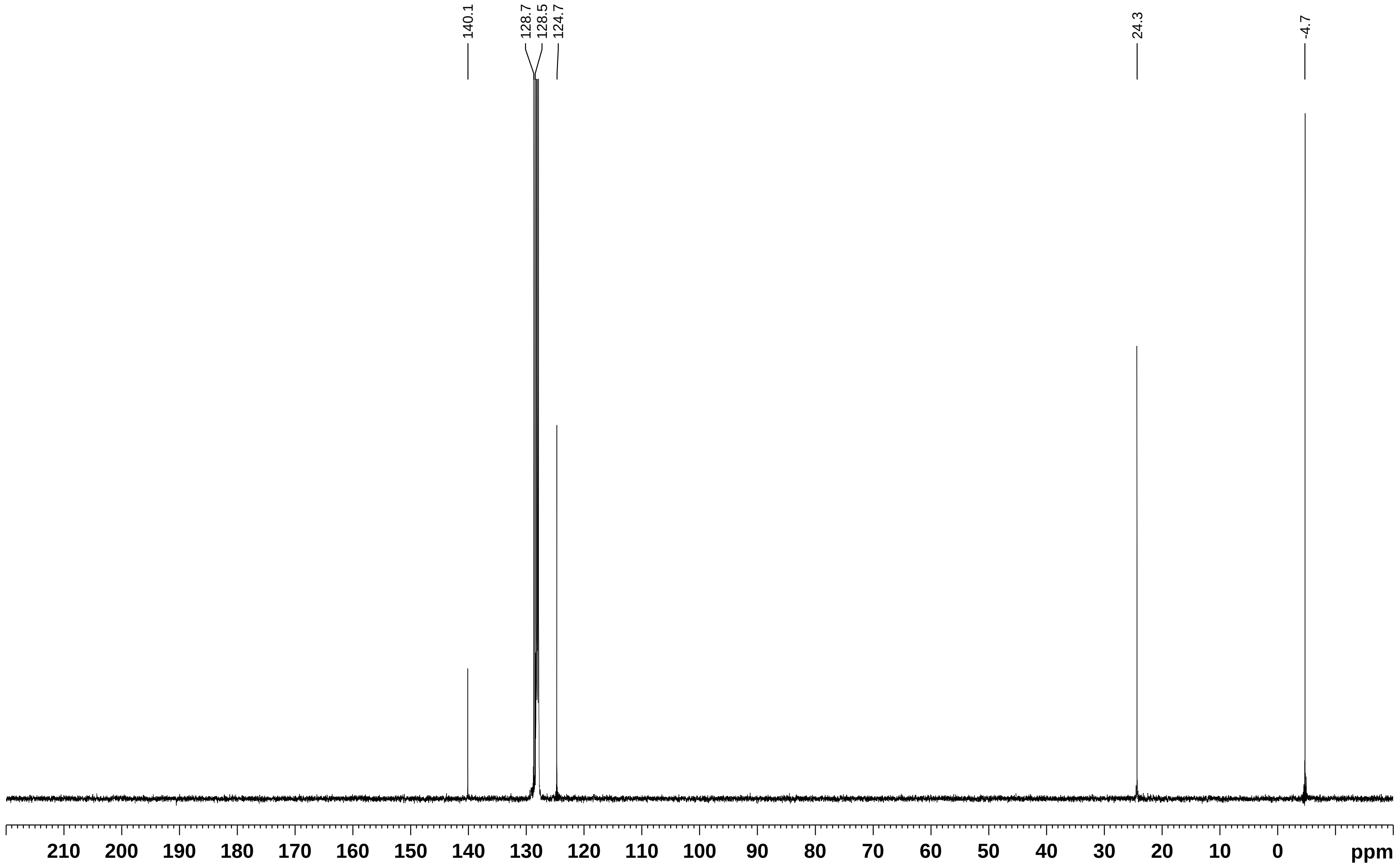

Figure S6. ^{29}Si DEPT NMR spectrum (99 MHz, C_6D_6, 300 K, optimized for J = 200 Hz) of Me_2BnSiH.

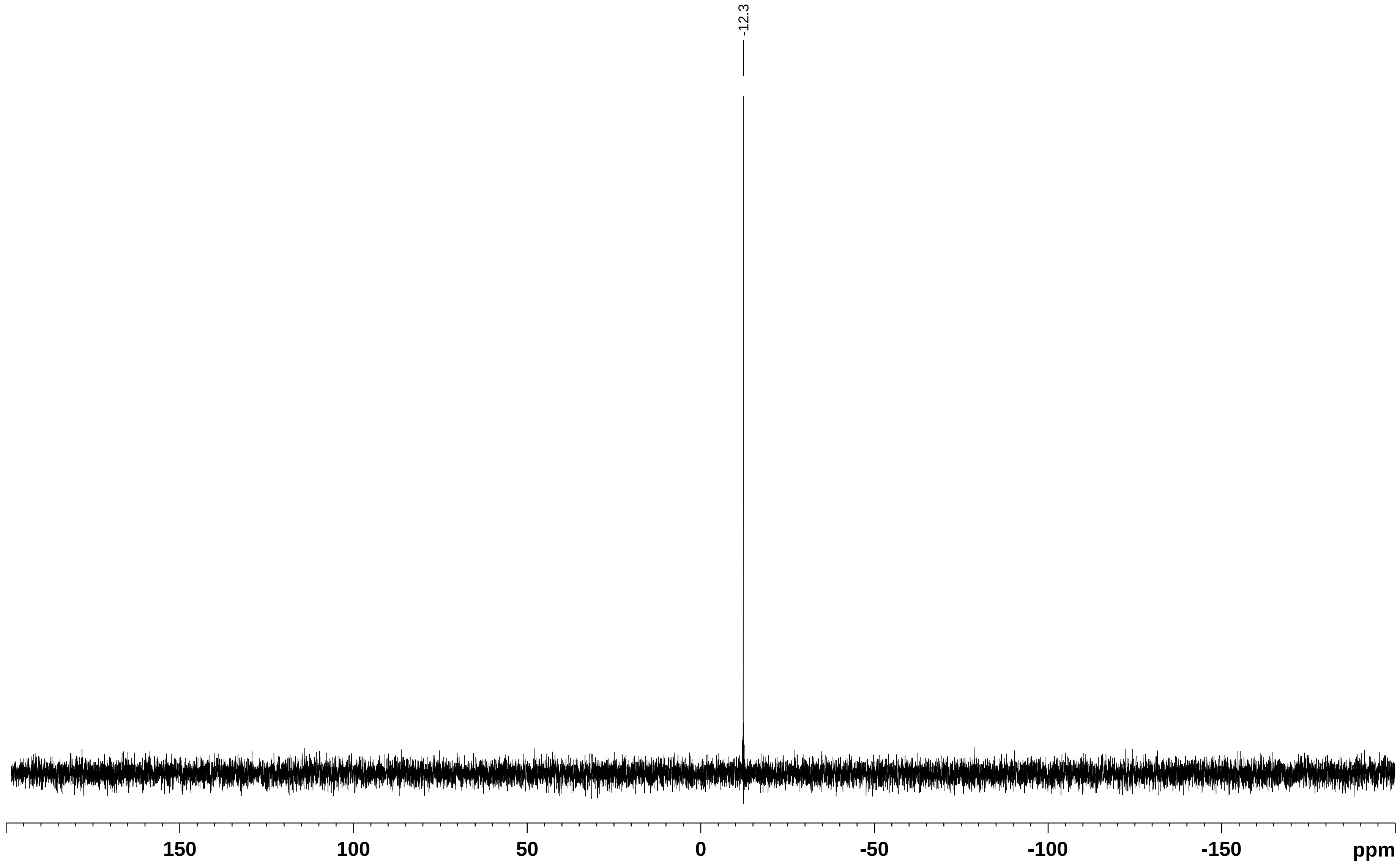

Figure S7. ^{1}H NMR spectrum (500 MHz, C_6D_6, 298 K) of *i*Pr_2PhSiH.

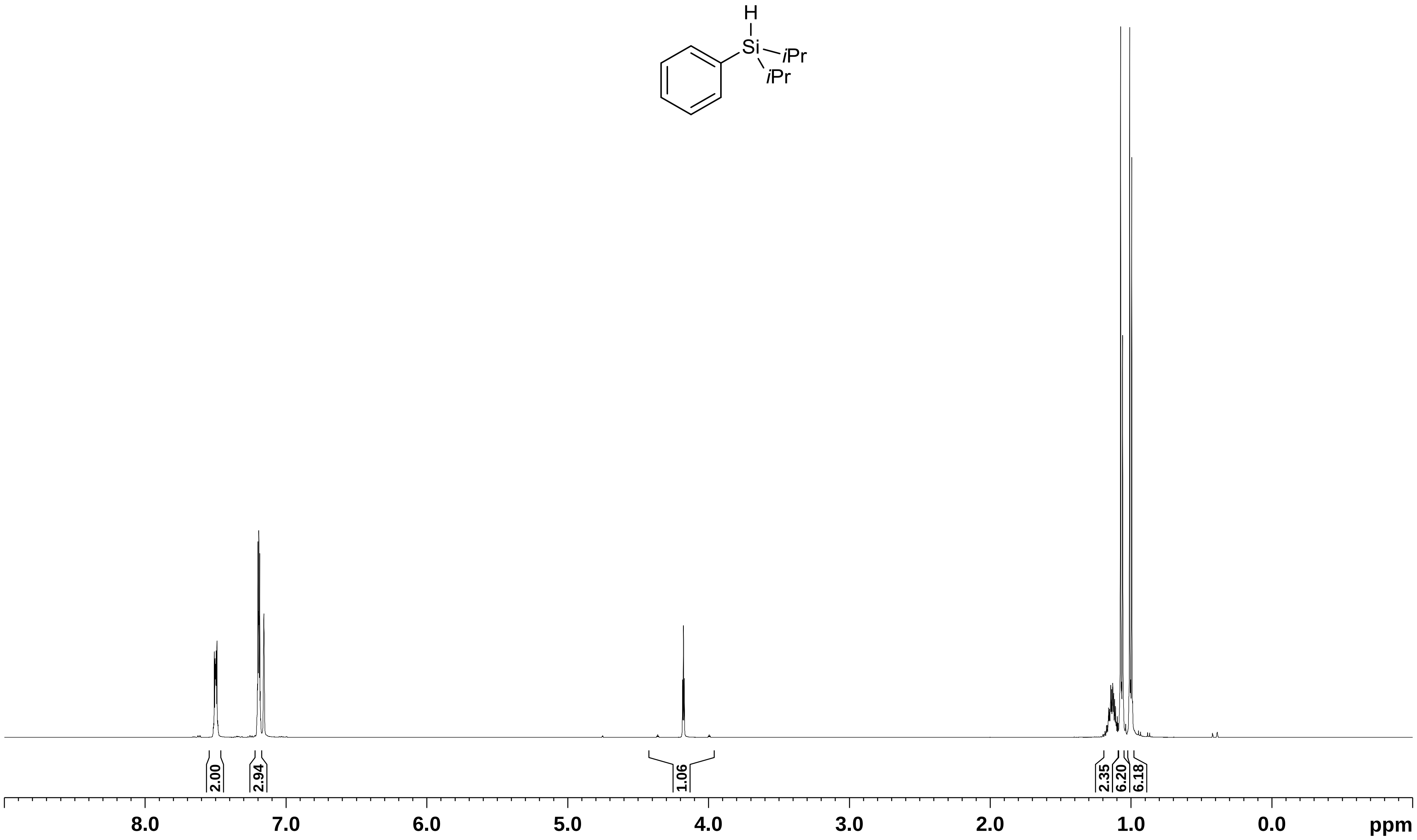

Figure S8. $^{13}C\{^1H\}$ NMR spectrum (126 MHz, C_6D_6, 298 K) of *i*Pr_2PhSiH.

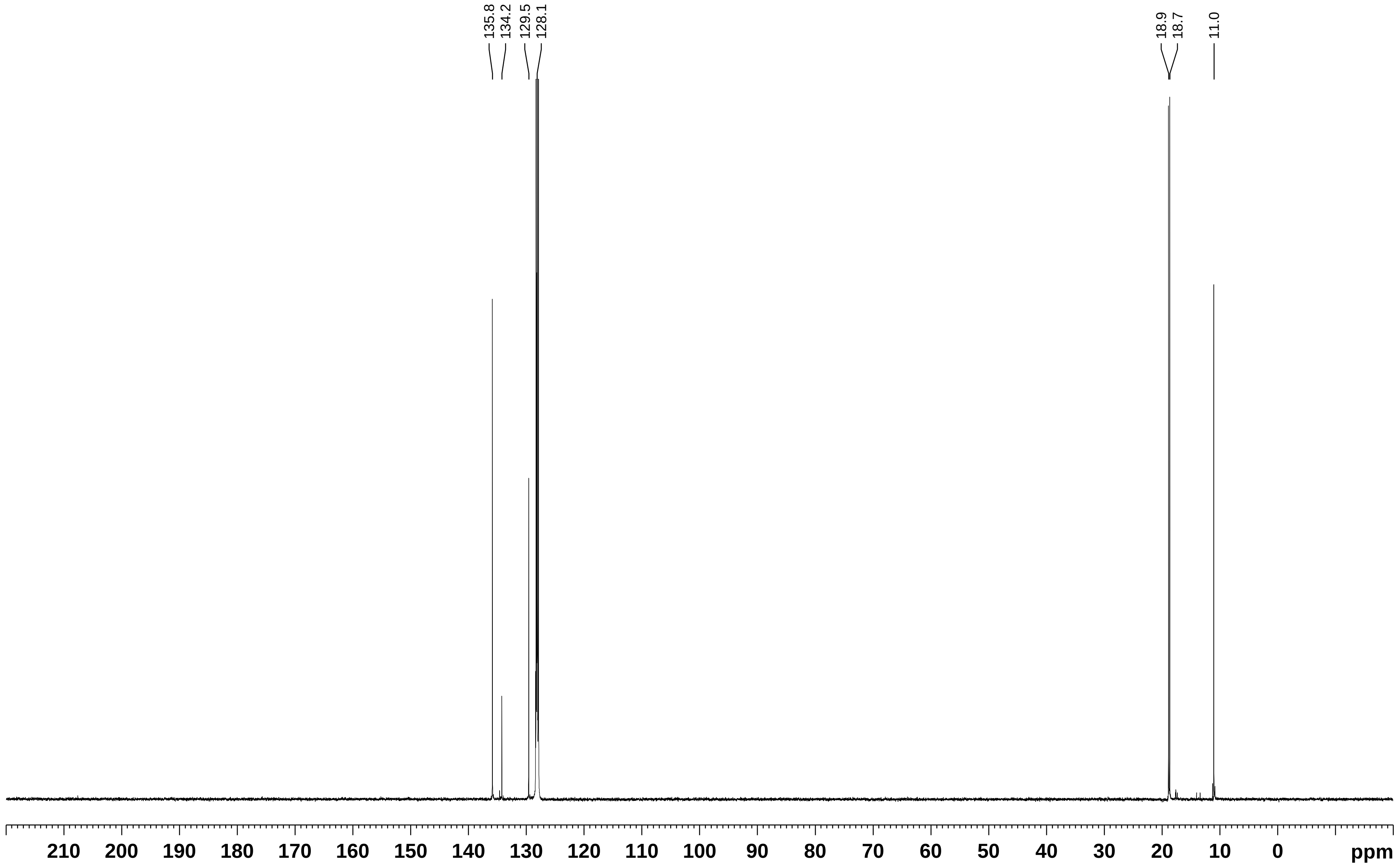

Figure S9. ^{29}Si DEPT NMR spectrum (99 MHz, C_6D_6, 298 K, optimized for J = 200 Hz) of iPr$_2$PhSiH.

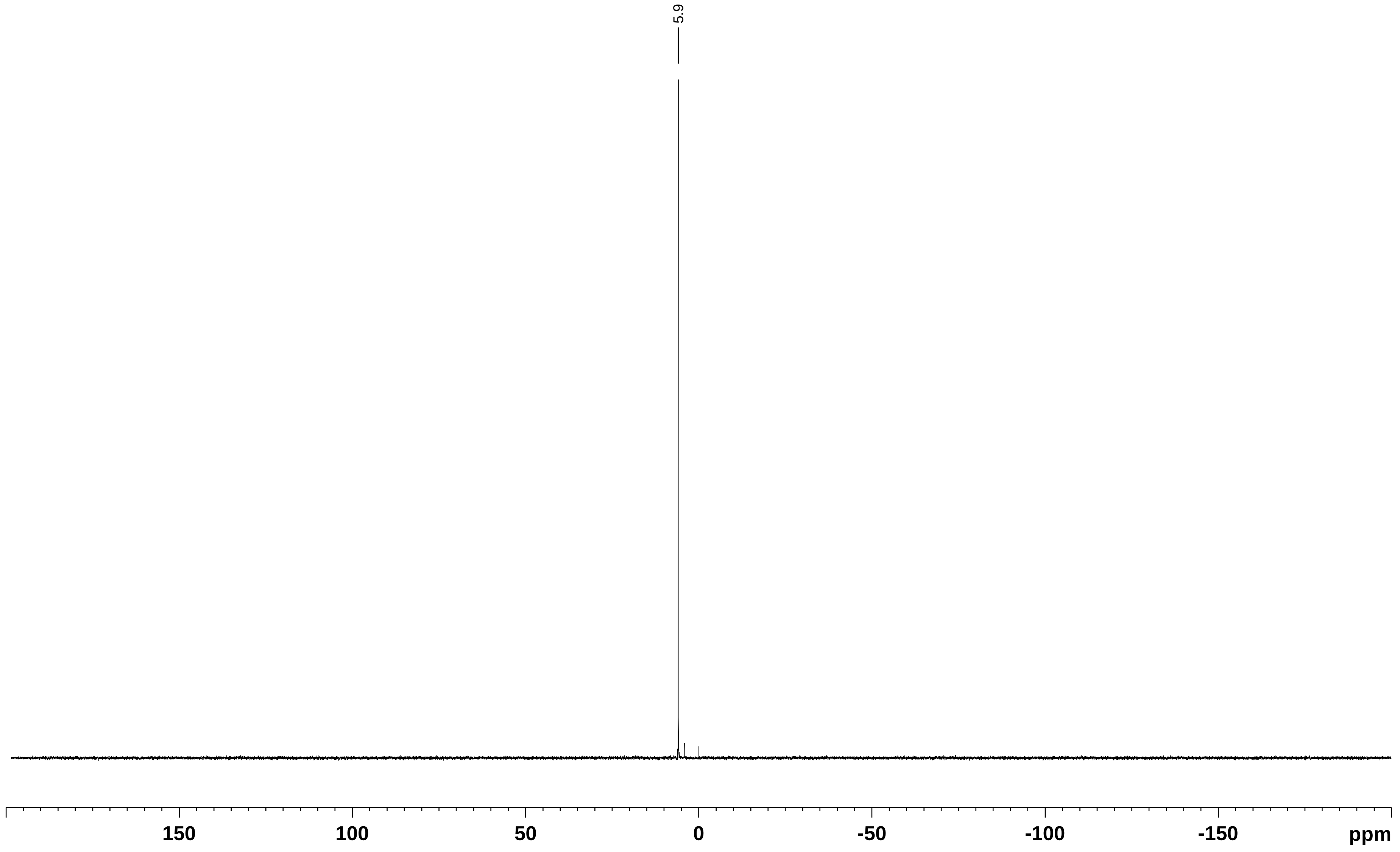

Figure S10. ^{1}H NMR spectrum (500 MHz, C_6D_6, 300 K) of *t*Bu_2PhSiH.

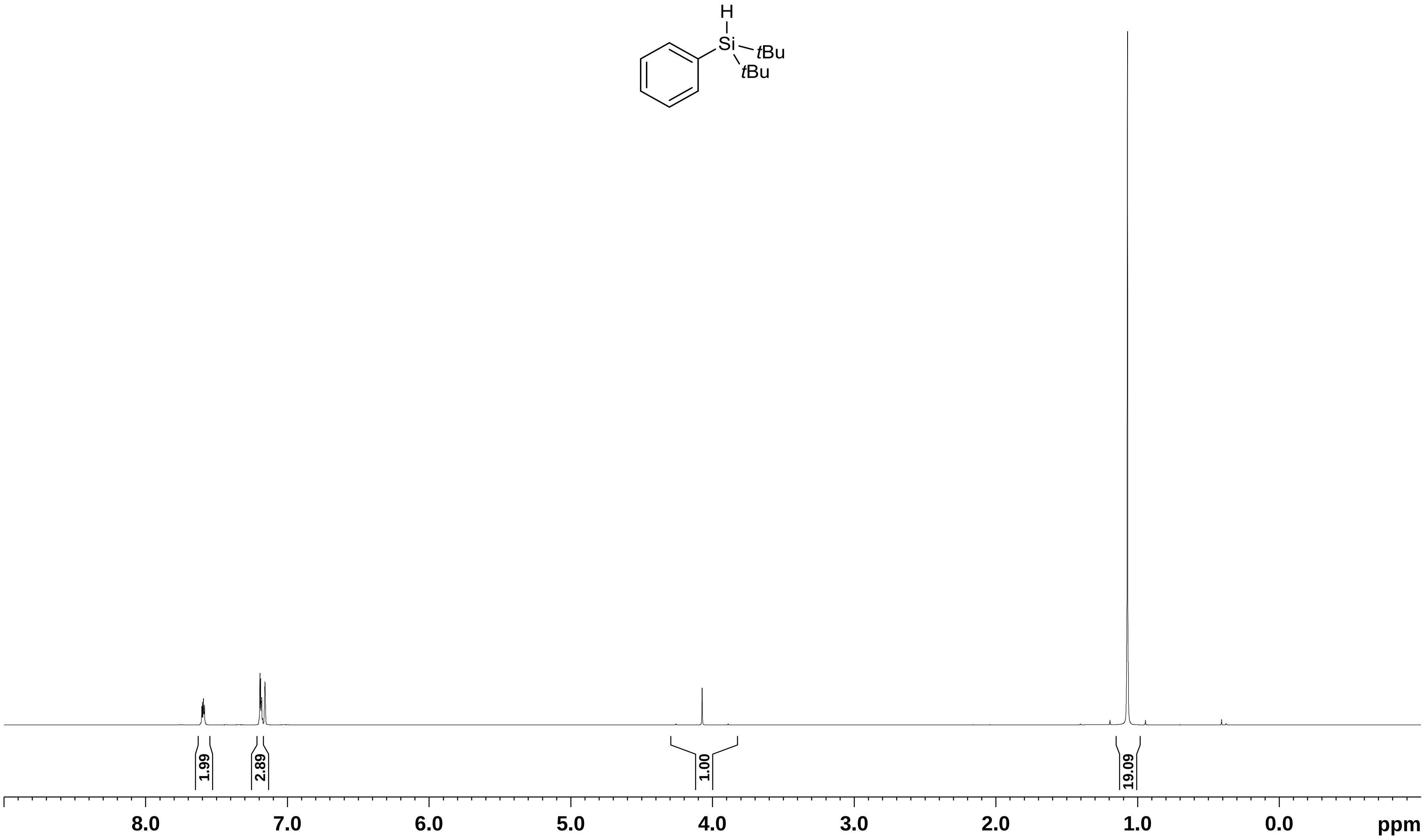

Figure S11. $^{13}C\{^{1}H\}$ spectrum NMR (126 MHz, C_6D_6, 300 K) of *t*Bu_2PhSiH.

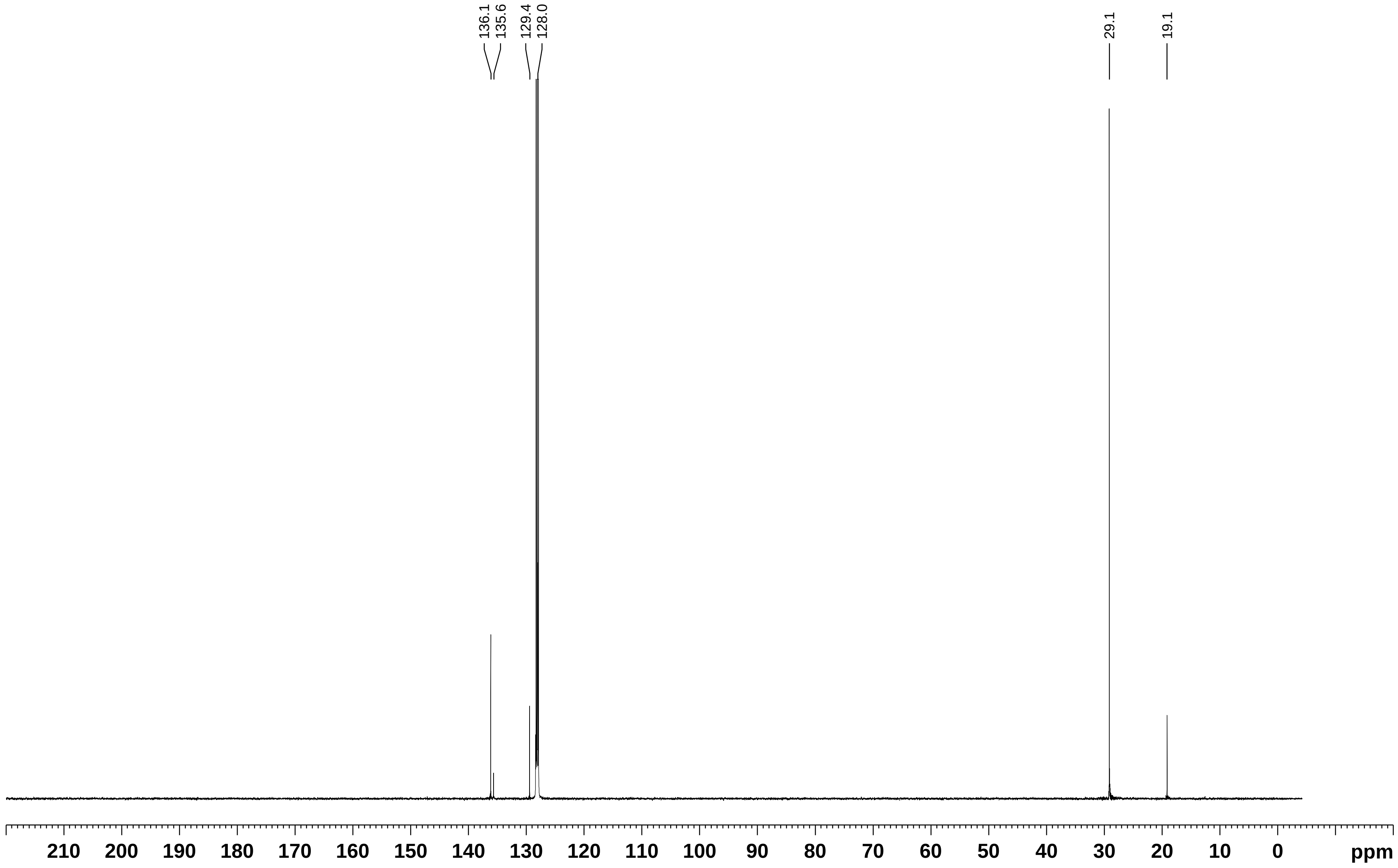

Figure S12. ^{29}Si DEPT NMR spectrum (99 MHz, C_6D_6, 300 K, optimized for J = 200 Hz) of tBu$_2$PhSiH.

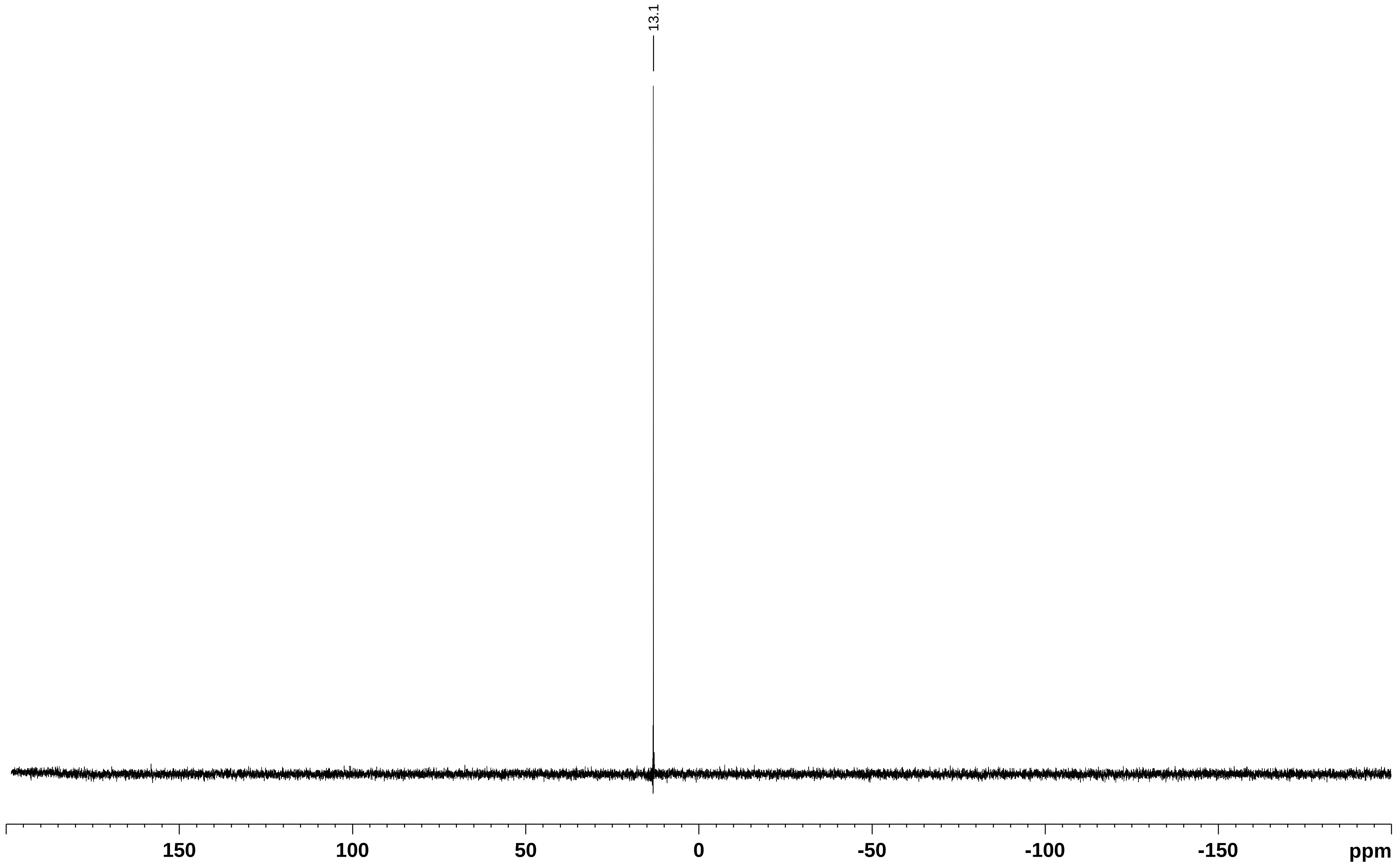

Figure S13. ^{1}H NMR spectrum (500 MHz, CD_2Cl_2, 300 K) of $Ph_3C^+[CHB_{11}H_5Br_6]$ (* = toluene).

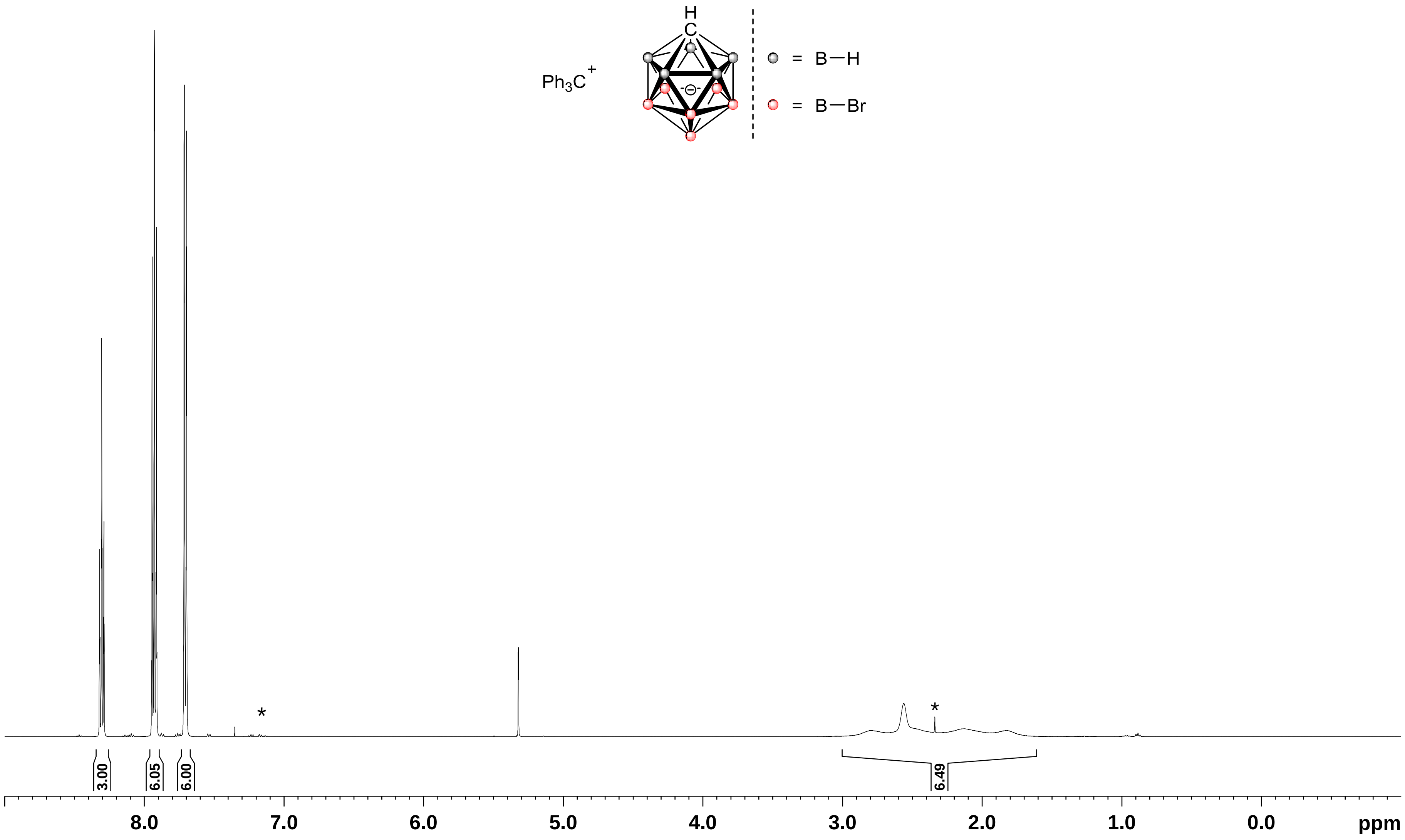

Figure S14. ^{11}B NMR spectrum (161 MHz, CD_2Cl_2, 300 K) of $Ph_3C^+[CHB_{11}H_5Br_6]$.

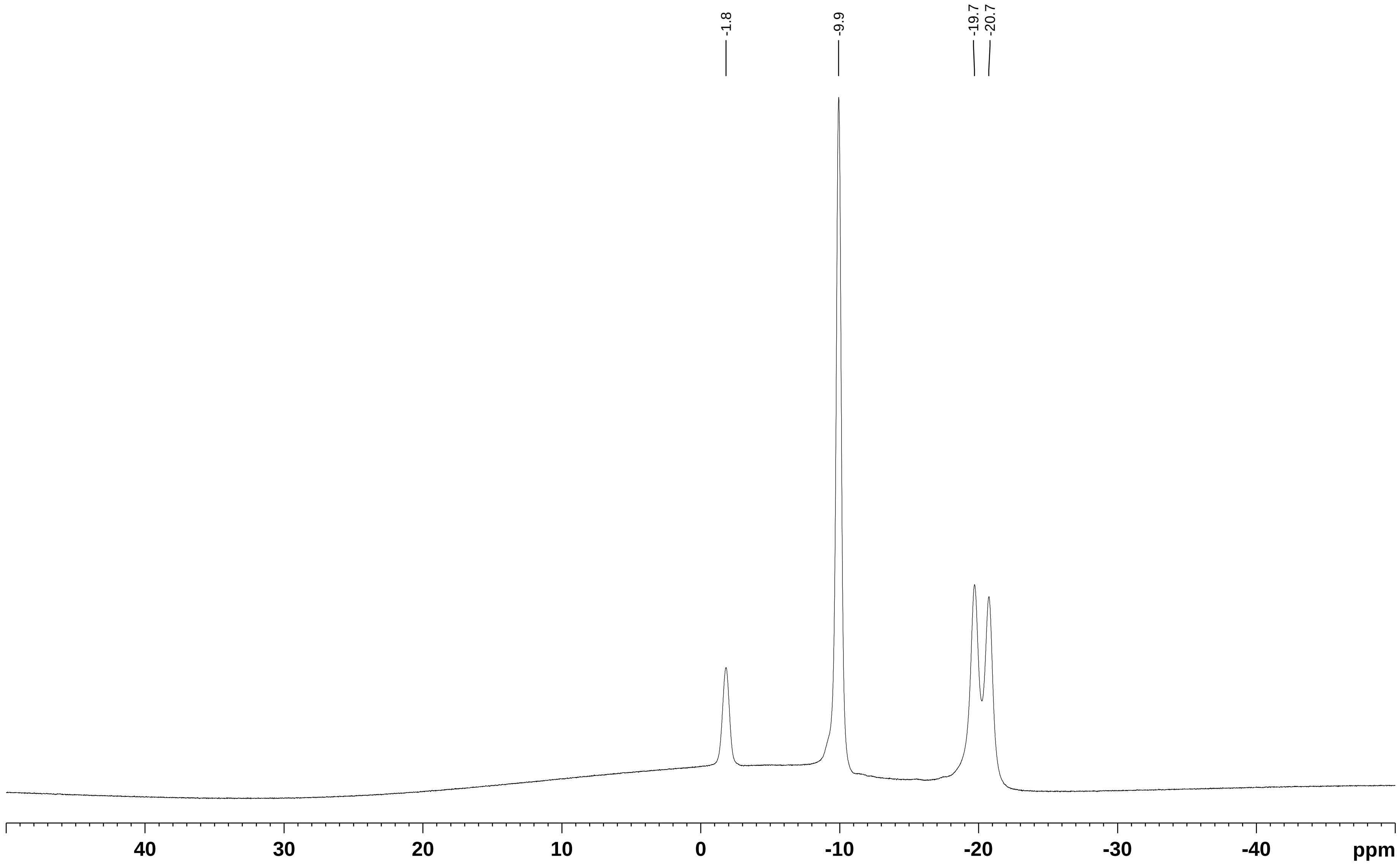

Figure S15. $^{13}C\{^{1}H\}$ NMR spectrum (126 MHz, CD_2Cl_2, 300 K) of $Ph_3C^{+}[CHB_{11}H_5Br_6]$.

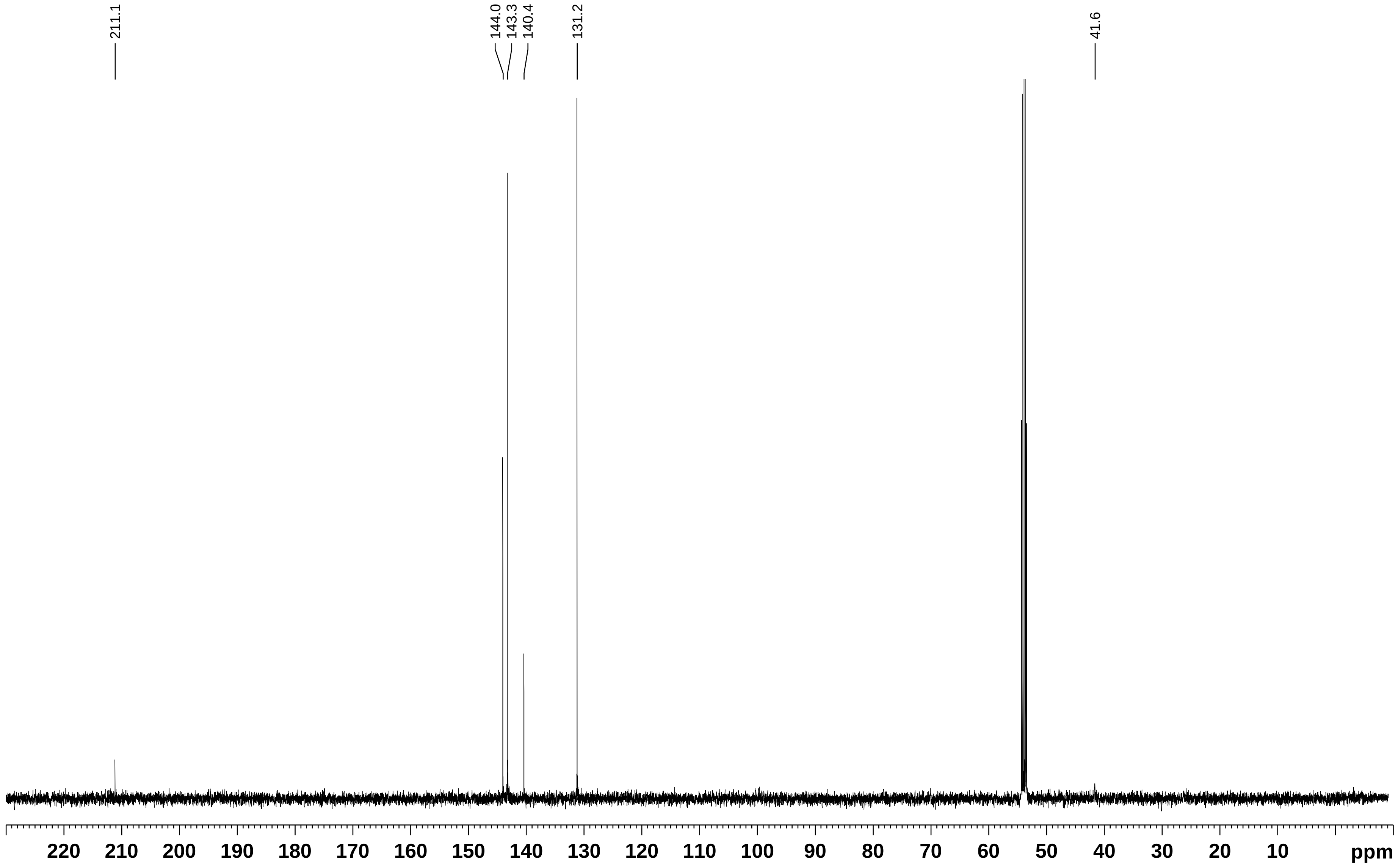

Figure S16. ^{1}H NMR spectrum (500 MHz, *o*-$Cl_2C_6D_4$, 298 K) of $(C_6Me_5)_3Si^+[B(C_6F_5)_4]^-$ from the reaction of $Me(C_6Me_5)_2SiH$ with $Ph_3C^+[B(C_6F_5)_4]^-$.

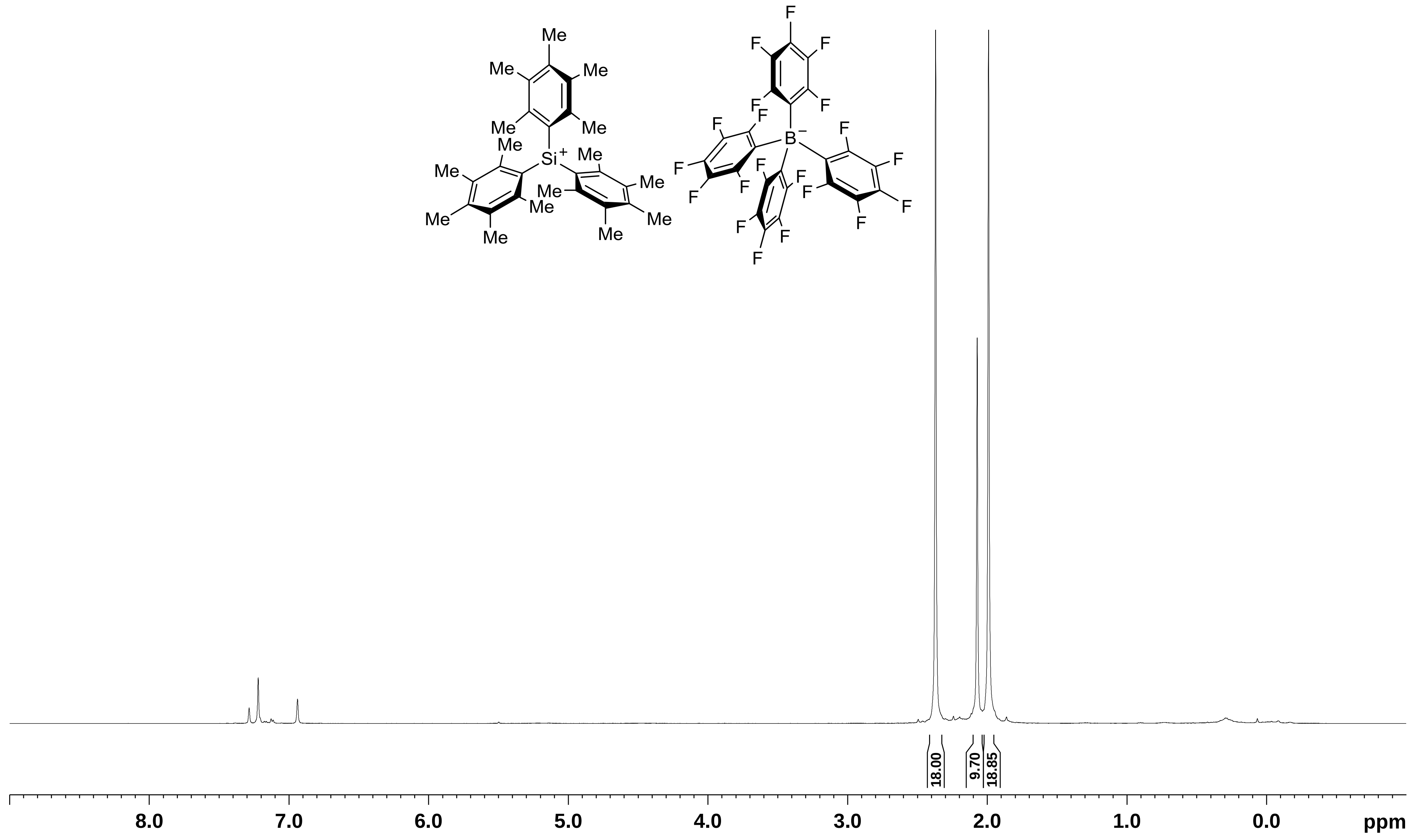

Figure S17. $^{11}B\{^{1}H\}$ NMR spectrum (161 MHz, *o*-$Cl_2C_6D_4$, 298 K) of $(C_6Me_5)_3Si^{+}[B(C_6F_5)_4]^{-}$ from the reaction of $Me(C_6Me_5)_2SiH$ with $Ph_3C^{+}[B(C_6F_5)_4]^{-}$.

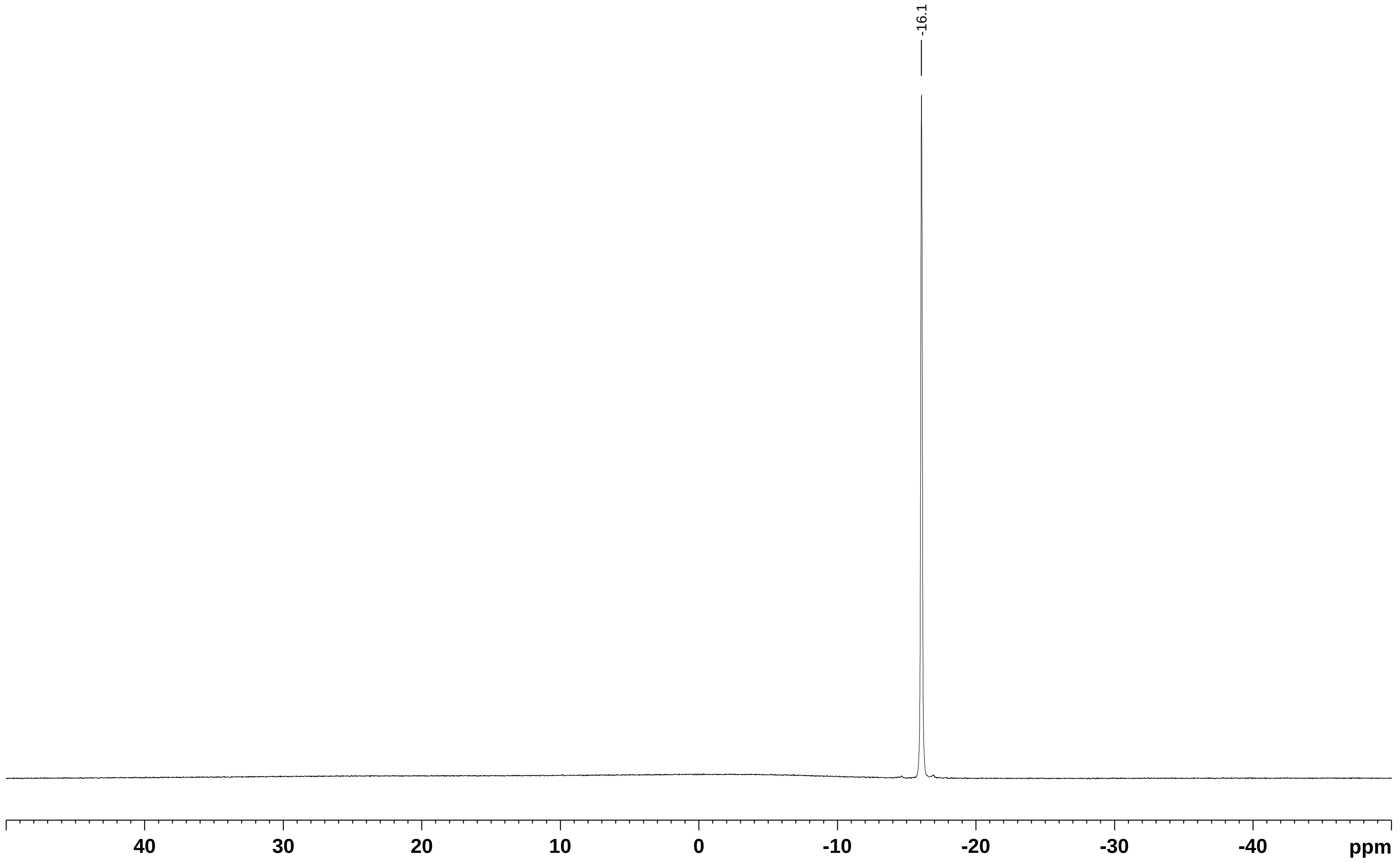

Figure S18. $^{13}C\{^{1}H\}$ NMR spectrum (126 MHz, *o*-$Cl_2C_6D_4$, 298 K) of $(C_6Me_5)_3Si^+[B(C_6F_5)_4]^-$ from the reaction of $Me(C_6Me_5)_2SiH$ with $Ph_3C^+[B(C_6F_5)_4]^-$.

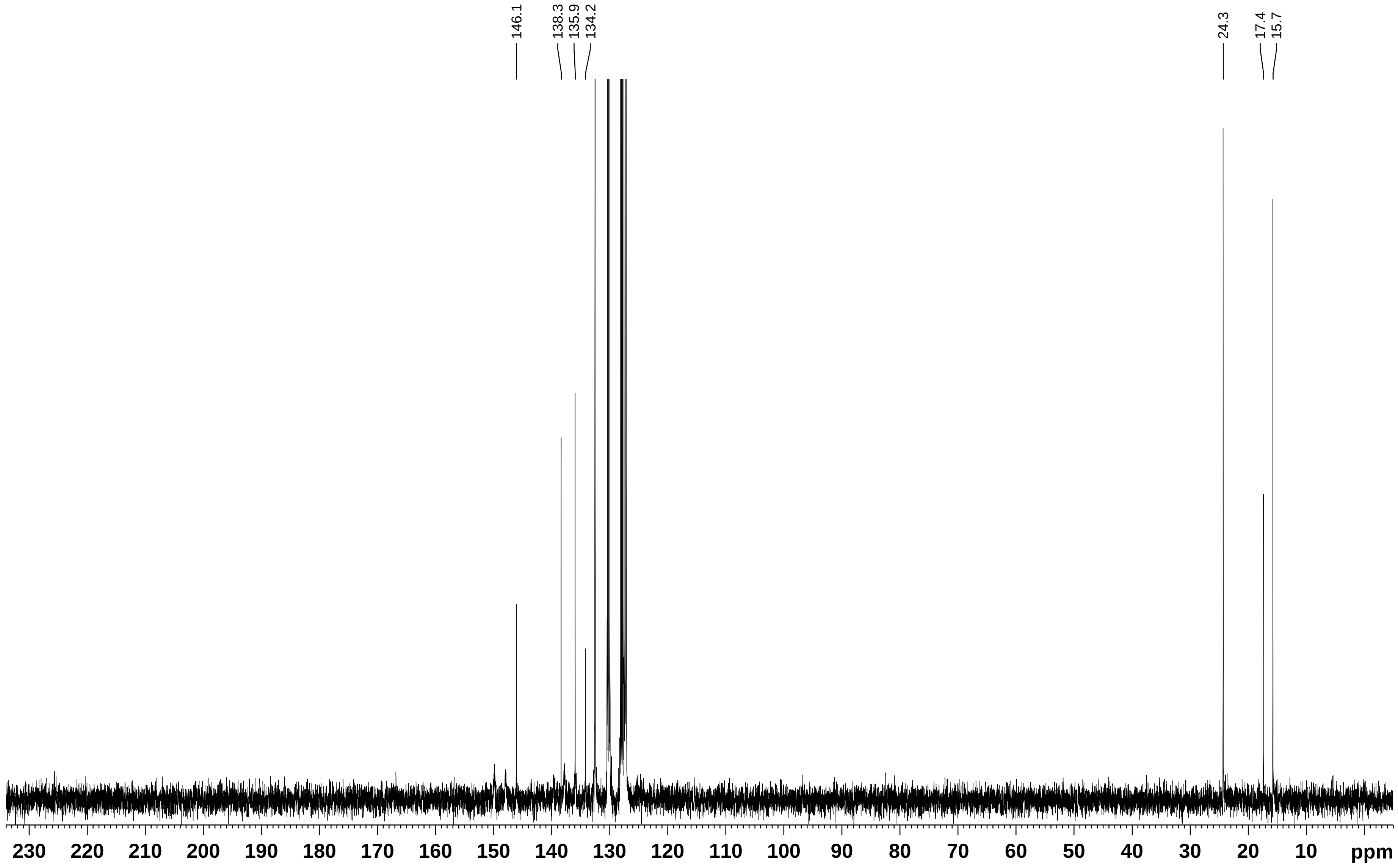

Figure S19. ^{19}F NMR spectrum (471 MHz, *o*-$Cl_2C_6D_4$, 298 K) of $(C_6Me_5)_3Si^+[B(C_6F_5)_4]^-$ from the reaction of $Me(C_6Me_5)_2SiH$ with $Ph_3C^+[B(C_6F_5)_4]^-$.

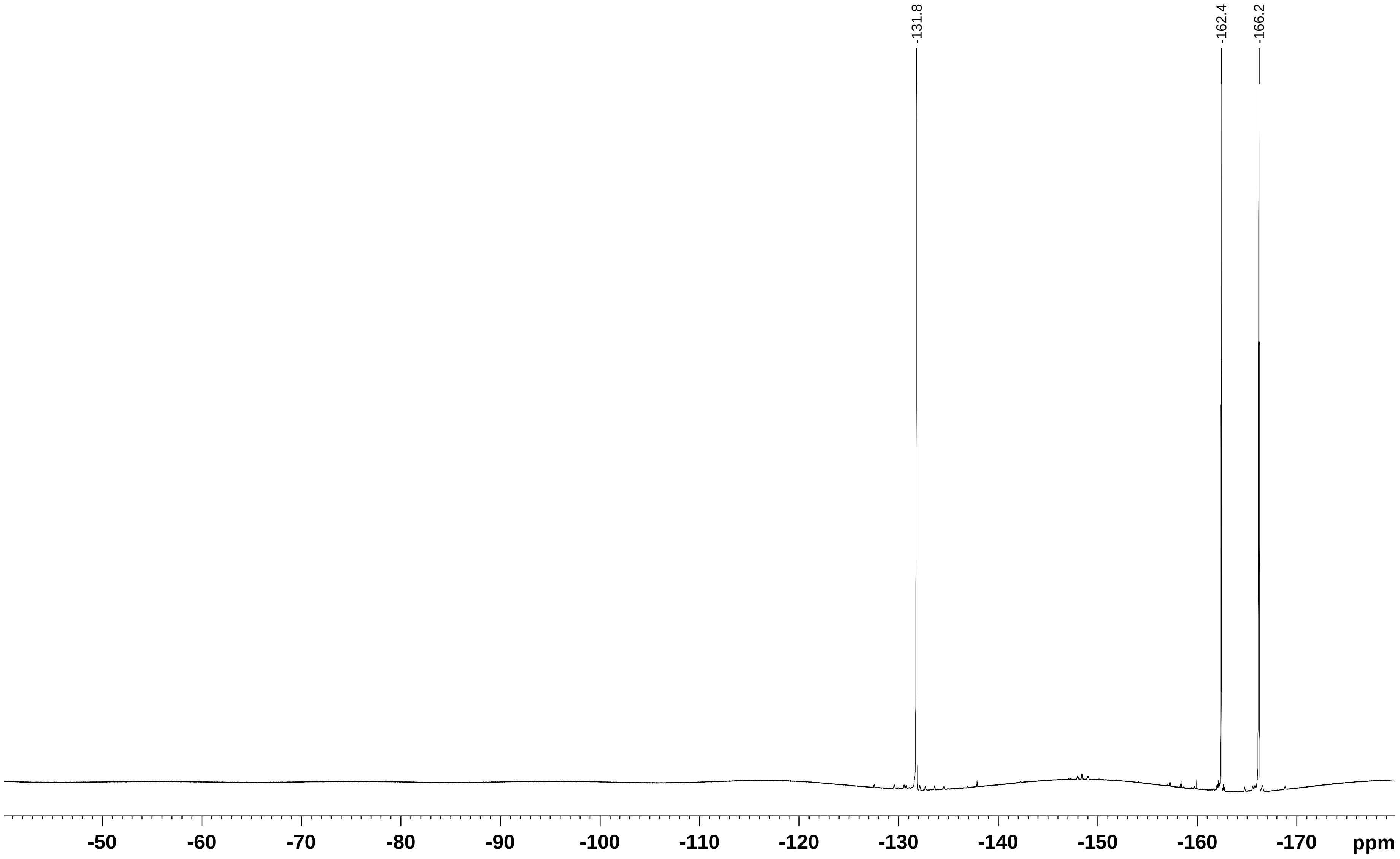

Figure S20. $^{1}H/^{29}Si$ HMQC NMR spectrum (500/99 MHz, *o*-$Cl_2C_6D_4$, 298 K, optimized for *J* = 3 Hz) of $(C_6Me_5)_3Si^+[B(C_6F_5)_4]^-$ from the reaction of $Me(C_6Me_5)_2SiH$ with $Ph_3C^+[B(C_6F_5)_4]^-$.

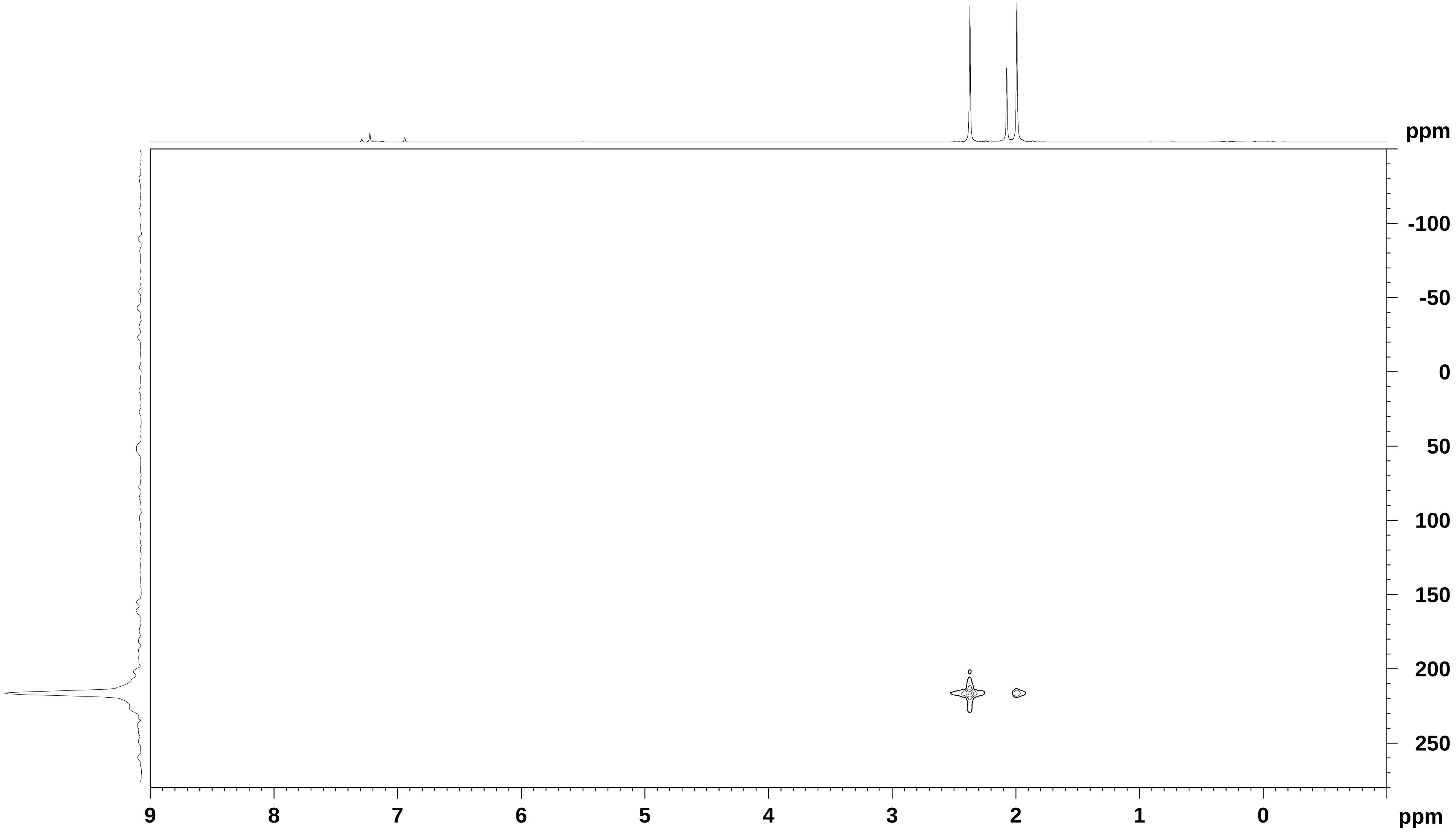

Figure S21. ^{1}H NMR spectrum (700 MHz, *o*-$Cl_2C_6D_4$, 298 K) of $(C_6Me_5)_3Si^+[CHB_{11}H_5Br_6]^-$ from the reaction of $Me(C_6Me_5)_2SiH$ with $Ph_3C^+[CHB_{11}H_5Br_6]^-$ (* = toluene).

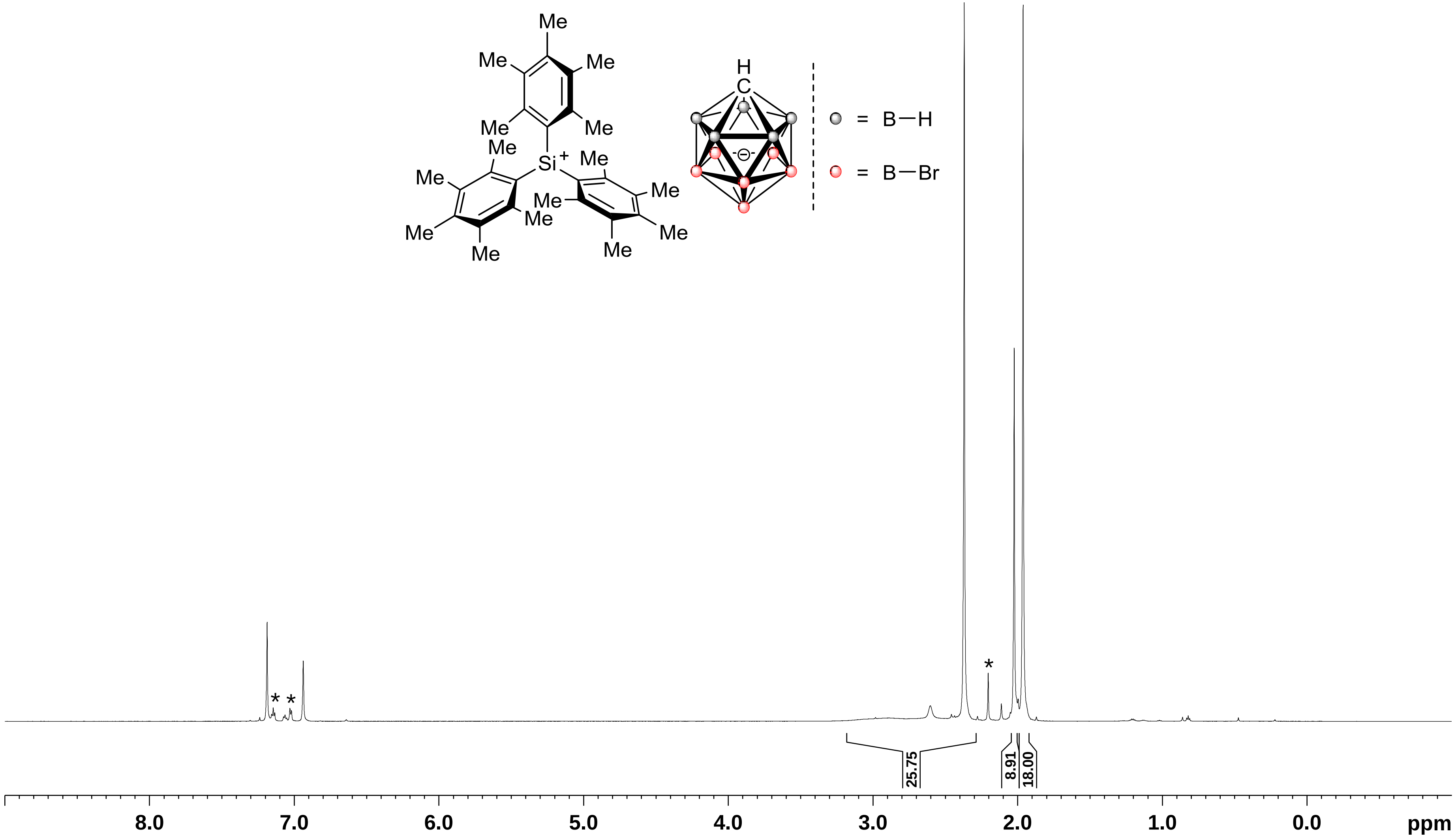

Figure S22. ^{11}B NMR spectrum (224 MHz, *o*-$Cl_2C_6D_4$, 298 K) of $(C_6Me_5)_3Si^+[CHB_{11}H_5Br_6]^-$ from the reaction of $Me(C_6Me_5)_2SiH$ with $Ph_3C^+[CHB_{11}H_5Br_6]^-$.

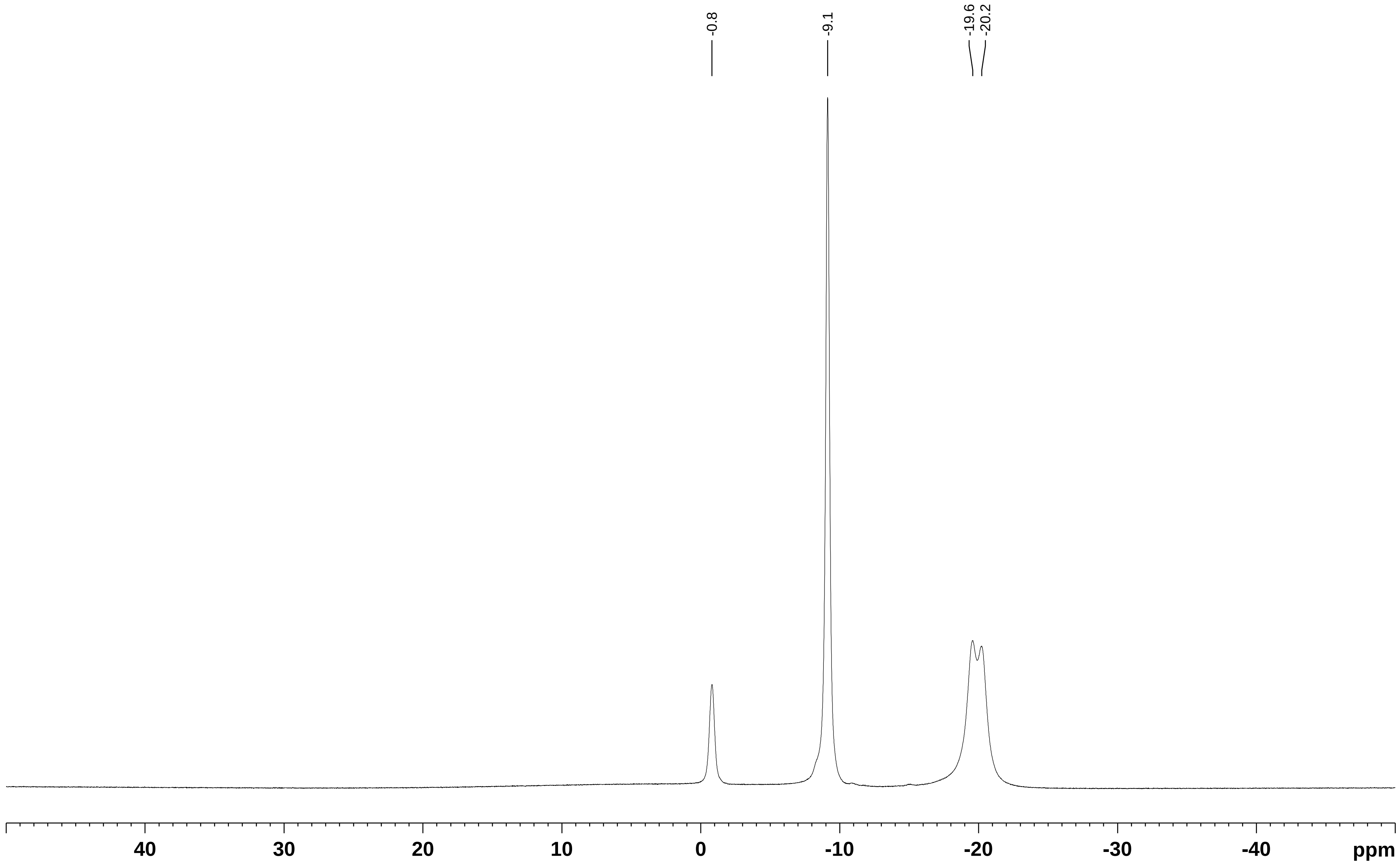

Figure S23. $^{13}C\{^{1}H\}$ NMR spectrum (175 MHz, *o*-$Cl_2C_6D_4$, 298 K) of $(C_6Me_5)_3Si^{+}[CHB_{11}H_5Br_6]^{-}$ from the reaction of $Me(C_6Me_5)_2SiH$ with $Ph_3C^{+}[CHB_{11}H_5Br_6]^{-}$ (* = toluene).

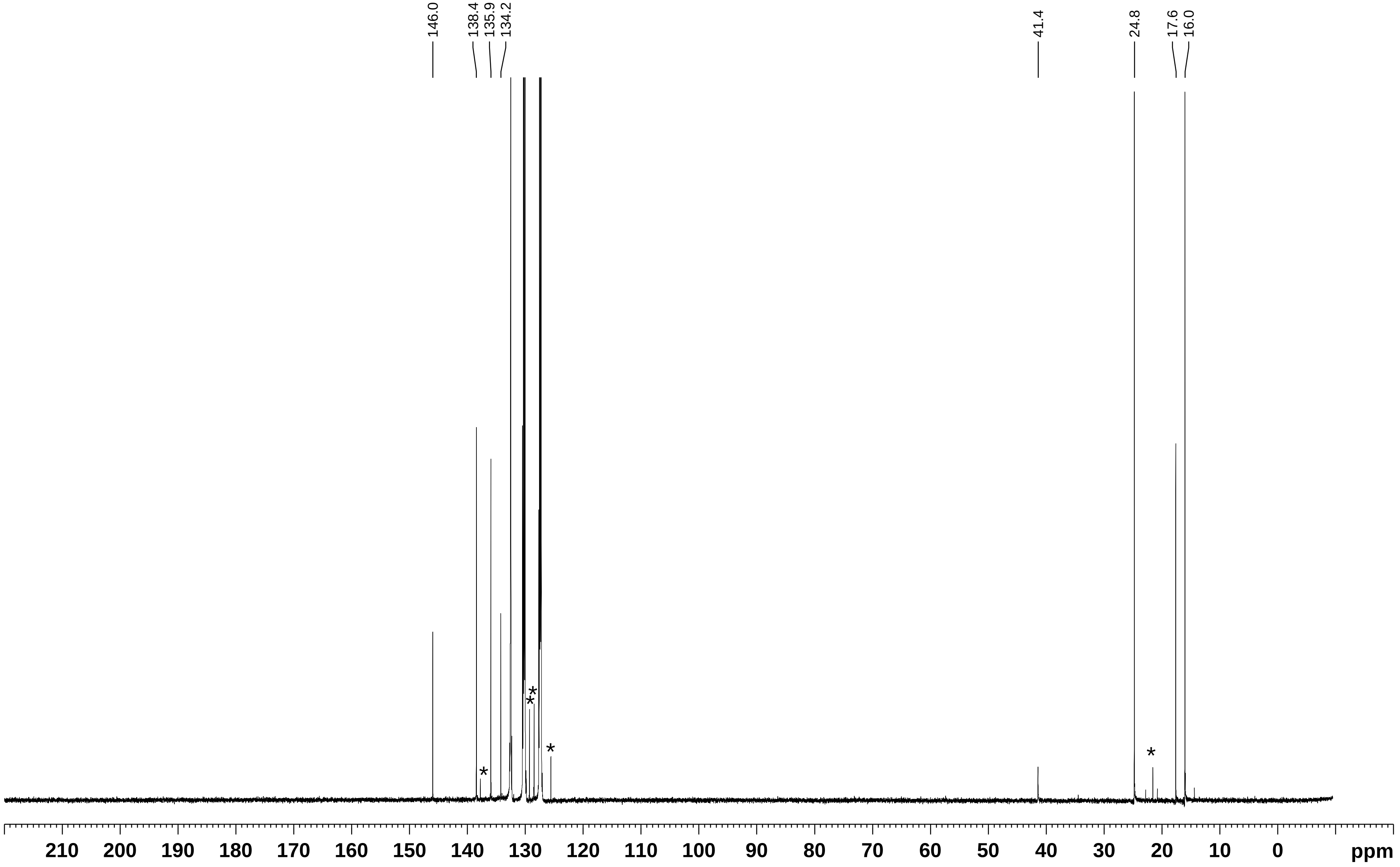

Figure S24. $^{1}H/^{29}Si$ HMQC NMR spectrum (700/139 MHz, *o*-$Cl_2C_6D_4$, 298 K, optimized for J = 3 Hz) of $(C_6Me_5)_3Si^{+}[CHB_{11}H_5Br_6]^{-}$ from the reaction of $Me(C_6Me_5)_2SiH$ with $Ph_3C^{+}[CHB_{11}H_5Br_6]^{-}$.

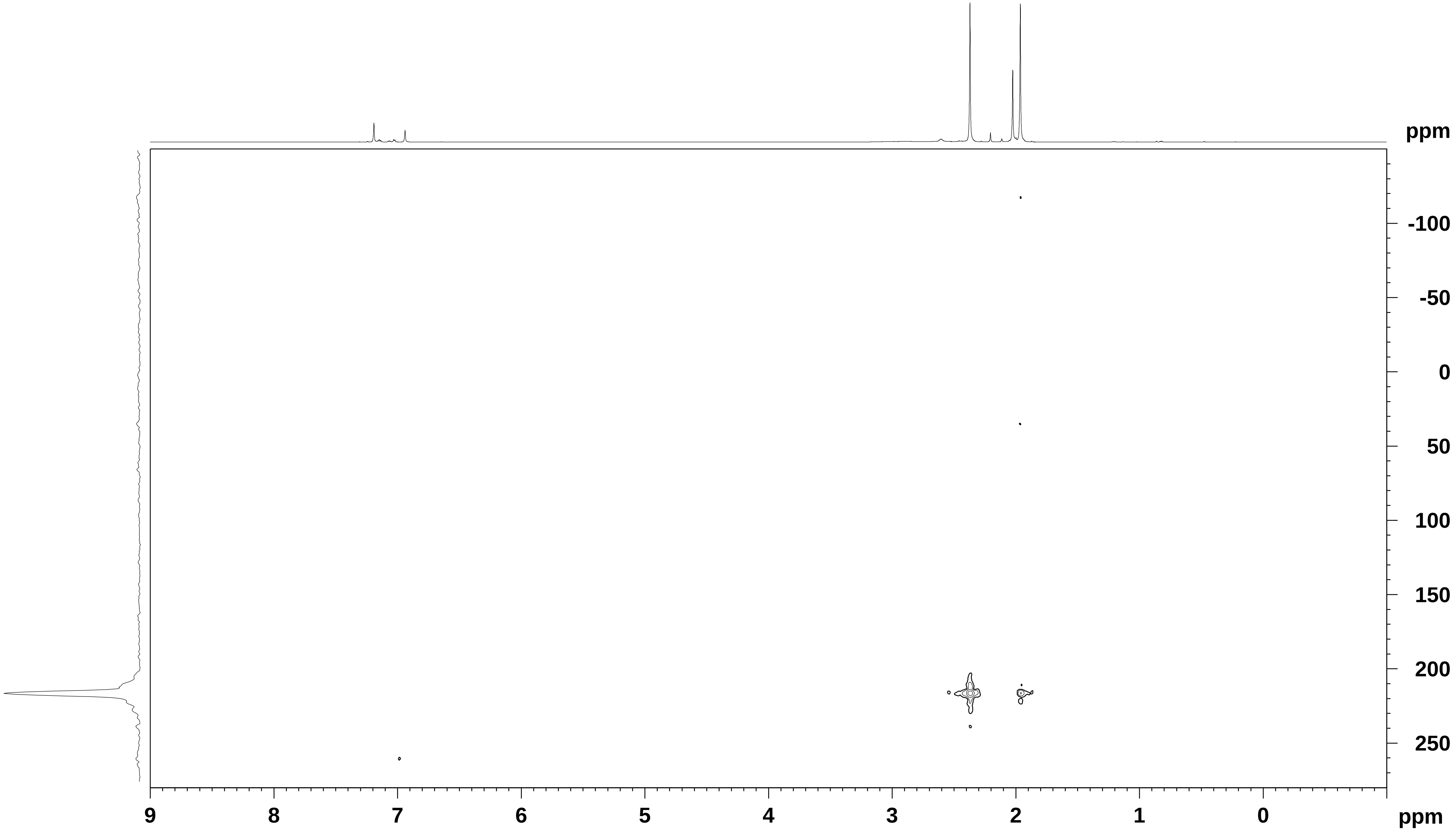

Figure S25. ^{19}F NMR spectra (659 MHz, *o*-$Cl_2C_6D_4$, 298 K) from the reactions of $MePh_2SiH$ with $Ph_3C^+[B(C_6F_5)_4]^-$ (top) and Me_2PhSiH with $Ph_3C^+[B(C_6F_5)_4]^-$ (middle) as well as a clean sample of $B(C_6F_5)_3$ (bottom; \$ = $[B(C_6F_5)_4]^-$).

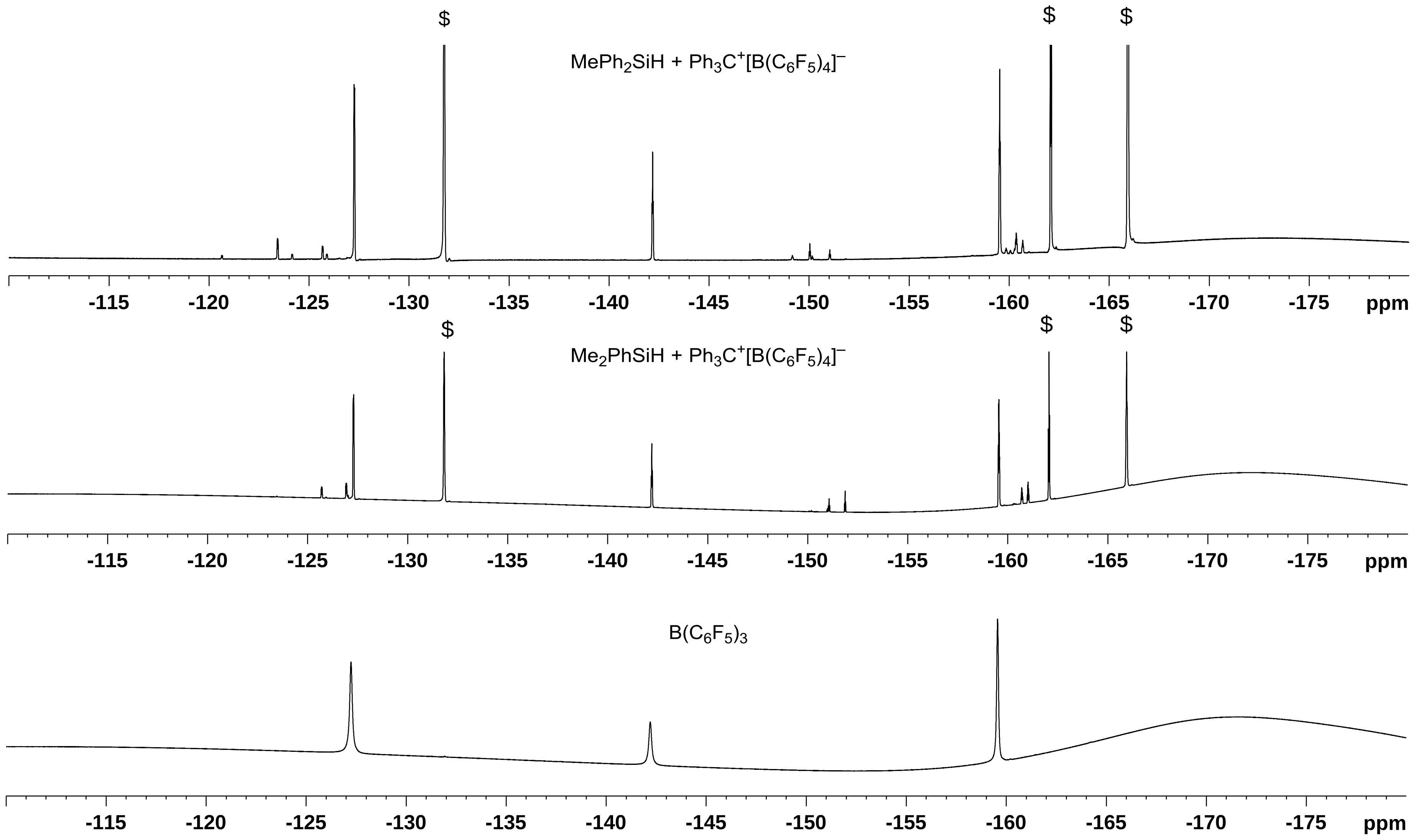

Figure S26. ^{1}H NMR spectrum (500 MHz, *o*-$Cl_2C_6D_4$, 300 K) of $MePh_2Si^+[CHB_{11}H_5Br_6]^-$ from the reaction of $MePh_2SiH$ with $Ph_3C^+[CHB_{11}H_5Br_6]^-$ (+ = *n*-pentane, * = toluene, # = $Me_2PhSi^+[CHB_{11}H_5Br_6]^-$).

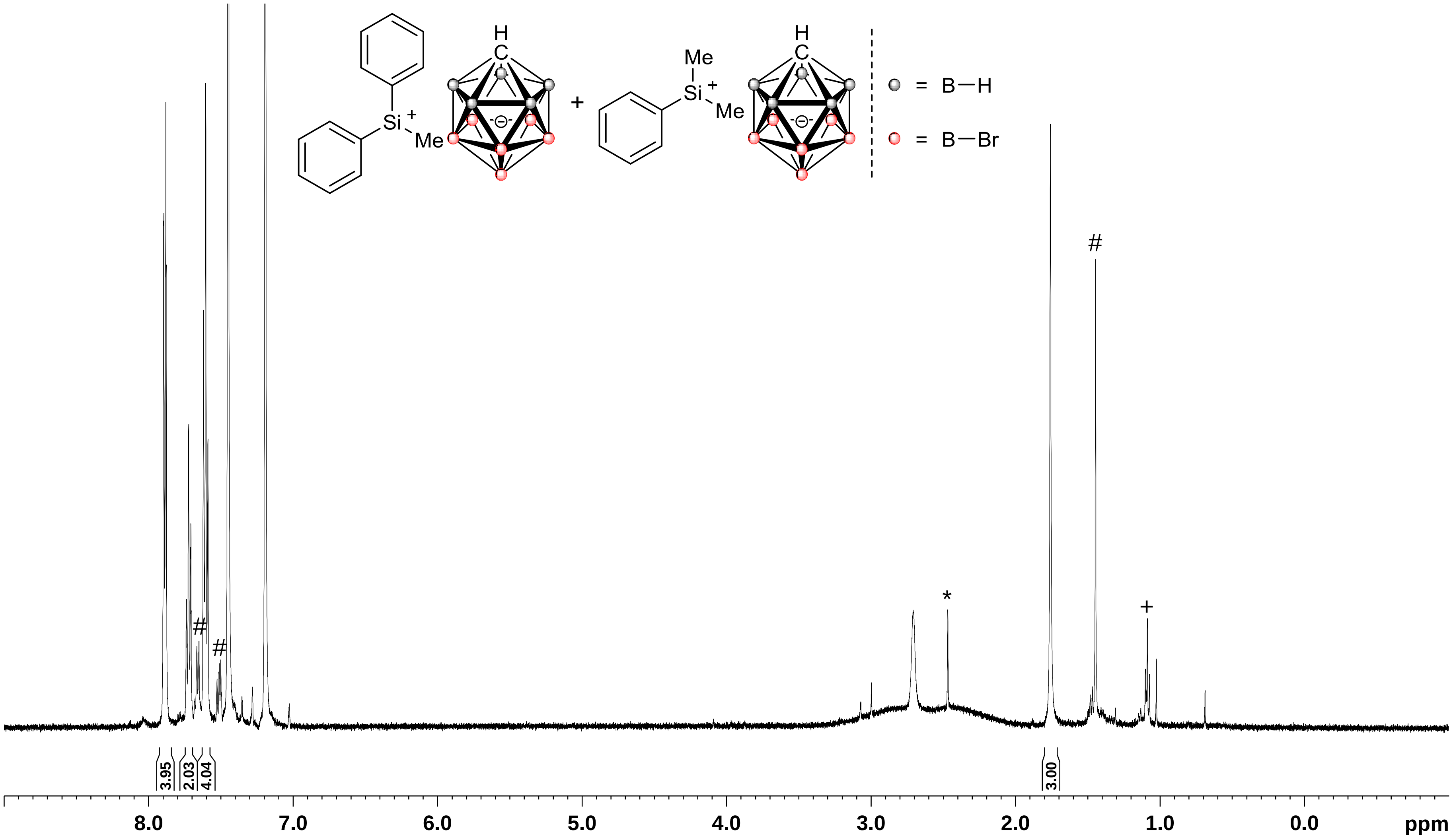

Figure S27. ^{11}B NMR spectrum (161 MHz, *o*-$Cl_2C_6D_4$, 300 K) of $MePh_2Si^+[CHB_{11}H_5Br_6]^-$ from the reaction of $MePh_2SiH$ with $Ph_3C^+[CHB_{11}H_5Br_6]^-$.

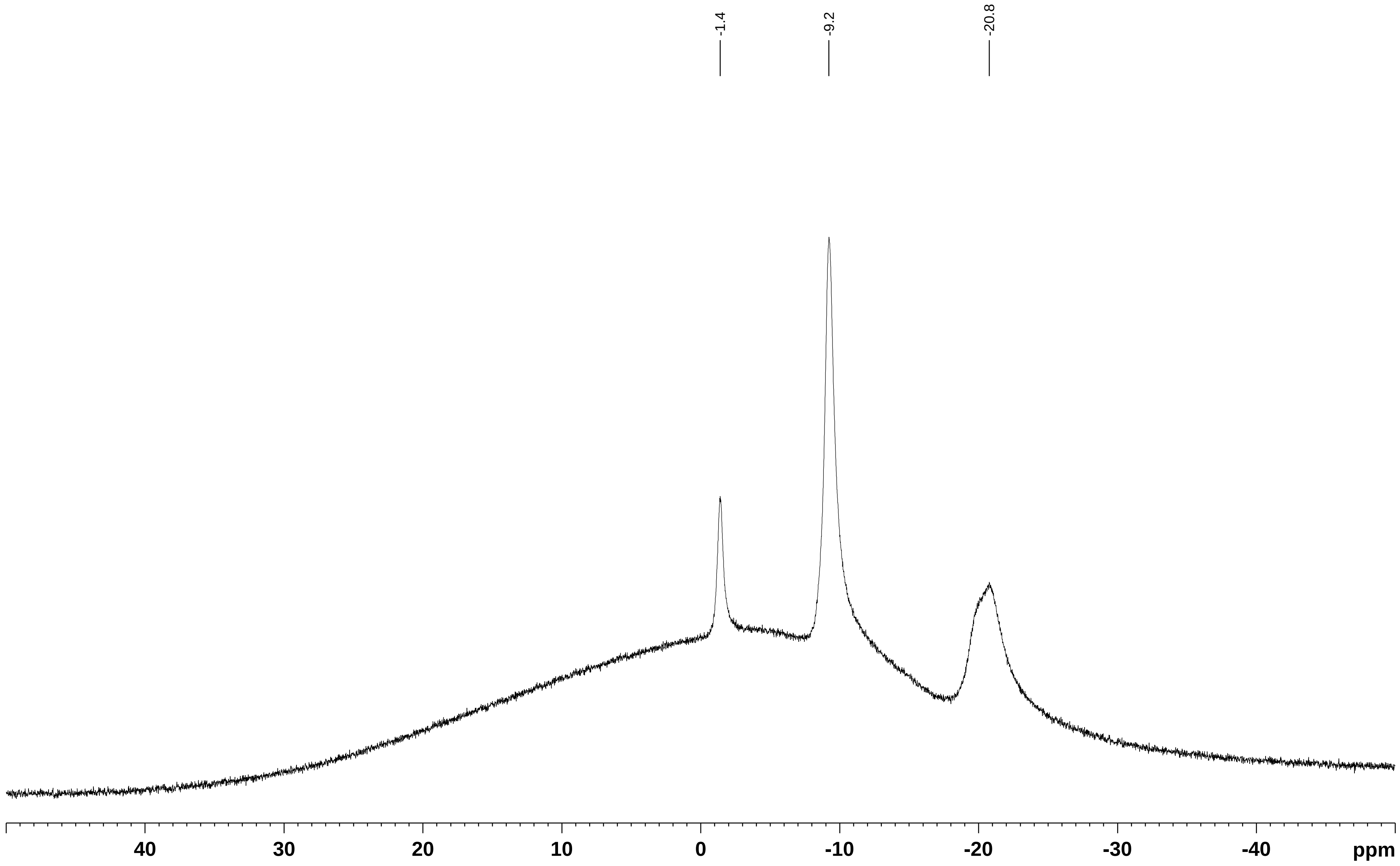

Figure S28. $^{13}C\{^{1}H\}$ NMR spectrum (126 MHz, *o*-$Cl_2C_6D_4$, 300 K) of $MePh_2Si^{+}[CHB_{11}H_5Br_6]^{-}$ from the reaction of $MePh_2SiH$ with $Ph_3C^{+}[CHB_{11}H_5Br_6]^{-}$.

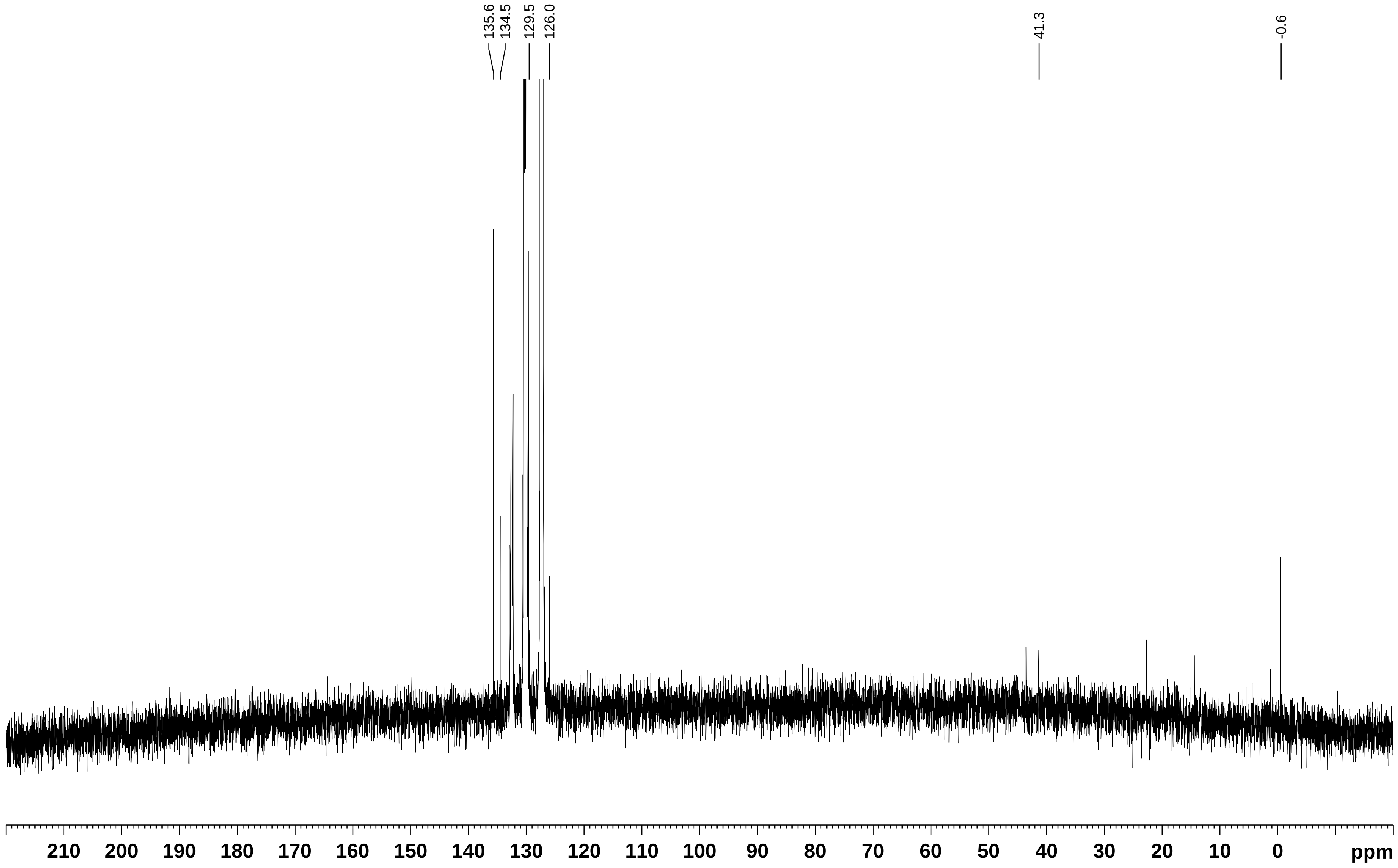

Figure S29. $^1H/^{29}Si$ HMQC NMR spectrum (500/99 MHz, *o*-$Cl_2C_6D_4$, 300 K, optimized for *J* = 7 Hz) of $MePh_2Si^+[CHB_{11}H_5Br_6]^-$ from the reaction of $MePh_2SiH$ with $Ph_3C^+[CHB_{11}H_5Br_6]^-$.

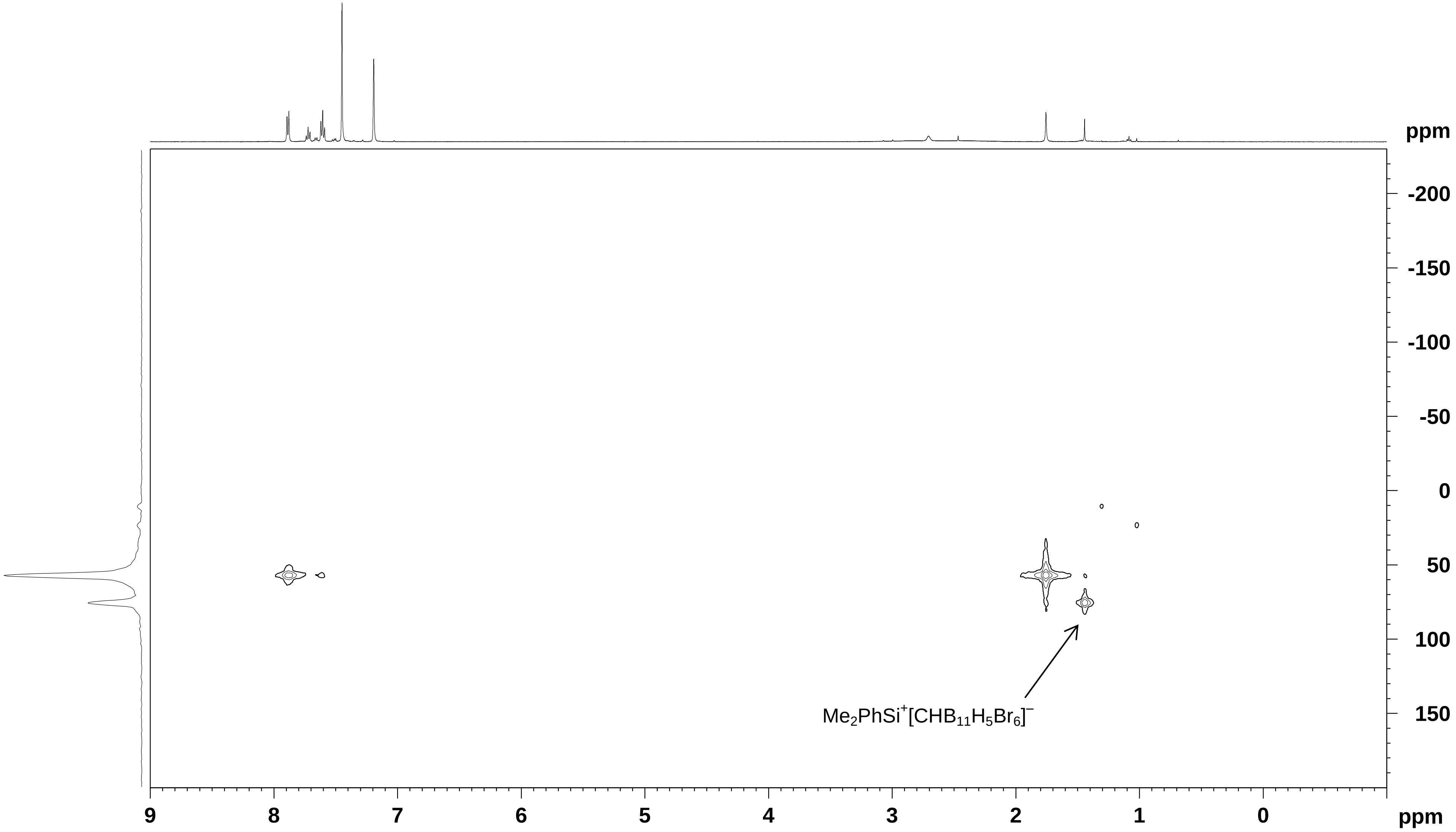

Figure S30. ^{1}H NMR spectrum (500 MHz, *o*-$Cl_2C_6D_4$, 300 K) of $Me_3Si^+[CHB_{11}H_5Br_6]^-$ from the reaction of Me_2PhSiH with $Ph_3C^+[CHB_{11}H_5Br_6]^-$ (+ = *n*-pentane, # = impurity).

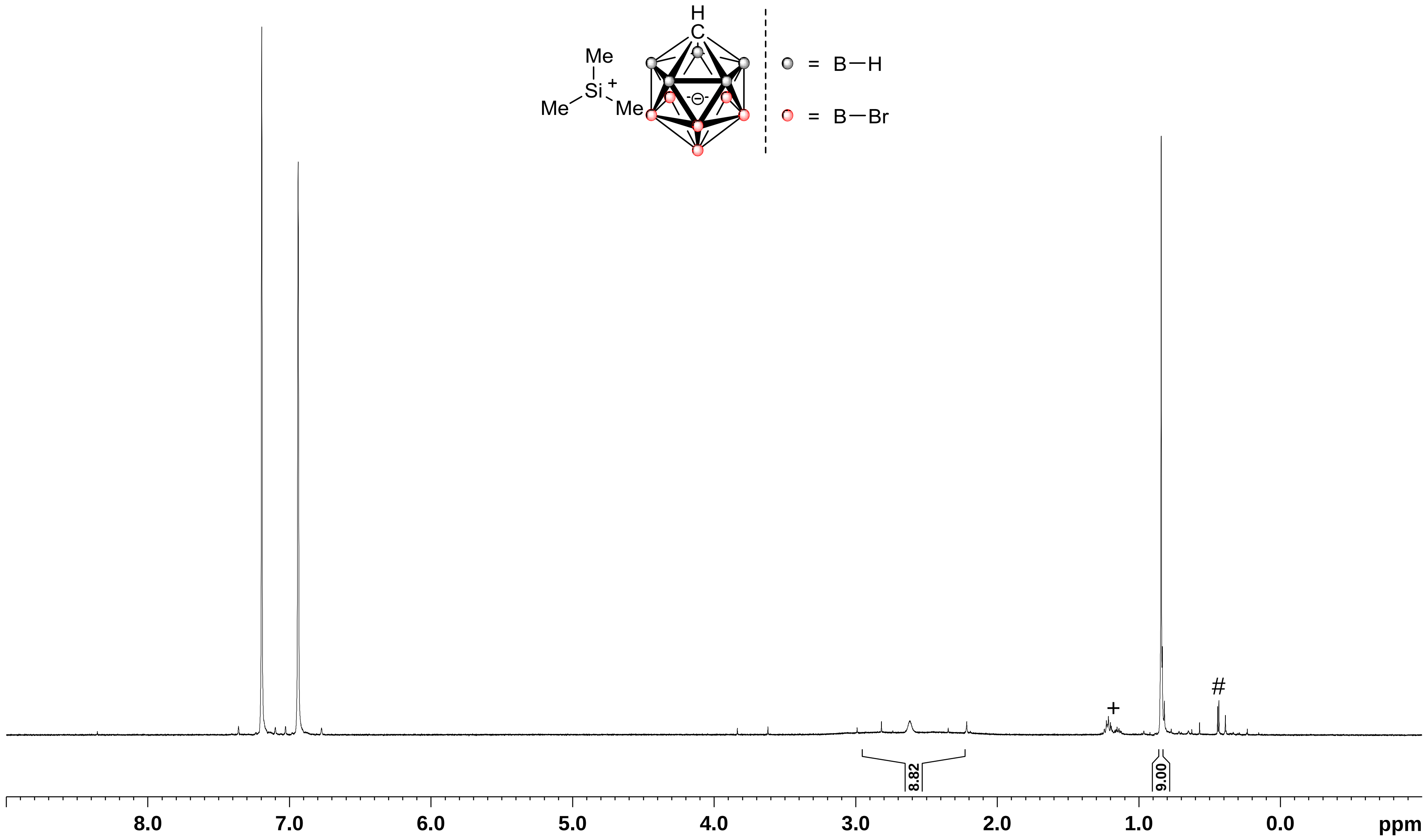

Figure S31. ^{11}B NMR spectrum (161 MHz, *o*-$Cl_2C_6D_4$, 300 K) of $Me_3Si^+[CHB_{11}H_5Br_6]^-$ from the reaction of Me_2PhSiH with $Ph_3C^+[CHB_{11}H_5Br_6]^-$.

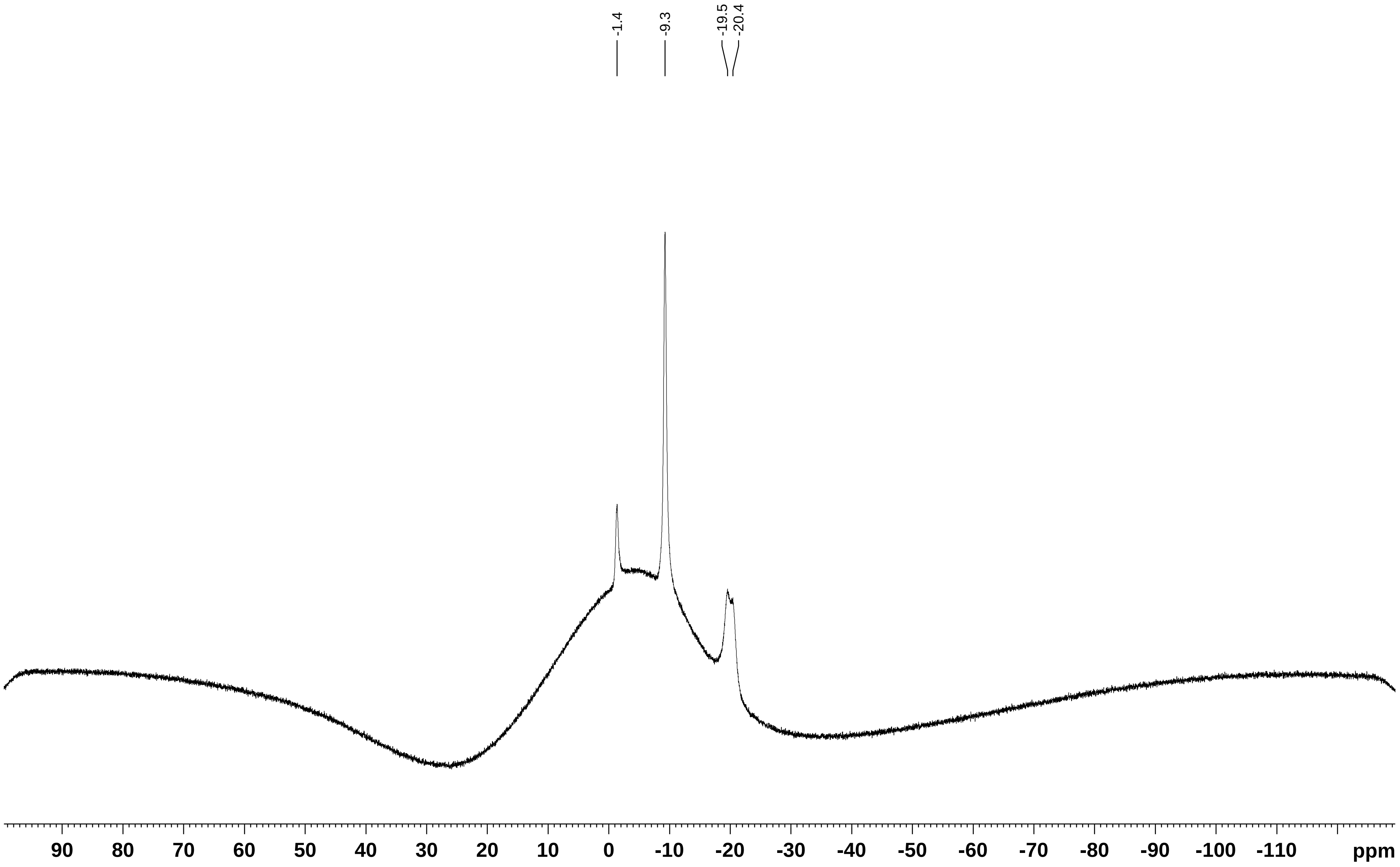

Figure S32. $^{13}C\{^1H\}$ DEPT NMR spectrum (126 MHz, *o*-$Cl_2C_6D_4$, 300 K) of $Me_3Si^+[CHB_{11}H_5Br_6]^-$ from the reaction of Me_2PhSiH with $Ph_3C^+[CHB_{11}H_5Br_6]^-$.

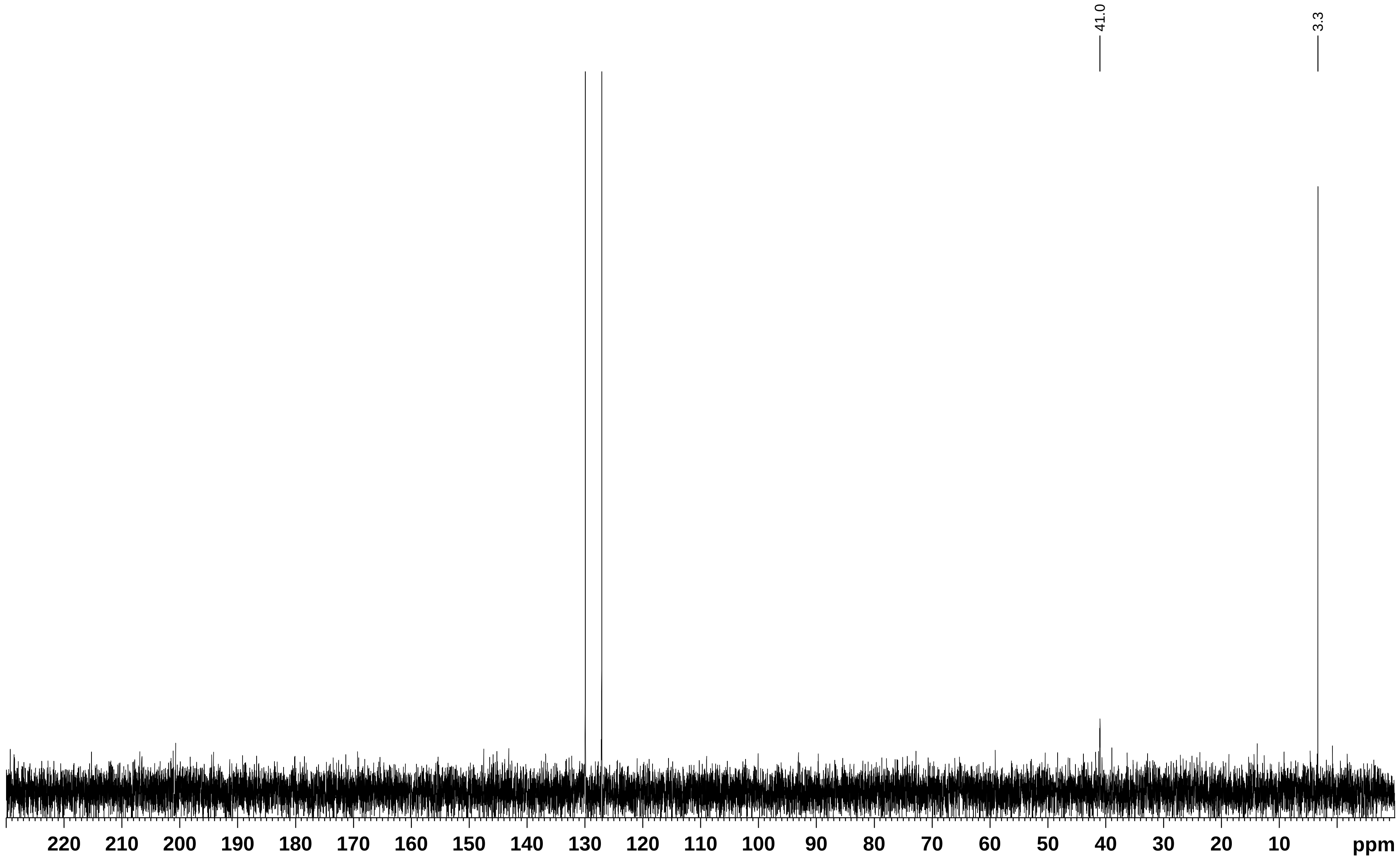

Figure S33. $^{1}H/^{29}Si$ HMQC NMR spectrum (500/99 MHz, *o*-$Cl_2C_6D_4$, 300 K, optimized for *J* = 7 Hz) of $Me_3Si^{+}[CHB_{11}H_5Br_6]^{-}$ from the reaction of Me_2PhSiH with $Ph_3C^{+}[CHB_{11}H_5Br_6]^{-}$.

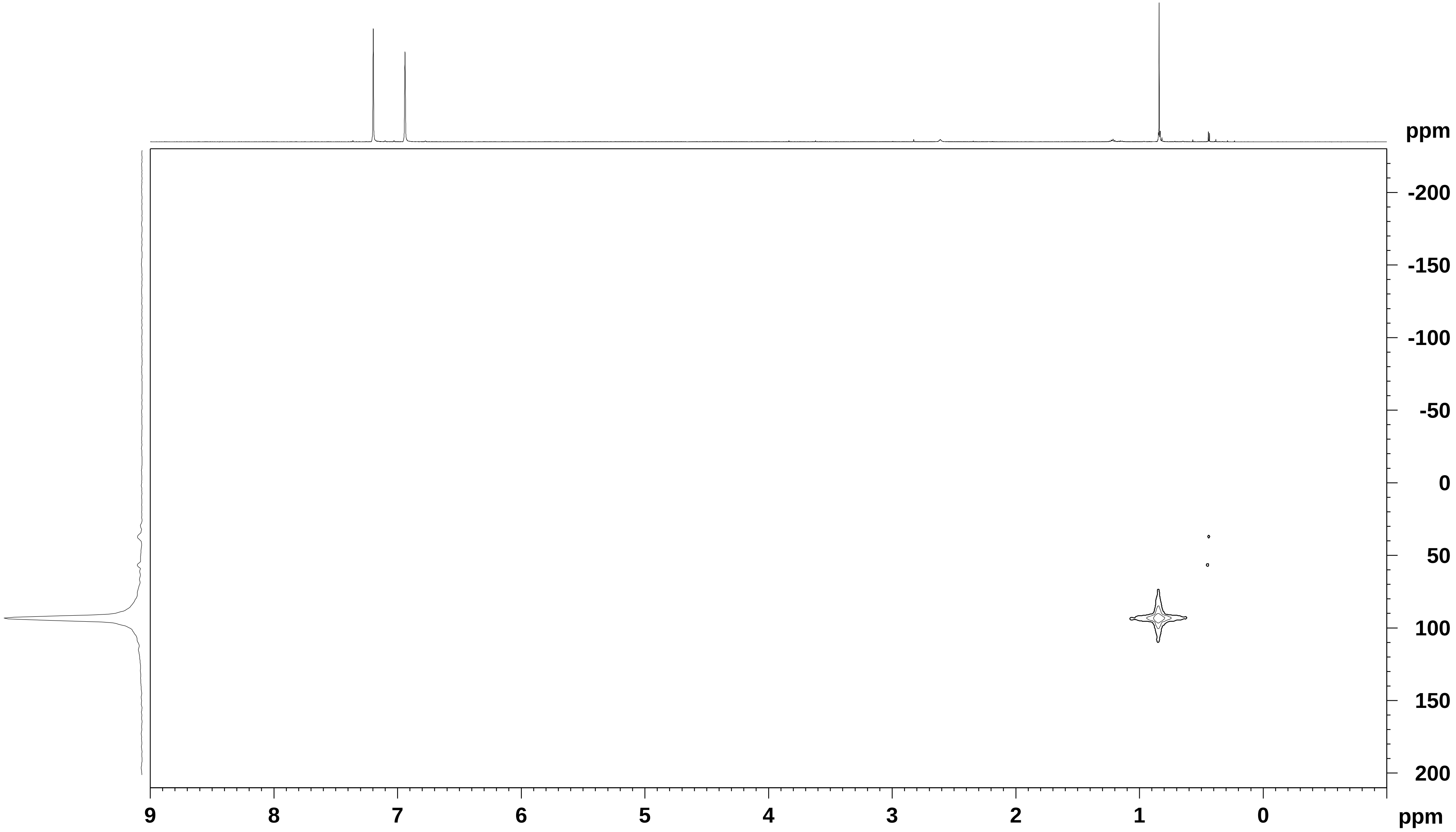

Figure S34. ^{1}H NMR spectrum (500 MHz, *o*-$Cl_2C_6D_4$, 298 K) of $(C_6Me_5)_3Si^{+}[B(C_6F_5)_4]^{-}$ from the reaction of $Me_2(C_6Me_5)SiH$ with $Ph_3C^{+}[B(C_6F_5)_4]^{-}$ (# = impurities).

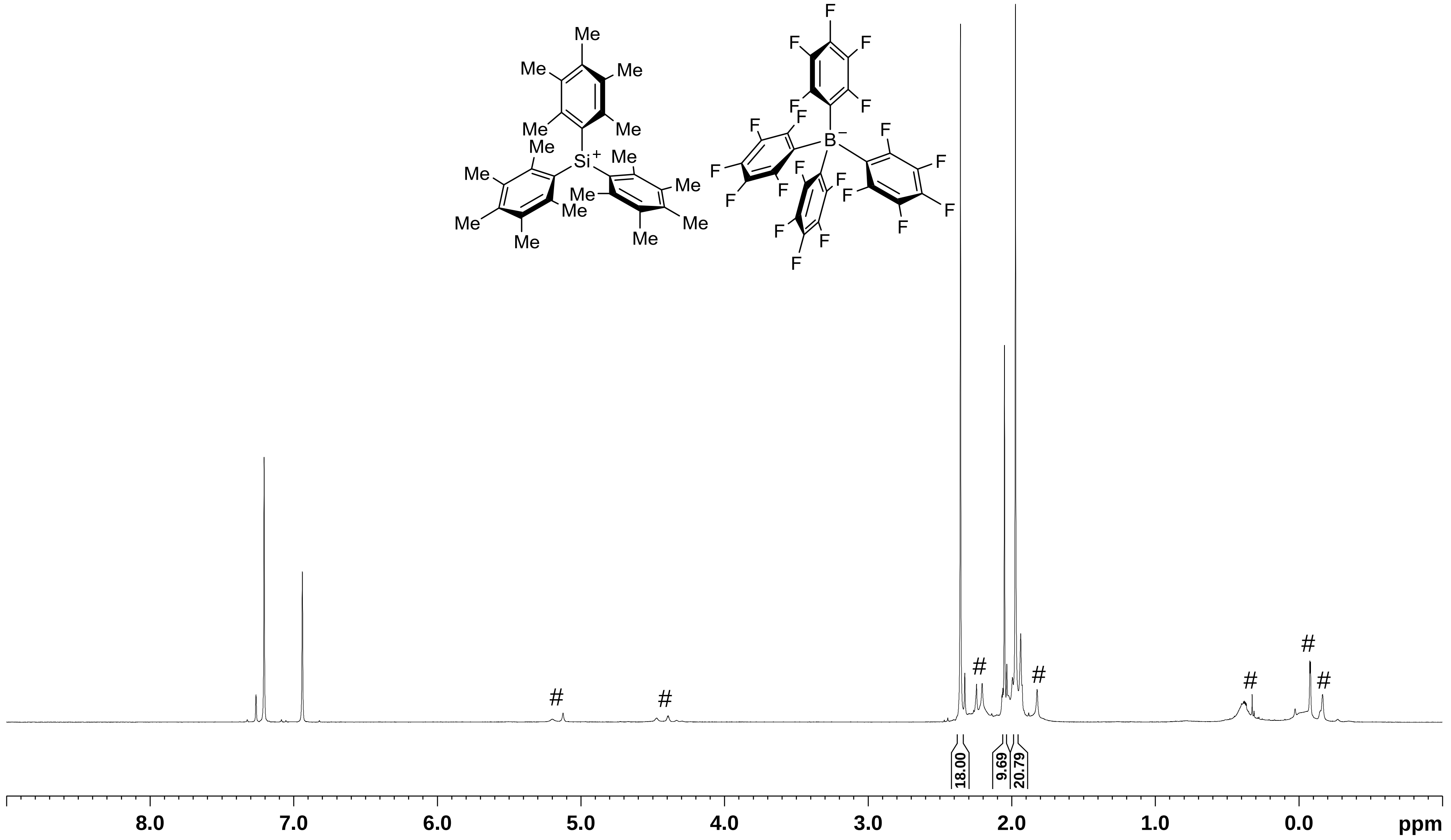

Figure S35. $^{1}H/^{29}Si$ HMQC NMR spectrum (500/99 MHz, *o*-$Cl_2C_6D_4$, 298 K, optimized for *J* = 3 Hz) of $(C_6Me_5)_3Si^+[B(C_6F_5)_4]^-$ from the reaction of $Me_2(C_6Me_5)SiH$ with $Ph_3C^+[B(C_6F_5)_4]^-$.

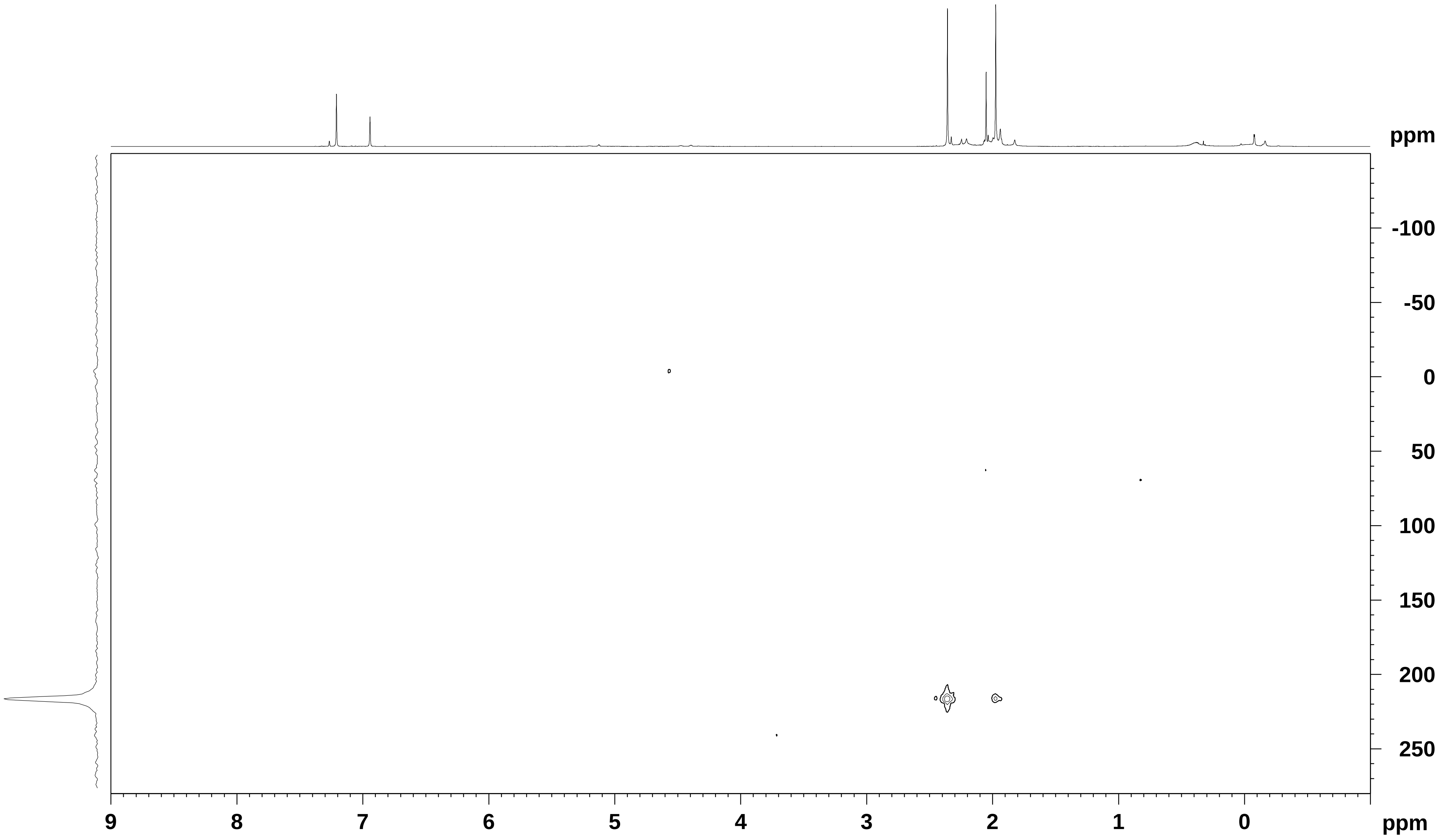

Figure S36. ^{1}H NMR spectrum (500 MHz, *o*-$Cl_2C_6D_4$, 300 K) of $Me_3Si^+[CHB_{11}H_5Br_6]^-$ with small amounts of $(C_6Me_5)_3Si^+[CHB_{11}H_5Br_6]^-$ from the reaction of $Me_2(C_6Me_5)SiH$ with $Ph_3C^+[CHB_{11}H_5Br_6]^-$ (* = impurity,+ = *n*-pentane, # = $(C_6Me_5)_3Si^+[CHB_{11}H_5Br_6]^-$).

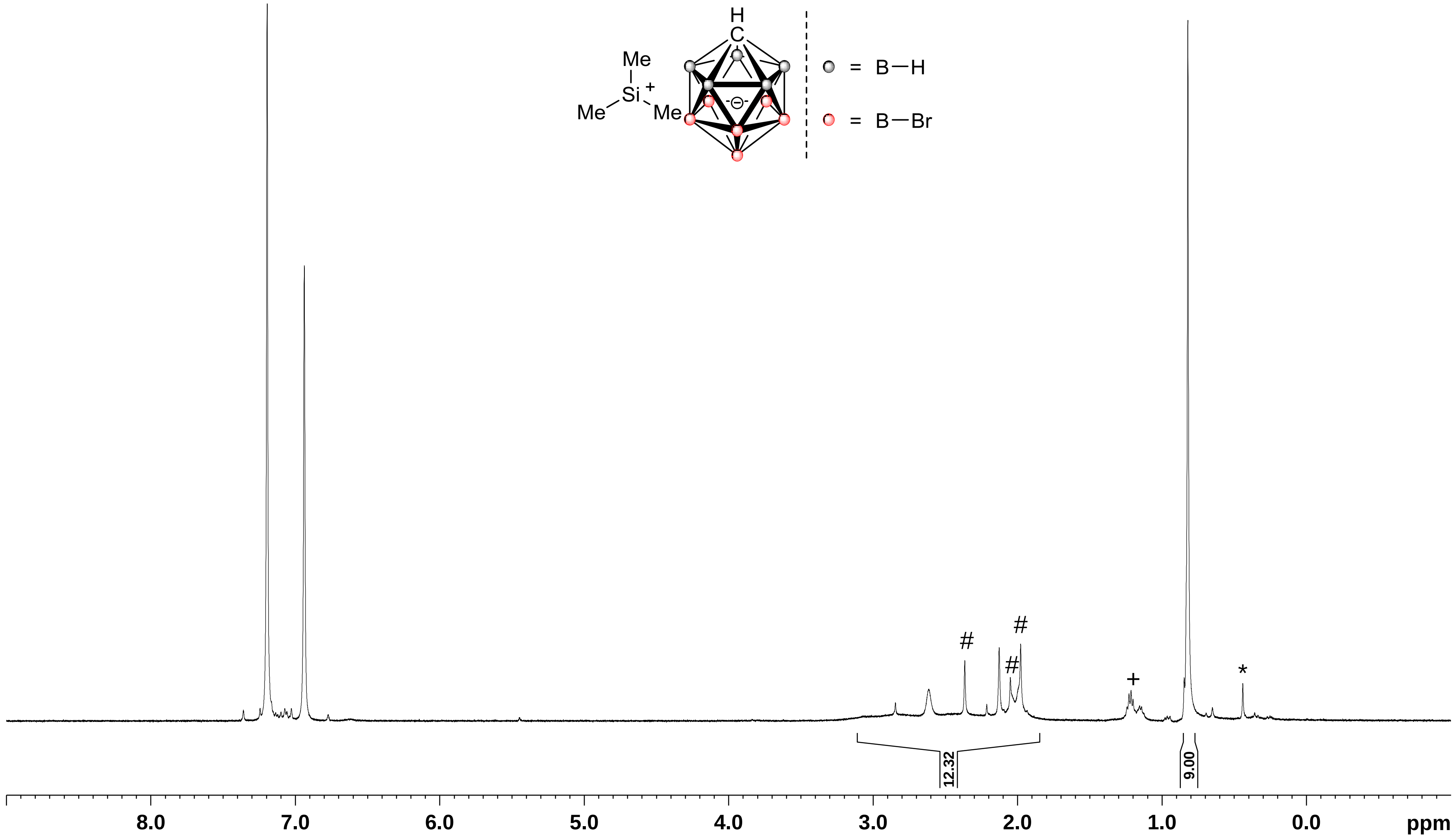

Figure S37. $^{1}H/^{29}Si$ HMQC NMR spectrum (500/99 MHz, *o*-$Cl_2C_6D_4$, 300 K, optimized for *J* = 7 Hz) of $Me_3Si^+[CHB_{11}H_5Br_6]^-$ from the reaction of $Me_2(C_6Me_5)SiH$ with $Ph_3C^+[CHB_{11}H_5Br_6]^-$.

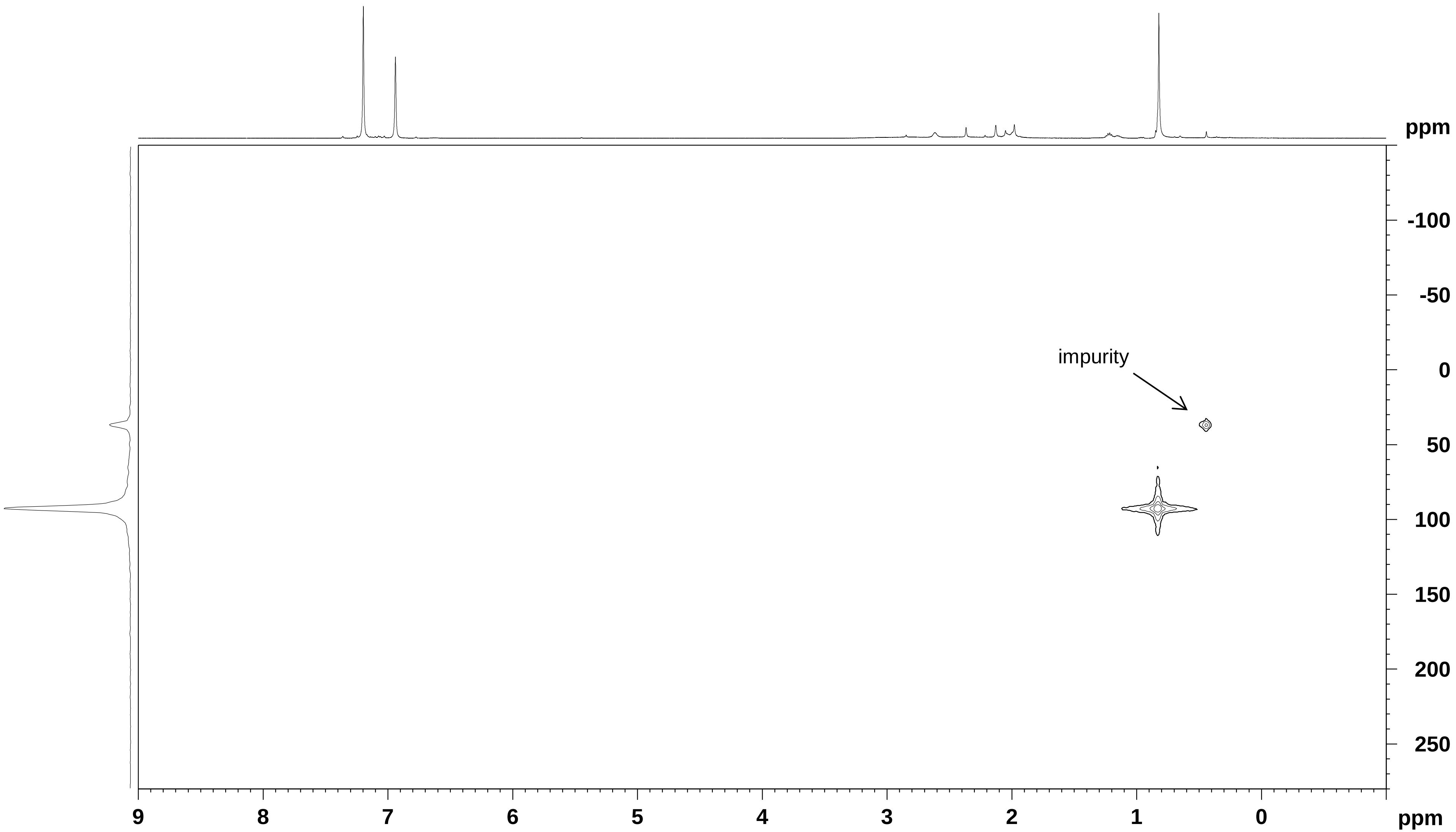

Figure S38. ^{1}H NMR spectrum (500 MHz, *o*-$Cl_2C_6D_4$, 298 K) of $Et_3Si^+[CHB_{11}H_5Br_6]^-$ from the reaction of Et_2PhSiH with $Ph_3C^+[CHB_{11}H_5Br_6]^-$ (# = impurity).

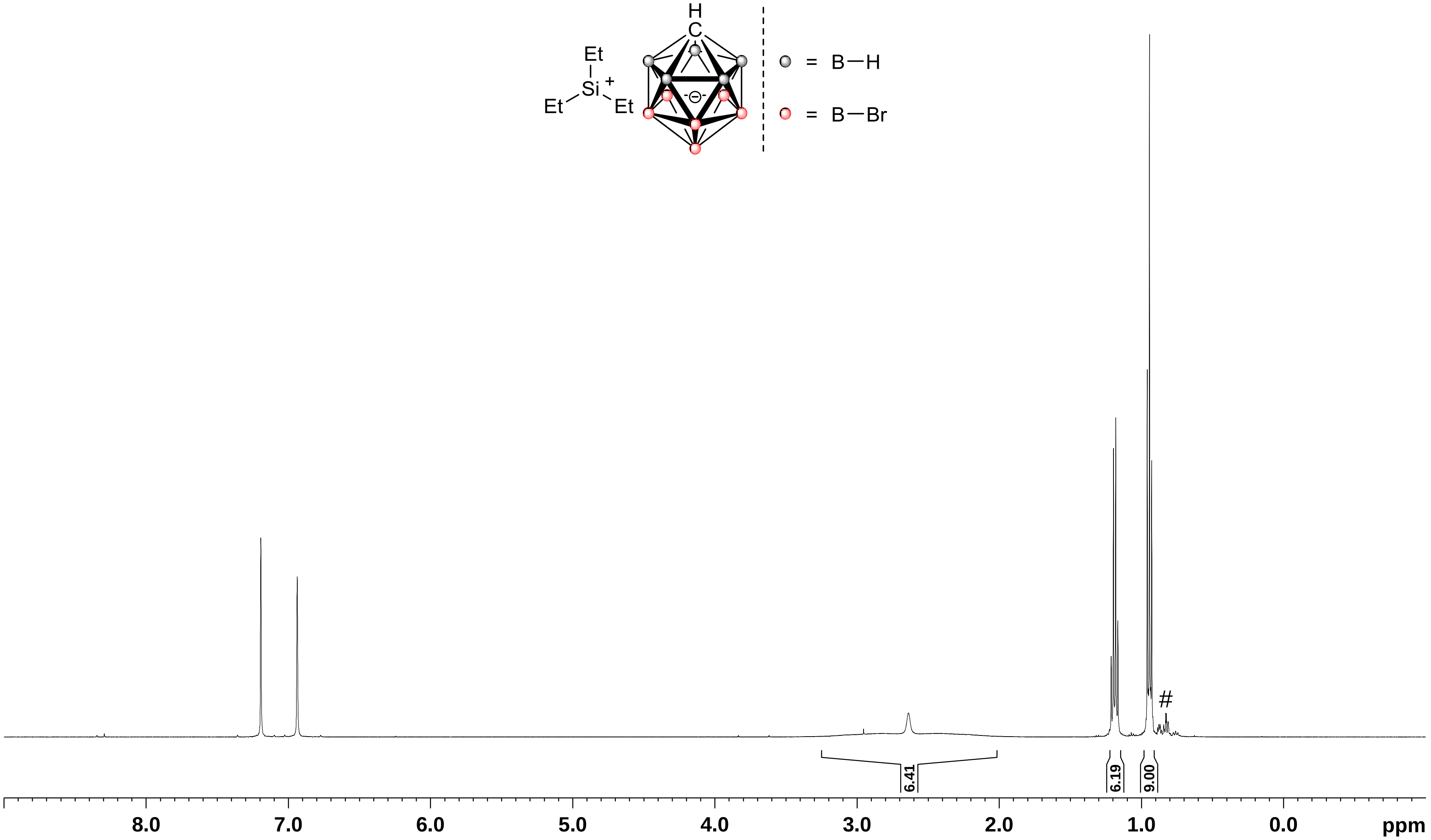

Figure S39. ^{11}B NMR spectrum (161 MHz, *o*-$Cl_2C_6D_4$, 298 K) of $Et_3Si^+[CHB_{11}H_5Br_6]^-$ from the reaction of Et_2PhSiH with $Ph_3C^+[CHB_{11}H_5Br_6]^-$.

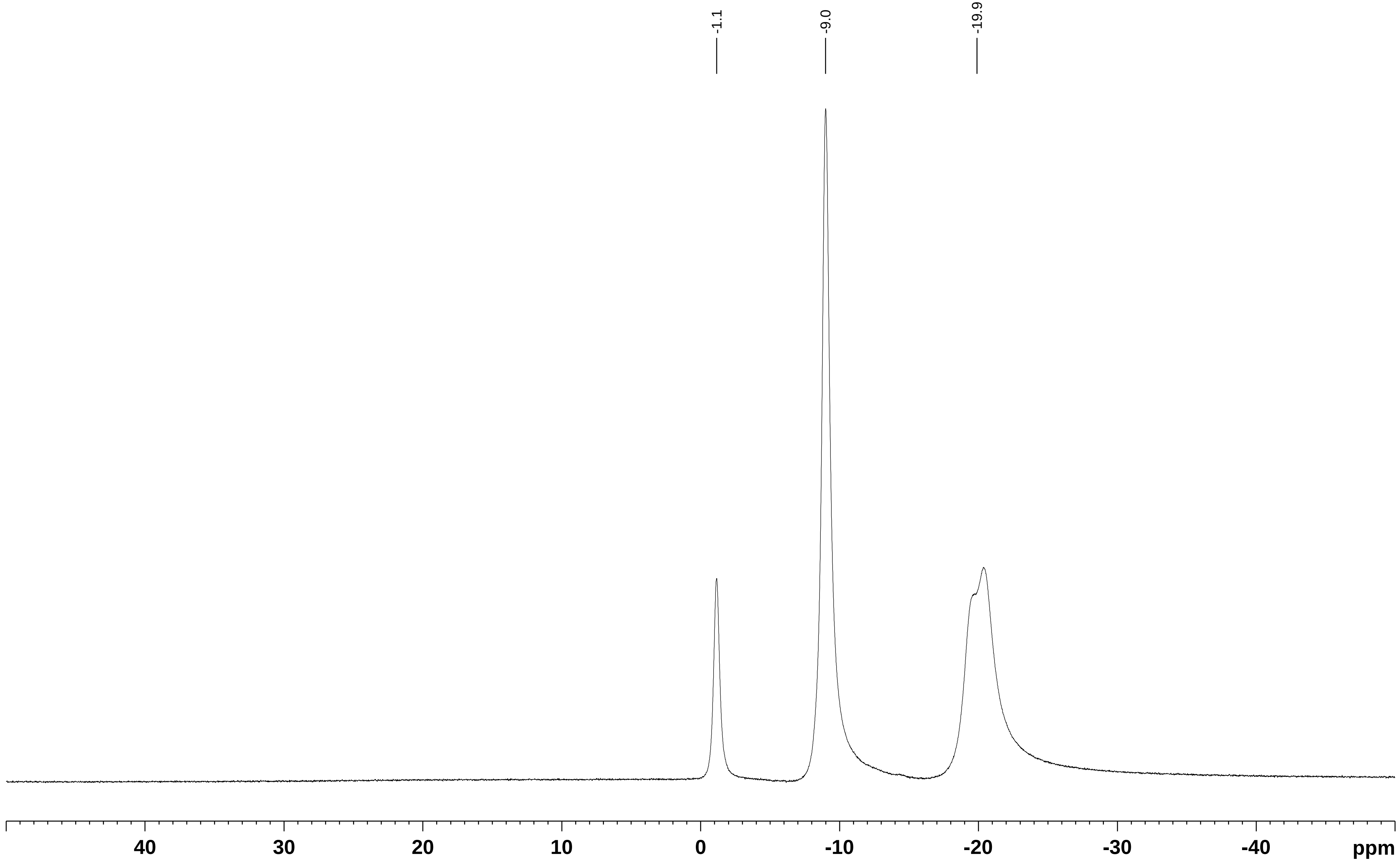

Figure S40. $^{13}C\{^{1}H\}$ DEPT NMR spectrum (126 MHz, *o*-$Cl_2C_6D_4$, 298 K) of $Et_3Si^{+}[CHB_{11}H_5Br_6]^{-}$ from the reaction of Et_2PhSiH with $Ph_3C^{+}[CHB_{11}H_5Br_6]^{-}$.

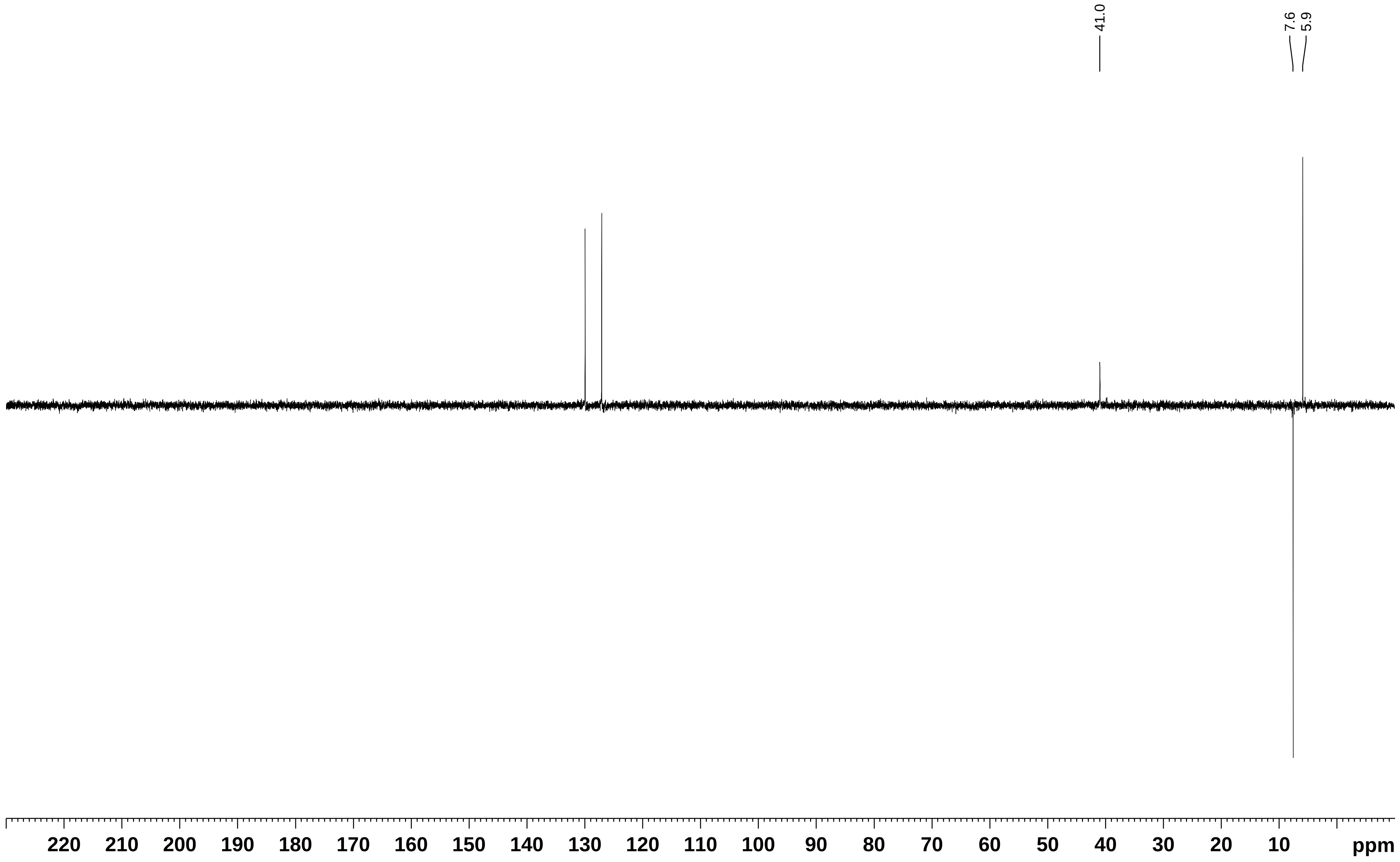

Figure S41. $^{1}H/^{29}Si$ HMQC NMR spectrum (500/99 MHz, *o*-$Cl_2C_6D_4$, 298 K, optimized for *J* = 7 Hz) of $Et_3Si^{+}[CHB_{11}H_5Br_6]^{-}$ from the reaction of Et_2PhSiH with $Ph_3C^{+}[CHB_{11}H_5Br_6]^{-}$.

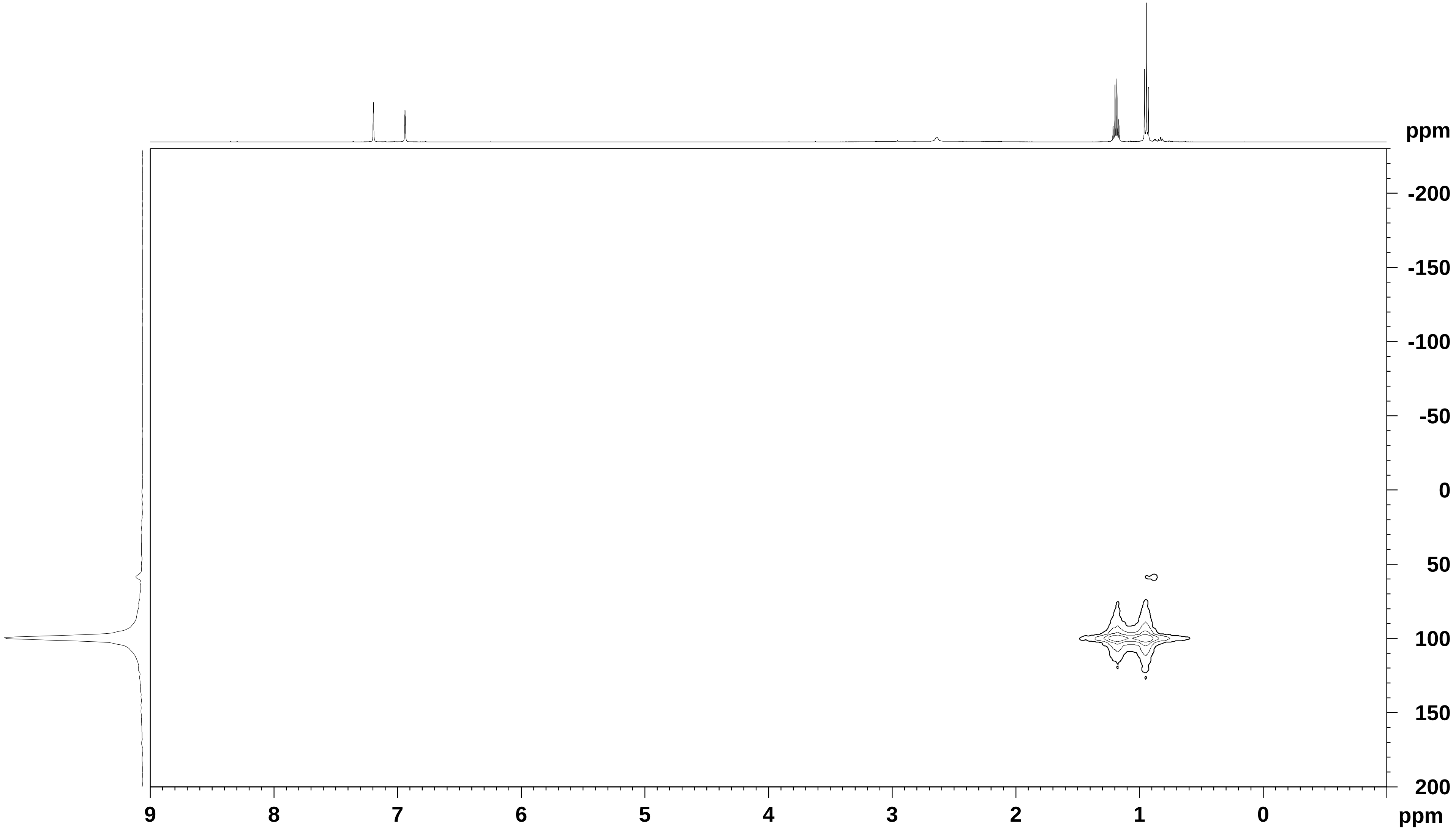

Figure S42. ^{1}H NMR spectrum (500 MHz, *o*-$Cl_2C_6D_4$, 298 K) of $Et_3Si^+[CHB_{11}H_5Br_6]^-$ with small amounts of $Et_2PhSi^+[CHB_{11}H_5Br_6]^-$ from the reaction of Et_2PhSiH with $Ph_3C^+[CHB_{11}H_5Br_6]^-$ (reaction performed with 2.0 equiv of Et_2PhSiH; stopped after 18 h reaction time; + = *n*-pentane).

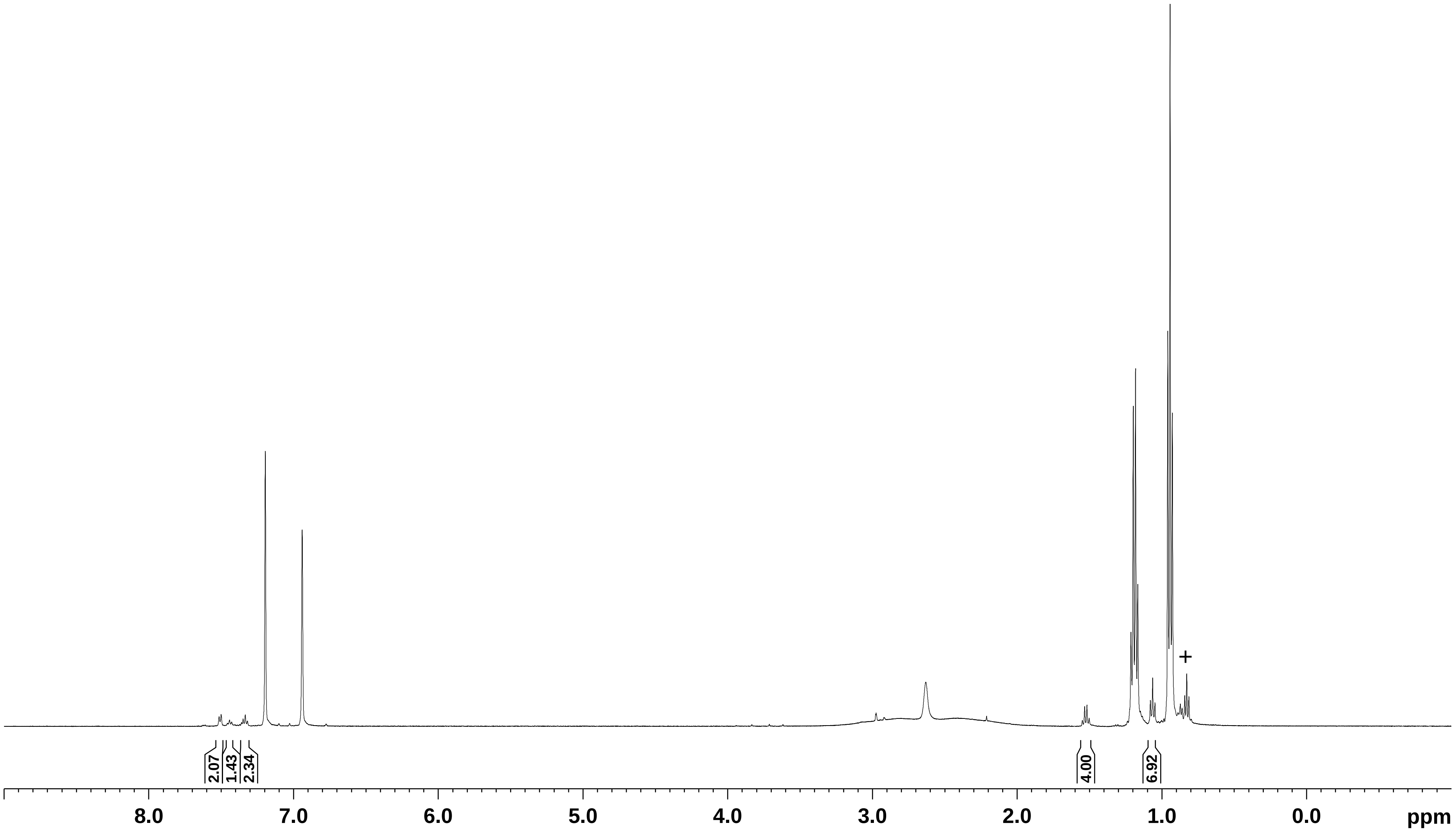

Figure S43. ^{1}H/^{29}Si HMQC NMR spectrum (500/99 MHz, *o*-$Cl_2C_6D_4$, 298 K, optimized for *J* = 7 Hz) of $Et_3Si^+[CHB_{11}H_5Br_6]^-$ with small amounts of $Et_2PhSi^+[CHB_{11}H_5Br_6]^-$ from the reaction of Et_2PhSiH with $Ph_3C^+[CHB_{11}H_5Br_6]^-$.

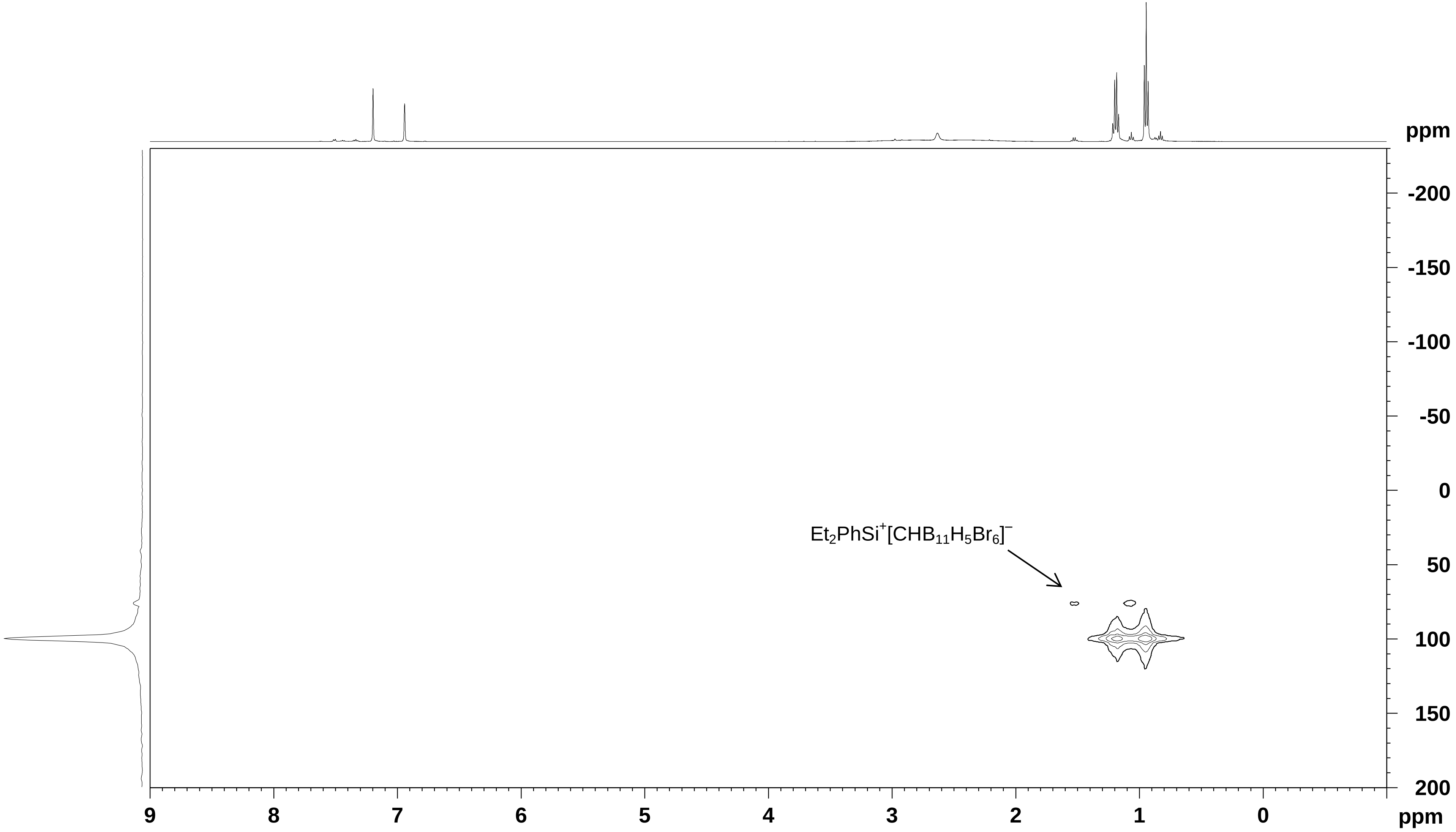

Figure S44. ^{1}H NMR spectrum (500 MHz, *o*-$Cl_2C_6D_4$, 298 K) of $Et_3Si^+[CHB_{11}H_5Br_6]^-$ from the reaction of Et_3SiH with $Ph_3C^+[CHB_{11}H_5Br_6]^-$ (+ = *n*-pentane, # = impurity).

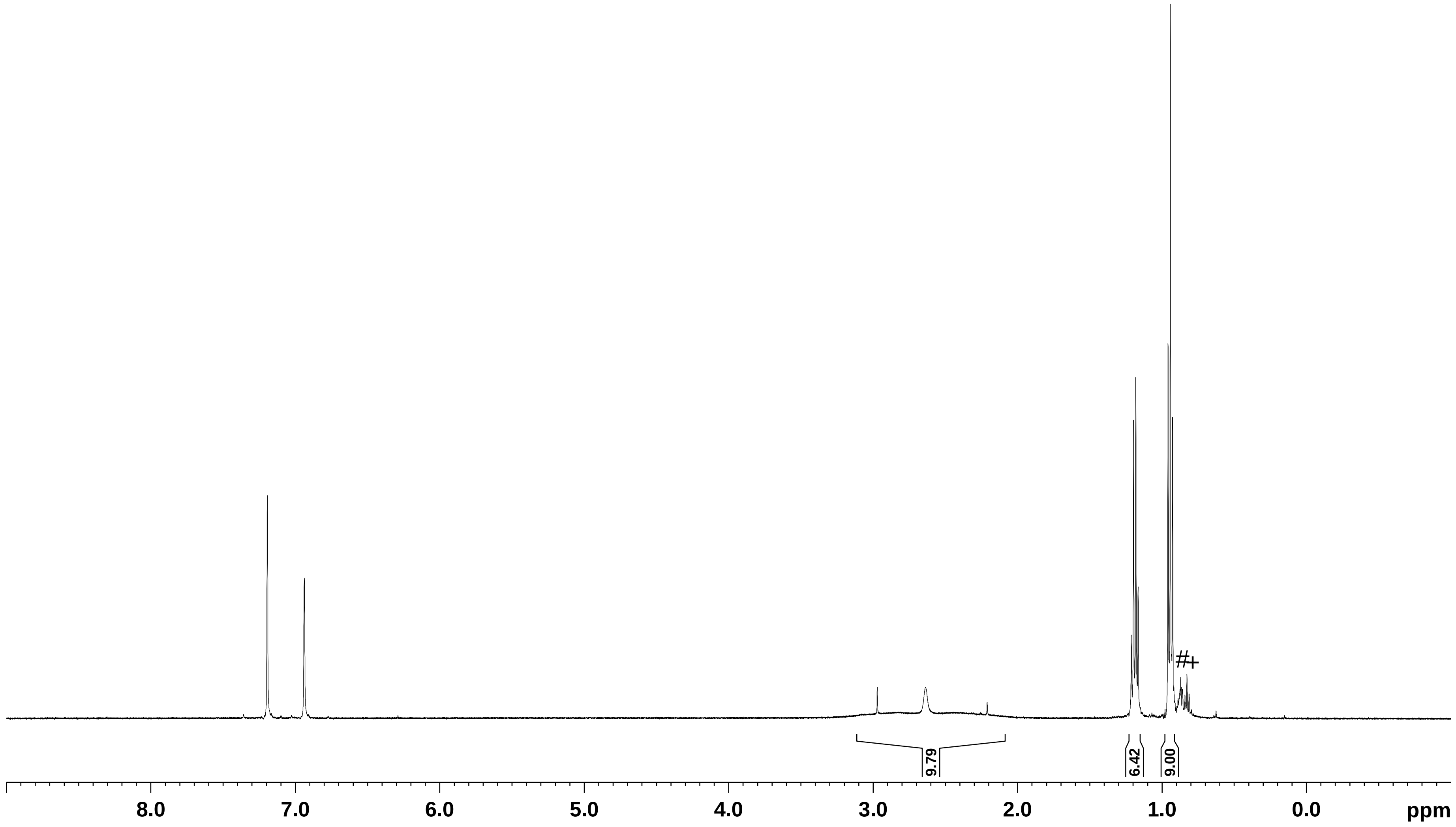

Figure S45. $^{1}H/^{29}Si$ HMQC NMR spectrum (500/99 MHz, *o*-$Cl_2C_6D_4$, 298 K, optimized for J = 7 Hz) of $Et_3Si^+[CHB_{11}H_5Br_6]^-$ from the reaction of Et_3SiH with $Ph_3C^+[CHB_{11}H_5Br_6]^-$.

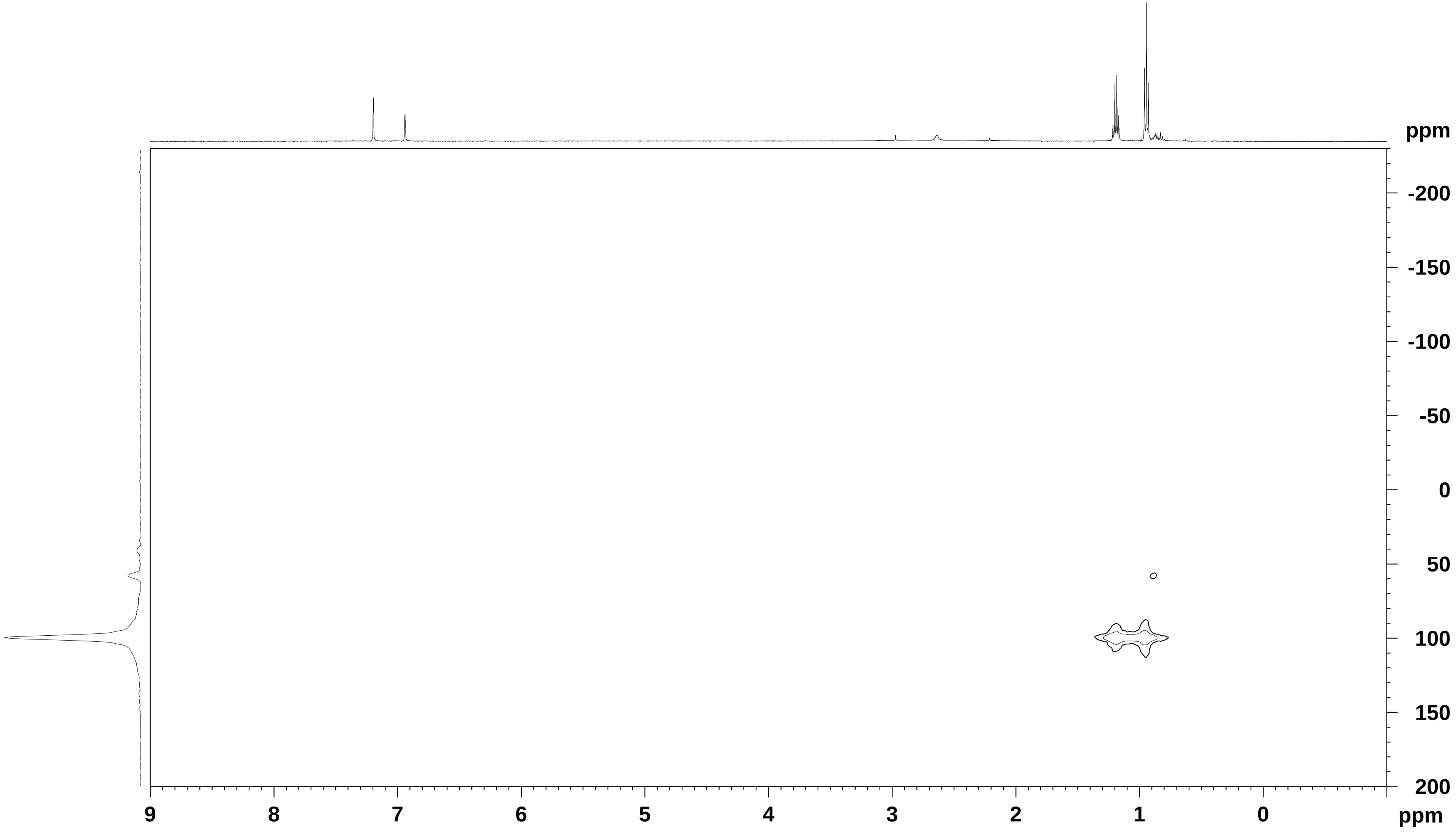

Figure S46. ^{1}H NMR spectrum (500 MHz, *o*-$Cl_2C_6D_4$, 298 K) of *i*$Pr_2PhSi^+[CHB_{11}H_5Br_6]^-$ from the reaction of *i*Pr_2PhSiH with $Ph_3C^+[CHB_{11}H_5Br_6]^-$ (+ = *n*-pentane).

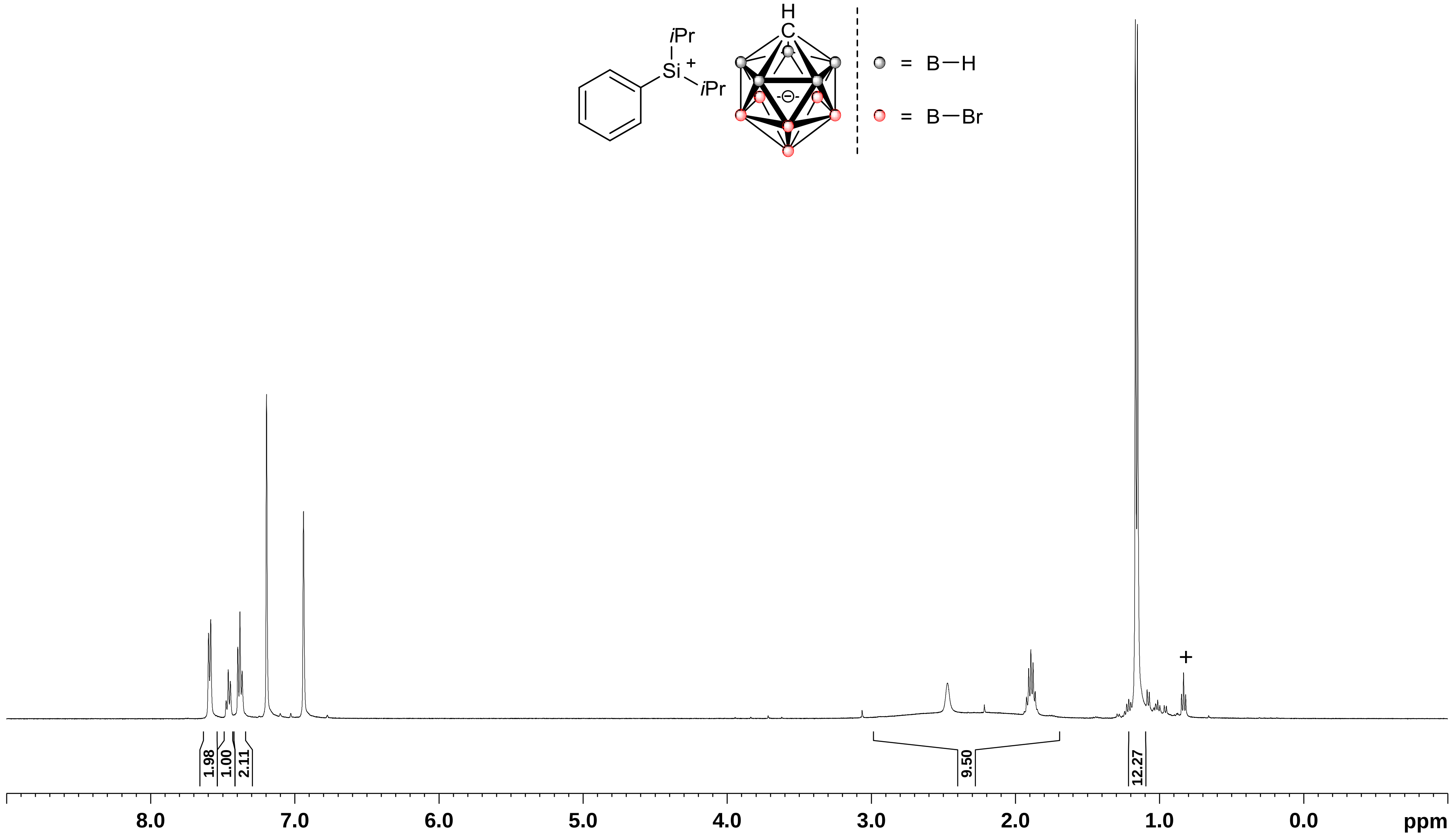

Figure S47. ^{11}B NMR spectrum (161 MHz, *o*-$Cl_2C_6D_4$, 298 K) of *i*$Pr_2PhSi^+[CHB_{11}H_5Br_6]^-$ from the reaction of *i*Pr_2PhSiH with $Ph_3C^+[CHB_{11}H_5Br_6]^-$.

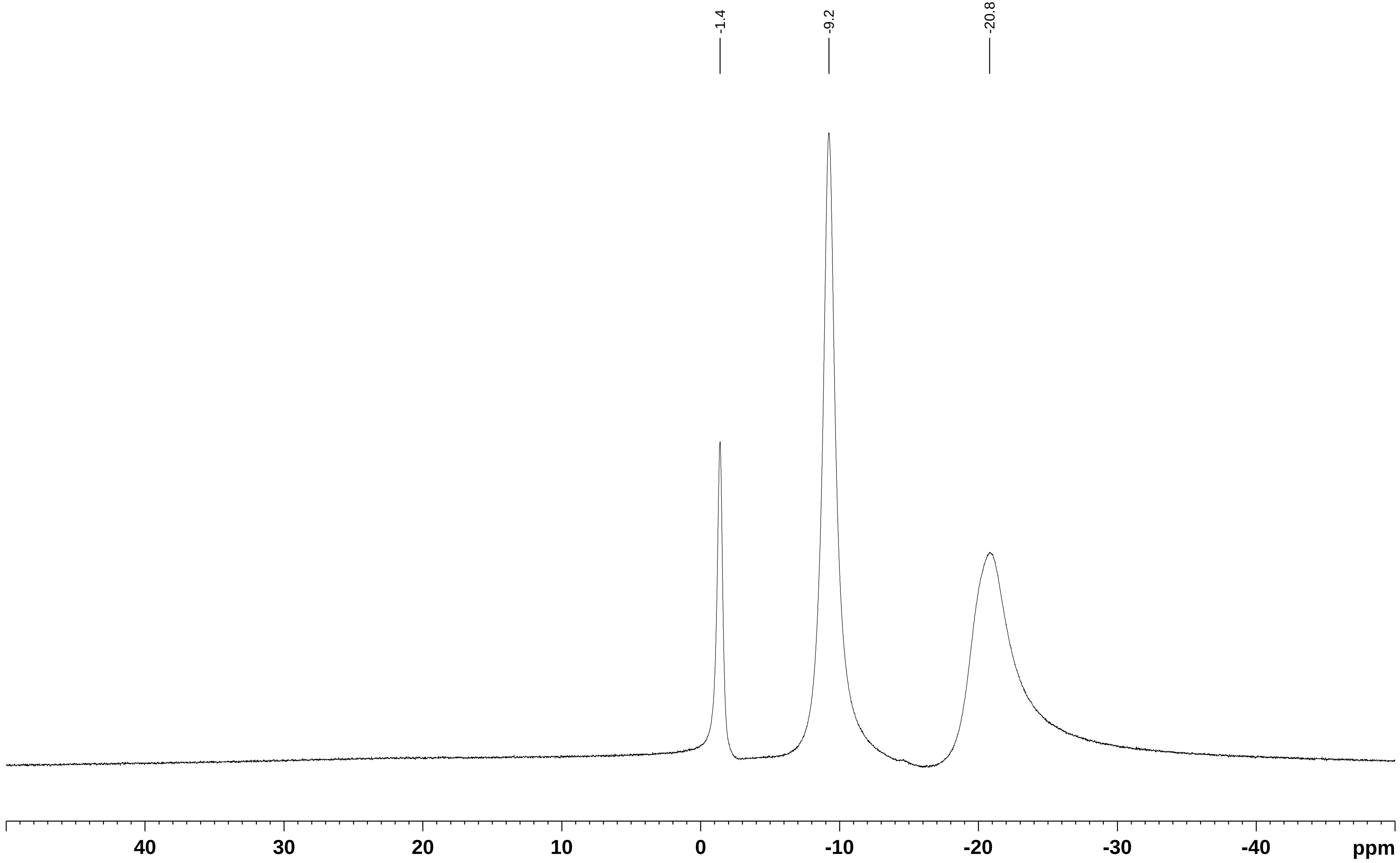

Figure S48. $^{13}C\{^{1}H\}$ NMR spectrum (126 MHz, *o*-$Cl_2C_6D_4$, 298 K) of *i*$Pr_2PhSi^+[CHB_{11}H_5Br_6]^-$ from the reaction of *i*Pr_2PhSiH with $Ph_3C^+[CHB_{11}H_5Br_6]^-$ (+ = *n*-pentane).

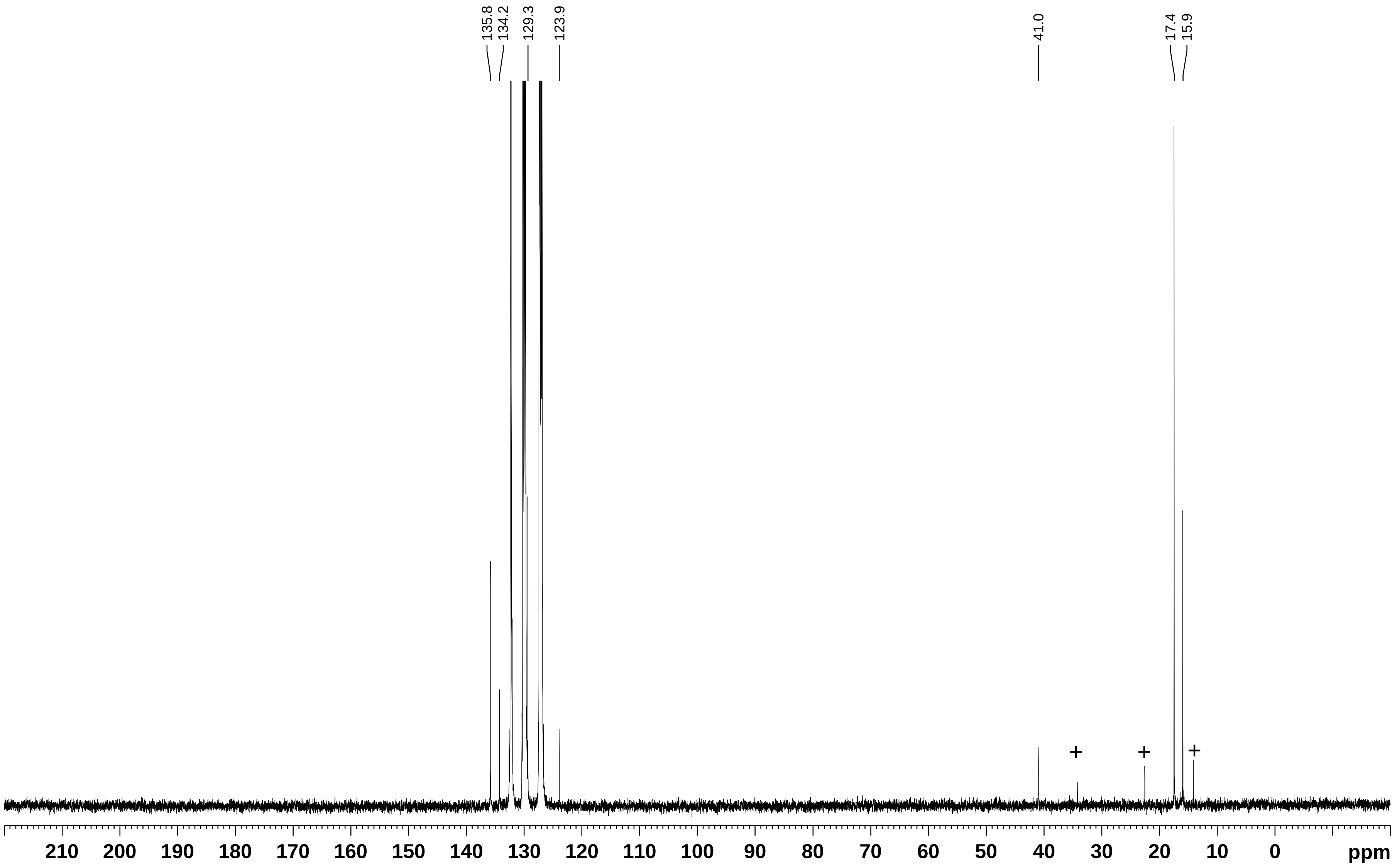

Figure S49. $^{1}H/^{29}Si$ HMQC NMR spectrum (500/99 MHz, *o*-$Cl_2C_6D_4$, 298 K, optimized for J = 7 Hz) of *i*$Pr_2PhSi^+[CHB_{11}H_5Br_6]^-$ from the reaction of *i*Pr_2PhSiH with $Ph_3C^+[CHB_{11}H_5Br_6]^-$.

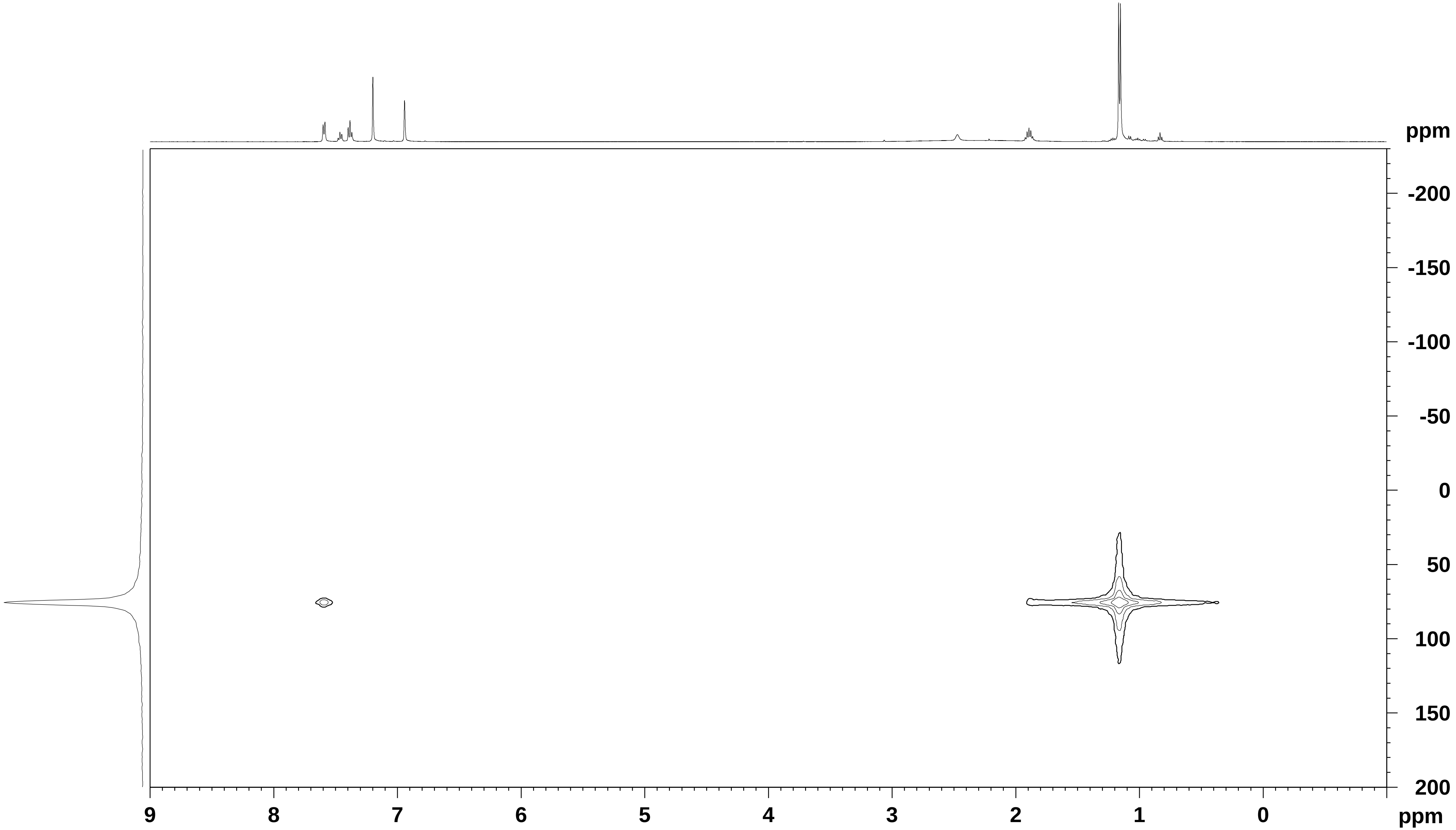

Figure S50. ^{1}H NMR spectrum (500 MHz, *o*-$Cl_2C_6D_4$, 300 K) of $Me_3Si^+[CHB_{11}H_5Br_6]^-$ from the reaction of Me_2BnSiH with $Ph_3C^+[CHB_{11}H_5Br_6]^-$ (+ = *n*-pentane, # = impurity).

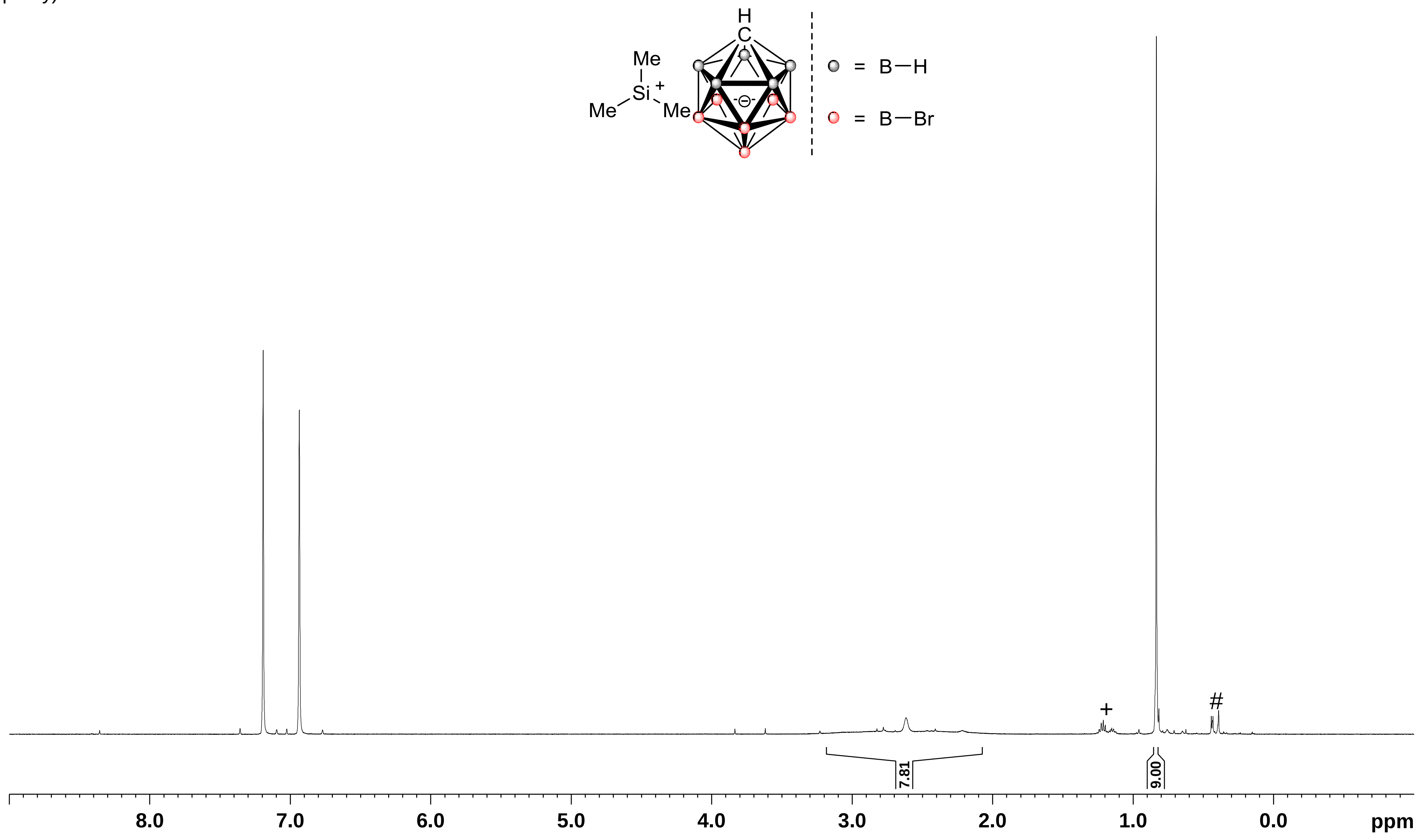

Figure S51. $^{1}H/^{29}Si$ HMQC NMR spectrum (500/99 MHz, *o*-$Cl_2C_6D_4$, 300 K, optimized for *J* = 7 Hz) of $Me_3Si^+[CHB_{11}H_5Br_6]^-$ from the reaction of Me_2BnSiH with $Ph_3C^+[CHB_{11}H_5Br_6]^-$.

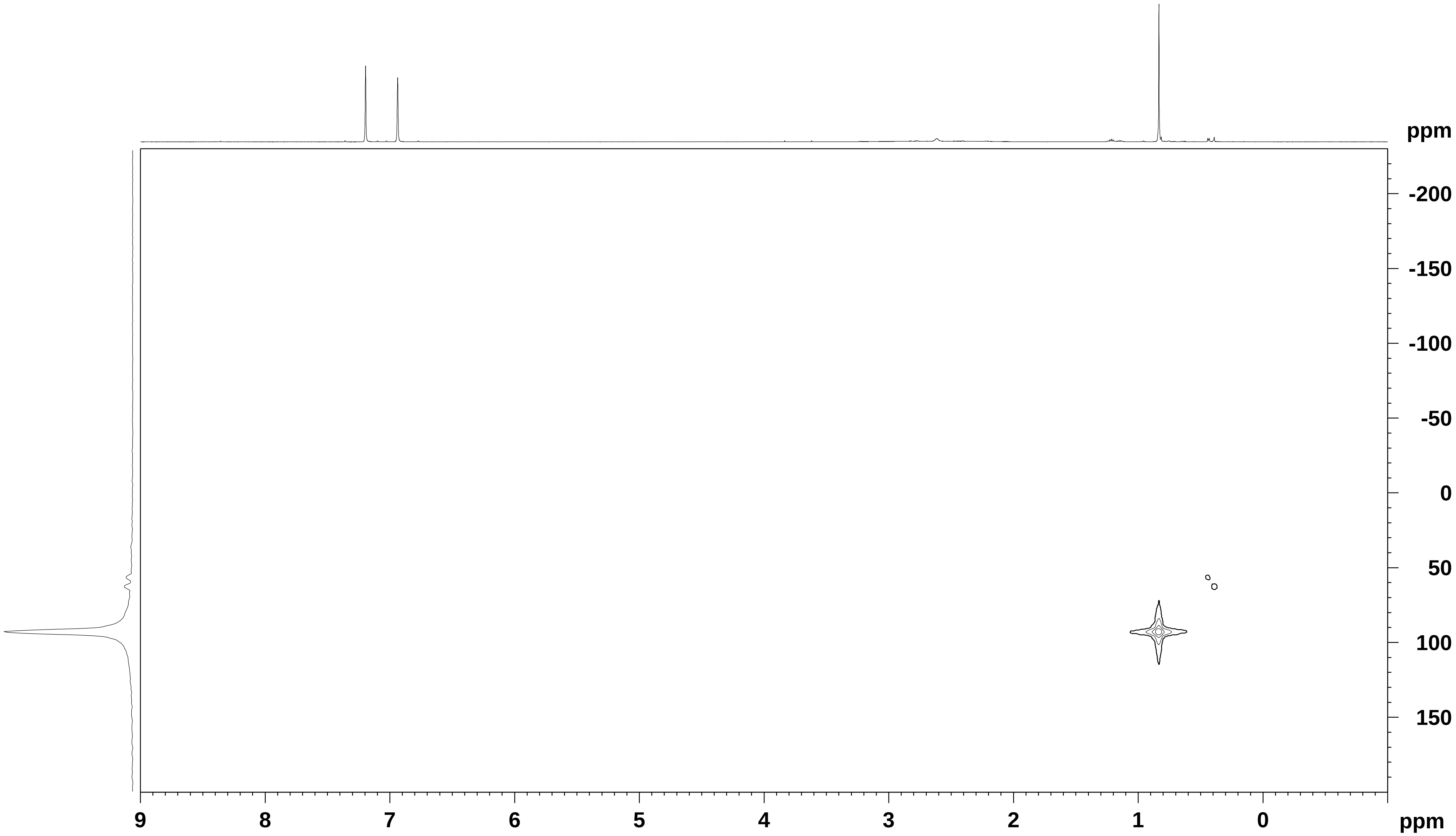

Figure S52. ^{1}H NMR spectrum (500 MHz, *o*-$Cl_2C_6D_4$, 300 K) of $Me_2tBuSi^+[CHB_{11}H_5Br_6]^-$ from the reaction of $Me_2tBuSiH$ with $Ph_3C^+[CHB_{11}H_5Br_6]^-$.

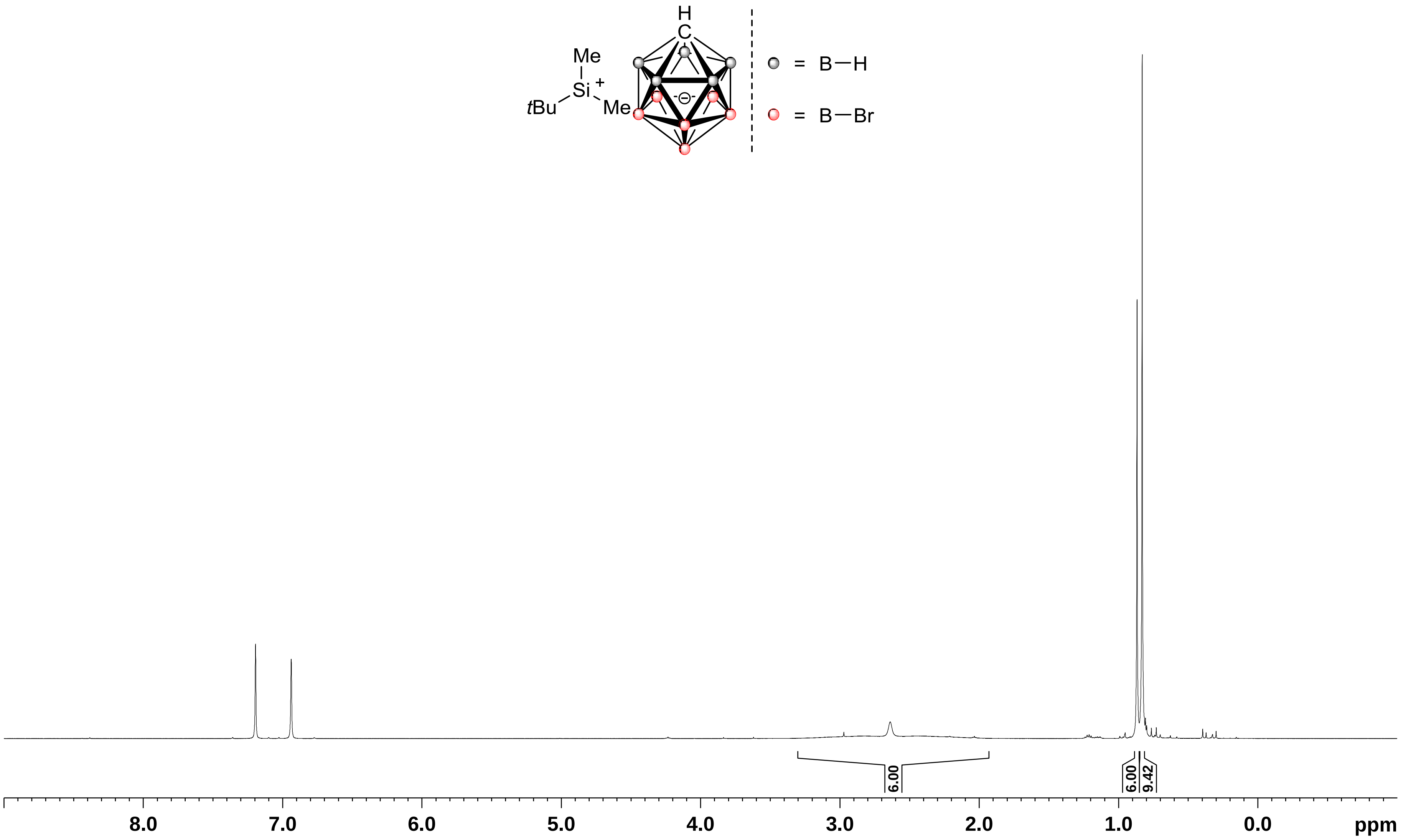

Figure S53. ^{11}B NMR spectrum (161 MHz, *o*-$Cl_2C_6D_4$, 300 K) of $Me_2tBuSi^+[CHB_{11}H_5Br_6]^-$ from the reaction of $Me_2tBuSiH$ with $Ph_3C^+[CHB_{11}H_5Br_6]^-$.

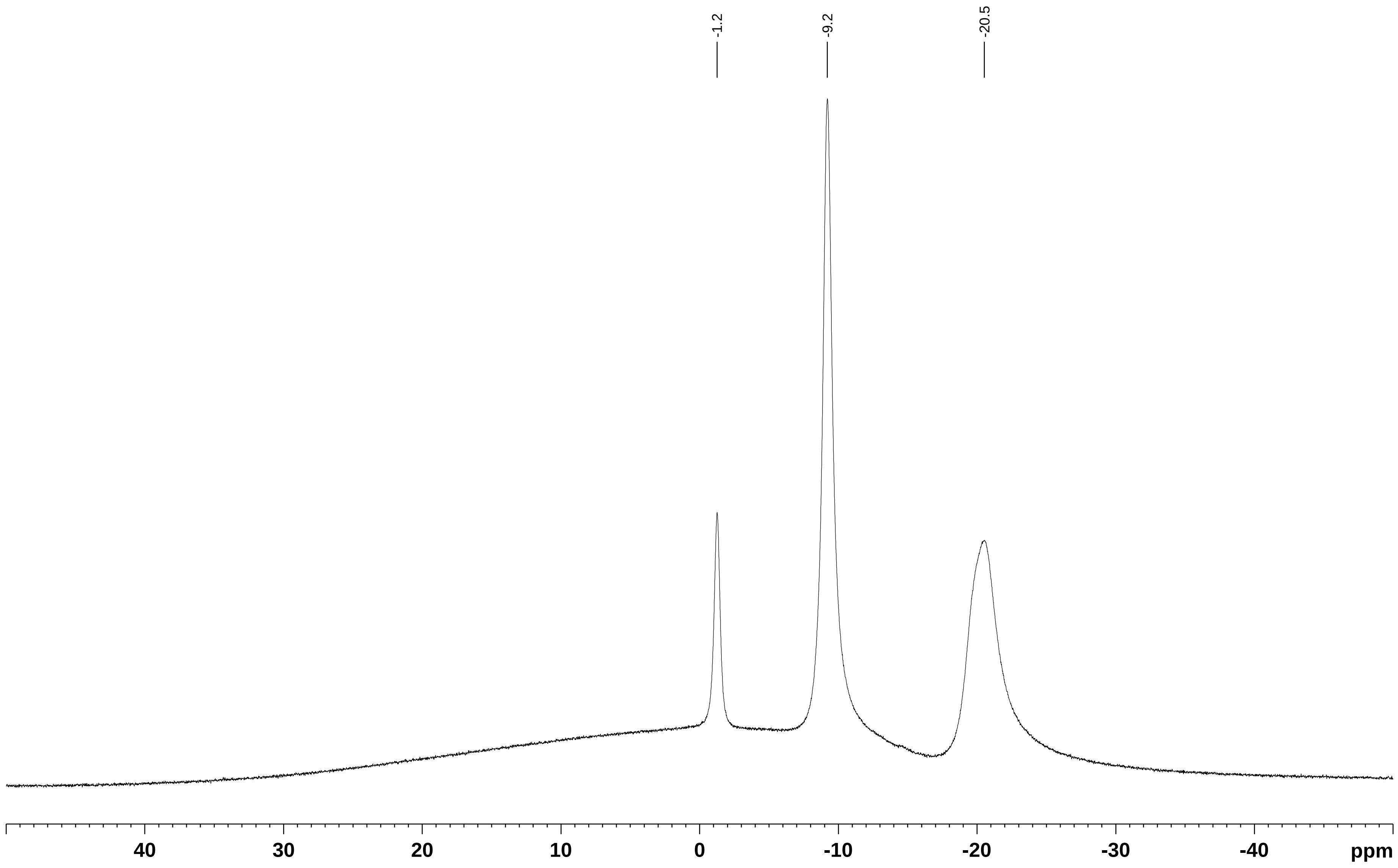

Figure S54. $^{13}C\{^1H\}$ NMR spectrum (126 MHz, *o*-$Cl_2C_6D_4$, 300 K) of $Me_2tBuSi^+[CHB_{11}H_5Br_6]^-$ from the reaction of $Me_2tBuSiH$ with $Ph_3C^+[CHB_{11}H_5Br_6]^-$.

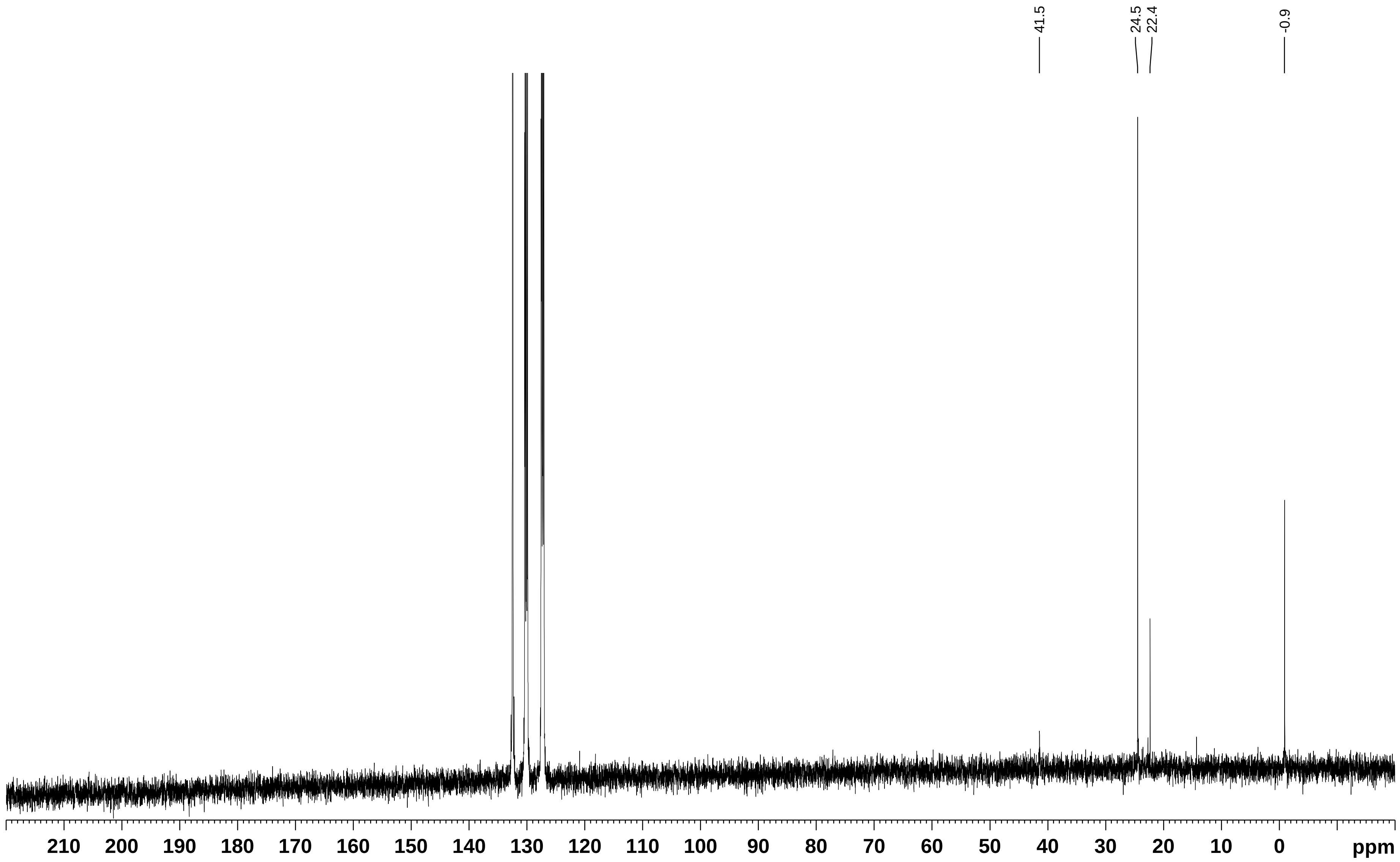

Figure S55. $^{1}H/^{29}Si$ HMQC NMR spectrum (500/99 MHz, *o*-$Cl_2C_6D_4$, 300 K, optimized for J = 7 Hz) of $Me_2tBuSi^+[CHB_{11}H_5Br_6]^-$ from the reaction of $Me_2tBuSiH$ with $Ph_3C^+[CHB_{11}H_5Br_6]^-$.

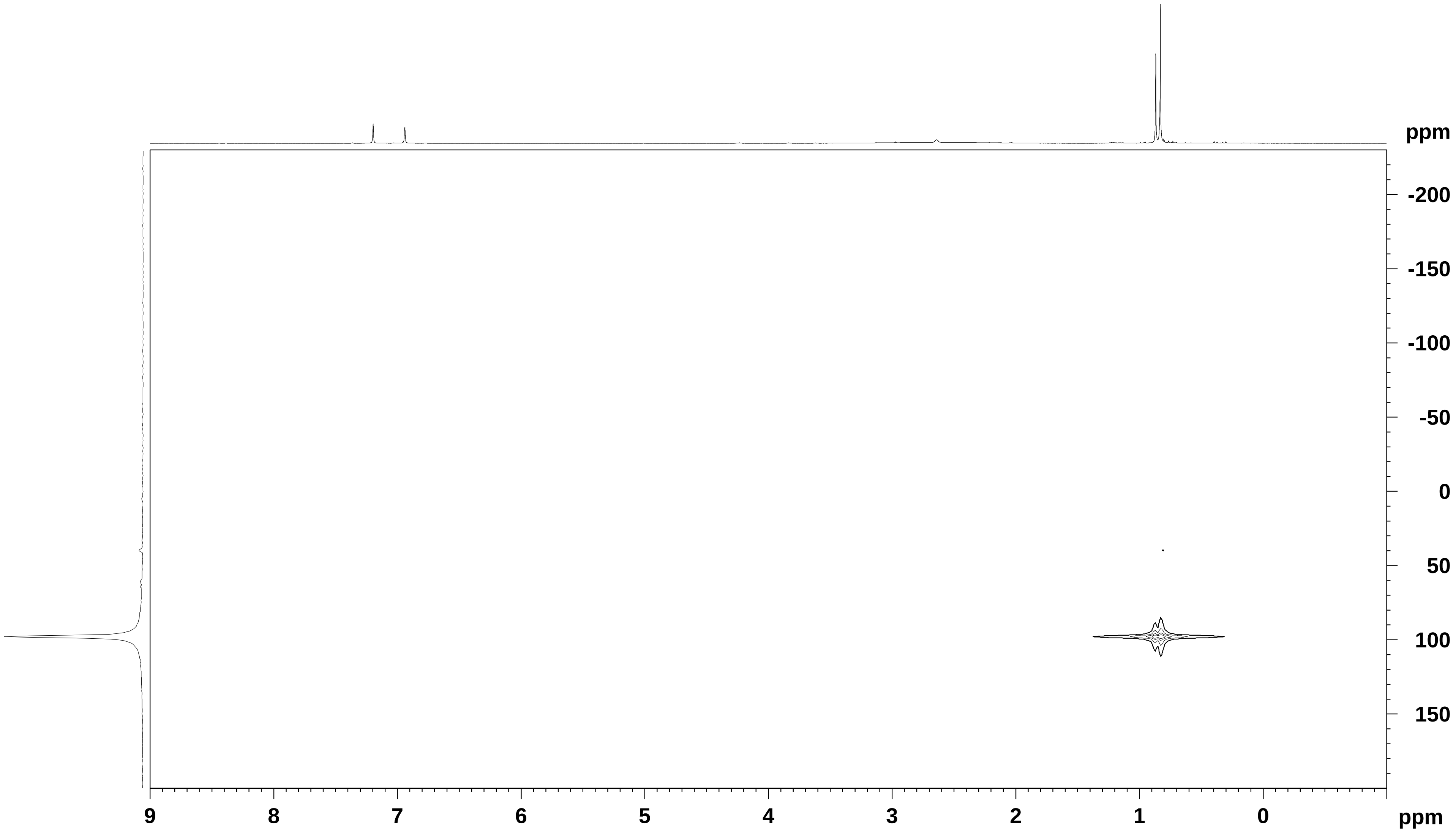

Figure S56. ^{1}H NMR spectrum (700 MHz, *o*-$Cl_2C_6D_4$, 298 K) of $Me_3Si^+[CHB_{11}H_5Br_6]^-$ with small amounts of $Me_2PhSi^+[CHB_{11}H_5Br_6]^-$ from the reaction of Me_2PhSiH with $Ph_3C^+[CHB_{11}H_5Br_6]^-$ (stopped after 10 min reaction time; * = toluene, # = $Me_2PhSi^+[CHB_{11}H_5Br_6]^-$).

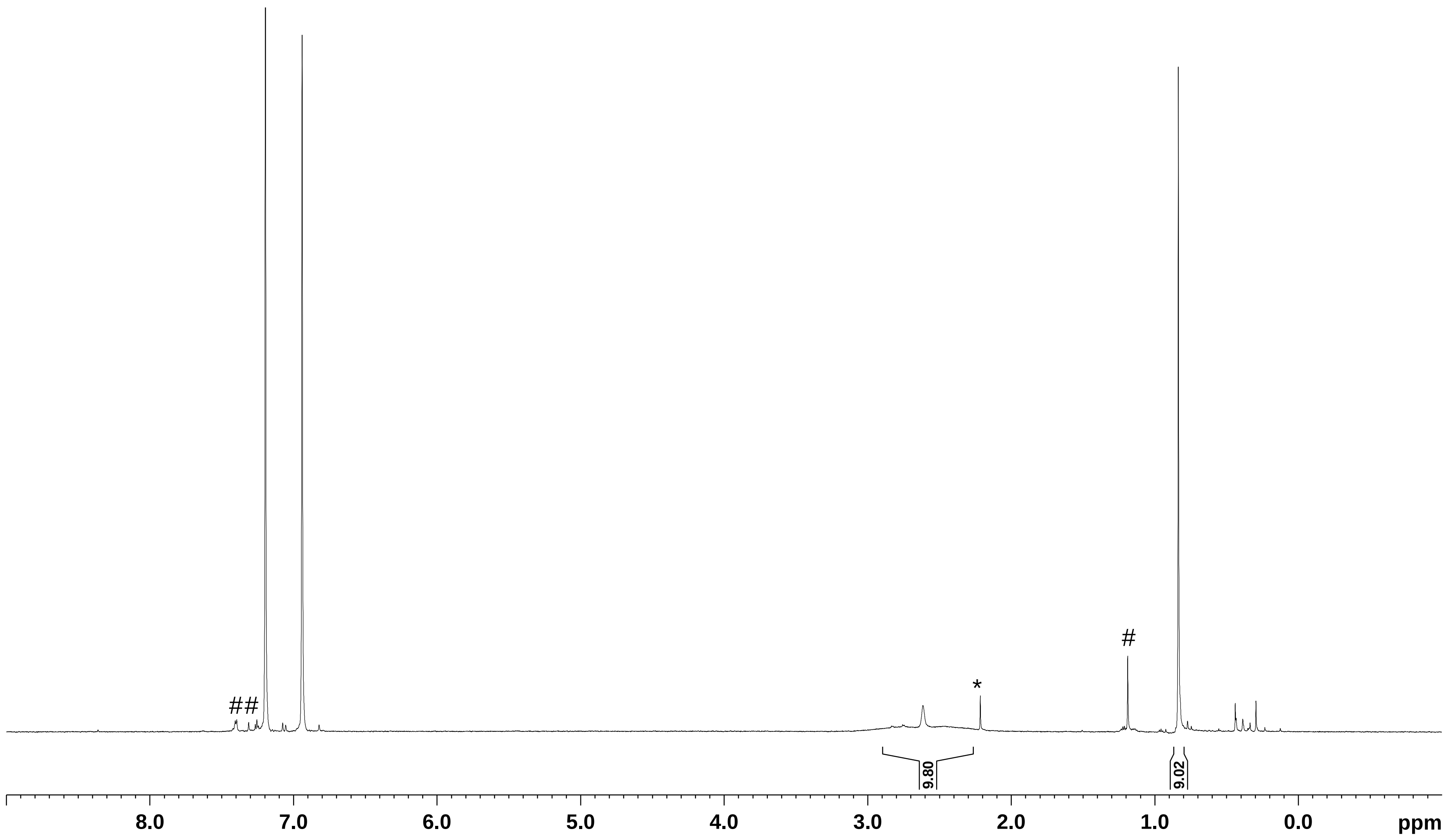

Figure S57. $^{1}H/^{29}Si$ HMQC NMR spectrum (700/139 MHz, *o*-$Cl_2C_6D_4$, 298 K, optimized for *J* = 7 Hz) of $Me_3Si^+[CHB_{11}H_5Br_6]^-$ with small amounts of $Me_2PhSi^+[CHB_{11}H_5Br_6]^-$ from the reaction of Me_2PhSiH with $Ph_3C^+[CHB_{11}H_5Br_6]^-$ (stopped after 10 min reaction time).

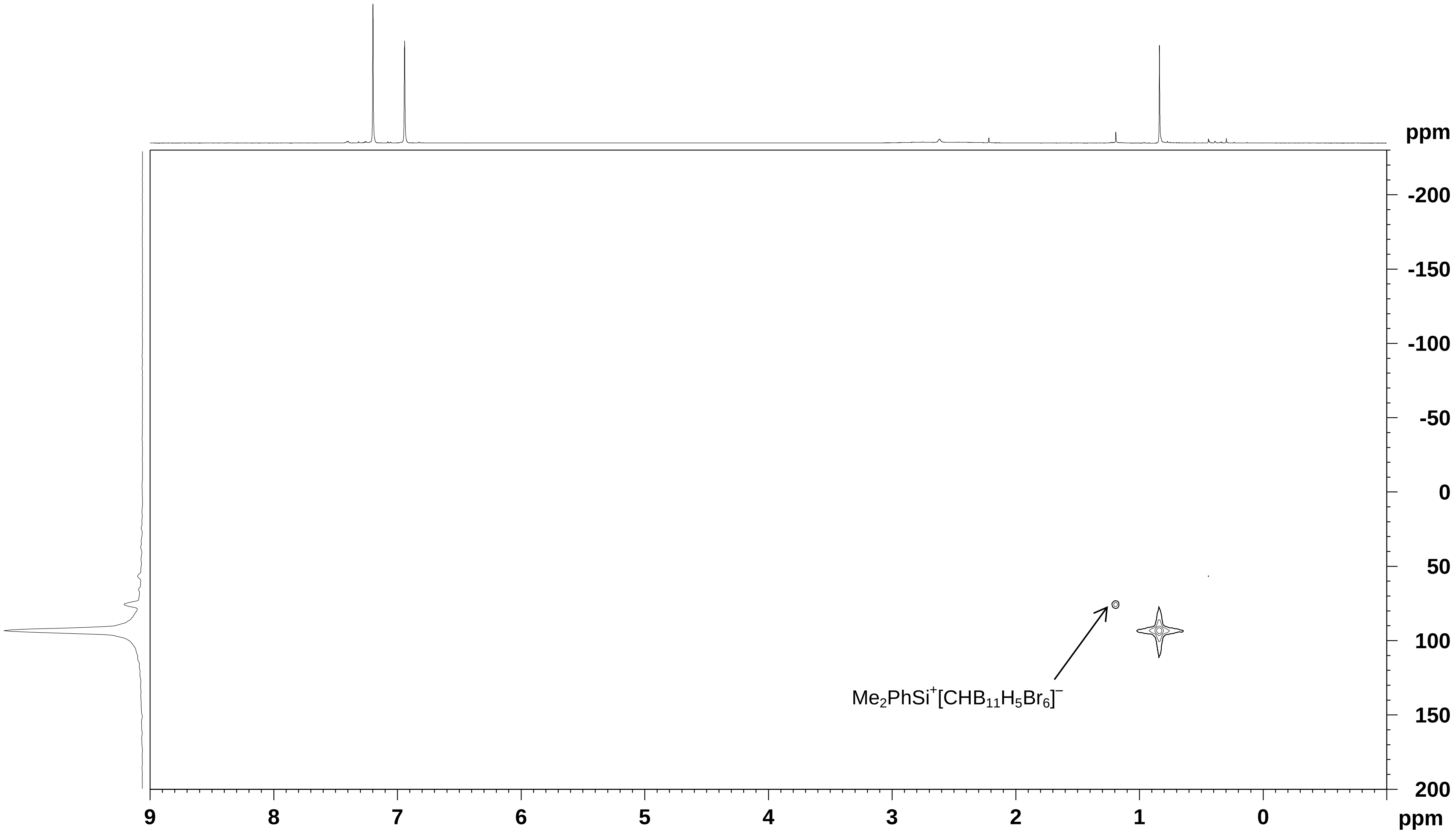

Figure S58. ^{1}H NMR spectrum (500 MHz, *o*-$Cl_2C_6D_4$, 298 K) of $Me_3Si^{+}[B(C_6F_5)_4]^{-}$ and $Me_2PhSi^{+}[B(C_6F_5)_4]^{-}$ from the reaction of Me_2PhSiH with $Ph_3C^{+}[B(C_6F_5)_4]^{-}$ (stopped after 10 min reaction time; * = toluene-d8).

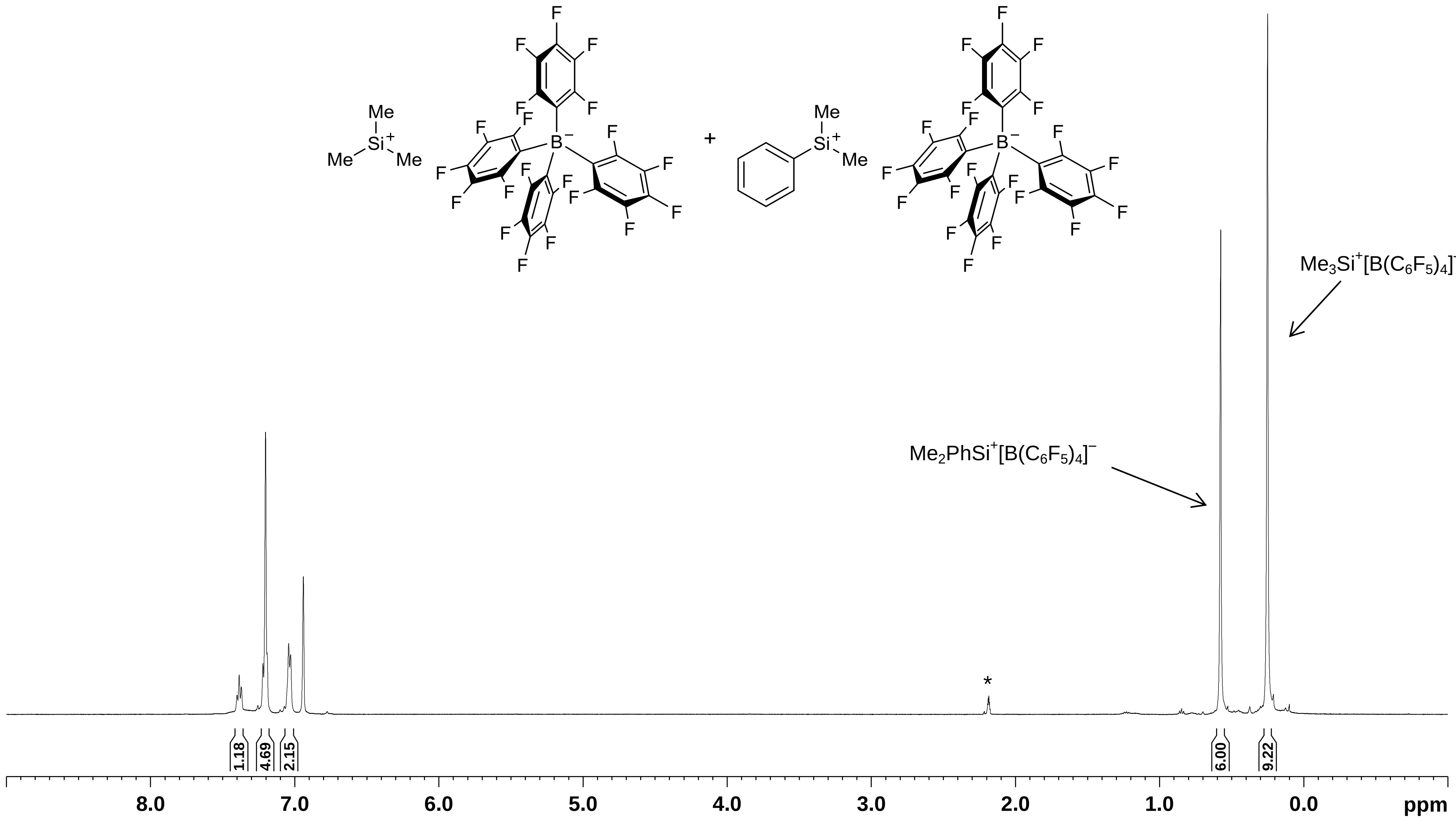

Figure S59. $^{1}H/^{29}Si$ HMQC NMR spectrum (500/99 MHz, *o*-$Cl_2C_6D_4$, 298 K, optimized for J = 7 Hz) of $Me_3Si^+[B(C_6F_5)_4]^-$ and $Me_2PhSi^+[B(C_6F_5)_4]^-$ from the reaction of Me_2PhSiH with $Ph_3C^+[B(C_6F_5)_4]^-$ (stopped after 10 min reaction time).

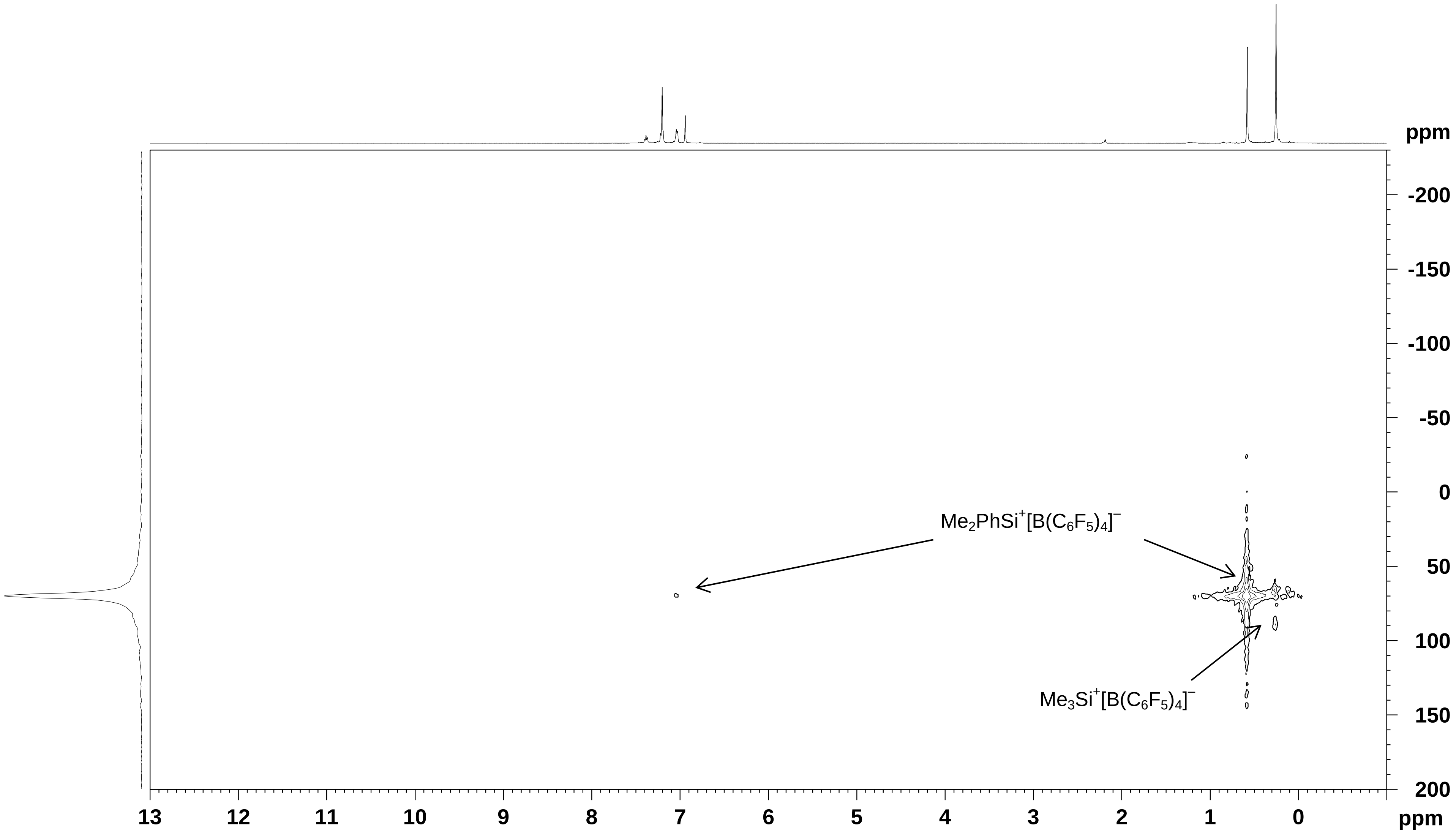

8 Crystallographic Data

Data for the single-crystal structure determination were collected with an *Agilent* SuperNova diffractometer equipped with a CCD area Atlas detector and a mirror monochromator by utilizing Cu-K_α radiation (λ= 1.5418 Å). Software packages used: CrysAlis PRO for data collection, cell refinement, and data reduction,[S10] SHELXS-97 for structure solution,[S11] SHELXL-97 for structure refinement,[S12] and Mercury[S13] for graphics.

8.1 Molecular Structure of $MePh_2Si^+[CHB_{11}H_5Br_6]^-$ (CCDC-1818582)

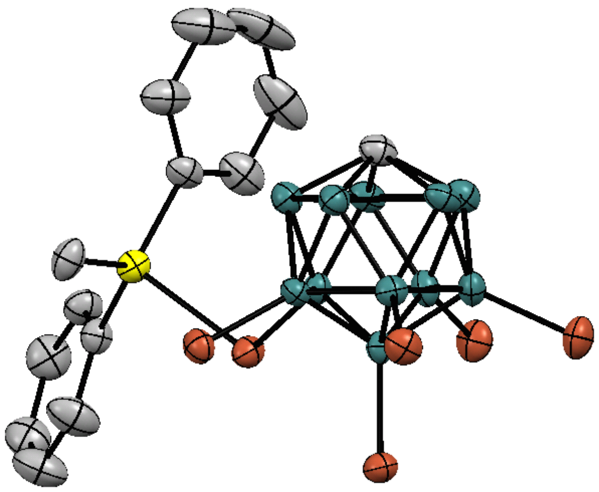

Figure S60. Molecular Structure of $MePh_2Si^+[CHB_{11}H_5Br_6]^-$ (thermal ellipsoids at 50% probability level; H atoms omitted for clarity).

Empirical formula	C14 H19 B11 Br6 Si
Formula weight	813.75
Temperature	150.00(10) K
Wavelength	1.54184 Å
Crystal system	Orthorhombic
Space group	Pbca
Unit cell dimensions	a = 18.5845(4) Å
	b = 14.7047(2) Å
	c = 19.6730(3) Å
	α = 90°
	β = 90°
	γ = 90°
Volume	5376.25(17) $Å^3$
Z	8
Density (calculated)	2.011 Mg/m^3
Absorption coefficient	11.234 mm^{-1}
F(000)	3056
Crystal size	0.32 x 0.17 x 0.12 mm^3
Theta range for data collection	4.44 to 67.48°.
Index ranges	-22<=h<=17, -15<=k<=17, -23<=l<=23
Reflections collected	36966
Independent reflections	4839 [R(int) = 0.0863]
Completeness to theta = 67.48°	100.0 %
Absorption correction	Semi-empirical from equivalents
Max. and min. transmission	0.3458 and 0.1231
Refinement method	Full-matrix least-squares on F^2
Data / restraints / parameters	4839 / 0 / 313
Goodness-of-fit on F^2	1.084
Final R indices [I>2sigma(I)]	R1 = 0.0368, wR2 = 0.0808
R indices (all data)	R1 = 0.0519, wR2 = 0.0881
Largest diff. peak and hole	1.670 and -0.891 e.$Å^{-3}$

8.2 Molecular Structure of $Me_3Si^+[CHB_{11}H_5Br_6]^-$ (CCDC-1818576)

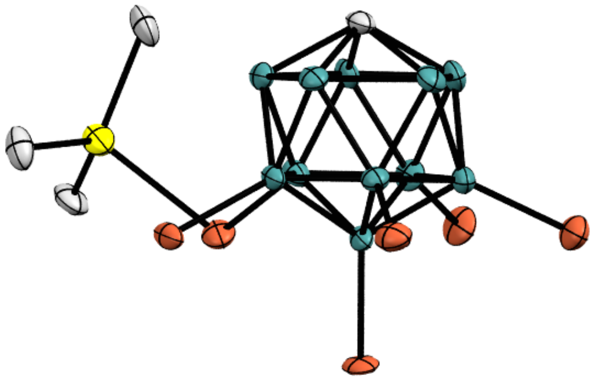

Figure S61. Molecular Structure of $Me_3Si^+[CHB_{11}H_5Br_6]^-$ (thermal ellipsoids at 50% probability level; H atoms omitted for clarity).

Empirical formula	C4 H15 B11 Br6 Si
Formula weight	689.62
Temperature	150.00(10) K
Wavelength	1.54184 Å
Crystal system	Triclinic
Space group	P-1
Unit cell dimensions	a = 7.6280(5) Å
	b = 10.6903(8) Å
	c = 12.8641(7) Å
	α = 83.210(6)°
	β = 88.683(5)°
	γ = 73.760(7)°
Volume	1000.03(12) $Å^3$
Z	2
Density (calculated)	2.290 Mg/m^3
Absorption coefficient	14.919 mm^{-1}
F(000)	636
Crystal size	0.16 x 0.15 x 0.08 mm^3
Theta range for data collection	3.46 to 67.50°.
Index ranges	-9<=h<=8, -12<=k<=11, -12<=l<=15
Reflections collected	6364
Independent reflections	3603 [R(int) = 0.1375]
Completeness to theta = 67.50°	99.7 %
Absorption correction	Semi-empirical from equivalents
Max. and min. transmission	0.4008 and 0.2057
Refinement method	Full-matrix least-squares on F^2
Data / restraints / parameters	3603 / 218 / 220
Goodness-of-fit on F^2	1.122
Final R indices [I>2sigma(I)]	R1 = 0.1000, wR2 = 0.2298
R indices (all data)	R1 = 0.1348, wR2 = 0.2554
Largest diff. peak and hole	2.524 and -2.535 e.$Å^{-3}$

8.3 Molecular Structure of *i*$Pr_2PhSi^+[CHB_{11}H_5Br_6]^-$ (CCDC-1818581)

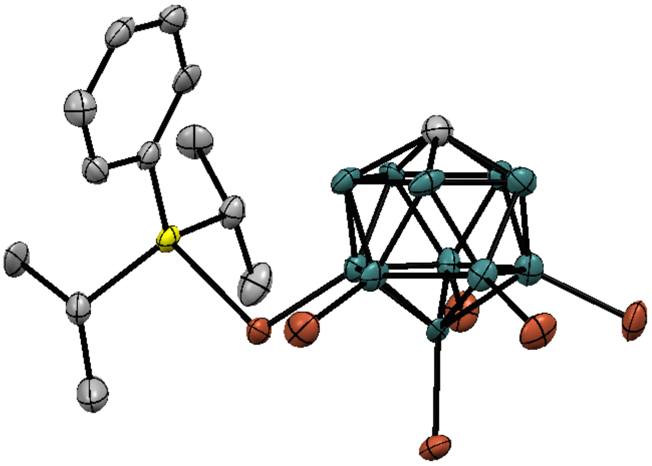

Figure S62. Molecular Structure of *i*$Pr_2PhSi^+[CHB_{11}H_5Br_6]^-$ (thermal ellipsoids at 50% probability level; H atoms and one molecule of *o*-$Cl_2C_6H_4$ omitted for clarity).

Empirical formula	C19 H29 B11 Br6 Cl2 Si
Formula weight	954.78
Temperature	150.00(10) K
Wavelength	1.54184 Å
Crystal system	Monoclinic
Space group	Cc
Unit cell dimensions	a = 10.26054(14) Å
	b = 38.4104(6) Å
	c = 8.64061(13) Å
	α = 90°.
	β = 100.5518(14)°.
	γ = 90°.
Volume	3347.77(9) $Å^3$
Z	4
Density (calculated)	1.894 Mg/m^3
Absorption coefficient	10.566 mm^{-1}
F(000)	1824
Crystal size	0.41 x 0.29 x 0.23 mm^3
Theta range for data collection	4.53 to 67.49°.
Index ranges	-12<=h<=9, -46<=k<=45, -9<=l<=10
Reflections collected	6169
Independent reflections	3859 [R(int) = 0.0309]
Completeness to theta = 67.49°	99.8 %
Absorption correction	Analytical
Max. and min. transmission	0.1949 and 0.0988
Refinement method	Full-matrix least-squares on F^2
Data / restraints / parameters	3859 / 8 / 374
Goodness-of-fit on F^2	1.098
Final R indices [I>2sigma(I)]	R1 = 0.0517, wR2 = 0.1337
R indices (all data)	R1 = 0.0525, wR2 = 0.1345
Absolute structure parameter	0.07(5)
Largest diff. peak and hole	1.029 and -1.757 $e.Å^{-3}$

9 Computational Data

We used Jaguar 9.1 quantum chemistry software[S14] to perform density functional theory calculations with M06 hybrid meta-GGA approximation of the electronic exchange and correlation energies. The Gibbs energies of the calculated structures include free energy and solvation corrections calculated with 6-31G** basis set. In Jaguar program, this basis set is referred to as LACVP**. The electronic energy is reevaluated as a single point energy at the cc-pVTZ(-f) basis, which is a triple-zeta basis set level. The unscaled vibrational frequencies were used to derive zero point energies and other thermodynamic parameters. Solution phase energies were calculated as the self-consistent reaction field (SCRF) energies of the gas phase geometries in the dielectric constant of toluene (ε = 2.379), which is the solvent used in the experiments.

9.1 Evaluation of Silylium Ion Stability

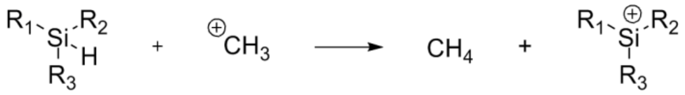

Figure S63. A model hydride transfer reaction to evaluate Si cation relative stability.

Table S1. Gibbs energies (kcal mol^{-1}) of the hydride transfer reaction in Figure S63. Data shows Ph_2MeSi^+ cation is the least electrophilic of the cations examined.

Hydrosilane		Si cation		Δ_rG	$\Delta\Delta_rG^a$
**$PhMe_2Si$–H**	**1CH, 1A**	**$PhMe_2Si^+$**	**1C, 6A**	–83.27	–0.10
**$PhMeSiH_2$**	**2CH**	**$PhMeHSi^+$**	**2C**	-75.87	7.40
**Ph_2MeSi–H**	**3CH, 1B**	**Ph_2MeSi^+**	**3C, 6B**	–84.19	–1.02
**Ph_3Si–H**	**4CH, 18B**	**Ph_3Si^+**	**4C, 19B**	–83.17	0.00
**Me_3Si–H**	**5CH, 19B**	**Me_3Si^+**	**5C, 18B**	–80.93	2.34
**Ph_2SiH_2**	**6CH**	**Ph_2HSi^+**	**6C**	–77.33	5.94
**Me_2SiH_2**	**7CH**	**Me_2HSi^+**	**7C**	–72.37	10.90

^{a} $\Delta\Delta_rG = \Delta_rG - \Delta_rG(4C)$

9.2 Silylium Ion Binding to Various Nucleophiles

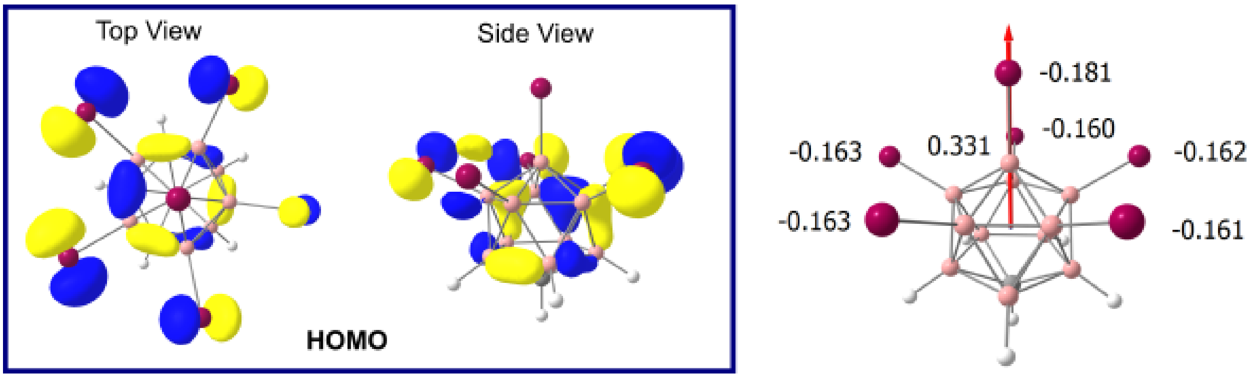

Figure S64. Calculated HOMO of the carborate and its dipole moment (7.946 Debye) is represented by a red arrow. Electrostatic charges are shown for the bromine and the apical boron.

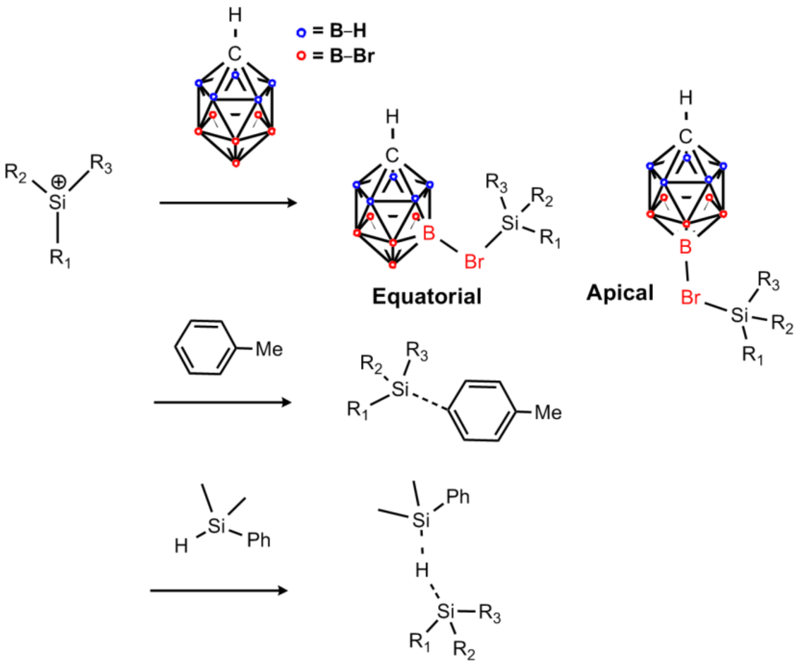

Figure S65 Strategy for calculating binding affinities of Si cations for carborate, toluene and Me_2PhSiH (*via* hydride bridge).

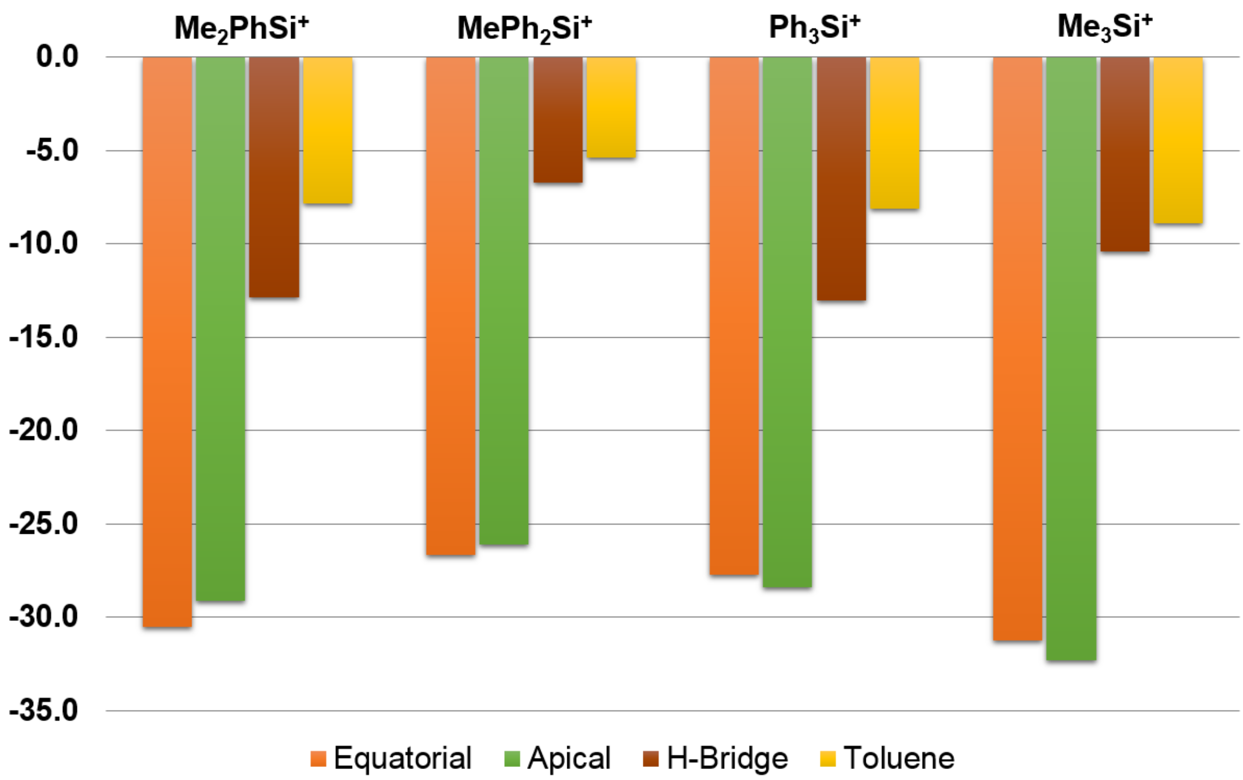

Figure S66. The association energies (kcal mol^{-1}) of selected silylium ions with carborate (quatorial and apical), Me_2PhSiH and toluene. The energies are calculated using free silylium ions as the references.

The calculated HOMO of the carborate in Figure S64 shows the negative charge is delocalized over the equatorial bromine atoms and the carborane cage. The calculated atomic charges from the electrostatic potential analysis show the apical bromine is slightly more electron rich than the equatorial ones. Additionally, the dipole moment of 7.946 Debye aligns with the apical B-Br bond. Consequently, the Si cations can bind to either of the equatorial or the apical bromides. The calculated binding energies, shown in Table S2, suggest that the Si association with the equatorial vs. apical bromide are similar, but the binding to the apical bromide is slightly preferred in most cases. Table S2 also lists the energy associated with the transfer of Si cations from the solvent toluene to carborate. The transfer energy is calculated as the difference in the free energies when Si cation binds with toluene vs. carborate. These energies indicate Me_3Si^+ has a high preference for carborate, and liberates the most energy during the transfer from being toluene bound to carborate bound.

Table S2 Binding energies (kcal mol^{-1}) of Si cations with toluene, carborate and Me_2PhSiH (*via* Hydride Bridge). The differences in the association energies with carborate vs. Toluene are tabulated as transfer energies.

**Cations**	(nC)	**Toluene** (nCtol)	**H-Bridge**	**Carborate-Eq** (nCAn1)	**Carborate-Ap** (nCAn2)	**Transfer Energy**
**$PhMe_2Si^+$**	1C	–7.84	–12.88 (**7A**)	**–30.51**	–29.11	–22.67
**$PhMeHSi^+$**	2C	–12.92	–	–29.07	–33.38	–20.47
**Ph_2MeSi^+**	3C	–5.39	–6.71 (**14B**)	**–26.65**	–26.13	–21.26
**Ph_3Si^+**	4C	–8.11	–13.02 (**11B**)	–27.73	–28.28	–20.17
**Me_3Si^+**	5C	–8.90	–10.41 (**HB**)	–31.23	–32.32	–23.42
**Ph_2HSi^+**	6C	–9.57	–	–27.71	–32.17	–22.60
**Me_2HSi^+**	7C	–15.32	–	–36.88	–38.31	–22.99

n = 1–7

9.3 Evaluation of Methyl Transfer Mechanism

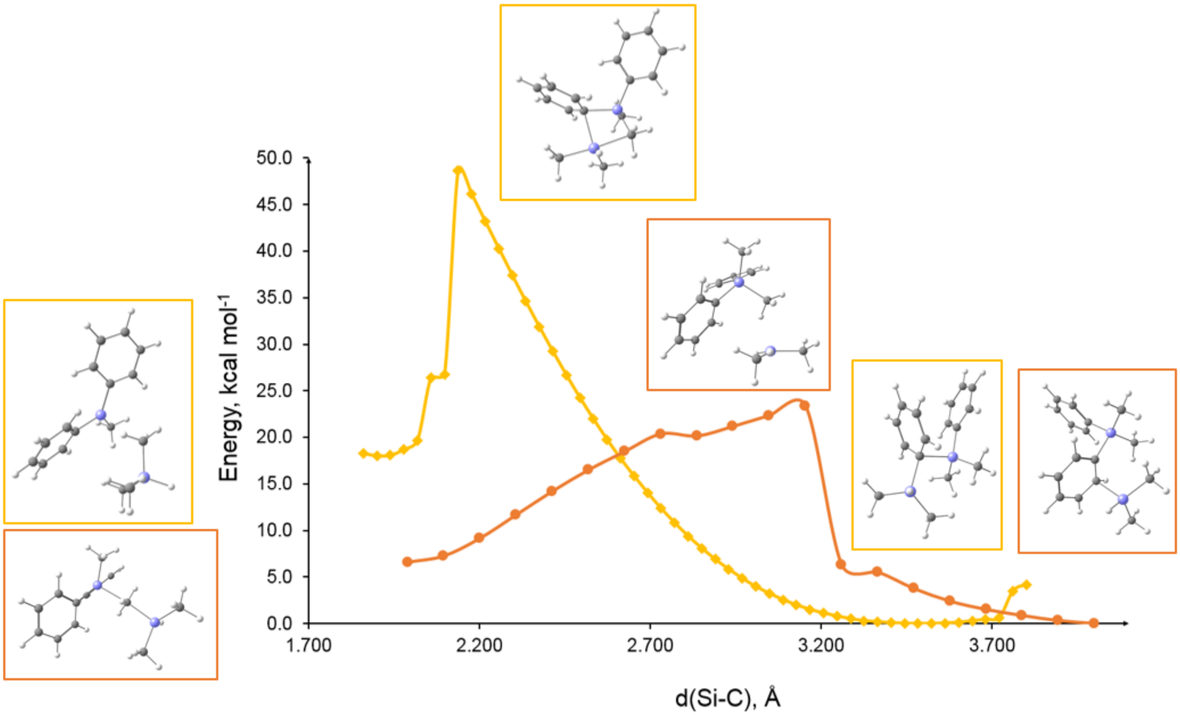

Figure S67. Relaxed geometry scans of **10A** formation from **8A** (yellow curve) and **9A** (orange curve) intermediates.

9.4 Geometries of the Bridged Structures

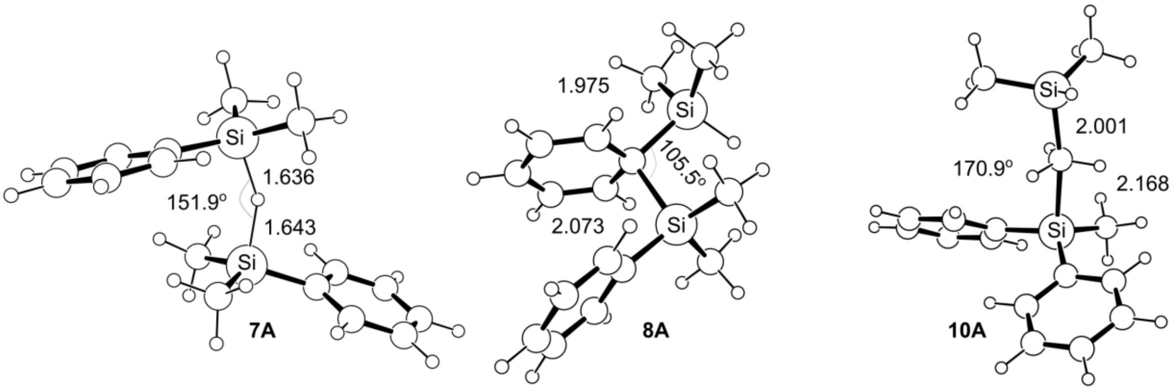

Figure S68. Calculated Hydride (**7A**), phenyl (**8A**) and methyl (**10A**) bridged structures. Bond lengths are in Å and bond angles are in degrees.

9.5 Energy Data

Table S3 Energy data of Me_2PhSiH Reactant

	**E(SCF)**	**ZPE**	**H**	**S**	**G(solv)**	**G(sol)**	**ΔG(sol)**
	eV	kcal/mol	eV	eu	kcal/mol	eV	kcal/mol
	cc-pvtz(-f)	LACVP**		LACVP**	LACVP**		
**00-Carborate**	-10731.366	76.890	-10728.032	141.827	-23.05	-10730.865	0.00
**1A**	-16365.679	108.31	-16360.982	101.121	-1.41	-16362.351	0.00
**2A**	-19930.575	175.43	-19922.968	122.053	-24.29	-19925.599	0.00
**3A**	-36296.702	284.06	-36284.384	188.500	-23.01	-36287.819	3.01
**3A-TS**	-36296.231	282.60	-36283.976	179.817	-22.54	-36287.279	15.47
**4A**	-36296.468	284.24	-36284.142	189.303	-23.65	-36287.615	7.71
**5A**	-19953.653	181.73	-19945.772	128.405	-3.00	-19947.563	7.71
**6A**	-16342.064	103.12	-16337.592	93.215	-30.18	-16340.106	6.45
**7A**	-32709.065	212.22	-32699.862	162.179	-24.36	-32703.015	-6.46
**7A-TS**	-32708.537	212.06	-32699.341	156.550	-24.67	-32702.435	6.93
**8A**	-32709.155	213.10	-32699.914	155.779	-25.24	-32703.023	-6.63
**8A-TS**	-32708.756	212.63	-32699.535	152.438	-25.78	-32702.624	2.56
**9A**	-32708.934	212.91	-32699.701	157.034	-25.72	-32702.847	-2.57
**9A-TS**	-32707.963	211.67	-32698.784	155.023	-27.16	-32701.966	17.80
**10A**	-32708.557	212.55	-32699.340	164.757	-25.31	-32702.568	3.87
**10A-TS**	-32708.435	211.82	-32699.250	161.470	-24.73	-32702.410	7.51
**11A**	-32709.060	212.22	-32699.857	164.468	-24.15	-32703.031	-6.82
**12A Me3Si+**	-11127.056	68.09	-11124.103	89.027	-35.23	-11126.782	3.80
**12A Ph2MeSiH**	-21580.319	142.19	-21574.153	119.286	-2.15	-21575.789	3.80
**13A Me3SiH**	-11151.035	74.25	-11147.815	82.670	-0.61	-11148.911	0.99
**13A MePh2Si+**	-21557.050	137.33	-21551.095	117.386	-26.97	-21553.782	0.99
**6A'**	-27077.395	181.02	-27069.545	199.450	-8.97	-27072.513	24.06
**12A'**	-21862.658	147.04	-21856.282	182.095	-8.41	-21859.001	-27.43
**12A''**	-21862.829	147.08	-21856.451	181.276	-5.85	-21859.048	-28.52
**13A'**	-32292.068	214.89	-32282.749	209.349	-9.00	-32285.846	-25.66

Table S4. Energy data of $MePh_2SiH$ Reactant

	E(SCF) eV cc-pvtz(-f)	ZPE kcal/mol LACVP**	H eV	S eu LACVP**	G(solv) kcal/mol LACVP**	G(sol) eV	ΔG(sol) kcal/mol
**1B**	-21580.327	142.23	-21574.159	118.092	-2.12	-21575.778	0.00
**2B**	-19930.575	175.43	-19922.968	122.053	-24.29	-19925.599	0.00
**3B**	-41511.386	317.97	-41497.598	205.103	-22.87	-41501.241	3.14
**3B-TS**	-41511.016	316.66	-41497.284	194.586	-22.10	-41500.758	14.26
**4B**	-41511.267	318.84	-41497.441	205.163	-23.76	-41501.124	5.84
**5B**	-19953.653	181.73	-19945.772	128.405	-3.00	-19947.563	5.84
**6B**	-21557.050	137.33	-21551.095	117.386	-26.97	-21553.782	0.75
**7B**	-43138.644	280.24	-43126.492	195.334	-22.56	-43129.995	-9.29
**7B-TS**	-43138.040	280.06	-43125.895	191.769	-23.52	-43129.395	4.56
**8B**	-43138.586	281.00	-43126.401	186.517	-23.87	-43129.847	-5.88
**8B-TS**	-43138.375	280.22	-43126.224	185.571	-23.78	-43129.654	-1.42
**9B**	-43138.445	280.76	-43126.270	193.001	-23.58	-43129.788	-4.51
**9B-TS**	-43137.416	279.40	-43125.300	194.368	-26.12	-43128.946	14.91
**10B**	-43138.053	280.38	-43125.895	194.128	-23.19	-43129.410	4.21
**10B-TS**	-43137.898	279.98	-43125.757	199.824	-23.50	-43129.359	5.37
**11B**	-43138.646	280.17	-43126.497	197.918	-22.37	-43130.026	-9.99
**12B Me2PhSi+**	-16342.064	103.12	-16337.592	93.215	-30.18	-16340.106	5.91
**12B Ph3SiH**	-26794.971	176.14	-26787.333	137.051	-2.89	-26789.230	5.91
**13B Ph3Si+**	-26771.717	171.51	-26764.280	134.395	-24.68	-26767.087	3.58
**13B PhMe2SiH**	-16365.679	108.32	-16360.982	101.121	-1.41	-16362.351	3.58
**14B**	-37923.783	246.07	-37913.112	177.252	-23.51	-37916.424	-6.53
**14B-TS**	-37923.570	246.72	-37912.871	168.295	-23.47	-37916.065	1.74
**15B**	-37923.812	247.28	-37913.089	170.000	-24.71	-37916.358	-5.03
**15B-TS**	-37923.500	246.82	-37912.797	165.347	-25.00	-37916.019	2.81
**16B**	-37923.577	247.10	-37912.862	172.804	-24.84	-37916.173	-0.75
**16B-TS**	-37922.502	244.78	-37911.887	180.681	-27.51	-37915.416	16.70
**17B**	-37923.320	246.38	-37912.636	182.115	-23.85	-37916.025	2.67
**17B-TS**	-37923.200	246.20	-37912.524	178.125	-23.02	-37915.825	7.27
**18B**	-37923.750	246.40	-37913.065	179.263	-23.13	-37916.386	-5.66
**19B Me3Si+**	-11127.056	68.09	-11124.103	89.027	-35.23	-11126.782	2.96
**19B Ph3SiH**	-26794.971	176.14	-26787.333	137.051	-2.89	-26789.230	2.96
**20B Ph3Si+**	-26771.717	171.51	-26764.280	134.395	-24.68	-26767.087	3.58
**20B Me3SiH**	-11151.035	74.25	-11147.815	82.670	-0.61	-11148.911	3.58
**6B'**	-32292.068	214.90	-32282.749	209.349	-9.00	-32285.846	-25.90
**12B'**	-27077.395	181.03	-27069.545	199.450	-8.97	-27072.513	-24.60
**13B'' , 20B”**	-37506.620	248.83	-37495.830	235.919	-7.03	-37499.185	-24.70
**19B''**	-21862.829	147.08	-21856.451	181.276	-5.85	-21859.048	-29.36

Table S5. Energy data of Structures in Table S1 and Table S2

	E(SCF)	ZPE	H	S	G(solv)	G(sol)	ΔG(sol)
	eV	kcal/mol	eV	eu	kcal/mol	eV	kcal/mol
	cc-pvtz(-f)	LACVP**		LACVP**	LACVP**		
**00-CH3+**	-1073.660	19.25	-1072.825	44.642	-50.57	-1075.595	
**00-CH4**	-1101.830	28.04	-1100.614	47.242	-0.22	-1101.234	
**00-Carborate**	-10731.366	76.89	-10728.032	141.827	-23.05	-10730.865	
**00-toluene**	-7385.728	79.92	-7382.262	79.537	-1.40	-7383.352	
**1C**	-16342.154	102.88	-16337.693	102.204	-30.22	-16340.325	
**1CH**	-16365.680	108.32	-16360.983	101.121	-1.44	-16362.353	-83.27
**1CAn**	-27077.395	181.03	-27069.545	199.450	-8.97	-27072.513	-30.51
**1Ctol**	-23729.133	184.47	-23721.134	134.726	-26.30	-23724.016	-7.84
**2C**	-15272.245	85.17	-15268.552	91.145	-31.33	-15271.089	
**2CH**	-15296.095	90.52	-15292.170	93.740	-1.30	-15293.438	-75.87
**2CAn**	-26007.619	163.26	-26000.539	179.448	-8.19	-26003.214	-29.07
**2Ctol**	-22659.424	166.73	-22652.194	127.005	-26.85	-22655.000	-12.92
**3C**	-21557.050	137.33	-21551.095	117.386	-26.97	-21553.782	
**3CH**	-21580.311	142.08	-21574.150	117.962	-2.20	-21575.770	-84.19
**3CAn**	-32292.068	214.90	-32282.749	209.349	-9.00	-32285.846	-26.65
**3Ctol**	-28943.840	218.44	-28934.367	151.757	-24.94	-28937.411	-5.39
**4C**	-26771.717	171.50	-26764.280	134.531	-24.77	-26767.093	
**4CH**	-26794.965	176.01	-26787.333	128.878	-2.93	-26789.126	-83.17
**4CAn**	-37506.652	248.80	-37495.863	224.814	-9.02	-37499.161	-27.73
**4Ctol**	-34158.527	252.47	-34147.579	169.268	-23.73	-34150.796	-8.11
**5C**	-26794.965	176.01	-26787.333	128.878	-2.93	-26789.126	
**5CH**	-11151.035	74.26	-11147.815	82.712	-0.62	-11148.911	-80.93
**5CAn**	-21862.658	147.04	-21856.282	182.095	-8.41	-21859.001	-31.23
**5Ctol**	-18514.303	150.43	-18507.780	119.615	-27.50	-18510.519	-8.90
**6C**	-20487.207	119.50	-20482.025	108.328	-27.48	-20484.617	
**6CH**	-20510.736	124.47	-20505.339	114.161	-2.05	-20506.903	-77.33
**6CAn**	-31222.268	197.25	-31213.715	201.159	-8.50	-31216.684	-27.71
**6Ctol**	-27874.115	200.67	-27865.413	145.084	-25.25	-27868.384	-9.57
**7C**	-10057.034	50.32	-10054.852	76.978	-37.76	-10057.485	
**7CH**	-10081.476	56.57	-10079.023	72.944	-0.45	-10079.986	-72.37
**7CAn**	-20792.912	129.29	-20787.305	176.444	-8.35	-20789.949	-36.88
**7Ctol**	-17444.609	132.81	-17438.850	110.707	-28.12	-17441.500	-15.32
**1CAn2**	-27077.453	181.05	-27069.602	198.468	-6.54	-27072.452	-29.11
**2CAn2**	-26007.734	163.31	-26000.652	193.131	-6.46	-26003.429	-33.38
**3CAn2**	-32292.067	215.07	-32282.741	212.091	-6.86	-32285.780	-26.13
**4CAn2**	-37506.620	248.83	-37495.830	235.919	-7.03	-37499.185	-28.42
**5CAn2**	-21862.829	147.08	-21856.451	181.276	-5.85	-21859.048	-32.32
**6CAn2**	-31222.442	197.18	-31213.891	210.365	-6.04	-31216.873	-32.17
**7CAn2**	-20793.103	129.39	-20787.492	175.074	-5.84	-20790.009	-38.31
**HB**	-27494.309	178.21	-27486.581	145.727	-25.80	-27489.584	

9.6 Coordinates of the Calculated Structures

==============================
1A
==============================

Si	-2.2010587379	-0.2625150744	-5.5481060162
C	-2.6788657607	-1.0648806125	-7.1795520101
H	-3.7653490278	-1.0945225447	-7.3124363685
H	-2.2576597553	-0.4925126476	-8.0155844398
H	-2.2990376385	-2.0898963394	-7.2525043378
C	-2.8382288695	1.5023545887	-5.4966124492
C	-3.7333881975	1.9355830959	-4.5122479262
C	-2.4072408015	2.4390713051	-6.4456565967
C	-4.1876509860	3.2507756618	-4.4780718784
C	-2.8550651381	3.7540546952	-6.4189217706
C	-3.7487863848	4.1613299426	-5.4325180794
H	-4.0857755338	1.2309622483	-3.7585852857
H	-1.7066753146	2.1353079380	-7.2262767533
H	-4.8856353826	3.5660649708	-3.7051247846
H	-2.5087148173	4.4642644891	-7.1668891604
H	-4.1016751052	5.1901984642	-5.4081130981
C	-0.3275528112	-0.2563249779	-5.3904088363
H	-0.0016738171	0.1921969156	-4.4463460908
H	0.0874051585	-1.2687987057	-5.4476629645
H	0.1137408744	0.3299197384	-6.2061851800
H	-2.8219344840	-0.9939834601	-4.4082510384

==============================
2A
==============================

C	-1.2358184993	2.8052108999	-3.0531558142
C	-0.7081322697	2.1002633615	-1.9716002483
C	-1.8183612395	2.1288095808	-4.1249421025
C	-0.7478011253	0.7186271734	-1.9639590926
C	-1.8890457396	0.7484977673	-4.1125458585
C	-1.3457923037	0.0147867822	-3.0340353434
C	-4.7004289516	-3.4040119070	-4.8002941132
C	-4.8884181138	-2.1736200689	-4.1709085577
C	-3.4348528734	-3.9876872987	-4.8531104367
C	-3.8113753066	-1.5165458579	-3.6059921403
C	-2.3573845089	-3.3523830126	-4.2642215366
C	-2.5243858942	-2.0984878039	-3.6336998443
C	-1.4050941680	-1.4247456909	-3.0253484838
C	1.7008466563	-3.6623832360	-1.2130341556
C	2.0000942144	-2.4663833258	-1.8650461895
C	-0.6277164051	-3.4091622181	-1.7556112220
C	0.9873163198	-1.7251986090	-2.4451830984
C	-0.3481536745	-2.1872307394	-2.4093082776
H	-3.9602838742	-0.5733254895	-3.0861761919
H	-1.3642774538	-3.7893174049	-4.3316163933
H	-3.2934986205	-4.9376373475	-5.3604024678
H	-5.5477344493	-3.9128807378	-5.2528872010
H	-5.8804755217	-1.7348094862	-4.1170693935
H	1.2216338552	-0.8095877788	-2.9822483722
H	3.0278918358	-2.1202756017	-1.9239048573
C	0.3876410652	-4.1282949807	-1.1525475300
H	-2.3132300998	0.2164104928	-4.9605330240
H	-0.3712064852	0.1671491698	-1.1060890922
H	-0.2769946396	2.6355936431	-1.1306775264
H	-1.1926253646	3.8912824406	-3.0611495354
H	-2.2098388718	2.6843061763	-4.9721674564
H	2.4982742758	-4.2367161350	-0.7483387911
H	0.1602864623	-5.0505821352	-0.6259034010
H	-1.6564258288	-3.7522217038	-1.6804146358

==============================
3A
==============================

C	7.069235451	11.197978189	-1.024742190
C	7.448709726	10.082203697	-0.268202735
C	6.248417939	12.154777665	-0.409428069
C	7.020382929	9.920587870	1.046700637
C	5.809339143	11.997006534	0.901107826
C	6.194103121	10.875175253	1.631347093
H	8.095753245	9.323509889	-0.708526519
H	5.941330894	13.043792323	-0.964938330
H	7.337605153	9.051334283	1.619048518
H	5.175472323	12.754061581	1.358733995
H	5.861343669	10.752616989	2.659457763
H	6.386563574	11.063197643	-3.668398650
C	8.073092076	13.173154784	-3.158706062
H	7.265783260	13.876897915	-2.922779532
H	8.937938595	13.450743503	-2.545564922
H	8.348629182	13.319465697	-4.209033182
Si	7.584234174	11.393618258	-2.822076051
C	8.972078487	10.207862819	-3.241328051
H	9.315465070	10.358066417	-4.270389797
H	9.831753503	10.368519681	-2.580335380
H	8.667503135	9.159893469	-3.144557162
C	3.557481888	10.741195166	-3.485262385
C	3.405994214	11.981927230	-2.763913168
C	4.030800528	13.161050014	-3.227796661
C	2.623093019	12.044695501	-1.591324502
C	3.883047940	14.350236243	-2.536972001
C	2.458592994	13.243982793	-0.921893208
C	3.091180760	14.394723902	-1.389322794
H	4.676786699	13.113441878	-4.101455320
H	2.103855442	11.154370524	-1.247331957
H	4.389017650	15.246143984	-2.886348248
H	1.829623205	13.288190796	-0.037424756
H	2.966264955	15.333749506	-0.856070757
C	3.575787366	9.488531304	-2.777459989
C	4.107604309	9.405985375	-1.469609115
C	3.060242718	8.316644185	-3.379280240
C	4.135496415	8.192846323	-0.805255744
C	3.065383205	7.117752615	-2.693873601
C	3.609832614	7.053042531	-1.410406848
H	4.551061092	10.286060007	-1.007571000
H	2.607115527	8.376812324	-4.365034556
H	4.578287820	8.137863307	0.185492380
H	2.638799873	6.229734088	-3.151313722
H	3.621797228	6.104779470	-0.878809148
C	3.674938214	10.764381330	-4.923503271
C	4.480289236	9.818135465	-5.594894494
C	2.994870422	11.739438150	-5.685262410
C	4.608822601	9.859551982	-6.970622887
C	3.107194942	11.756127455	-7.063448592
C	3.918996383	10.822895621	-7.705803329
H	5.047277175	9.095047386	-5.014542563
H	2.339058070	12.446779994	-5.184957238
H	5.254317883	9.147294116	-7.476245623
H	2.558751332	12.492958659	-7.643066057
H	4.016102762	10.847177236	-8.788183662

==============================
3A-TS
==============================

C	6.266732889	11.847826764	-1.262983465
C	5.878000043	11.242867423	-0.056707790
C	6.454374699	13.238545359	-1.280177692
C	5.667142727	12.001328247	1.086705065
C	6.258774988	13.998597063	-0.133881864
C	5.854090423	13.380874885	1.046044293
H	5.752338077	10.159647500	-0.003867543

H	6.760066905	13.737123380	-2.200532891
H	5.365542665	11.519558513	2.013604232
H	6.419546050	15.073791401	-0.159134055
H	5.692948434	13.975750207	1.941988512
H	4.880461672	10.589173592	-3.188283998
C	7.057387290	11.823221314	-4.282098834
H	6.410655760	12.672153538	-4.532686024
H	8.058878042	12.215625105	-4.067786724
H	7.131457732	11.186695398	-5.170307007
Si	6.414602769	10.858740069	-2.820829108
C	7.122499496	9.143150051	-2.644217901
H	6.993100683	8.570657759	-3.571148953
H	8.197829489	9.206985904	-2.436769115
H	6.656629582	8.574999929	-1.831337648
C	3.382668185	10.389324028	-3.444929302
C	2.857878968	11.306150526	-2.416990069
C	3.259119671	12.652810109	-2.413814751
C	1.953282902	10.869958895	-1.437521767
C	2.784670655	13.530018363	-1.455113611
C	1.462699089	11.757733350	-0.490277200
C	1.883353517	13.082842203	-0.490166398
H	3.967378309	13.000649486	-3.165152005
H	1.605649458	9.840998493	-1.440976246
H	3.121161660	14.563265920	-1.452795288
H	0.745868108	11.411928866	0.249444421
H	1.505404167	13.772407235	0.260257783
C	3.328024356	8.935770575	-3.180521627
C	3.740720365	8.445274290	-1.930115633
C	2.851265082	8.030788499	-4.138195021
C	3.687623789	7.092831243	-1.646231722
C	2.781836538	6.674404911	-3.843268717
C	3.203393510	6.203217911	-2.605873715
H	4.116006096	9.151952757	-1.190157453
H	2.502482186	8.392790470	-5.100988799
H	4.020870254	6.725590875	-0.679125420
H	2.393739459	5.982541243	-4.585695082
H	3.155152910	5.140336486	-2.383708223
C	3.359023395	10.839248037	-4.852060284
C	4.227864584	10.256954681	-5.789213760
C	2.440762344	11.805322803	-5.283588734
C	4.185215764	10.634702589	-7.119105207
C	2.388109843	12.167734010	-6.624536141
C	3.259554899	11.590578158	-7.539526788
H	4.948972122	9.510586711	-5.454914326
H	1.744640213	12.245255619	-4.574383133
H	4.870525504	10.187158516	-7.833801266
H	1.659771525	12.903595427	-6.953994509
H	3.220709165	11.883035819	-8.585533720

==============================

4A

==============================

C	7.348948234	9.368242648	-3.009400583
C	7.795799516	9.076273607	-4.312057301
C	7.439223000	8.371615135	-2.021403038
C	8.316069347	7.826985558	-4.613520754
C	7.962441803	7.124250189	-2.325476080
C	8.397374146	6.853478924	-3.620313102
H	7.740781503	9.833168355	-5.094615241
H	7.105549109	8.572674162	-1.004245268
H	8.663551370	7.609100439	-5.619709415
H	8.034398002	6.361247872	-1.555327276
H	8.806896553	5.874725593	-3.857580179
H	4.901143865	10.765197536	-3.011273647
C	6.452454868	11.526105696	-0.862666423
H	6.337875809	10.677362288	-0.182314517
H	7.324849311	12.108075701	-0.538282017
H	5.571093014	12.174115976	-0.769111843
Si	6.692901788	11.024013998	-2.624526703

C	6.955368394	12.352993724	-3.884575975
H	6.701794098	13.337424957	-3.478645477
H	8.007934836	12.369078509	-4.194054142
H	6.346389780	12.174905982	-4.781365577
C	3.744339761	10.718189211	-3.242823024
C	3.150044056	11.735323345	-2.300240892
C	3.599919832	13.062590757	-2.357642000
C	2.168823153	11.397038874	-1.365987862
C	3.090408700	14.023143339	-1.496257794
C	1.649014600	12.363735376	-0.508829097
C	2.110347724	13.672595343	-0.565834513
H	4.346431741	13.346026773	-3.103456313
H	1.799414272	10.375734669	-1.312540909
H	3.450546236	15.047908128	-1.553324088
H	0.878846033	12.086223494	0.208834049
H	1.706692318	14.423006441	0.109307048
C	3.398927854	9.282299874	-2.932736418
C	3.906241385	8.684306831	-1.776239179
C	2.533764535	8.552046603	-3.747981918
C	3.574672153	7.379012321	-1.445138907
C	2.187439671	7.247857471	-3.408863026
C	2.710341492	6.656961440	-2.264939144
H	4.555768355	9.262183483	-1.115817457
H	2.123611233	9.006307210	-4.647530802
H	3.984061699	6.924823674	-0.545539039
H	1.506398401	6.690613568	-4.047445146
H	2.444020711	5.634759329	-2.008901638
C	3.659206810	11.060843698	-4.708707790
C	4.471764964	10.358221650	-5.607676046
C	2.761879856	12.005553230	-5.204871638
C	4.405852649	10.610807500	-6.969494419
C	2.686204406	12.248294605	-6.573906993
C	3.509338910	11.560829805	-7.455870533
H	5.146079672	9.588106492	-5.228289248
H	2.109544458	12.545811759	-4.523068336
H	5.043112751	10.059044158	-7.656213752
H	1.976182094	12.980540723	-6.949757655
H	3.450148307	11.757349152	-8.523132185

==============================
5A
==============================

C	-1.217728800	-0.257537446	-2.609031636
C	-2.724579435	-0.093927238	-2.661913184
C	-3.363573476	1.088812496	-2.293607027
C	-3.506424753	-1.186369538	-3.043596638
C	-4.752862397	1.177125718	-2.308425837
C	-4.891905230	-1.100590023	-3.062640422
C	-5.521010416	0.085391138	-2.693819586
H	-2.767779125	1.950146467	-1.997185489
H	-3.012217445	-2.118044229	-3.320688347
H	-5.235480273	2.108095764	-2.018713713
H	-5.483876410	-1.960922554	-3.367219046
H	-6.606124637	0.157113521	-2.709111604
C	-0.761126330	-1.041278525	-1.392980885
C	-1.498878149	-1.089403520	-0.211395857
C	0.473961231	-1.691480068	-1.439399777
C	-1.011349172	-1.773806722	0.898720315
C	0.961214874	-2.377198896	-0.335291844
C	0.217325559	-2.419986040	0.840756102
H	-2.464662193	-0.589013210	-0.161205719
H	1.060572491	-1.645342834	-2.357420188
H	-1.598988876	-1.801987445	1.813817118
H	1.924052542	-2.880430932	-0.391242571
H	0.595370584	-2.956705719	1.707872582
C	-0.485789552	1.063530717	-2.755068315
C	0.069552908	1.738001669	-1.668841867
C	-0.410932886	1.653500494	-4.018841123
C	0.687619430	2.973178650	-1.843944707

C	0.207383983	2.883537587	-4.197112319
C	0.760402066	3.548863170	-3.106376834
H	0.022173975	1.289761941	-0.677737830
H	-0.855310412	1.136561210	-4.869936338
H	1.116879189	3.486009178	-0.985884258
H	0.259443957	3.325196793	-5.189937396
H	1.247263445	4.511901903	-3.242223667
H	-0.941480347	-0.862491837	-3.488754875

==============================

6A

==============================

C	-2.624725336	0.433110404	-4.434501679
C	-3.510211796	-0.580850138	-4.864042712
C	-2.915263901	1.779762410	-4.750159329
C	-4.643987494	-0.255910547	-5.586579500
C	-4.053632262	2.097025419	-5.468447844
C	-4.912887840	1.079812559	-5.885613492
H	-3.306816062	-1.625068788	-4.633787442
H	-2.248259348	2.577934101	-4.427891370
H	-5.322608729	-1.036155994	-5.918595494
H	-4.277638878	3.132235379	-5.708566213
H	-5.805726001	1.332214963	-6.452304137
C	-0.751628936	-1.711645265	-3.057442278
H	-0.325786558	-1.749632021	-2.033787677
H	-1.627511172	-2.385455338	-3.118736435
H	0.034137976	-2.097844750	-3.749638109
Si	-1.145665950	0.024703048	-3.505308792
C	0.008723889	1.358709647	-2.985245120
H	0.981348570	0.923091305	-2.622805701
H	0.199408769	2.079798811	-3.830944868
H	-0.459120573	1.948629805	-2.129551787

==============================

7A

==============================

C	-2.687067223	-1.827053161	-1.037553019
C	-4.085484530	-1.755762638	-0.921295981
C	-1.949634430	-2.435693435	-0.006967669
C	-4.728145240	-2.282986252	0.191154813
C	-2.592262659	-2.957711204	1.106498165
C	-3.980988081	-2.881098775	1.203383562
H	-4.682030416	-1.294803731	-1.709708136
H	-0.862673671	-2.508943678	-0.074580278
H	-5.812090533	-2.231069568	0.268964331
H	-2.013827806	-3.429318307	1.898166551
H	-4.484548896	-3.293851048	2.073927436
H	-1.276532612	0.281050408	-1.913004319
C	-0.235942664	-1.936623911	-3.004189847
H	0.461550196	-2.021986503	-2.164046171
H	-0.431268968	-2.950150969	-3.374453196
H	0.249904287	-1.376176018	-3.811054793
Si	-1.839155951	-1.139718717	-2.515994155
C	-2.935887285	-0.568426764	-3.898745485
H	-2.346779278	-0.168973047	-4.732090707
H	-3.527877827	-1.412610161	-4.272422419
H	-3.636974968	0.208475771	-3.571310810
C	-0.571075333	2.014848265	-3.917828104
C	0.446768399	1.457890926	-4.708979573
C	-1.604345440	2.722030531	-4.551843463
C	0.420226183	1.585317156	-6.091850704
C	-1.626990209	2.856836578	-5.934386192
C	-0.619424480	2.281410162	-6.703754657
H	1.276080968	0.924924540	-4.242466904
H	-2.402147930	3.174044963	-3.962534522
H	1.213231203	1.149941867	-6.694078750
H	-2.429880569	3.410635399	-6.413714379
H	-0.639690924	2.382577827	-7.785757250
C	1.034819979	1.443572986	-1.275003466
H	0.899970619	1.114492463	-0.239167027

H	1.623669323	0.686107683	-1.803402830
H	1.624911252	2.368054986	-1.258699790
Si	-0.599355218	1.759700243	-2.094911393
C	-1.857131986	2.750435729	-1.157088145
H	-2.869163801	2.585499634	-1.544637665
H	-1.853272295	2.485917690	-0.095086443
H	-1.638222874	3.821675401	-1.240389243

==============================

7A-TS

==============================

C	-3.034402502	-0.785322040	-1.485553031
C	-4.320242173	-0.219620804	-1.399069788
C	-2.577095139	-1.610466749	-0.439746412
C	-5.128162379	-0.480607221	-0.301555208
C	-3.388832565	-1.873798966	0.651279590
C	-4.662025168	-1.307304623	0.718717406
H	-4.695449000	0.423567395	-2.195275304
H	-1.578539577	-2.046328960	-0.480973727
H	-6.121362548	-0.043655443	-0.239858152
H	-3.034722320	-2.517694742	1.452799970
H	-5.296582857	-1.512716237	1.577599333
H	-0.557062471	0.412145947	-1.287854862
C	-0.625830523	-1.697773726	-3.305828001
H	0.138465147	-1.673842909	-2.521240521
H	-1.034970291	-2.717614527	-3.338959464
H	-0.155752463	-1.478417796	-4.269996834
Si	-2.021792596	-0.537048987	-2.973536206
C	-2.837367310	0.306816323	-4.393091182
H	-2.110512103	0.615425693	-5.151123660
H	-3.528074636	-0.418664633	-4.848684576
H	-3.420172182	1.181489200	-4.087008004
C	-0.342878147	2.202441510	-3.239063771
C	0.544557178	1.476839500	-4.054241939
C	-1.167649960	3.157517633	-3.853010244
C	0.591932803	1.679041487	-5.429977613
C	-1.119756314	3.370351273	-5.226378717
C	-0.245316555	2.626101070	-6.016288322
H	1.225364739	0.750730786	-3.602423424
H	-1.858006912	3.747580113	-3.249180723
H	1.288576923	1.112233765	-6.043901081
H	-1.762222824	4.118680611	-5.685066937
H	-0.208715021	2.792355523	-7.090363120
C	1.313591792	2.301283587	-0.614012549
H	1.341900576	2.029119100	0.446141631
H	2.126148581	1.772439093	-1.123951292
H	1.514142301	3.376011534	-0.693770084
Si	-0.339596541	1.907392656	-1.382946394
C	-1.801814674	2.741567831	-0.571931891
H	-2.747240699	2.461724430	-1.054150536
H	-1.875385062	2.467399807	0.485914413
H	-1.711643920	3.833113230	-0.624277094

==============================

8A

==============================

C	-0.248228344	-1.924347936	-2.873626269
C	0.166305583	-2.108714253	-4.231100659
C	-0.293519464	-3.103518855	-2.066288774
C	0.548255768	-3.341507010	-4.720628153
C	0.085147134	-4.340059689	-2.551783237
C	0.511785090	-4.453366751	-3.875807457
H	0.188226934	-1.241706709	-4.893396046
H	-0.615125483	-3.017463734	-1.027706852
H	0.871061120	-3.451213819	-5.751978449
H	0.057902434	-5.216475704	-1.910768847
H	0.817586379	-5.424789677	-4.257666356
H	-1.447507420	0.586413731	-2.912404126
C	-2.971402999	-1.423655241	-4.109401561

H	-2.573362116	-1.175809957	-5.098687424
H	-3.083574796	-2.513761350	-4.050219418
H	-3.970819918	-0.981836830	-4.031150075
Si	-1.877906404	-0.819097070	-2.723256620
C	-2.676021731	-1.115868673	-1.063970364
H	-3.545130289	-0.453844584	-0.972648572
H	-3.047570128	-2.144850452	-0.992835040
H	-2.025846992	-0.918248896	-0.206656945
C	2.718167259	-1.936839364	-1.939837926
C	2.952002319	-2.642917102	-0.750483673
C	3.601323753	-2.129620534	-3.011402146
C	4.028189883	-3.513500369	-0.636445212
C	4.678541929	-3.000856082	-2.901216797
C	4.890775194	-3.693017574	-1.713630353
H	2.290477466	-2.508742802	0.106275779
H	3.455930276	-1.588911821	-3.947050850
H	4.200364693	-4.047331324	0.294722703
H	5.358356991	-3.134284745	-3.738892037
H	5.736130430	-4.370715472	-1.624234259
C	0.703820810	-0.163181148	-0.410520623
H	-0.082233637	0.595152293	-0.491795114
H	0.360488045	-0.955791873	0.262802290
H	1.567278962	0.314878534	0.070643374
Si	1.279912564	-0.772513569	-2.076099522
C	1.597624851	0.623491346	-3.268661231
H	2.037769142	0.292562819	-4.214725872
H	0.702351821	1.213851701	-3.488482704
H	2.323504865	1.295431738	-2.793066970

==============================

8A-TS

==============================

C	-0.624654123	-1.403700836	-2.523005755
C	-0.653212721	-1.559567266	-3.933844406
C	-1.471825355	-2.266800619	-1.775356150
C	-1.414620621	-2.540299775	-4.538851618
C	-2.265264675	-3.254970755	-2.399396551
C	-2.227267487	-3.392323376	-3.771842386
H	-0.022761784	-0.918935512	-4.549615092
H	-1.390474907	-2.277674413	-0.687171859
H	-1.381945433	-2.659950081	-5.619171915
H	-2.885014387	-3.909012702	-1.790820109
H	-2.816335811	-4.163344599	-4.261029696
H	-1.752834667	0.962580267	-2.078362497
C	-3.806568782	-0.428924313	-3.331963404
H	-3.374796087	-0.389510815	-4.334696020
H	-4.417048294	-1.331766728	-3.238130492
H	-4.477097246	0.434949997	-3.228966351
Si	-2.564989858	-0.266157690	-1.965700338
C	-3.310448883	-0.427370724	-0.266422683
H	-3.995169167	0.417474920	-0.118195086
H	-3.890760929	-1.351675895	-0.171415229
H	-2.565165313	-0.393206109	0.534305322
C	2.253239941	-1.680913313	-1.890692634
C	2.384334099	-2.733535125	-0.972298044
C	3.155168886	-1.613865962	-2.962348542
C	3.385333049	-3.685968513	-1.116643726
C	4.157484535	-2.565283544	-3.110753915
C	4.271583672	-3.601338583	-2.187901603
H	1.700992959	-2.810975432	-0.123543918
H	3.084829742	-0.803022423	-3.689555681
H	3.479029063	-4.492746176	-0.392436046
H	4.853896326	-2.495974215	-3.943870900
H	5.056370602	-4.344955677	-2.302013101
C	0.463806516	-0.076980974	0.061917270
H	-0.300624356	0.702108783	0.169026680
H	0.150537273	-0.955478350	0.639087458
H	1.373131579	0.302203570	0.543672052
Si	0.864888108	-0.452426693	-1.728162799

C	1.173792688	1.101914996	-2.716844032
H	1.302439627	0.918585043	-3.789683016
H	0.369536680	1.835896679	-2.594868979
H	2.099249584	1.570682420	-2.361254765

==============================
9A
==============================

C	-0.555798476	-1.691579682	-2.246270670
C	-0.503720125	-2.306126150	-3.498815100
C	-1.723867941	-1.935176650	-1.451681411
C	-1.510049103	-3.167642724	-3.929163048
C	-2.703038454	-2.888686942	-1.872098804
C	-2.613884217	-3.467427751	-3.119428569
H	0.365480342	-2.146054348	-4.137017433
H	-1.655963068	-1.731931458	-0.374494882
H	-1.428506058	-3.634513380	-4.908646061
H	-3.531738819	-3.127527546	-1.208240619
H	-3.368157655	-4.170921266	-3.459072232
H	-1.849929635	0.793290550	-1.207503202
C	-3.031121867	0.090700651	-3.640152357
H	-2.090319444	0.246260471	-4.175775685
H	-3.573872025	-0.737077542	-4.108134767
H	-3.641638802	0.993034021	-3.769201596
Si	-2.786671101	-0.170191467	-1.820576036
C	-4.354982861	-0.355955223	-0.839159919
H	-4.819057630	0.630004967	-0.718511879
H	-5.075926075	-1.001190694	-1.352354248
H	-4.177056006	-0.756009129	0.164953849
C	2.419695564	-1.670179724	-2.102049295
C	2.650454894	-2.866560205	-1.406730873
C	3.302255036	-1.318583531	-3.131218952
C	3.729292294	-3.681216704	-1.723578634
C	4.384271276	-2.131944317	-3.451378978
C	4.597138290	-3.312091105	-2.747611835
H	1.979141160	-3.168827934	-0.600482828
H	3.154006539	-0.393501912	-3.688399033
H	3.897775157	-4.603027030	-1.172311899
H	5.064925688	-1.842430669	-4.248128327
H	5.443908793	-3.947039228	-2.996126565
C	0.783445110	-0.339797342	0.169587592
H	-0.083952367	0.274359800	0.439067630
H	0.724023182	-1.284615654	0.723492355
H	1.674669159	0.184674928	0.532105799
Si	0.942922755	-0.620026000	-1.672604541
C	0.913649353	0.962140327	-2.675602264
H	0.870407123	0.756823985	-3.752443925
H	0.063266556	1.603041100	-2.417487390
H	1.825467542	1.541599642	-2.487957152

==============================
9A-TS
==============================

H	-3.835787773	-1.969757915	-1.361427426
C	-5.954818249	-0.154704452	-1.896690369
H	-6.140125751	-0.113448530	-0.656393051
H	-6.021147728	0.935804129	-2.399835348
H	-6.778791904	-0.943343878	-2.364395380
Si	-4.292693615	-0.851224720	-2.197197437
C	-5.267311096	-2.478243113	-4.731244564
H	-5.825335026	-1.531256080	-4.767399788
H	-4.627340794	-2.532990456	-5.618515015
H	-4.574442863	-2.484679937	-3.850068331
C	-5.538505554	-5.533025742	-4.496491909
C	-5.796998978	-6.578792572	-3.597216845
C	-4.598942757	-5.751842976	-5.515947342
C	-5.135603905	-7.797166824	-3.708422422
C	-3.934958935	-6.967775822	-5.630563736
C	-4.202580452	-7.990439415	-4.723723888

H	-6.526005268	-6.440975666	-2.796855450
H	-4.384045601	-4.964154243	-6.241730213
H	-5.348936558	-8.597935677	-3.002962828
H	-3.209383249	-7.120467186	-6.427338123
H	-3.684030533	-8.942654610	-4.811169624
Si	-6.471710205	-3.923495054	-4.400836945
C	-7.888786793	-3.801304817	-5.611683846
H	-8.663309097	-4.541102886	-5.381569386
H	-8.351440430	-2.806695223	-5.612888336
H	-7.534763336	-4.005905628	-6.628247738
C	-3.040279150	-0.001434812	-3.227644682
H	-2.268233776	-0.691653728	-3.585241556
H	-3.495406628	0.516663849	-4.076717377
H	-2.543839931	0.756517172	-2.600733757
C	-7.023809433	-3.591034651	-2.632732153
C	-8.270326614	-3.025738716	-2.334398270
C	-6.133956432	-3.806639433	-1.568194032
C	-8.617173195	-2.689606667	-1.026944637
C	-6.473389149	-3.469588280	-0.258662283
C	-7.717580795	-2.908774853	0.011733352
H	-8.993721008	-2.856434584	-3.132107019
H	-5.170584679	-4.284255028	-1.760417819
H	-9.595340729	-2.264574528	-0.818387270
H	-5.776803017	-3.666985989	0.553678453
H	-7.992936611	-2.657066584	1.032485247

==============================

10A

==============================

H	-1.746505163	-0.945071323	-3.120141933
C	-3.369343430	-0.045023463	-1.024444464
H	-2.737940091	0.756954759	-0.626728721
H	-4.413676281	0.238176094	-0.845705783
H	-3.155831517	-0.948606390	-0.442668412
Si	-3.046108780	-0.302530706	-2.839470597
C	-4.374056340	-1.698295673	-3.382467070
H	-5.241348357	-1.051119638	-3.223325104
H	-4.038474926	-1.893513162	-4.403758516
H	-4.070424778	-2.393996141	-2.594671473
C	-4.978245347	-4.765679335	-3.789666165
C	-5.033215666	-5.723788476	-2.765997110
C	-4.221677188	-5.045631375	-4.938736958
C	-4.347500824	-6.926338285	-2.888669614
C	-3.536237336	-6.245429287	-5.059000228
C	-3.599743551	-7.184565031	-4.032260076
H	-5.628889684	-5.535609630	-1.873267668
H	-4.170536938	-4.324752747	-5.756296126
H	-4.401335888	-7.665274843	-2.093580849
H	-2.956264129	-6.454589083	-5.953821758
H	-3.065643201	-8.126409922	-4.128335337
Si	-5.922581646	-3.192191349	-3.644840781
C	-6.744126522	-2.597304039	-5.197793700
H	-7.572469425	-3.270734791	-5.448565370
H	-7.152929745	-1.586812259	-5.088240422
H	-6.058280766	-2.602877135	-6.051962485
C	-3.393744378	1.175665976	-3.912635086
H	-3.309644605	0.939864681	-4.978422971
H	-4.397760421	1.573046045	-3.724604008
H	-2.678880439	1.977495395	-3.696881786
C	-6.900801683	-2.961223192	-2.096777774
C	-8.279535237	-2.720805194	-2.178243057
C	-6.304803998	-3.029496612	-0.827208462
C	-9.040989854	-2.562857283	-1.025583701
C	-7.063229415	-2.866489566	0.323415269
C	-8.433047191	-2.634705308	0.222234761
H	-8.771600002	-2.660657766	-3.148552663
H	-5.234433779	-3.223811507	-0.726968628
H	-10.109968722	-2.383038447	-1.102126018
H	-6.590661911	-2.923590052	1.300537621

H -9.028331814 -2.509956529 1.123083550

==============================

10A-TS

==============================

H	-7.961181217	-1.008630249	-3.469679405
C	-7.111652725	-3.203206392	-4.893237499
H	-8.111167284	-3.523246742	-5.205757130
H	-6.405630017	-3.505338248	-5.674917096
H	-6.855791333	-3.754102024	-3.978792321
Si	-7.047156427	-1.355453448	-4.601465179
C	-6.998748927	0.833849992	-1.282678973
H	-6.485743647	1.617942455	-1.850132622
H	-7.238915162	1.258696193	-0.298584038
C	-4.183716723	-0.348183960	-0.555649521
C	-3.534018312	0.844016651	-0.930569847
C	-3.484084159	-1.288644130	0.226627761
C	-2.220567561	1.075339965	-0.556764822
C	-2.173612178	-1.046411699	0.607586822
C	-1.542383915	0.129464839	0.209866830
H	-4.053749102	1.591429310	-1.528848970
H	-3.974837983	-2.202307199	0.556877129
H	-1.721797597	1.992000267	-0.858367360
H	-1.642361645	-1.771677976	1.217493065
H	-0.512545539	0.314016804	0.504807362
Si	-5.909695062	-0.632151000	-1.035599712
C	-5.300872663	-0.876713711	-4.026796688
H	-4.893223845	-1.571014070	-3.275368259
H	-4.593776783	-0.931445439	-4.865034614
H	-5.236897617	0.159535286	-3.663439979
C	-7.490694793	-0.353459296	-6.116873363
H	-7.522024160	0.719716995	-5.899749626
H	-6.758521188	-0.513006315	-6.916664654
H	-8.473587653	-0.638637358	-6.506941034
C	-6.619846474	-2.298675336	-0.954221512
C	-8.010177194	-2.455010252	-0.796701439
C	-5.810860306	-3.447868518	-1.059577280
C	-8.569844381	-3.721291754	-0.725341502
C	-6.377016507	-4.711283093	-1.000279597
C	-7.753843184	-4.845534850	-0.827273771
H	-8.658874617	-1.582648335	-0.731640882
H	-4.736957355	-3.351322331	-1.214768220
H	-9.642419602	-3.835588121	-0.597065026
H	-5.749933236	-5.593616744	-1.093010305
H	-8.195894050	-5.837555132	-0.780440895
H	-7.932413704	0.588591249	-1.798020453

==============================

11A

==============================

C	5.945808531	10.729287614	-1.042995552
H	4.602354457	10.843494898	-3.398651483
C	6.894508743	12.140502395	-3.675908387
H	6.444332095	13.066770633	-3.303291080
H	7.962736290	12.171517692	-3.428686689
H	6.807662575	12.121875232	-4.766743102
Si	6.115422911	10.648963614	-2.889310626
C	6.575069848	8.999405260	-3.606361121
H	6.376830865	8.957502277	-4.683651952
H	7.646344976	8.815431642	-3.462948399
H	6.024995201	8.185781729	-3.119525417
C	2.310261766	11.508823040	-2.252031091
C	2.681318447	12.862139013	-2.295640886
C	1.371099279	11.092864351	-1.297461017
C	2.134659493	13.773390559	-1.403564092
C	0.818648671	12.008441340	-0.409510889
C	1.202978362	13.343865222	-0.460850223
H	3.405767770	13.209326744	-3.035724112
H	1.061085808	10.049524034	-1.251193737

H	2.429479928	14.818733344	-1.441460421
H	0.087515283	11.679039863	0.323930349
H	0.773186130	14.057582363	0.237301250
C	3.098252395	8.557846304	-2.979803964
C	3.236351271	8.183708205	-1.632001667
C	3.104068261	7.556065822	-3.962936880
C	3.387588382	6.848755177	-1.281442179
C	3.248105381	6.220165997	-3.610494690
C	3.395098476	5.869201390	-2.271702935
H	3.221338194	8.943642042	-0.850052042
H	2.993719466	7.818062371	-5.014909011
H	3.493706716	6.568222521	-0.236811963
H	3.246216116	5.451372389	-4.378467999
H	3.512129924	4.824029313	-1.996650016
C	2.793110493	10.675719026	-5.277512666
Si	3.016416683	10.325777691	-3.470197608
H	3.550703800	10.170639670	-5.887101299
H	1.806157734	10.328738727	-5.605833963
H	2.849465671	11.751622427	-5.473445719
H	6.936941659	10.838072948	-0.586314519
H	5.343038546	11.590679778	-0.733686966
H	5.492186642	9.818453196	-0.638490807

===============================

12A-Me3Si+

===============================

C	-3.312073205	-0.300161576	-1.091388748
H	-3.160506183	0.649229544	-0.571447702
H	-4.324198019	-0.668792945	-0.872379114
H	-2.625290462	-1.052259962	-0.679762122
Si	-3.083964106	-0.170878431	-2.901809628
C	-3.214562112	-1.666956893	-3.946446819
H	-3.543306229	-2.548643021	-3.390253109
H	-3.895880258	-1.488492493	-4.788425522
H	-2.233816796	-1.882238427	-4.395385980
C	-2.725097208	1.450859340	-3.668510961
H	-2.534348203	1.381205900	-4.742603089
H	-3.573754596	2.130306377	-3.504269799
H	-1.864806566	1.922366920	-3.175267092

===============================

12A-Ph2MeSiH

===============================

H	4.314883112	11.039400079	-4.077067935
C	2.332314321	11.444415041	-2.258174928
C	3.122021421	12.398432974	-1.605717490
C	1.022573837	11.251299339	-1.796638372
C	2.625362017	13.135304576	-0.534732835
C	0.519819411	11.983081154	-0.727059696
C	1.322379427	12.929014014	-0.095019814
H	4.144796435	12.569740070	-1.942630205
H	0.384139911	10.507339512	-2.277220912
H	3.256687181	13.872161440	-0.042352272
H	-0.499412580	11.816638240	-0.384294828
H	0.931146059	13.504109592	0.741292180
C	3.255780005	8.651722952	-3.159512308
C	3.047147750	8.231127497	-1.839552057
C	3.660681639	7.691302580	-4.099382637
C	3.234656000	6.902150352	-1.471055123
C	3.852248045	6.363417828	-3.738113557
C	3.637775218	5.967113833	-2.419882995
H	2.730848024	8.955158443	-1.086664709
H	3.833773308	7.985297596	-5.137324009
H	3.066533141	6.595872084	-0.439547481
H	4.168621598	5.635219011	-4.484132988
H	3.786307995	4.928284446	-2.132848188
C	1.824184720	10.488591755	-5.148378368
Si	3.007024088	10.438833712	-3.688957028
H	2.193908545	9.884900690	-5.985075423

H	0.844813794	10.087632782	-4.861403239
H	1.678732744	11.512903097	-5.507960205

===============================

13A-Me3SiH

===============================

C	-2.461195359	-0.836356324	-1.265559885
H	-1.923133360	-0.091008810	-0.669483684
H	-3.295797284	-1.207938659	-0.658611958
H	-1.781050487	-1.676185619	-1.445262123
Si	-3.099994222	-0.103414793	-2.876060889
C	-4.000058611	-1.427527580	-3.864015238
H	-4.850818165	-1.817922091	-3.292389838
H	-4.388637016	-1.030090944	-4.808170789
H	-3.343308872	-2.271778987	-4.100872526
H	-1.950526823	0.410793675	-3.674286976
C	-4.279729520	1.312862634	-2.500014822
H	-3.790146591	2.102609904	-1.919575173
H	-4.671626980	1.765392854	-3.417579929
H	-5.134810068	0.951788928	-1.915655251

===============================

13A-Ph2MeSi+

===============================

Si	-3.613646286	0.120272267	-2.823068149
C	-4.661906794	-0.983375538	-3.860597844
H	-5.188282977	-1.737226014	-3.269631946
H	-5.392355316	-0.403422417	-4.435582425
H	-4.029204941	-1.514442713	-4.584036334
C	-2.821901693	1.534672839	-3.619015533
C	-2.343441588	2.634479371	-2.876122209
C	-2.721094530	1.564215105	-5.025846417
C	-1.771948765	3.717903185	-3.521427653
C	-2.137794457	2.645766951	-5.663833947
C	-1.664194786	3.719004144	-4.911147526
H	-2.444697477	2.650538754	-1.792628163
H	-3.093285552	0.733328828	-5.623716816
H	-1.413888991	4.566967395	-2.946310925
H	-2.053396340	2.659201390	-6.746638529
H	-1.212537143	4.570074330	-5.414435310
C	-3.390745626	-0.232475909	-1.065805150
C	-4.325177838	-1.045591995	-0.391063727
C	-2.278828417	0.265921383	-0.353569152
C	-4.165489927	-1.327350364	0.955280133
C	-2.117455891	-0.032656645	0.988505806
C	-3.063139216	-0.821589416	1.641627784
H	-5.188999918	-1.448328374	-0.917336160
H	-1.525949137	0.865924088	-0.861246390
H	-4.894659690	-1.943972444	1.472781566
H	-1.253820484	0.343285921	1.529521671
H	-2.935640258	-1.050538828	2.696588370

===============================

1B

===============================

C	-0.487007532	-0.581726567	-1.631880217
C	0.034075111	-1.792541039	-1.156645453
C	0.089037828	0.608305892	-1.164173904
C	1.093364039	-1.818096549	-0.255036357
C	1.149027193	0.590828130	-0.265268866
C	1.653150868	-0.624872411	0.190433387
H	-0.396679093	-2.737625539	-1.490432054
H	-0.308730293	1.567648557	-1.500576299
H	1.480377941	-2.770771447	0.103760568
H	1.580084558	1.526928574	0.085708638
H	2.479759754	-0.641570775	0.897688786
H	-1.432543450	-0.297292156	-4.240407211
C	-2.877640984	-2.139507513	-2.761106554
H	-2.271223895	-3.002860879	-3.057228472

H	-3.232278772	-2.310095672	-1.737069529
H	-3.753307821	-2.104598049	-3.417772055
Si	-1.916818166	-0.527868952	-2.849932419
C	-3.021746565	0.921848927	-2.409018128
C	-3.407497315	1.869530537	-3.364079635
C	-3.498799759	1.075284506	-1.099486101
C	-4.242846477	2.931191843	-3.028687132
C	-4.334017958	2.131603305	-0.757263053
C	-4.707202528	3.061889824	-1.724496331
H	-3.045175021	1.778720251	-4.388807819
H	-3.200472005	0.360425096	-0.329441126
H	-4.529398108	3.659380818	-3.785412559
H	-4.692364702	2.234532592	0.265234605
H	-5.358464416	3.892011989	-1.458773834

==============================

2B

==============================

C	-1.2358184993	2.8052108999	-3.0531558142
C	-0.7081322697	2.1002633615	-1.9716002483
C	-1.8183612395	2.1288095808	-4.1249421025
C	-0.7478011253	0.7186271734	-1.9639590926
C	-1.8890457396	0.7484977673	-4.1125458585
C	-1.3457923037	0.0147867822	-3.0340353434
C	-4.7004289516	-3.4040119070	-4.8002941132
C	-4.8884181138	-2.1736200689	-4.1709085577
C	-3.4348528734	-3.9876872987	-4.8531104367
C	-3.8113753066	-1.5165458579	-3.6059921403
C	-2.3573845089	-3.3523830126	-4.2642215366
C	-2.5243858942	-2.0984878039	-3.6336998443
C	-1.4050941680	-1.4247456909	-3.0253484838
C	1.7008466563	-3.6623832360	-1.2130341556
C	2.0000942144	-2.4663833258	-1.8650461895
C	-0.6277164051	-3.4091622181	-1.7556112220
C	0.9873163198	-1.7251986090	-2.4451830984
C	-0.3481536745	-2.1872307394	-2.4093082776
H	-3.9602838742	-0.5733254895	-3.0861761919
H	-1.3642774538	-3.7893174049	-4.3316163933
H	-3.2934986205	-4.9376373475	-5.3604024678
H	-5.5477344493	-3.9128807378	-5.2528872010
H	-5.8804755217	-1.7348094862	-4.1170693935
H	1.2216338552	-0.8095877788	-2.9822483722
H	3.0278918358	-2.1202756017	-1.9239048573
C	0.3876410652	-4.1282949807	-1.1525475300
H	-2.3132300998	0.2164104928	-4.9605330240
H	-0.3712064852	0.1671491698	-1.1060890922
H	-0.2769946396	2.6355936431	-1.1306775264
H	-1.1926253646	3.8912824406	-3.0611495354
H	-2.2098388718	2.6843061763	-4.9721674564
H	2.4982742758	-4.2367161350	-0.7483387911
H	0.1602864623	-5.0505821352	-0.6259034010
H	-1.6564258288	-3.7522217038	-1.6804146358

==============================

3B

==============================

C	6.539462024	10.623910130	-2.838700807
C	5.491037229	9.728601882	-2.588369288
C	6.843525104	11.569347161	-1.848070924
C	4.763918948	9.776552198	-1.404171510
C	6.116936779	11.628605733	-0.663133838
C	5.073293844	10.733973397	-0.441780195
H	5.244186058	8.966996687	-3.332505733
H	7.664226739	12.271034843	-2.002390468
H	3.956824937	9.067402473	-1.229307641
H	6.370396184	12.368137241	0.093677417
H	4.509629183	10.775893337	0.487548237
H	6.624617807	9.815714750	-5.412905038
Si	7.537675381	10.474094631	-4.418725056

C	9.054427176	9.403287943	-4.170496510
H	9.596613844	9.218986355	-5.104613286
H	9.744987519	9.885434494	-3.468809176
H	8.774638734	8.431403449	-3.748923914
C	4.125060366	10.743916319	-6.648223337
C	3.439084906	11.060586730	-5.423769778
C	3.909954752	12.116352324	-4.610610300
C	2.314597509	10.320649735	-4.987987301
C	3.297762278	12.408175416	-3.406380577
C	1.694127538	10.634210827	-3.793777482
C	2.189674884	11.669328050	-2.997799974
H	4.799376720	12.662435791	-4.912823438
H	1.905905959	9.540613180	-5.624540025
H	3.699359289	13.193232849	-2.770710437
H	0.814200185	10.080671620	-3.478327133
H	1.707430939	11.899127301	-2.050571664
C	4.136693489	9.393744149	-7.157114462
C	4.129456741	8.284781578	-6.283523629
C	4.156924429	9.161632189	-8.549478419
C	4.144838411	6.996786670	-6.785864889
C	4.144832361	7.869688120	-9.043935051
C	4.144023147	6.788182413	-8.164747888
H	4.165775987	8.454153013	-5.210025306
H	4.125961167	10.006236026	-9.232074854
H	4.166374061	6.149812912	-6.106079519
H	4.129001098	7.699947012	-10.116701921
H	4.144486351	5.774178850	-8.556745256
C	4.822675917	11.779927636	-7.371025904
C	6.053544964	11.496312218	-8.003805911
C	4.316426747	13.097505218	-7.423433293
C	6.762300300	12.503720782	-8.632842254
C	5.015087954	14.089466634	-8.085265346
C	6.245213038	13.797517928	-8.674838481
H	6.472785977	10.495822228	-7.930372884
H	3.348982664	13.315345491	-6.978508402
H	7.729737419	12.288886181	-9.077921911
H	4.606131954	15.094209206	-8.144810039
H	6.805248398	14.585483776	-9.172623928
C	8.035939060	12.166910283	-5.070657009
C	9.104944500	12.278933882	-5.972187945
C	7.366407442	13.345245677	-4.708381751
C	9.482907130	13.510222945	-6.497951390
C	7.731306591	14.580112704	-5.236554932
C	8.791358804	14.663103185	-6.134869640
H	9.664518563	11.388689135	-6.264424265
H	6.566499515	13.303134335	-3.966667804
H	10.325093494	13.573330945	-7.184570553
H	7.201154325	15.480881305	-4.932564325
H	9.090075394	15.628580047	-6.537714987

==============================

3B-TS

==============================

C	6.398637932	10.767669567	-2.847700301
C	5.517020243	9.779360359	-2.382488246
C	6.653910902	11.873015114	-2.023585659
C	4.905326019	9.891833393	-1.141852271
C	6.048104217	11.985440249	-0.777618644
C	5.170420971	10.998722231	-0.339569836
H	5.302690830	8.903061155	-2.997331179
H	7.343525930	12.648811043	-2.354939392
H	4.223885242	9.118073536	-0.796779032
H	6.264739071	12.842018893	-0.144030046
H	4.696121094	11.088343621	0.634752852
H	5.821826546	10.585389881	-5.446871770
Si	7.120638227	10.612940103	-4.551027749
C	7.966431848	8.992652297	-4.928720938
H	8.079108532	8.843682261	-6.009316072
H	8.964798254	8.970413559	-4.476244655

H	7.403633668	8.143692186	-4.526565558
C	4.480990856	10.732667380	-6.347141136
C	3.541941332	10.953692064	-5.241228969
C	3.721532418	12.051051415	-4.378553431
C	2.463112107	10.084292648	-5.008062722
C	2.860133108	12.269871711	-3.319309078
C	1.590347352	10.318123338	-3.955501762
C	1.790622123	11.401712342	-3.106320725
H	4.565183331	12.719718596	-4.538938449
H	2.285750140	9.249291927	-5.679104273
H	3.027732568	13.108965051	-2.649722482
H	0.745928107	9.652411084	-3.799934002
H	1.109977912	11.573478564	-2.276427838
C	4.535268066	9.397919325	-6.969699489
C	4.621607374	8.244542980	-6.172560525
C	4.464683307	9.251841009	-8.361958556
C	4.644780158	6.987013213	-6.747585552
C	4.464703703	7.986872573	-8.935465897
C	4.560976767	6.856098313	-8.134037534
H	4.692266854	8.353148705	-5.090718831
H	4.373891834	10.131324821	-8.992946187
H	4.729700845	6.104133960	-6.119896608
H	4.389106812	7.886596557	-10.014635461
H	4.572039042	5.868189339	-8.586889749
C	4.923245171	11.881784546	-7.149592139
C	6.133723333	11.811355142	-7.862017483
C	4.155129050	13.052761008	-7.237665256
C	6.568611210	12.878669152	-8.625922823
C	4.585161204	14.114132327	-8.022228665
C	5.791929962	14.033631419	-8.708387936
H	6.744457021	10.912332959	-7.787480005
H	3.200356991	13.113357409	-6.721923086
H	7.518598837	12.818008806	-9.149785949
H	3.971062660	15.007000780	-8.101216153
H	6.129322250	14.871834349	-9.312626017
C	8.036904960	12.106629017	-5.165770343
C	9.242576237	11.960862760	-5.868021097
C	7.502341006	13.398663155	-5.032675575
C	9.895788420	13.063772220	-6.406145078
C	8.146841420	14.501753791	-5.576892285
C	9.346461355	14.334661081	-6.264251808
H	9.684275229	10.972508100	-5.994339138
H	6.568689713	13.551323768	-4.489356683
H	10.836412328	12.932834545	-6.935403901
H	7.716636434	15.493858142	-5.463183853
H	9.855994504	15.197223280	-6.686534895

==============================

4B

==============================

C	6.778917590	10.588672893	-2.806372595
C	6.088983959	9.444912195	-2.363831015
C	6.802108748	11.734883325	-1.989830947
C	5.415603420	9.458384006	-1.152595444
C	6.134610317	11.739864726	-0.774540273
C	5.435085451	10.606965890	-0.363928104
H	6.069927881	8.543261036	-2.976756242
H	7.347790895	12.622047129	-2.308369147
H	4.873414830	8.577842038	-0.819627855
H	6.158056231	12.624617431	-0.144154668
H	4.904222193	10.616894207	0.584740370
H	5.569294523	10.706131747	-5.385072005
Si	7.547414656	10.605749329	-4.449518033
C	7.970901440	9.006777182	-5.260673750
H	8.657892390	9.146535627	-6.100940516
H	8.412629972	8.304376523	-4.545415182
H	7.051225188	8.545650879	-5.656275594
C	4.689887734	10.768554627	-6.099644156
C	3.493375709	11.088244995	-5.227924766

C	3.642982346	11.763277264	-4.017579906
C	2.206911385	10.774370205	-5.671598196
C	2.532440850	12.118159467	-3.259698128
C	1.095880916	11.138538141	-4.921589411
C	1.255193747	11.809214649	-3.712540746
H	4.640285200	12.011329800	-3.653212729
H	2.079215865	10.248271113	-6.616737558
H	2.667809771	12.639003951	-2.314407266
H	0.099664190	10.894740573	-5.282601011
H	0.384170934	12.088617611	-3.125382882
C	4.572319506	9.386732867	-6.713651718
C	4.513382085	8.287827853	-5.847528670
C	4.448095256	9.166714975	-8.084018464
C	4.361121845	6.998612048	-6.337942886
C	4.284673704	7.874166444	-8.576533617
C	4.248374291	6.789373144	-7.710458062
H	4.551322637	8.463783792	-4.770005243
H	4.468817782	10.008518005	-8.772395060
H	4.314530360	6.156770196	-5.651320296
H	4.180672834	7.718624655	-9.647514874
H	4.122139162	5.782717724	-8.100297178
C	5.044997862	11.871392937	-7.073181882
C	6.213738459	11.755151272	-7.835678222
C	4.261950163	13.015577834	-7.224557321
C	6.586470107	12.747094803	-8.730253082
C	4.631865073	14.010480630	-8.126312107
C	5.791585082	13.881631310	-8.879087041
H	6.830596475	10.859703778	-7.737583723
H	3.350115570	13.128978661	-6.642436000
H	7.496195781	12.635127227	-9.315810927
H	4.002042244	14.889141918	-8.242303850
H	6.074683921	14.657915075	-9.585673064
C	8.097156755	12.176632441	-5.174832510
C	9.252571481	12.209956846	-5.977580162
C	7.346703211	13.356662635	-5.007142893
C	9.660061528	13.395011857	-6.569836982
C	7.743800867	14.531977597	-5.624319561
C	8.901194090	14.550725088	-6.398760836
H	9.843401641	11.307650939	-6.132500001
H	6.425643757	13.346885465	-4.424443404
H	10.562707734	13.419218915	-7.174176342
H	7.146071109	15.432371498	-5.512671591
H	9.212305772	15.474830425	-6.879569755

==============================

5B

===============================

C	-1.217728800	-0.257537446	-2.609031636
C	-2.724579435	-0.093927238	-2.661913184
C	-3.363573476	1.088812496	-2.293607027
C	-3.506424753	-1.186369538	-3.043596638
C	-4.752862397	1.177125718	-2.308425837
C	-4.891905230	-1.100590023	-3.062640422
C	-5.521010416	0.085391138	-2.693819586
H	-2.767779125	1.950146467	-1.997185489
H	-3.012217445	-2.118044229	-3.320688347
H	-5.235480273	2.108095764	-2.018713713
H	-5.483876410	-1.960922554	-3.367219046
H	-6.606124637	0.157113521	-2.709111604
C	-0.761126330	-1.041278525	-1.392980885
C	-1.498878149	-1.089403520	-0.211395857
C	0.473961231	-1.691480068	-1.439399777
C	-1.011349172	-1.773806722	0.898720315
C	0.961214874	-2.377198896	-0.335291844
C	0.217325559	-2.419986040	0.840756102
H	-2.464662193	-0.589013210	-0.161205719
H	1.060572491	-1.645342834	-2.357420188
H	-1.598988876	-1.801987445	1.813817118
H	1.924052542	-2.880430932	-0.391242571

H	0.595370584	-2.956705719	1.707872582
C	-0.485789552	1.063530717	-2.755068315
C	0.069552908	1.738001669	-1.668841867
C	-0.410932886	1.653500494	-4.018841123
C	0.687619430	2.973178650	-1.843944707
C	0.207383983	2.883537587	-4.197112319
C	0.760402066	3.548863170	-3.106376834
H	0.022173975	1.289761941	-0.677737830
H	-0.855310412	1.136561210	-4.869936338
H	1.116879189	3.486009178	-0.985884258
H	0.259443957	3.325196793	-5.189937396
H	1.247263445	4.511901903	-3.242223667
H	-0.941480347	-0.862491837	-3.488754875

==============================

6B

==============================

Si	-3.613646286	0.120272267	-2.823068149
C	-4.661906794	-0.983375538	-3.860597844
H	-5.188282977	-1.737226014	-3.269631946
H	-5.392355316	-0.403422417	-4.435582425
H	-4.029204941	-1.514442713	-4.584036334
C	-2.821901693	1.534672839	-3.619015533
C	-2.343441588	2.634479371	-2.876122209
C	-2.721094530	1.564215105	-5.025846417
C	-1.771948765	3.717903185	-3.521427653
C	-2.137794457	2.645766951	-5.663833947
C	-1.664194786	3.719004144	-4.911147526
H	-2.444697477	2.650538754	-1.792628163
H	-3.093285552	0.733328828	-5.623716816
H	-1.413888991	4.566967395	-2.946310925
H	-2.053396340	2.659201390	-6.746638529
H	-1.212537143	4.570074330	-5.414435310
C	-3.390745626	-0.232475909	-1.065805150
C	-4.325177838	-1.045591995	-0.391063727
C	-2.278828417	0.265921383	-0.353569152
C	-4.165489927	-1.327350364	0.955280133
C	-2.117455891	-0.032656645	0.988505806
C	-3.063139216	-0.821589416	1.641627784
H	-5.188999918	-1.448328374	-0.917336160
H	-1.525949137	0.865924088	-0.861246390
H	-4.894659690	-1.943972444	1.472781566
H	-1.253820484	0.343285921	1.529521671
H	-2.935640258	-1.050538828	2.696588370

==============================

7B

==============================

C	-2.618746801	-1.725024590	-0.975602941
C	-3.979747591	-1.929436901	-0.707308581
C	-1.679262122	-1.992616963	0.032620653
C	-4.389279015	-2.400697341	0.535866835
C	-2.089591893	-2.456909089	1.275086897
C	-3.446146130	-2.662089825	1.523809679
H	-4.723750413	-1.722714466	-1.475989671
H	-0.613913916	-1.836161257	-0.154705569
H	-5.446159115	-2.562871593	0.734695389
H	-1.356570189	-2.661289772	2.051263772
H	-3.768638308	-3.027305168	2.496537987
H	-1.186331584	0.150061203	-2.061163059
C	-0.695590957	-2.047630249	-3.483399912
H	0.112687172	-2.297450632	-2.785797553
H	-1.078367167	-2.990528263	-3.891677473
H	-0.275075709	-1.458574351	-4.308871690
Si	-2.051157491	-1.115401231	-2.621315463
C	0.051424382	1.575990540	-4.040851739
C	1.375597841	1.309817291	-4.426209594
C	-0.923026348	1.740989479	-5.040353905
C	1.717190872	1.211672047	-5.769953156

C	-0.581035117	1.632027151	-6.382801192
C	0.737449085	1.368236743	-6.746226942
H	2.150902035	1.179407700	-3.671408874
H	-1.961593305	1.937735996	-4.769389728
H	2.746688262	1.011671082	-6.057589146
H	-1.345217732	1.753433559	-7.146916441
H	1.004351912	1.285782038	-7.797807616
C	0.974548435	1.219784896	-1.060533089
H	0.584511496	1.120667183	-0.041448021
H	1.501948055	0.294639920	-1.321310008
H	1.702802833	2.039106739	-1.060641658
Si	-0.400776240	1.571259367	-2.257977940
C	-1.754887824	2.694256557	-1.713963564
C	-2.403389044	2.446824571	-0.493038867
C	-2.182108628	3.768901016	-2.505364358
C	-3.458789907	3.249211639	-0.080019231
C	-3.236534598	4.573775393	-2.089055185
C	-3.875518936	4.310718773	-0.881526361
H	-2.091319550	1.609740501	0.135745879
H	-1.686842384	3.980874338	-3.452467169
H	-3.957389780	3.051162034	0.865569045
H	-3.559525946	5.408127699	-2.706841713
H	-4.701316302	4.940051923	-0.558092086
C	-3.326669225	-0.349043409	-3.705777605
C	-3.396425043	-0.695558002	-5.063488344
C	-4.212421328	0.622463861	-3.209192769
C	-4.328050567	-0.094328809	-5.901082263
C	-5.130693067	1.236550837	-4.052071816
C	-5.190136624	0.875552968	-5.396381012
H	-2.712367371	-1.437646104	-5.475472729
H	-4.171674369	0.920273302	-2.159878037
H	-4.380257941	-0.379102432	-6.948971713
H	-5.802347392	1.996274544	-3.659702279
H	-5.913811820	1.351321426	-6.053680779

==============================

7B-TS

==============================

C	-0.723443380	-2.701656008	-2.874068404
C	-0.019184162	-2.915312489	-4.070292866
C	-0.580683066	-3.647505116	-1.847140888
C	0.813168746	-4.018401660	-4.227779319
C	0.244363533	-4.756106935	-2.002759098
C	0.947319227	-4.937355704	-3.191310668
H	-0.149533779	-2.218363629	-4.900804023
H	-1.134852193	-3.526750996	-0.914809983
H	1.348599016	-4.169740147	-5.162767524
H	0.334518704	-5.486053504	-1.200781748
H	1.593559789	-5.804034213	-3.313304646
H	-0.861856211	-0.095583515	-2.137249658
Si	-1.790321718	-1.174477811	-2.649998246
C	-3.108256661	-1.451808243	-1.355216569
H	-3.753336995	-0.572229315	-1.261075061
H	-3.738270557	-2.303292956	-1.638385553
H	-2.680783442	-1.663577722	-0.367989336
C	2.633063275	-1.650934866	-2.147507550
C	2.763012230	-2.629970698	-1.144715569
C	3.529961143	-1.667685330	-3.234517823
C	3.757129472	-3.593227483	-1.227031717
C	4.519628084	-2.634323840	-3.313383687
C	4.631638548	-3.595218929	-2.311036488
H	2.080951488	-2.640483263	-0.295238920
H	3.468445643	-0.903276437	-4.006261463
H	3.853332935	-4.343549129	-0.446979751
H	5.214248320	-2.633922545	-4.149277794
H	5.413792492	-4.348251163	-2.372001940
C	0.942843104	0.077841076	-0.192776552
H	0.289429333	0.952897150	-0.135179827
H	0.387814764	-0.781694178	0.204999455

H	1.819169896	0.230063150	0.448791277
Si	1.475698723	-0.268931518	-1.927389565
C	1.363179102	1.002972903	-3.229707302
C	1.211114571	2.352911676	-2.867142589
C	1.386038772	0.659388338	-4.593552606
C	1.121208719	3.334384826	-3.844003180
H	1.175592869	2.643926991	-1.817306834
C	1.274378142	1.642036370	-5.566024188
H	1.465605489	-0.385665656	-4.894366377
C	1.150628062	2.977812801	-5.190494458
H	1.022585576	4.378416220	-3.557541105
H	1.277881575	1.367674926	-6.617799505
H	1.070304618	3.747723867	-5.953801545
C	-2.380217981	-0.531468782	-4.292430217
C	-2.259486585	0.826289177	-4.614133551
C	-2.974221660	-1.390629109	-5.228871526
C	-2.721071604	1.313595732	-5.831764157
H	-1.790939393	1.513054485	-3.905941901
C	-3.436471200	-0.906788144	-6.446485342
H	-3.068331971	-2.455027502	-5.009110513
C	-3.310231466	0.446540095	-6.746921433
H	-2.620045713	2.370625708	-6.068098964
H	-3.895017102	-1.584106122	-7.163561029
H	-3.672251946	0.825438656	-7.699793333

==============================

8B

==============================

C	-0.198128402	-1.884293037	-2.874100740
C	0.228097435	-2.094601100	-4.223602291
C	-0.387240667	-3.062911310	-2.086447336
C	0.477748017	-3.356730043	-4.727517531
C	-0.126785276	-4.326657844	-2.581387935
C	0.309630853	-4.469197345	-3.900123386
H	0.355341096	-1.226254870	-4.872965543
H	-0.750469600	-2.947695552	-1.064633667
H	0.803911520	-3.487895582	-5.755833503
H	-0.262532133	-5.203605352	-1.954714825
H	0.515513463	-5.463501568	-4.291973463
H	-1.331250052	0.644602283	-3.169562778
C	-2.895760080	-1.473807295	-4.157392729
H	-2.491363946	-1.327449293	-5.164511626
H	-3.002003803	-2.554026249	-3.990582370
H	-3.897318650	-1.031741000	-4.123242829
Si	-1.811563055	-0.717438339	-2.836268098
C	2.757421593	-1.980204668	-1.902516478
C	3.217743982	-2.474412239	-0.671900749
C	3.456346200	-2.342806867	-3.066421509
C	4.326622437	-3.310546055	-0.608295275
C	4.565747655	-3.175961233	-3.005716032
C	4.999374075	-3.663457552	-1.774486716
H	2.721104521	-2.184593709	0.254420338
H	3.141667810	-1.962386682	-4.040518411
H	4.672576326	-3.677747723	0.356158416
H	5.100559879	-3.438135660	-3.917008733
H	5.871078382	-4.311331336	-1.724102123
Si	1.321518322	-0.798500432	-1.990996725
C	1.698952083	0.619282314	-3.145193038
H	2.120241284	0.272827552	-4.095877202
H	0.840897500	1.265822170	-3.359847093
H	2.473027979	1.233607220	-2.667067716
C	0.732309400	-0.233081032	-0.321814075
C	0.393516104	-1.113574935	0.719352002
C	0.703107953	1.143747826	-0.055935615
C	0.038923032	-0.637677351	1.973568295
C	0.359587615	1.623835563	1.203771849
C	0.026387186	0.733592067	2.217392058
H	0.418859527	-2.192107002	0.560238636
H	0.958236401	1.859113245	-0.837442491

H	-0.222505441	-1.335413430	2.765111094
H	0.354777260	2.694578069	1.392960345
H	-0.242017356	1.107324208	3.202582182
C	-2.618480718	-0.743583487	-1.170189228
C	-3.259840154	-1.895113238	-0.689049392
C	-2.671723989	0.423611347	-0.397690335
C	-3.910687599	-1.887279380	0.537762616
C	-3.330938895	0.436667386	0.826187808
C	-3.944021386	-0.719621408	1.296115457
H	-3.262806100	-2.812007380	-1.282242098
H	-2.187596768	1.333645060	-0.751765307
H	-4.402974296	-2.786818336	0.899570529
H	-3.363684930	1.350816546	1.414612934
H	-4.458711157	-0.711029287	2.254018075

==============================

8B-TS

==============================

C	-0.732801000	-2.012800000	-3.150117000
C	-0.394456000	-2.264624000	-4.499615000
C	-0.206182000	-2.909531000	-2.185727000
C	0.377481000	-3.355285000	-4.861690000
C	0.547042000	-4.040356000	-2.562209000
C	0.843391000	-4.256044000	-3.892314000
H	-0.770212000	-1.600901000	-5.277619000
H	-0.525820000	-2.824314000	-1.145194000
H	0.607576000	-3.528240000	-5.910555000
H	0.908914000	-4.722539000	-1.796990000
H	1.428213000	-5.121806000	-4.190820000
Si	-2.128081000	-0.767248000	-2.719352000
Si	1.365893000	-1.057227000	-2.055069000
C	1.577389000	-1.291781000	-0.228557000
H	0.754113000	0.219589000	-2.461242000
C	-1.883450000	-0.228272000	-0.941968000
C	-1.293474000	0.999093000	-0.604407000
C	-2.273378000	-1.081870000	0.102967000
C	-1.078500000	1.352413000	0.724548000
H	-1.004102000	1.699242000	-1.388900000
C	-2.053405000	-0.737179000	1.433200000
H	-2.776071000	-2.023055000	-0.127963000
C	-1.451618000	0.480128000	1.743855000
H	-0.629126000	2.312593000	0.966498000
H	-2.365789000	-1.409209000	2.228879000
H	-1.286858000	0.755546000	2.782661000
C	-3.710475000	-1.741038000	-2.902731000
C	-4.928943000	-1.051935000	-2.778616000
C	-3.747151000	-3.124420000	-3.129572000
C	-6.141831000	-1.721760000	-2.880493000
H	-4.932077000	0.025086000	-2.594952000
C	-4.960278000	-3.798914000	-3.228710000
H	-2.819771000	-3.692532000	-3.236011000
C	-6.156590000	-3.097268000	-3.105260000
H	-7.076821000	-1.172789000	-2.784015000
H	-4.972503000	-4.872799000	-3.404784000
H	-7.105079000	-3.622853000	-3.184563000
C	-2.037418000	0.665871000	-3.919907000
H	-2.722656000	1.463098000	-3.607854000
H	-1.034680000	1.104275000	-3.996319000
H	-2.348606000	0.349381000	-4.921921000
C	2.736821000	-1.536422000	-3.167849000
C	2.864149000	-0.895592000	-4.411161000
C	3.695470000	-2.492773000	-2.794762000
C	3.918562000	-1.205490000	-5.260243000
H	2.132309000	-0.145869000	-4.715643000
C	4.751046000	-2.799379000	-3.641794000
H	3.617894000	-3.001043000	-1.833153000
C	4.858832000	-2.157317000	-4.874270000
H	4.012990000	-0.703114000	-6.219270000
H	5.493580000	-3.534586000	-3.343341000

H	5.686726000	-2.397761000	-5.536563000
H	2.342672000	-0.586251000	0.118946000
H	0.647550000	-1.074704000	0.310558000
H	1.909362000	-2.305808000	0.018211000

==============================

9B

==============================

C	-3.467549452	-1.017509046	-2.635754730
C	-3.742531831	-0.070349192	-3.622080735
C	-2.850986286	-2.238362459	-3.059647040
C	-3.368936032	-0.275323232	-4.949131586
C	-2.415005226	-2.404145018	-4.407056578
C	-2.699950119	-1.437891207	-5.348614801
H	-4.212747496	0.873256408	-3.345860427
H	-2.350592179	-2.842159816	-2.287509581
H	-3.587451096	0.492499432	-5.688540512
H	-1.891009916	-3.317426671	-4.684014160
H	-2.390945587	-1.564132979	-6.382220114
H	-4.839914775	-3.454666821	-1.613391993
C	-5.769777271	-3.020427834	-4.235759815
H	-6.206276225	-2.059502991	-3.943282824
H	-5.388533371	-2.932583899	-5.259312751
H	-6.573968312	-3.766958071	-4.243920113
Si	-4.470109457	-3.583378354	-3.036312007
C	-3.311691864	1.100388365	-0.476612989
C	-2.041693228	1.360637277	0.056652639
C	-4.133089797	2.194545568	-0.790505355
C	-1.601957451	2.664618887	0.254974131
C	-3.696333940	3.499371226	-0.593461247
C	-2.426889983	3.735362638	-0.074126913
H	-1.390588109	0.534144002	0.340631926
H	-5.139476758	2.032308009	-1.179959591
H	-0.614978101	2.845581271	0.674004535
H	-4.350194065	4.333423228	-0.836824327
H	-2.084224447	4.754990928	0.083168786
Si	-3.906366133	-0.640026519	-0.795627499
C	-5.773114467	-0.730724359	-0.644548593
H	-6.258035080	-0.095727035	-1.396375149
H	-6.163370947	-1.746964694	-0.768805577
H	-6.089189830	-0.366646330	0.340389687
C	-3.024537219	-1.942660655	0.212077592
C	-1.643247889	-2.157509725	0.067243615
C	-3.722716025	-2.754858641	1.115659157
C	-0.986856593	-3.145472197	0.794123057
C	-3.070556691	-3.744452846	1.845821224
C	-1.703013805	-3.940941729	1.684430065
H	-1.064442712	-1.543379007	-0.626633898
H	-4.794534123	-2.615004417	1.256428851
H	0.083287328	-3.292736262	0.669678753
H	-3.630176241	-4.361679952	2.544699721
H	-1.192350598	-4.712619655	2.256177197
C	-3.679920515	-5.215258591	-3.369357410
C	-3.728407140	-5.817454959	-4.635432505
C	-3.014194990	-5.876224121	-2.325307640
C	-3.131595898	-7.053837598	-4.851017587
C	-2.413638271	-7.110866234	-2.543883694
C	-2.474114012	-7.698354382	-3.805361616
H	-4.242455365	-5.322181670	-5.461028781
H	-2.970363827	-5.425110236	-1.330323449
H	-3.180818866	-7.518414924	-5.833081581
H	-1.902872140	-7.619838395	-1.730028787
H	-2.007987663	-8.666186075	-3.974982254

==============================
9B-TS
==============================

```
 H   -8.357692000  -1.739665000  -4.492647000
 Si  -7.330507000  -2.674640000  -4.976432000
 C   -4.199706000  -0.388089000  -0.810750000
 C   -3.330375000   0.657729000  -1.158605000
 C   -3.849629000  -1.208101000   0.269781000
 C   -2.149767000   0.871842000  -0.457323000
 C   -2.668323000  -0.997706000   0.974173000
 C   -1.817762000   0.040642000   0.609512000
 H   -3.583355000   1.326160000  -1.983798000
 H   -4.508668000  -2.024084000   0.566528000
 H   -1.488502000   1.687506000  -0.739966000
 H   -2.411786000  -1.643165000   1.810803000
 H   -0.894057000   0.205986000   1.158869000
 Si  -5.808613000  -0.604020000  -1.730841000
 C   -5.495154000  -0.618539000  -3.604054000
 H   -4.760450000   0.112939000  -3.960757000
 H   -5.110346000  -1.612118000  -3.890623000
 H   -6.438133000  -0.381591000  -4.119958000
 C   -6.544223000  -2.304744000  -1.373849000
 C   -7.820122000  -2.471760000  -0.814205000
 C   -5.765168000  -3.457433000  -1.590757000
 C   -8.296797000  -3.735538000  -0.478336000
 C   -6.232742000  -4.721789000  -1.250894000
 C   -7.502154000  -4.859836000  -0.694585000
 H   -8.437005000  -1.595232000  -0.615089000
 H   -4.756255000  -3.358103000  -1.998063000
 H   -9.281436000  -3.843880000  -0.028320000
 H   -5.610344000  -5.599239000  -1.414246000
 H   -7.868803000  -5.846016000  -0.417008000
 C   -6.282736000  -2.250276000  -6.429993000
 C   -7.030197000   0.751520000  -1.323690000
 C   -8.250594000   0.856827000  -2.009065000
 C   -6.762366000   1.685674000  -0.314423000
 C   -9.170688000   1.850674000  -1.699312000
 H   -8.498159000   0.142642000  -2.799171000
 C   -7.678326000   2.685954000  -0.001355000
 H   -5.822032000   1.631882000   0.233798000
 C   -8.882281000   2.768391000  -0.692087000
 H  -10.111703000   1.913904000  -2.241014000
 H   -7.451418000   3.402342000   0.784414000
 H   -9.598456000   3.549214000  -0.448008000
 H   -5.272892000  -2.663826000  -6.334282000
 H   -6.739100000  -2.697821000  -7.323337000
 H   -6.205223000  -1.170054000  -6.576525000
 C   -7.536984000  -4.385938000  -4.481396000
 C   -6.662813000  -5.400838000  -4.930816000
 C   -8.680852000  -4.735258000  -3.732018000
 C   -6.937589000  -6.725516000  -4.646638000
 H   -5.772594000  -5.148018000  -5.505212000
 C   -8.962622000  -6.069851000  -3.477208000
 H   -9.350959000  -3.961326000  -3.359914000
 C   -8.090749000  -7.056999000  -3.928147000
 H   -6.267266000  -7.508147000  -4.991087000
 H   -9.853139000  -6.339840000  -2.915927000
 H   -8.308684000  -8.101802000  -3.719610000
```

==============================
10B
==============================

```
 C   -1.946437127   0.493562115  -1.504660238
 C   -0.770423924   1.249963295  -1.408289940
 C   -2.165774717  -0.264420085  -2.666671955
 C    0.158049553   1.248877277  -2.443626134
 C   -1.240345681  -0.264814522  -3.700113779
 C   -0.077727146   0.494938077  -3.586829950
 H   -0.575341009   1.840514843  -0.514082219
```

H	-3.062389865	-0.882509570	-2.764357539
H	1.067862548	1.836817510	-2.356249621
H	-1.419108139	-0.858094890	-4.593170687
H	0.648485019	0.494938941	-4.395758734
H	-7.566608682	2.031445001	-1.452929855
C	-6.375060189	1.095699109	-3.830845526
H	-5.922830733	2.064240228	-4.069718242
H	-5.687375700	0.308856984	-4.165021675
H	-7.294497803	1.000969441	-4.418152330
Si	-6.742974335	0.940483847	-2.013770667
C	-2.912830838	1.761710537	1.188819246
C	-2.877267138	1.386930936	2.539934731
C	-2.727465779	3.112981765	0.856023504
C	-2.658716478	2.337617641	3.530366369
C	-2.513144907	4.061197924	1.845649470
C	-2.478977600	3.671609964	3.183000876
H	-3.011894197	0.342538424	2.819580942
H	-2.737663531	3.430570741	-0.188020150
H	-2.625868272	2.035879608	4.573932519
H	-2.368198856	5.104630030	1.578148851
H	-2.308209809	4.414572749	3.957964825
Si	-3.151715320	0.474262938	-0.106141521
C	-4.971973164	1.103378102	-1.109506016
H	-4.539720826	2.040706176	-1.463112110
H	-4.671129155	0.174892889	-1.606278513
H	-5.372301001	1.062960974	-0.093026176
C	-3.657300095	-1.197127728	0.497993168
C	-2.927206515	-2.331275338	0.114361256
C	-4.761378759	-1.362959188	1.350062662
C	-3.288410245	-3.593779356	0.572864894
C	-5.123824376	-2.622912347	1.803436319
C	-4.384017456	-3.738251924	1.415931005
H	-2.064965335	-2.227349026	-0.543534296
H	-5.348780786	-0.500201599	1.671798448
H	-2.710082828	-4.464593614	0.274967645
H	-5.983119125	-2.738105936	2.459321411
H	-4.664214152	-4.725218659	1.775917696
C	-7.315890558	-0.722425189	-1.429030559
C	-6.643219729	-1.886495225	-1.831242104
C	-8.359990120	-0.838484763	-0.503236545
C	-7.002627223	-3.128520260	-1.325263607
C	-8.725288035	-2.082053546	0.002491014
C	-8.047238306	-3.224264919	-0.408392801
H	-5.825908722	-1.830825072	-2.555661174
H	-8.900178973	0.050152413	-0.176371146
H	-6.472927872	-4.022604426	-1.645543695
H	-9.543490346	-2.159810020	0.714311881
H	-8.334277791	-4.196334365	-0.014062820

==============================

10B-TS

==============================

H	-7.911236719	-0.893994077	-3.478811011
C	-7.469086663	-3.244572745	-4.847941880
H	-8.495932518	-3.372731810	-5.205319871
H	-6.798845794	-3.698352912	-5.587544195
H	-7.361937505	-3.807577559	-3.911370824
Si	-7.063696850	-1.435327849	-4.583908150
C	-4.195358868	-0.804575627	-0.581863635
C	-3.338433260	0.164708473	-1.138333736
C	-3.640368819	-1.837519680	0.197575630
C	-1.970112149	0.092664557	-0.931535603
C	-2.271492454	-1.895513129	0.413225455
C	-1.439058352	-0.935760669	-0.155828566
H	-3.744872757	0.969863435	-1.749555767
H	-4.284331263	-2.588406330	0.652896963
H	-1.313531181	0.836329871	-1.374500077
H	-1.851283429	-2.688896738	1.025104285
H	-0.365705697	-0.988071148	0.007767047

Si	-5.991095090	-0.725447440	-0.863457932
C	-5.254127391	-1.254460024	-4.069617896
H	-4.954537897	-1.926794332	-3.253404328
H	-4.607700025	-1.502120945	-4.922129028
H	-5.013185371	-0.217318354	-3.797803333
C	-6.957783748	-2.260813631	-0.835165217
C	-8.345650417	-2.231593848	-0.597664840
C	-6.331449068	-3.502563625	-1.063045548
C	-9.080242998	-3.406627537	-0.587215160
C	-7.070563885	-4.675087539	-1.047238634
C	-8.442987362	-4.625128225	-0.810547818
H	-8.848197213	-1.284549443	-0.408524835
H	-5.262943936	-3.548166730	-1.270072209
H	-10.150105800	-3.377070175	-0.400760302
H	-6.581710253	-5.628755270	-1.226252294
H	-9.022023759	-5.545126359	-0.801922154
C	-6.779042537	0.910648750	-0.927085844
C	-8.049014083	1.146359757	-1.491527630
C	-6.110661863	1.977891816	-0.291059610
C	-8.622569701	2.405686234	-1.424727819
H	-8.574493917	0.350726531	-2.016519275
C	-6.695816580	3.232932078	-0.219165982
H	-5.133601442	1.819346463	0.162696659
C	-7.948568788	3.445899218	-0.786862451
H	-9.596684617	2.581069923	-1.873140796
H	-6.176151101	4.046212585	0.279840149
H	-8.403894081	4.431754016	-0.734315132
C	-7.365489215	-0.374631959	-6.087244591
C	-8.024532630	0.856942102	-5.988349189
C	-6.888914915	-0.771168257	-7.344285815
C	-8.202855998	1.666909635	-7.105181314
H	-8.406399160	1.189940940	-5.021109548
C	-7.066578150	0.032157266	-8.464126360
H	-6.369985345	-1.725615250	-7.454673022
C	-7.725706529	1.252383952	-8.344323391
H	-8.718821313	2.620100051	-7.011964366
H	-6.693224501	-0.292114359	-9.432955780
H	-7.867011730	1.881986943	-9.219922199

==============================

11B

==============================

C	-7.113579116	1.407377751	0.137177168
C	-6.658075215	0.091721944	0.311999931
C	-7.425663837	1.852618798	-1.159235429
C	-6.526174371	-0.758425033	-0.778859729
C	-7.279985938	1.004304827	-2.248066292
C	-6.834083011	-0.301045061	-2.056207801
H	-6.413917039	-0.272148884	1.309566038
H	-7.780745862	2.870601252	-1.321335419
H	-6.182996359	-1.779293820	-0.633043408
H	-7.518926364	1.360719242	-3.247171580
H	-6.727842697	-0.967550271	-2.908568296
H	-6.120391755	3.656424094	1.251391396
C	-5.237772397	6.055773759	1.912667702
H	-4.895379998	5.582394917	2.840183078
H	-4.542188503	6.867579521	1.670571775
H	-6.221654494	6.502353011	2.092318238
Si	-5.288178321	4.823363817	0.524610301
C	-6.578613631	1.957629427	3.191006938
C	-7.365410200	1.923757783	4.350558220
C	-5.236418478	1.552586550	3.266781895
C	-6.825465097	1.484211471	5.553577897
C	-4.697360669	1.119962925	4.470056794
C	-5.494581568	1.085188005	5.612042736
H	-8.407694967	2.238619367	4.312430893
H	-4.609530526	1.568653035	2.372752852
H	-7.443485025	1.454129106	6.447060908
H	-3.657650120	0.807403211	4.521751349

```
 H   -5.073019456   0.744542996   6.554405612
 Si  -7.286692609   2.522038034   1.588747543
 C   -3.715224591   3.911273749   0.145788591
 H   -3.178822800   3.656179951   1.065604401
 H   -3.047397788   4.524601450  -0.470661460
 H   -3.924443810   2.985278193  -0.403371944
 C   -8.778086125   3.594833481   1.676984591
 C   -9.860427147   3.412711846   0.805588618
 C   -8.827380667   4.640701971   2.613697481
 C   -10.962606470   4.257508823   0.865601073
 C   -9.924740773   5.489433582   2.666947023
 C   -10.990714942   5.296756795   1.789876585
 H   -9.843369078   2.602337170   0.077856795
 H   -8.000336558   4.788957014   3.310664022
 H   -11.801523321   4.104350556   0.191653285
 H   -9.956770345   6.296289739   3.394644352
 H   -11.852577465   5.958064139   1.834389804
 C   -6.322082677   5.245506245  -0.941664390
 C   -5.892922830   4.903193781  -2.232795272
 C   -7.578975148   5.851719070  -0.786803710
 C   -6.695044473   5.160697040  -3.337535219
 C   -8.387141597   6.095751068  -1.889481596
 C   -7.943333085   5.751875464  -3.163887929
 H   -4.924941032   4.424988965  -2.380216488
 H   -7.944692602   6.118198077   0.205741213
 H   -6.349021524   4.899000171  -4.334241167
 H   -9.363145097   6.555677469  -1.755368420
 H   -8.574012077   5.947702710  -4.027666554
```

==============================

12B-Me2PhSi+

==============================

```
 C   -2.624725336   0.433110404  -4.434501679
 C   -3.510211796  -0.580850138  -4.864042712
 C   -2.915263901   1.779762410  -4.750159329
 C   -4.643987494  -0.255910547  -5.586579500
 C   -4.053632262   2.097025419  -5.468447844
 C   -4.912887840   1.079812559  -5.885613492
 H   -3.306816062  -1.625068788  -4.633787442
 H   -2.248259348   2.577934101  -4.427891370
 H   -5.322608729  -1.036155994  -5.918595494
 H   -4.277638878   3.132235379  -5.708566213
 H   -5.805726001   1.332214963  -6.452304137
 C   -0.751628936  -1.711645265  -3.057442278
 H   -0.325786558  -1.749632021  -2.033787677
 H   -1.627511172  -2.385455338  -3.118736435
 H    0.034137976  -2.097844750  -3.749638109
 Si  -1.145665950   0.024703048  -3.505308792
 C    0.008723889   1.358709647  -2.985245120
 H    0.981348570   0.923091305  -2.622805701
 H    0.199408769   2.079798811  -3.830944868
 H   -0.459120573   1.948629805  -2.129551787
```

==============================

12B-Ph3SiH

==============================

```
 H    4.634065841  11.618441676  -3.395548305
 C    2.218993987  11.777736964  -2.169692944
 C    2.654181350  12.922622433  -1.491853454
 C    0.932971668  11.293860237  -1.888732476
 C    1.837175080  13.565274043  -0.566825208
 C    0.111433117  11.930907649  -0.967157947
 C    0.564421291  13.069667415  -0.304917740
 H    3.651261203  13.318394116  -1.689602273
 H    0.569319919  10.396579709  -2.393766209
 H    2.194772040  14.453345640  -0.048588458
 H   -0.883521687  11.539700488  -0.762229255
 H   -0.076714032  13.569812162   0.417831567
 C    3.414970692   9.109919979  -3.075579141
```

C	3.244924317	8.625031565	-1.771955511
C	3.697310063	8.185132253	-4.090222054
C	3.355386707	7.267772058	-1.489967467
C	3.808797453	6.827183266	-3.814425713
C	3.637958582	6.367325819	-2.512196878
H	3.011616552	9.320744431	-0.965140482
H	3.820304878	8.532573501	-5.116752055
H	3.216557286	6.910919977	-0.471186374
H	4.025095775	6.124988660	-4.617299731
H	3.720963073	5.304554217	-2.294522212
Si	3.305366222	10.945364103	-3.450056233
C	2.634514990	11.185973195	-5.183503228
C	3.189704644	12.149151276	-6.034725163
C	1.533631315	10.452408196	-5.647791651
C	2.665870509	12.376505417	-7.303133330
H	4.050095722	12.730498156	-5.700530029
C	1.003638329	10.675842727	-6.912736320
H	1.089667381	9.682395227	-5.013975528
C	1.570417929	11.640299462	-7.742108495
H	3.114008106	13.127651707	-7.951043050
H	0.148879845	10.095451717	-7.255411688
H	1.158427962	11.815354973	-8.733682521

==============================

13B-Ph3Si+

==============================

C	-1.648602067	5.916179465	-0.607315871
C	-0.425445839	6.480550552	-0.187031674
C	-1.618416348	4.792042346	-1.459701890
C	0.777464639	5.958127651	-0.629460183
C	-0.411582066	4.283316462	-1.910483633
C	0.783411978	4.868599262	-1.498984180
H	-0.413108514	7.309062842	0.516732053
H	-2.545894030	4.309457607	-1.762322221
H	1.714352237	6.393000835	-0.293288993
H	-0.398478154	3.422753959	-2.573198697
H	1.729856699	4.462976788	-1.847188874
Si	-3.251780581	6.524491041	-0.020332836
C	-4.732419129	5.486835250	-0.290946247
C	-5.096199454	5.097857336	-1.592618380
C	-5.524935492	5.069094941	0.792998081
C	-6.227557966	4.321257522	-1.800982826
C	-6.643547938	4.276149694	0.577940795
C	-6.996103105	3.907755836	-0.717023529
H	-4.507982381	5.418578496	-2.452285485
H	-5.257791588	5.343110166	1.813466384
H	-6.510412332	4.036994084	-2.810742372
H	-7.242441404	3.944911265	1.421772070
H	-7.876924456	3.293181764	-0.882771298
C	-3.449155818	8.117849401	0.820617011
C	-4.666252750	8.391172612	1.480390810
C	-2.457361654	9.121101361	0.785911740
C	-4.862589945	9.603710062	2.120604675
C	-2.666432917	10.336633505	1.413998285
C	-3.862133897	10.572172373	2.090522356
H	-5.465381207	7.652068197	1.482644169
H	-1.533006533	8.964053405	0.235592493
H	-5.799014843	9.801593352	2.634252825
H	-1.902013400	11.107209342	1.373320064
H	-4.021114116	11.526350920	2.586233086

==============================

13B-PhMe2SiH

==============================

Si	-2.2010587379	-0.2625150744	-5.5481060162
C	-2.6788657607	-1.0648806125	-7.1795520101
H	-3.7653490278	-1.0945225447	-7.3124363685
H	-2.2576597553	-0.4925126476	-8.0155844398
H	-2.2990376385	-2.0898963394	-7.2525043378

C	-2.8382288695	1.5023545887	-5.4966124492
C	-3.7333881975	1.9355830959	-4.5122479262
C	-2.4072408015	2.4390713051	-6.4456565967
C	-4.1876509860	3.2507756618	-4.4780718784
C	-2.8550651381	3.7540546952	-6.4189217706
C	-3.7487863848	4.1613299426	-5.4325180794
H	-4.0857755338	1.2309622483	-3.7585852857
H	-1.7066753146	2.1353079380	-7.2262767533
H	-4.8856353826	3.5660649708	-3.7051247846
H	-2.5087148173	4.4642644891	-7.1668891604
H	-4.1016751052	5.1901984642	-5.4081130981
C	-0.3275528112	-0.2563249779	-5.3904088363
H	-0.0016738171	0.1921969156	-4.4463460908
H	0.0874051585	-1.2687987057	-5.4476629645
H	0.1137408744	0.3299197384	-6.2061851800
H	-2.8219344840	-0.9939834601	-4.4082510384

==============================

14B

==============================

C	-2.849324962	0.579864258	-4.239672118
C	-3.616675411	-0.354133748	-4.951085081
C	-3.183722802	1.939424383	-4.331837812
C	-4.670310582	0.062839015	-5.756890748
C	-4.250925016	2.354926028	-5.115520373
C	-4.984768279	1.415684850	-5.838517185
H	-3.390933149	-1.417899685	-4.880217668
H	-2.619734061	2.682748854	-3.764678676
H	-5.255585740	-0.667268814	-6.310196765
H	-4.508540109	3.409451353	-5.172267066
H	-5.817931482	1.741082990	-6.457278100
C	-1.397434056	-1.806733068	-2.824753617
H	-0.603525174	-2.049527561	-2.110720462
H	-2.348209949	-2.174722455	-2.419677143
H	-1.193933863	-2.365374468	-3.749422125
Si	-1.468012186	0.024064551	-3.159027779
C	0.146317580	0.937333745	-3.312445639
H	0.768069690	0.798534431	-2.419516214
H	0.715413100	0.575835996	-4.176855478
H	-0.020976363	2.011539918	-3.453600890
H	-1.850199637	0.605524019	-1.701393218
Si	-2.747167806	1.004791289	-0.374635531
C	-1.845298672	2.577707279	0.031395566
H	-2.333206485	3.065737071	0.885281147
H	-0.802444768	2.397428407	0.309835923
H	-1.867311477	3.275750645	-0.813679670
C	-4.442325373	1.214893252	-1.038773135
C	-5.072239408	2.467130746	-0.986060435
C	-5.094090654	0.154805750	-1.685690615
C	-6.320207276	2.652662856	-1.568044173
C	-6.327783419	0.347462040	-2.290624580
C	-6.941015179	1.596223729	-2.226747886
H	-4.589004081	3.306848631	-0.487601381
H	-4.636741183	-0.836217177	-1.710663640
H	-6.810534746	3.620569863	-1.504604569
H	-6.825500961	-0.481744249	-2.786527313
H	-7.919924297	1.740525292	-2.676936591
C	-2.396719457	-0.455248286	0.681277842
C	-3.430292457	-1.157718484	1.314175432
C	-1.067588583	-0.842421736	0.909005953
C	-3.139058259	-2.215393996	2.167826113
C	-0.778428255	-1.907749195	1.747376995
C	-1.816569421	-2.588548082	2.381142934
H	-4.467852328	-0.868581110	1.147073155
H	-0.248559628	-0.309590313	0.419008651
H	-3.942885422	-2.748050589	2.669029214
H	0.252572410	-2.206903076	1.916538048
H	-1.589248092	-3.414816403	3.049939606

==============================

14B-TS

==============================

C	-0.7713736757	-2.0007758698	-3.1730520672
C	-0.4091135838	-2.2741271814	-4.5095069124
C	-0.2613311330	-2.8779881408	-2.1850881710
C	0.3738601998	-3.3694016189	-4.8377477129
C	0.5112469596	-4.0042893410	-2.5227544272
C	0.8337053544	-4.2440497614	-3.8440934530
H	-0.7733684629	-1.6266273680	-5.3067157083
H	-0.5974258015	-2.7642677227	-1.1541999441
H	0.6213193613	-3.5619166477	-5.8791266030
H	0.8666362660	-4.6669775051	-1.7370543665
H	1.4348761611	-5.1077927228	-4.1143632861
H	-1.5289460544	0.6347594567	-2.7984158320
Si	-2.1501141231	-0.7173681559	-2.7895887151
C	-2.8895645280	-1.0944934699	-1.1151973254
H	-3.7387439684	-0.4273368677	-0.9292997694
H	-3.2666727230	-2.1229630791	-1.0700923216
H	-2.1747053779	-0.9506847807	-0.2960678039
C	2.9114987301	-2.0378419548	-1.9253990760
C	3.3743099969	-2.5258611497	-0.6925568697
C	3.6601410357	-2.3135736943	-3.0818202081
C	4.5405998194	-3.2747940480	-0.6211669380
C	4.8255990584	-3.0638368430	-3.0085579965
C	5.2627885298	-3.5479460814	-1.7790518224
H	2.8366695385	-2.2969546389	0.2255609693
H	3.3355423624	-1.9388284389	-4.0515745130
H	4.8933605481	-3.6384937681	0.3402906467
H	5.3996020515	-3.2656474709	-3.9090639157
H	6.1774541687	-4.1325474539	-1.7212906850
Si	1.3995200314	-0.9961402393	-2.0388306258
C	1.4825384030	0.2086175427	-3.4483023144
H	1.7645307113	-0.2841240544	-4.3835546152
H	0.5478713643	0.7520465062	-3.6098648790
H	2.2721714060	0.9332945887	-3.2065083831
C	0.6894845020	-0.3465096308	-0.4677782609
C	0.4930089396	-1.1404263960	0.6756233820
C	0.3864948398	1.0219300879	-0.3926418346
C	0.0165045233	-0.5843528399	1.8534947478
H	0.7041105826	-2.2099917044	0.6475755280
C	-0.0917183885	1.5788795372	0.7883307452
H	0.5244910305	1.6650298164	-1.2607422728
C	-0.2765535733	0.7770874704	1.9085147613
H	-0.1306870687	-1.2102185056	2.7296480384
H	-0.3209677962	2.6400713765	0.8327661468
H	-0.6528784958	1.2122876720	2.8306677230
C	-3.4021482630	-0.8540627458	-4.1710223381
H	-2.9916971266	-0.5527177446	-5.1406607330
H	-3.7780391877	-1.8787905671	-4.2652114109
H	-4.2582794224	-0.2025518612	-3.9647724113

==============================

15B

==============================

C	-2.307070533	0.521714810	-3.378443919
C	-2.859774173	-0.746323073	-3.746149862
C	-1.961327504	1.393614113	-4.459840633
C	-3.007520097	-1.127237568	-5.067792214
C	-2.115883318	1.020769670	-5.780573369
C	-2.631925516	-0.243409534	-6.080824972
H	-3.153861819	-1.433518942	-2.950134025
H	-1.558011981	2.380303175	-4.227816238
H	-3.413386679	-2.103513999	-5.317475976
H	-1.843366104	1.702133466	-6.581324996
H	-2.746385555	-0.540806249	-7.120760974
C	0.187292266	-1.330322434	-3.391096380
H	1.205802050	-1.500206144	-3.022938369
H	-0.365352164	-2.269634080	-3.294884097

H	0.263409641	-1.080595271	-4.456551662
Si	-0.583759842	0.071234071	-2.434557829
C	0.544147091	1.555203479	-2.466447517
H	1.401730325	1.346845802	-1.815141543
H	0.936643831	1.706022898	-3.478964188
H	0.091068025	2.488696306	-2.123354316
H	-1.023052399	-0.332909502	-1.081080088
Si	-3.428679939	1.426204954	-1.952190477
C	-2.415705197	2.681844033	-1.012046404
H	-3.073736834	3.133400306	-0.258257493
H	-1.565849898	2.256741756	-0.468571919
H	-2.053172440	3.492410614	-1.654858208
C	-4.759024271	2.263776494	-2.952431771
C	-5.140757014	3.573208609	-2.630141905
C	-5.439284414	1.615479282	-3.993625290
C	-6.168444509	4.208960773	-3.319039452
C	-6.467620908	2.245585566	-4.681309602
C	-6.833401364	3.545205548	-4.342743934
H	-4.636661436	4.112181928	-1.828893436
H	-5.164209480	0.598959933	-4.278735085
H	-6.450584815	5.224668165	-3.053895351
H	-6.986560660	1.724483552	-5.482117186
H	-7.638191436	4.040636007	-4.879956066
C	-4.078030512	0.080802508	-0.843229909
C	-5.237469192	-0.629621309	-1.192218890
C	-3.463193108	-0.214982151	0.382228883
C	-5.751051143	-1.613962293	-0.357493526
C	-3.977746311	-1.196317679	1.221381877
C	-5.118201139	-1.899560341	0.848648332
H	-5.760349965	-0.395464222	-2.120151299
H	-2.577007789	0.334597169	0.698920283
H	-6.652478460	-2.151381211	-0.641064472
H	-3.491618319	-1.409110823	2.170032001
H	-5.522117019	-2.666135721	1.505221189

==============================

15B-TS

==============================

C	-0.578311086	-1.494558930	-2.056690454
C	-0.734803617	-2.032912016	-3.362584114
C	-1.478675008	-1.964186549	-1.064839840
C	-1.687801242	-2.990958691	-3.639998913
C	-2.446861267	-2.953114271	-1.348948956
C	-2.547031879	-3.457823753	-2.628363609
H	-0.064750083	-1.699066401	-4.154787064
H	-1.328057885	-1.662650824	-0.023491912
H	-1.769329786	-3.400414467	-4.644689083
H	-3.105800152	-3.304593801	-0.558373630
H	-3.283049822	-4.224581718	-2.856953859
H	-1.408770919	0.894330859	-0.763557196
C	-2.326440811	0.707028210	-3.441924334
H	-1.344416499	0.966679931	-3.850010633
H	-2.806926250	-0.036557987	-4.087928772
H	-2.956799746	1.606656194	-3.450721025
Si	-2.248765230	0.112866409	-1.686666965
C	-3.958191156	-0.200910345	-1.016528487
H	-4.489218235	0.760576189	-0.999042273
H	-4.518640041	-0.876736403	-1.672008157
H	-3.961197853	-0.606550932	-0.000316631
C	2.377659321	-1.790149689	-2.279506207
C	2.198895693	-3.181797266	-2.172789097
C	3.581889629	-1.309060812	-2.822222233
C	3.183545589	-4.063004017	-2.601994514
C	4.571682930	-2.188456535	-3.249262571
C	4.371488094	-3.565158129	-3.142822266
H	1.275257468	-3.589020014	-1.746668816
H	3.747492075	-0.233398885	-2.922276735
H	3.027772903	-5.138958931	-2.513115644
H	5.498327732	-1.798260331	-3.671356440

H	5.142178535	-4.253385067	-3.480918407
C	1.176285625	-0.418066531	0.228810757
H	0.357084692	0.161596164	0.669962525
H	1.198680639	-1.406547904	0.704160273
H	2.113282681	0.084132530	0.496361256
Si	1.096735954	-0.599537611	-1.629813313
C	1.151956201	1.030449033	-2.535908222
C	0.912382662	2.242380619	-1.870926499
C	1.361910820	1.068630338	-3.924445391
C	0.862871289	3.446473360	-2.566780567
C	1.311451316	2.269578457	-4.624416351
C	1.055058241	3.458944321	-3.944860935
H	0.768065929	2.252298832	-0.789783180
H	1.589114904	0.148581624	-4.467459202
H	0.680709302	4.377052307	-2.032864332
H	1.480095148	2.280727863	-5.698944092
H	1.016618371	4.399210453	-4.490189552

===============================

16B

===============================

C	-2.207861185	1.384471297	-2.209895372
C	-1.936509490	0.637750447	-1.063988447
C	-2.311004877	0.663478732	-3.448616505
C	-1.829323649	-0.750820577	-1.102049351
C	-2.319343567	-0.765449047	-3.443100452
C	-2.029989004	-1.462299109	-2.288455486
H	-1.852111697	1.142493963	-0.102138773
H	-2.799479008	1.172412753	-4.290911674
H	-1.614859939	-1.294752002	-0.184885755
H	-2.516629696	-1.297269702	-4.371430397
H	-1.994018793	-2.547407866	-2.292172670
C	-0.138220355	-0.475097626	-5.546241760
H	0.792561650	-0.254029572	-6.083535194
H	-0.053631440	-1.483571529	-5.132044792
H	-0.944525540	-0.462019175	-6.288958549
Si	-0.371534288	0.840445459	-4.249596119
C	-0.317037672	2.518432617	-5.043755054
H	-1.101507902	2.609338045	-5.805601597
H	-0.426967889	3.357858181	-4.354070663
H	0.650956273	2.614579678	-5.552250385
H	0.459859580	0.650819719	-3.044646740
Si	-2.464614868	3.283931732	-1.984244227
C	-0.797863543	3.995987654	-1.504489779
H	-0.906469226	5.043850899	-1.200366974
H	-0.372556001	3.453125000	-0.651881695
H	-0.058670685	3.956210375	-2.312197447
C	-3.120256901	3.958982944	-3.595196724
C	-2.566509247	5.114524841	-4.162444115
C	-4.173007488	3.330648422	-4.280808449
C	-3.029317617	5.612427711	-5.376005173
C	-4.636691570	3.822359800	-5.496032238
C	-4.059417725	4.961998463	-6.046952248
H	-1.755626678	5.636466980	-3.653305531
H	-4.650925159	2.444199324	-3.858027458
H	-2.586292744	6.510452271	-5.799126625
H	-5.452847004	3.321253777	-6.010722160
H	-4.419423103	5.348411083	-6.996989250
C	-3.672040224	3.435737133	-0.569896340
C	-5.055471897	3.418696404	-0.796465099
C	-3.217862844	3.545222759	0.752711654
C	-5.953548908	3.495431423	0.260869294
C	-4.113857269	3.623650074	1.812045932
C	-5.482420444	3.594716072	1.565769672
H	-5.440416336	3.365928650	-1.814899206
H	-2.148961544	3.587335110	0.965153933
H	-7.023117542	3.485711098	0.066681452
H	-3.744540215	3.715743303	2.830376387
H	-6.184367180	3.658329964	2.393316746

==============================
16B-TS
==============================

C	-2.599230112	0.297949882	-3.281029208
C	-2.250765138	-1.040833324	-3.043542475
C	-2.229921227	0.865575118	-4.512916008
C	-1.555060512	-1.783099223	-3.996364227
C	-1.555599295	0.123468074	-5.474512592
C	-1.211581209	-1.202973151	-5.214888288
H	-2.556518506	-1.516410010	-2.108627059
H	-2.515727844	1.894271318	-4.739647267
H	-1.305250056	-2.822960959	-3.801266775
H	-1.305191214	0.571820159	-6.432241561
H	-0.691280011	-1.789544658	-5.970656564
C	0.929568282	1.014079190	-3.177150494
H	1.983311438	0.699490761	-3.109079501
H	0.397132624	0.295207093	-3.806291101
H	0.912705718	2.004641347	-3.642630636
Si	0.319701146	1.052354392	-1.454258189
C	0.971340681	2.369223534	-0.348418155
H	2.026815200	2.142795886	-0.138654877
H	0.947624479	3.341769535	-0.849473324
H	0.442599596	2.437752484	0.604224863
H	-0.243324195	-0.159018339	-0.841536081
Si	-3.608720976	1.313686702	-2.051956543
C	-2.304877415	2.438397447	-1.246523591
H	-2.645669514	3.189966808	-0.525422652
H	-1.678598076	1.755498781	-0.636879158
H	-1.718250723	2.952753963	-2.022131321
C	-4.898845430	2.312158766	-2.941860824
C	-5.256648738	3.590017788	-2.491997870
C	-5.588082261	1.775330541	-4.039070734
C	-6.267927848	4.310561847	-3.116211044
C	-6.602297735	2.491303249	-4.663391043
C	-6.940548711	3.760236359	-4.202453740
H	-4.746646452	4.032562775	-1.635508592
H	-5.328125334	0.783915034	-4.413457130
H	-6.534057836	5.301136974	-2.754664356
H	-7.129606568	2.060987193	-5.511548569
H	-7.732847134	4.322059191	-4.691026696
C	-4.327101620	0.189143225	-0.746957038
C	-5.618278866	-0.337427112	-0.926188402
C	-3.592600105	-0.224741900	0.377872768
C	-6.151284542	-1.248672690	-0.021537151
C	-4.120376270	-1.133600681	1.287226188
C	-5.401755869	-1.648865923	1.084493234
H	-6.222482534	-0.019262230	-1.780449629
H	-2.587852446	0.172160785	0.565907047
H	-7.156283262	-1.643558342	-0.176148430
H	-3.536587720	-1.438682124	2.156518821
H	-5.818383050	-2.359127756	1.794596036

==============================
17B
==============================

C	2.380270640	0.771617824	-3.045087768
C	2.778336000	-0.199434837	-2.115413067
C	3.340400148	1.298713909	-3.924520292
C	4.098657465	-0.632349325	-2.068269434
C	4.658323986	0.868839429	-3.874278901
C	5.035806062	-0.097614339	-2.944227356
H	2.049718861	-0.626108407	-1.427368386
H	3.057053193	2.044195946	-4.671169983
H	4.395656219	-1.389315532	-1.347371780
H	5.393140810	1.281015584	-4.560948480
H	6.068006850	-0.436188413	-2.906071606
C	2.867477193	5.647098882	-3.470863491
H	3.152016022	6.704283059	-3.484825610
H	3.533332994	5.134504222	-2.767976523

H	3.054058457	5.244441795	-4.473951390
Si	1.076724829	5.439143047	-3.003126718
C	-0.130362826	5.989336908	-4.310202260
H	-0.046193791	7.065207848	-4.496676829
H	0.062152235	5.473985302	-5.259231330
H	-1.165809666	5.786232454	-4.016132030
H	0.754705515	5.941752692	-1.651884425
Si	0.615367300	1.294922262	-3.125790938
C	0.815450228	3.477574250	-2.868867449
H	-0.262423517	3.449629226	-2.689525307
H	1.478800939	3.242786908	-2.033199278
H	1.178522985	3.302966004	-3.887078241
C	-0.172196125	1.211465049	-4.792533372
C	-1.373684196	1.882682442	-5.074949691
C	0.425222057	0.443395911	-5.802325588
C	-1.960000387	1.786010513	-6.328807888
C	-0.166580042	0.342998409	-7.056548245
C	-1.355146366	1.013907137	-7.318456052
H	-1.867949655	2.478910027	-4.305026651
H	1.357906458	-0.084340806	-5.605383094
H	-2.890885987	2.307038940	-6.536823822
H	0.301486767	-0.260050125	-7.830016234
H	-1.816339252	0.935547656	-8.299598842
C	-0.446291663	0.820842052	-1.700204117
C	-1.690456997	0.216701123	-1.934374182
C	-0.025532768	1.014971538	-0.374654769
C	-2.489632286	-0.186040861	-0.870509721
C	-0.826006002	0.616497327	0.685447506
C	-2.057990768	0.015306728	0.435631463
H	-2.033281613	0.050343184	-2.955124127
H	0.941992502	1.473408224	-0.164726271
H	-3.449086234	-0.659547388	-1.061598592
H	-0.491814506	0.769394854	1.708211463
H	-2.683664482	-0.299802137	1.266949758

==============================

17B-TS

==============================

C	1.376187164	3.311775001	1.186980039
C	1.972638862	4.197574189	2.107450727
C	2.072710701	2.146274454	0.810203613
C	3.224341671	3.922745279	2.634415919
C	3.327644091	1.883072950	1.333701003
C	3.900134383	2.769685465	2.244852836
H	1.444856673	5.094967055	2.425165086
H	1.633234667	1.454932855	0.091427356
H	3.674762758	4.604953426	3.350530284
H	3.864094412	0.987847571	1.033750206
H	4.883146567	2.558555514	2.656760617
C	2.606520099	5.532490420	-4.125650346
H	2.504371538	6.583995723	-4.416014865
H	3.537389287	5.435601938	-3.548699125
H	2.722489108	4.941298706	-5.045280296
Si	1.120181011	4.932968729	-3.154500484
C	-0.477052656	5.051559633	-4.148037735
H	-0.698467005	6.085578584	-4.439305397
H	-0.415367197	4.446319691	-5.064850486
H	-1.336408458	4.683031241	-3.567506631
H	0.942922050	5.750483095	-1.905276110
Si	-0.272833124	3.654419034	0.515130843
C	1.382173332	3.133861580	-2.634472621
H	0.494804640	2.716291427	-2.134631884
H	2.248195259	3.018866251	-1.968030563
H	1.558052681	2.503645199	-3.518283447
C	-1.327516826	2.275800426	-0.014797813
C	-2.308001103	2.462629609	-1.008208509
C	-1.178732256	1.002418004	0.569695055
C	-3.109984699	1.404685622	-1.406141389
C	-1.995990665	-0.047445104	0.178035000

C	-2.956249639	0.154477888	-0.808985086
H	-2.426294471	3.437111364	-1.482007413
H	-0.429888236	0.837988365	1.342844577
H	-3.855993794	1.550100703	-2.181897475
H	-1.886034144	-1.024793431	0.640488127
H	-3.591093884	-0.671720130	-1.118971706
C	-0.925997480	5.343732357	0.506643122
C	-2.315607229	5.564724985	0.546901515
C	-0.055062228	6.452014279	0.484477284
C	-2.815846917	6.856595505	0.572400544
C	-0.562355266	7.739751331	0.500668423
C	-1.939985870	7.939029837	0.546969003
H	-3.002917128	4.720691438	0.576556705
H	1.022018874	6.294124115	0.429171400
H	-3.887400202	7.023423818	0.613554964
H	0.111660897	8.591207639	0.472365883
H	-2.335479443	8.951041521	0.561902360

==============================

18B

==============================

C	-2.092764446	5.719172653	2.508630466
C	-1.688420141	6.381217026	3.676340799
C	-2.142751734	4.316360896	2.505526829
C	-1.335436965	5.656633915	4.808843909
C	-1.784056615	3.594391586	3.634294811
C	-1.379053385	4.267111026	4.785314656
H	-1.658729523	7.469644091	3.708660420
H	-2.474753243	3.781928321	1.612776346
H	-1.026708128	6.177480847	5.711325803
H	-1.823880124	2.508338345	3.622935643
H	-1.099888926	3.702072802	5.671008371
C	0.880566469	5.650957560	-0.030867017
H	1.040669901	6.681269511	0.304311432
H	0.781261789	5.005316390	0.849244684
H	1.777704592	5.333270010	-0.575537667
Si	-0.604316335	5.515679714	-1.135096132
C	-0.892517255	6.901455338	-2.335134941
H	-0.667608389	7.874917392	-1.884135769
H	-0.253291477	6.778200454	-3.217136531
H	-1.934067301	6.910315990	-2.679267213
H	-1.823472023	5.690737159	-0.106548115
Si	-2.622139860	6.639384598	1.005241407
C	-0.958581896	3.801528344	-1.761171918
H	-1.872316149	3.764619437	-2.363206933
H	-1.048545346	3.084957106	-0.937350393
H	-0.129917766	3.466319287	-2.397114034
C	-4.298851745	6.334631386	0.296765250
C	-4.563884660	5.266242374	-0.570202303
C	-5.356493400	7.175118224	0.677831364
C	-5.848866856	5.044860126	-1.048565697
C	-6.641762410	6.950542775	0.200920274
C	-6.886317505	5.887437897	-0.662643656
H	-3.761065821	4.593063504	-0.875217674
H	-5.178897589	8.013606691	1.352054037
H	-6.042940219	4.214921206	-1.722955226
H	-7.453954499	7.606674720	0.502371596
H	-7.891688029	5.714823348	-1.037904887
C	-1.955347553	8.336020254	0.771403079
C	-2.624163774	9.235010891	-0.075206273
C	-0.726383949	8.723875313	1.332935350
C	-2.083728177	10.484959861	-0.348136737
C	-0.182713160	9.970098503	1.051006982
C	-0.862485886	10.849048351	0.211345680
H	-3.573061671	8.950401350	-0.529339423
H	-0.188646773	8.044095901	1.994465188
H	-2.612295960	11.175143206	-1.000304705
H	0.768653778	10.260825698	1.488816580
H	-0.436811917	11.825205121	-0.007277047

==============================
19B-Me3Si+
==============================

C	-3.312073205	-0.300161576	-1.091388748
H	-3.160506183	0.649229544	-0.571447702
H	-4.324198019	-0.668792945	-0.872379114
H	-2.625290462	-1.052259962	-0.679762122
Si	-3.083964106	-0.170878431	-2.901809628
C	-3.214562112	-1.666956893	-3.946446819
H	-3.543306229	-2.548643021	-3.390253109
H	-3.895880258	-1.488492493	-4.788425522
H	-2.233816796	-1.882238427	-4.395385980
C	-2.725097208	1.450859340	-3.668510961
H	-2.534348203	1.381205900	-4.742603089
H	-3.573754596	2.130306377	-3.504269799
H	-1.864806566	1.922366920	-3.175267092

==============================
19B-Ph3SiH
==============================

H	4.634065841	11.618441676	-3.395548305
C	2.218993987	11.777736964	-2.169692944
C	2.654181350	12.922622433	-1.491853454
C	0.932971668	11.293860237	-1.888732476
C	1.837175080	13.565274043	-0.566825208
C	0.111433117	11.930907649	-0.967157947
C	0.564421291	13.069667415	-0.304917740
H	3.651261203	13.318394116	-1.689602273
H	0.569319919	10.396579709	-2.393766209
H	2.194772040	14.453345640	-0.048588458
H	-0.883521687	11.539700488	-0.762229255
H	-0.076714032	13.569812162	0.417831567
C	3.414970692	9.109919979	-3.075579141
C	3.244924317	8.625031565	-1.771955511
C	3.697310063	8.185132253	-4.090222054
C	3.355386707	7.267772058	-1.489967467
C	3.808797453	6.827183266	-3.814425713
C	3.637958582	6.367325819	-2.512196878
H	3.011616552	9.320744431	-0.965140482
H	3.820304878	8.532573501	-5.116752055
H	3.216557286	6.910919977	-0.471186374
H	4.025095775	6.124988660	-4.617299731
H	3.720963073	5.304554217	-2.294522212
Si	3.305366222	10.945364103	-3.450056233
C	2.634514990	11.185973195	-5.183503228
C	3.189704644	12.149151276	-6.034725163
C	1.533631315	10.452408196	-5.647791651
C	2.665870509	12.376505417	-7.303133330
H	4.050095722	12.730498156	-5.700530029
C	1.003638329	10.675842727	-6.912736320
H	1.089667381	9.682395227	-5.013975528
C	1.570417929	11.640299462	-7.742108495
H	3.114008106	13.127651707	-7.951043050
H	0.148879845	10.095451717	-7.255411688
H	1.158427962	11.815354973	-8.733682521

==============================
20B-Ph3Si+
==============================

C	-1.648602067	5.916179465	-0.607315871
C	-0.425445839	6.480550552	-0.187031674
C	-1.618416348	4.792042346	-1.459701890
C	0.777464639	5.958127651	-0.629460183
C	-0.411582066	4.283316462	-1.910483633
C	0.783411978	4.868599262	-1.498984180
H	-0.413108514	7.309062842	0.516732053
H	-2.545894030	4.309457607	-1.762322221
H	1.714352237	6.393000835	-0.293288993

H	-0.398478154	3.422753959	-2.573198697
H	1.729856699	4.462976788	-1.847188874
Si	-3.251780581	6.524491041	-0.020332836
C	-4.732419129	5.486835250	-0.290946247
C	-5.096199454	5.097857336	-1.592618380
C	-5.524935492	5.069094941	0.792998081
C	-6.227557966	4.321257522	-1.800982826
C	-6.643547938	4.276149694	0.577940795
C	-6.996103105	3.907755836	-0.717023529
H	-4.507982381	5.418578496	-2.452285485
H	-5.257791588	5.343110166	1.813466384
H	-6.510412332	4.036994084	-2.810742372
H	-7.242441404	3.944911265	1.421772070
H	-7.876924456	3.293181764	-0.882771298
C	-3.449155818	8.117849401	0.820617011
C	-4.666252750	8.391172612	1.480390810
C	-2.457361654	9.121101361	0.785911740
C	-4.862589945	9.603710062	2.120604675
C	-2.666432917	10.336633505	1.413998285
C	-3.862133897	10.572172373	2.090522356
H	-5.465381207	7.652068197	1.482644169
H	-1.533006533	8.964053405	0.235592493
H	-5.799014843	9.801593352	2.634252825
H	-1.902013400	11.107209342	1.373320064
H	-4.021114116	11.526350920	2.586233086

==============================

20B-Me3SiH

==============================

C	-2.461195359	-0.836356324	-1.265559885
H	-1.923133360	-0.091008810	-0.669483684
H	-3.295797284	-1.207938659	-0.658611958
H	-1.781050487	-1.676185619	-1.445262123
Si	-3.099994222	-0.103414793	-2.876060889
C	-4.000058611	-1.427527580	-3.864015238
H	-4.850818165	-1.817922091	-3.292389838
H	-4.388637016	-1.030090944	-4.808170789
H	-3.343308872	-2.271778987	-4.100872526
H	-1.950526823	0.410793675	-3.674286976
C	-4.279729520	1.312862634	-2.500014822
H	-3.790146591	2.102609904	-1.919575173
H	-4.671626980	1.765392854	-3.417579929
H	-5.134810068	0.951788928	-1.915655251

==============================
00-CH3+
==============================

C	-0.3557214423	0.0000000552	-2.5248120937
H	-1.4301345029	-0.1733610579	-2.6647237583
H	0.2881917378	-0.8109976818	-2.1618848172
H	0.0746792244	0.9843575015	-2.7478450771

==============================
00-CH4
==============================

C	-0.3557113444	-0.0000179080	-2.5263061454
H	-1.3850767864	-0.0089540051	-2.1617160967
H	0.1666775997	-0.8868096464	-2.1615834677
H	0.1511182179	0.8958448517	-2.1617236082
H	-0.3558297126	0.0001290505	-3.6179619499

==============================
1C
==============================

Si	-2.4415263583	-0.2887064001	-5.3676869438
C	-2.9666261843	-1.2613862118	-6.8333765426
H	-3.7408259081	-0.7763660019	-7.4330931803
H	-2.0960117829	-1.4587449215	-7.4713384915
H	-3.3407123474	-2.2401177668	-6.5054900528
C	-3.2333463702	1.2572060491	-4.9182426220
C	-4.3020266573	1.7808089702	-5.6786183808
C	-2.7813367763	1.9699611046	-3.7845432038
C	-4.8978120409	2.9749674282	-5.3151397210
C	-3.3810086507	3.1640985894	-3.4279521949
C	-4.4361661079	3.6628098410	-4.1926720449
H	-4.6660150587	1.2482156716	-6.5555667396
H	-1.9587871146	1.5873645649	-3.1824886007
H	-5.7201469125	3.3760987973	-5.9001464592
H	-3.0325784266	3.7118364874	-2.5572499371
H	-4.9056642243	4.6015155587	-3.9093693556
C	-1.0631991245	-0.9273562121	-4.3333247366
H	-1.3876783169	-1.0600994734	-3.2937229793
H	-0.6908501041	-1.8859278163	-4.7051498246
H	-0.2289370130	-0.2145684280	-4.3191424803

==============================
1CH
==============================

Si	-2.2010587379	-0.2625150744	-5.5481060162
C	-2.6788657607	-1.0648806125	-7.1795520101
H	-3.7653490278	-1.0945225447	-7.3124363685
H	-2.2576597553	-0.4925126476	-8.0155844398
H	-2.2990376385	-2.0898963394	-7.2525043378
C	-2.8382288695	1.5023545887	-5.4966124492
C	-3.7333881975	1.9355830959	-4.5122479262
C	-2.4072408015	2.4390713051	-6.4456565967
C	-4.1876509860	3.2507756618	-4.4780718784
C	-2.8550651381	3.7540546952	-6.4189217706
C	-3.7487863848	4.1613299426	-5.4325180794
H	-4.0857755338	1.2309622483	-3.7585852857
H	-1.7066753146	2.1353079380	-7.2262767533
H	-4.8856353826	3.5660649708	-3.7051247846
H	-2.5087148173	4.4642644891	-7.1668891604
H	-4.1016751052	5.1901984642	-5.4081130981
C	-0.3275528112	-0.2563249779	-5.3904088363
H	-0.0016738171	0.1921969156	-4.4463460908
H	0.0874051585	-1.2687987057	-5.4476629645
H	0.1137408744	0.3299197384	-6.2061851800
H	-2.8219344840	-0.9939834601	-4.4082510384

==============================
2C
==============================

Si	-2.4714902510	-0.2844629970	-5.3973742910
H	-1.3724929700	-0.7432913340	-4.5361696570
C	-2.9396819520	-1.3082061490	-6.8406319210
H	-3.7105075720	-0.8452221370	-7.4620707480
H	-2.0538685110	-1.5059890430	-7.4573043330
H	-3.3028685400	-2.2861021650	-6.4978056930
C	-3.2487864960	1.2520492820	-4.9369489820
C	-4.3201133430	1.8010985660	-5.6817706260
C	-2.7710604160	1.9389710540	-3.7938542250
C	-4.8906365170	2.9967162810	-5.2920673600
C	-3.3493221470	3.1355296300	-3.4134671840
C	-4.4041648090	3.6594896150	-4.1620830040
H	-4.7005423750	1.2865692590	-6.5621871020
H	-1.9479117850	1.5287854820	-3.2105174240
H	-5.7124667910	3.4219382870	-5.8603867910
H	-2.9849511970	3.6659178070	-2.5388084450
H	-4.8567351430	4.6009244640	-3.8605230980

==============================
2CH
==============================

Si	-2.3621424566	-0.2264831844	-5.7196286654
H	-1.1535495119	-0.4100916392	-4.8718417492
C	-2.0170616140	-0.7356048507	-7.4959433428
H	-2.9068101752	-0.5911753285	-8.1188247498
H	-1.2069580204	-0.1349059756	-7.9214990929
H	-1.7331846230	-1.7907202216	-7.5623094116
C	-2.8439044546	1.5845756340	-5.6381462472
C	-3.8516698568	2.0987988253	-6.4644654236
C	-2.2167681174	2.4615872276	-4.7459461086
C	-4.2187744057	3.4378702444	-6.4048094276
C	-2.5782934082	3.8037084403	-4.6809602129
C	-3.5820115546	4.2919794128	-5.5096036691
H	-4.3602480154	1.4418657852	-7.1713468665
H	-1.4261477388	2.0896966000	-4.0940519869
H	-5.0047873324	3.8175621693	-7.0542653826
H	-2.0779089586	4.4695429332	-3.9809491709
H	-3.8672949497	5.3407867088	-5.4621386443
H	-3.4520837981	-1.0770077725	-5.1686671106

==============================
3C
==============================

Si	-3.613646286	0.120272267	-2.823068149
C	-4.661906794	-0.983375538	-3.860597844
H	-5.188282977	-1.737226014	-3.269631946
H	-5.392355316	-0.403422417	-4.435582425
H	-4.029204941	-1.514442713	-4.584036334
C	-2.821901693	1.534672839	-3.619015533
C	-2.343441588	2.634479371	-2.876122209
C	-2.721094530	1.564215105	-5.025846417
C	-1.771948765	3.717903185	-3.521427653
C	-2.137794457	2.645766951	-5.663833947
C	-1.664194786	3.719004144	-4.911147526
H	-2.444697477	2.650538754	-1.792628163
H	-3.093285552	0.733328828	-5.623716816
H	-1.413888991	4.566967395	-2.946310925
H	-2.053396340	2.659201390	-6.746638529
H	-1.212537143	4.570074330	-5.414435310
C	-3.390745626	-0.232475909	-1.065805150
C	-4.325177838	-1.045591995	-0.391063727
C	-2.278828417	0.265921383	-0.353569152
C	-4.165489927	-1.327350364	0.955280133
C	-2.117455891	-0.032656645	0.988505806
C	-3.063139216	-0.821589416	1.641627784
H	-5.188999918	-1.448328374	-0.917336160

```
 H   -1.525949137   0.865924088  -0.861246390
 H   -4.894659690  -1.943972444   1.472781566
 H   -1.253820484   0.343285921   1.529521671
 H   -2.935640258  -1.050538828   2.696588370
```

==============================

3CH

==============================

```
 H    4.314883112  11.039400079  -4.077067935
 C    2.332314321  11.444415041  -2.258174928
 C    3.122021421  12.398432974  -1.605717490
 C    1.022573837  11.251299339  -1.796638372
 C    2.625362017  13.135304576  -0.534732835
 C    0.519819411  11.983081154  -0.727059696
 C    1.322379427  12.929014014  -0.095019814
 H    4.144796435  12.569740070  -1.942630205
 H    0.384139911  10.507339512  -2.277220912
 H    3.256687181  13.872161440  -0.042352272
 H   -0.499412580  11.816638240  -0.384294828
 H    0.931146059  13.504109592   0.741292180
 C    3.255780005   8.651722952  -3.159512308
 C    3.047147750   8.231127497  -1.839552057
 C    3.660681639   7.691302580  -4.099382637
 C    3.234656000   6.902150352  -1.471055123
 C    3.852248045   6.363417828  -3.738113557
 C    3.637775218   5.967113833  -2.419882995
 H    2.730848024   8.955158443  -1.086664709
 H    3.833773308   7.985297596  -5.137324009
 H    3.066533141   6.595872084  -0.439547481
 H    4.168621598   5.635219011  -4.484132988
 H    3.786307995   4.928284446  -2.132848188
 C    1.824184720  10.488591755  -5.148378368
 Si   3.007024088  10.438833712  -3.688957028
 H    2.193908545   9.884900690  -5.985075423
 H    0.844813794  10.087632782  -4.861403239
 H    1.678732744  11.512903097  -5.507960205
```

==============================

4C

==============================

```
 C   -1.648602067   5.916179465  -0.607315871
 C   -0.425445839   6.480550552  -0.187031674
 C   -1.618416348   4.792042346  -1.459701890
 C    0.777464639   5.958127651  -0.629460183
 C   -0.411582066   4.283316462  -1.910483633
 C    0.783411978   4.868599262  -1.498984180
 H   -0.413108514   7.309062842   0.516732053
 H   -2.545894030   4.309457607  -1.762322221
 H    1.714352237   6.393000835  -0.293288993
 H   -0.398478154   3.422753959  -2.573198697
 H    1.729856699   4.462976788  -1.847188874
 Si  -3.251780581   6.524491041  -0.020332836
 C   -4.732419129   5.486835250  -0.290946247
 C   -5.096199454   5.097857336  -1.592618380
 C   -5.524935492   5.069094941   0.792998081
 C   -6.227557966   4.321257522  -1.800982826
 C   -6.643547938   4.276149694   0.577940795
 C   -6.996103105   3.907755836  -0.717023529
 H   -4.507982381   5.418578496  -2.452285485
 H   -5.257791588   5.343110166   1.813466384
 H   -6.510412332   4.036994084  -2.810742372
 H   -7.242441404   3.944911265   1.421772070
 H   -7.876924456   3.293181764  -0.882771298
 C   -3.449155818   8.117849401   0.820617011
 C   -4.666252750   8.391172612   1.480390810
 C   -2.457361654   9.121101361   0.785911740
 C   -4.862589945   9.603710062   2.120604675
 C   -2.666432917  10.336633505   1.413998285
 C   -3.862133897  10.572172373   2.090522356
```

```
H  -5.465381207   7.652068197   1.482644169
H  -1.533006533   8.964053405   0.235592493
H  -5.799014843   9.801593352   2.634252825
H  -1.902013400  11.107209342   1.373320064
H  -4.021114116  11.526350920   2.586233086
```

==============================

4CH

==============================

```
H   4.634065841  11.618441676  -3.395548305
C   2.218993987  11.777736964  -2.169692944
C   2.654181350  12.922622433  -1.491853454
C   0.932971668  11.293860237  -1.888732476
C   1.837175080  13.565274043  -0.566825208
C   0.111433117  11.930907649  -0.967157947
C   0.564421291  13.069667415  -0.304917740
H   3.651261203  13.318394116  -1.689602273
H   0.569319919  10.396579709  -2.393766209
H   2.194772040  14.453345640  -0.048588458
H  -0.883521687  11.539700488  -0.762229255
H  -0.076714032  13.569812162   0.417831567
C   3.414970692   9.109919979  -3.075579141
C   3.244924317   8.625031565  -1.771955511
C   3.697310063   8.185132253  -4.090222054
C   3.355386707   7.267772058  -1.489967467
C   3.808797453   6.827183266  -3.814425713
C   3.637958582   6.367325819  -2.512196878
H   3.011616552   9.320744431  -0.965140482
H   3.820304878   8.532573501  -5.116752055
H   3.216557286   6.910919977  -0.471186374
H   4.025095775   6.124988660  -4.617299731
H   3.720963073   5.304554217  -2.294522212
Si  3.305366222  10.945364103  -3.450056233
C   2.634514990  11.185973195  -5.183503228
C   3.189704644  12.149151276  -6.034725163
C   1.533631315  10.452408196  -5.647791651
C   2.665870509  12.376505417  -7.303133330
H   4.050095722  12.730498156  -5.700530029
C   1.003638329  10.675842727  -6.912736320
H   1.089667381   9.682395227  -5.013975528
C   1.570417929  11.640299462  -7.742108495
H   3.114008106  13.127651707  -7.951043050
H   0.148879845  10.095451717  -7.255411688
H   1.158427962  11.815354973  -8.733682521
```

==============================

5C

==============================

```
C  -3.312073205  -0.300161576  -1.091388748
H  -3.160506183   0.649229544  -0.571447702
H  -4.324198019  -0.668792945  -0.872379114
H  -2.625290462  -1.052259962  -0.679762122
Si -3.083964106  -0.170878431  -2.901809628
C  -3.214562112  -1.666956893  -3.946446819
H  -3.543306229  -2.548643021  -3.390253109
H  -3.895880258  -1.488492493  -4.788425522
H  -2.233816796  -1.882238427  -4.395385980
C  -2.725097208   1.450859340  -3.668510961
H  -2.534348203   1.381205900  -4.742603089
H  -3.573754596   2.130306377  -3.504269799
H  -1.864806566   1.922366920  -3.175267092
```

==============================

5CH

==============================

```
C  -2.461195359  -0.836356324  -1.265559885
H  -1.923133360  -0.091008810  -0.669483684
H  -3.295797284  -1.207938659  -0.658611958
H  -1.781050487  -1.676185619  -1.445262123
```

Si	-3.099994222	-0.103414793	-2.876060889
C	-4.000058611	-1.427527580	-3.864015238
H	-4.850818165	-1.817922091	-3.292389838
H	-4.388637016	-1.030090944	-4.808170789
H	-3.343308872	-2.271778987	-4.100872526
H	-1.950526823	0.410793675	-3.674286976
C	-4.279729520	1.312862634	-2.500014822
H	-3.790146591	2.102609904	-1.919575173
H	-4.671626980	1.765392854	-3.417579929
H	-5.134810068	0.951788928	-1.915655251

==============================

6C

==============================

Si	-2.5804602170	-0.1775466680	-5.6543672730
H	-1.1228450140	-0.2975065800	-5.4975117460
C	-3.3410517410	1.2832272060	-4.9396512490
C	-4.6633841380	1.6870138480	-5.2282784840
C	-2.5557690110	2.0638721320	-4.0622065570
C	-5.1827831390	2.8272776400	-4.6442800740
C	-3.0882651390	3.1986607550	-3.4745087500
C	-4.3979256710	3.5758802770	-3.7654139980
H	-5.2717324740	1.1214123080	-5.9311481600
H	-1.5289496270	1.7763760210	-3.8392007140
H	-6.1963617900	3.1435065380	-4.8729822140
H	-2.4860480860	3.7947208990	-2.7951953150
H	-4.8118792190	4.4711926180	-3.3085715120
C	-3.3967483690	-1.5057069580	-6.5443249550
C	-2.5796520260	-2.4111797070	-7.2572753850
C	-4.7975154340	-1.6849114190	-6.5527302950
C	-3.1514606130	-3.4473438390	-7.9758563280
C	-5.3584227440	-2.7290227140	-7.2641924080
C	-4.5363299050	-3.6026813300	-7.9782621430
H	-1.4962246750	-2.2978111270	-7.2501943560
H	-5.4416644870	-1.0215117410	-5.9791306000
H	-2.5231234480	-4.1386401870	-8.5296665840
H	-6.4349547840	-2.8728608740	-7.2634619840
H	-4.9829423910	-4.4209534190	-8.5373428360

==============================

6CH

==============================

Si	-2.8707374027	-0.4489299645	-6.0672252370
H	-1.7433340685	-1.2463430173	-5.5123137465
C	-2.6286337544	1.3205236417	-5.5017205456
C	-3.2909432224	2.3798117455	-6.1366208721
C	-1.7860949066	1.6212488277	-4.4250935353
C	-3.1245512079	3.6909517258	-5.7065813256
C	-1.6151479756	2.9311059318	-3.9898731561
C	-2.2874883074	3.9667828409	-4.6296047625
H	-3.9395934102	2.1754678640	-6.9889199272
H	-1.2491315616	0.8172711194	-3.9212607754
H	-3.6476294267	4.5004639026	-6.2111973898
H	-0.9568512972	3.1449123460	-3.1507081617
H	-2.1555644682	4.9923864914	-4.2913357564
C	-2.9398806860	-0.5171491552	-7.9377778493
C	-2.0433716118	0.2246394344	-8.7189821254
C	-3.8802451550	-1.3182639543	-8.5960471144
C	-2.0810555483	0.1648015434	-10.1066570789
C	-3.9232708975	-1.3821908527	-9.9851595575
C	-3.0230388465	-0.6397133498	-10.7408808247
H	-1.3139988759	0.8731105032	-8.2325330002
H	-4.5974518500	-1.8955082848	-8.0124365232
H	-1.3813433109	0.7527646852	-10.6967663350
H	-4.6662103519	-2.0046127408	-10.4791940893
H	-3.0569608656	-0.6839295511	-11.8273546933
H	-4.1413786441	-1.0357242630	-5.5606503060

==============================
7C
==============================

Si	-2.5479887380	-0.3145614650	-5.4096191170
H	-1.3127180930	-0.6473519970	-4.6875686640
C	-3.2646287440	-1.5968649030	-6.4861492180
H	-2.5714183780	-1.7864080870	-7.3204887960
H	-3.3410915930	-2.5499712740	-5.9458148760
H	-4.2397341040	-1.3198602760	-6.8945583100
C	-3.2388151800	1.3534471220	-5.1696517340
H	-4.1004186230	1.5578958060	-5.8101005410
H	-3.5396917320	1.4724115180	-4.1175919640
H	-2.4599786550	2.1098522290	-5.3387306370

==============================
7CH
==============================

Si	-2.6300585891	-0.3122951222	-5.4553793504
H	-1.6494986211	-0.6240167101	-4.3798962448
C	-2.3954710358	-1.4996783767	-6.8917459958
H	-1.3988366805	-1.3865917317	-7.3329667226
H	-2.5051243867	-2.5433897333	-6.5798308300
H	-3.1305925682	-1.3082173892	-7.6813123970
C	-2.3981352896	1.4608420390	-6.0299120896
H	-3.1072653435	1.7084858983	-6.8277582671
H	-2.5502779161	2.1762849359	-5.2152502954
H	-1.3880130290	1.6150121266	-6.4253143307
H	-3.9932858784	-0.4808686114	-4.8822027382

==============================
1CAn1 6A‘
==============================

Br	0.447798073	9.890656471	2.635499716
Si	1.492115736	7.948027134	1.605344415
C	1.252429605	6.636739731	2.870908260
H	1.540396452	5.773849010	2.501823425
H	0.303722441	6.587691307	3.111119986
H	1.784031153	6.847773552	3.663565397
Br	-1.753007174	11.000905037	-0.333748877
Br	-1.792595506	14.877610207	-0.315483272
Br	0.443890989	16.011547089	2.667049408
Br	1.833397985	12.874362946	4.359876156
Br	-1.778671145	12.869117737	2.993659019
B	2.465290546	12.083365440	1.282183528
H	3.240713596	11.589176178	1.610453606
B	1.458559275	11.534980774	-0.076880664
H	1.611377358	10.680480003	-0.536714375
B	0.800659001	12.964942932	-0.871371150
B	1.403711677	14.412940025	-0.015569500
H	1.554517031	15.253087997	-0.492008328
B	2.441200972	13.887371063	1.314116359
H	3.226031542	14.394173622	1.636000037
B	0.808675587	11.566850662	1.595125914
B	-0.239158675	12.082560539	0.251389533
B	-0.243348554	13.824429512	0.296095580
B	0.724242508	14.448039055	1.657714367
B	1.419417620	12.963745117	2.479028463
B	-0.234340504	12.918137550	1.827597380
C	2.370114088	12.984553337	-0.179065943
H	3.158750296	13.025039673	-0.761521995
C	0.698419094	7.510064125	-0.109833159
H	-0.234394804	7.009648323	0.046186913
H	0.532292843	8.408994675	-0.665913880
H	1.359598994	6.869992256	-0.655772090
H	0.600741684	12.971549988	-2.034293890
C	3.284849882	8.209227562	1.184062004
C	3.693661928	8.402678490	-0.146269038
C	4.268796444	8.218700409	2.187102556
C	5.037028790	8.598443031	-0.462284386

C	5.612318039	8.414415359	1.871714234
C	6.002873898	8.605242729	0.545341671
H	2.950617552	8.399208069	-0.937683940
H	3.975895643	8.071234703	3.221958637
H	5.329600334	8.745959282	-1.497971535
H	6.355456352	8.417800903	2.664015293
H	7.049599648	8.757749557	0.299367011

==============================

1Ctol

==============================

Si	13.585218430	8.447195053	8.337317467
C	13.995873451	6.681952000	7.919129848
H	13.598903656	6.029396534	8.682664871
H	15.068192482	6.564415455	7.867002964
H	13.558970451	6.427065849	6.964902878
C	12.630499840	10.793341637	5.407455921
C	12.933759689	11.376744270	6.649231434
C	13.272357941	9.590800285	5.066510201
C	13.845367432	10.779767990	7.518377304
C	14.184057236	8.993344307	5.935231209
C	14.474934578	9.585068703	7.165461063
H	12.449050903	12.301161766	6.926559448
H	13.050405502	9.129311562	4.115634441
H	14.063879013	11.245256424	8.468099594
H	14.666080475	8.068941116	5.653217316
H	15.248146057	9.174890518	7.798152447
C	11.918025017	11.620679855	4.321412086
H	11.886797905	11.056090355	3.401268721
H	10.910861969	11.843494415	4.641375065
H	12.456745148	12.542695045	4.159955502
C	14.119755745	8.811719894	10.081161499
H	14.222749710	9.889700890	10.249853134
H	13.393641472	8.431670189	10.808778763
H	15.086895943	8.348389626	10.307156563
C	11.750317574	8.710037231	8.183519363
C	11.142755508	9.874112129	8.683648109
C	10.932362556	7.750162125	7.563906670
C	9.767740250	10.071367264	8.568545341
C	9.557291031	7.946846485	7.448502541
C	8.968363762	9.108541489	7.950341225
H	11.755246162	10.629350662	9.166281700
H	11.380183220	6.843038559	7.170146465
H	9.320116997	10.979255676	8.962727547
H	8.944844246	7.190809727	6.965466976
H	7.897017956	9.262007713	7.860542774

==============================

2CAn1

==============================

Br	0.447798073	9.890656471	2.635499716
Si	1.492115736	7.948027134	1.605344415
C	0.338636935	7.544765949	0.232229784
H	0.731317997	6.837091923	-0.323402643
H	0.193260923	8.344858170	-0.314461410
H	-0.512525201	7.237038612	0.601374090
Br	-1.753007174	11.000905037	-0.333748877
Br	-1.792595506	14.877610207	-0.315483272
Br	0.443890989	16.011547089	2.667049408
Br	1.833397985	12.874362946	4.359876156
Br	-1.778671145	12.869117737	2.993659019
B	2.465290546	12.083365440	1.282183528
H	3.240713596	11.589176178	1.610453606
B	1.458559275	11.534980774	-0.076880664
H	1.611377358	10.680480003	-0.536714375
B	0.800659001	12.964942932	-0.871371150
B	1.403711677	14.412940025	-0.015569500
H	1.554517031	15.253087997	-0.492008328
B	2.441200972	13.887371063	1.314116359
H	3.226031542	14.394173622	1.636000037

B	0.808675587	11.566850662	1.595125914
B	-0.239158675	12.082560539	0.251389533
B	-0.243348554	13.824429512	0.296095580
B	0.724242508	14.448039055	1.657714367
B	1.419417620	12.963745117	2.479028463
B	-0.234340504	12.918137550	1.827597380
C	2.370114088	12.984553337	-0.179065943
H	3.158750296	13.025039673	-0.761521995
H	0.600741684	12.971549988	-2.034293890
C	1.723978043	6.524145603	2.779397964
C	2.311146021	6.716503143	4.041366100
C	1.316941977	5.225587368	2.429541111
C	2.485033274	5.649671078	4.921398163
C	1.490561962	4.158354759	3.309140205
C	2.075512409	4.365351200	4.559419632
H	2.633223295	7.711354733	4.332915306
H	0.860881031	5.053536892	1.459555864
H	2.941444635	5.822363853	5.891899586
H	1.168103456	3.163048267	3.016920567
H	2.210890055	3.533988237	5.244915485
H	2.872819662	8.224914551	1.149943471

==============================
2Ctol
==============================

Si	13.585218430	8.447195053	8.337317467
C	11.751458168	8.575294495	8.053568840
C	11.106348991	7.637079239	7.271194458
H	11.623205185	6.726835728	7.005268574
C	9.809763908	7.853351116	6.827855587
H	9.325370789	7.116695881	6.204075336
C	9.137959480	9.007821083	7.182552338
H	8.121639252	9.164461136	6.852465153
C	9.767314911	9.962518692	7.958842754
H	9.204303741	10.796521187	8.351089478
C	11.077601433	9.752075195	8.373099327
H	11.588804245	10.507070541	8.951935768
C	14.137685776	6.696107864	8.040544510
H	13.734094620	6.055814266	8.810958862
H	15.216480255	6.650261402	8.062944412
H	13.783272743	6.365489483	7.075410843
C	12.630499840	10.793341637	5.407455921
C	12.933759689	11.376744270	6.649231434
C	13.272357941	9.590800285	5.066510201
C	13.845367432	10.779767990	7.518377304
C	14.184057236	8.993344307	5.935231209
C	14.474934578	9.585068703	7.165461063
H	12.449050903	12.301161766	6.926559448
H	13.050405502	9.129311562	4.115634441
H	14.063879013	11.245256424	8.468099594
H	14.666080475	8.068941116	5.653217316
H	15.248146057	9.174890518	7.798152447
C	11.918025017	11.620679855	4.321412086
H	11.886797905	11.056090355	3.401268721
H	10.910861969	11.843494415	4.641375065
H	12.456745148	12.542695045	4.159955502
H	13.896799088	8.833204269	9.731704712

==============================
3CAn2 6B‘ 13A‘
==============================

Br	13.660349846	10.061539650	6.182040215
Br	12.807689667	9.869939804	9.924830437
Br	15.897919655	11.017939568	11.841770172
Br	18.570749283	12.138290405	9.286049843
Br	17.067630768	11.504659653	5.743529797
Br	16.292470932	9.038100243	8.545559883
Si	11.440429688	10.724140167	5.390799999

B	13.349249840	12.629870415	8.309880257
H	12.414449692	12.763680458	8.026579857
B	14.053600311	12.868080139	9.928959846
H	13.529520035	13.234230042	10.682439804
B	15.702039719	13.507740021	9.736169815
H	16.094179153	14.278260231	10.525059700
B	16.023559570	13.648900032	7.993140221
H	16.781799316	14.528240204	7.593780041
B	14.585120201	13.100419998	7.098020077
H	14.310070038	13.587140083	6.216670036
B	14.336079597	11.410849571	7.509180069
B	13.990409851	11.246150017	9.232540131
B	15.436289787	11.800519943	10.100119591
B	16.674009323	12.290189743	8.911870003
B	15.991959572	12.031390190	7.288849831
B	15.607259750	10.871179581	8.602999687
C	14.468029976	13.869469643	8.614809990
C	11.291939735	9.444829941	4.078209877
C	10.949990273	9.813920021	2.791599989
H	10.870070457	10.734430313	2.573230028
C	10.719539642	8.855170250	1.815819979
H	10.474220276	9.122799873	0.938399971
C	10.849630356	7.512629986	2.116810083
H	10.708390236	6.859739780	1.442029953
C	11.184149742	7.121490002	3.399490118
H	11.278929710	6.199500084	3.609999895
C	11.383009911	8.087590218	4.379209995
H	11.581859589	7.817019939	5.268430233
C	10.379440308	10.462389946	6.854070187
H	9.455789566	10.703550339	6.633739948
H	10.697239876	11.022640228	7.591810226
H	10.416609764	9.519820213	7.119659901
C	11.063149452	12.432270050	4.758698940
C	11.951406479	13.095785141	3.895518780
C	9.881788254	13.095785141	5.130805016
C	11.668956757	14.375828743	3.421667099
C	9.598782539	14.375828743	4.657285213
C	10.491142273	15.022023201	3.800348997
H	12.870331764	12.602256775	3.594737530
H	9.180875778	12.602256775	5.796851158
H	12.370655060	14.869244576	2.755208015
H	8.679120064	14.869244576	4.958562374
H	10.270859718	16.019350052	3.431283236
H	14.084432602	14.879042625	8.618786812

==============================

3Ctol

==============================

Si	13.585218430	8.447195053	8.337317467
C	13.786001205	6.688479900	7.766170502
C	12.677451134	5.875174999	7.631362915
H	11.692646980	6.317993641	7.653095245
C	12.818278313	4.504614830	7.469533920
H	11.940876007	3.881122828	7.381087780
C	14.077273369	3.936696529	7.421246052
H	14.184344292	2.871979713	7.275252342
C	15.199401855	4.731342316	7.560986042
H	16.180786133	4.315372944	7.387045860
C	15.048682213	6.099852085	7.753885746
H	15.918334007	6.723996162	7.897237301
C	14.302145004	8.630571365	10.043772697
H	13.696044922	8.082237244	10.749728203
H	14.317384720	9.675189972	10.317526817
H	15.309283257	8.240766525	10.054908752
C	11.777757645	8.884630203	8.373854637
C	11.037784576	8.914140701	7.179744244
C	11.072870255	8.858099937	9.589076042
C	9.644079208	8.917023659	7.199782848
C	9.679174423	8.860968590	9.609762192

C	8.958055496	8.890447617	8.415051460
H	11.564330101	8.934770584	6.237022400
H	11.626642227	8.835242271	10.516014099
H	9.094950676	8.939934731	6.270087719
H	9.157350540	8.840266228	10.555104256
H	7.878260612	8.892556190	8.435976982
C	12.630499840	10.793341637	5.407455921
C	12.933759689	11.376744270	6.649231434
C	13.272357941	9.590800285	5.066510201
C	13.845367432	10.779767990	7.518377304
C	14.184057236	8.993344307	5.935231209
C	14.474934578	9.585068703	7.165461063
H	12.449050903	12.301161766	6.926559448
H	13.050405502	9.129311562	4.115634441
H	14.063879013	11.245256424	8.468099594
H	14.666080475	8.068941116	5.653217316
H	15.248146057	9.174890518	7.798152447
C	11.918025017	11.620679855	4.321412086
H	11.886797905	11.056090355	3.401268721
H	10.910861969	11.843494415	4.641375065
H	12.456745148	12.542695045	4.159955502

===============================

4CAn1 13B‘ 20B‘

===============================

Br	0.447798073	9.890656471	2.635499716
Si	1.492115736	7.948027134	1.605344415
Br	-1.753007174	11.000905037	-0.333748877
Br	-1.792595506	14.877610207	-0.315483272
Br	0.443890989	16.011547089	2.667049408
Br	1.833397985	12.874362946	4.359876156
Br	-1.778671145	12.869117737	2.993659019
B	2.465290546	12.083365440	1.282183528
H	3.240713596	11.589176178	1.610453606
B	1.458559275	11.534980774	-0.076880664
H	1.611377358	10.680480003	-0.536714375
B	0.800659001	12.964942932	-0.871371150
B	1.403711677	14.412940025	-0.015569500
H	1.554517031	15.253087997	-0.492008328
B	2.441200972	13.887371063	1.314116359
H	3.226031542	14.394173622	1.636000037
B	0.808675587	11.566850662	1.595125914
B	-0.239158675	12.082560539	0.251389533
B	-0.243348554	13.824429512	0.296095580
B	0.724242508	14.448039055	1.657714367
B	1.419417620	12.963745117	2.479028463
B	-0.234340504	12.918137550	1.827597380
C	2.370114088	12.984553337	-0.179065943
H	3.158750296	13.025039673	-0.761521995
H	0.600741684	12.971549988	-2.034293890
C	3.269150734	8.228005409	1.132736325
C	4.272579193	8.313184738	2.112638235
C	3.646285057	8.360339165	-0.214330420
C	5.604342937	8.522989273	1.758786559
C	4.977880478	8.570156097	-0.568807483
C	5.963373184	8.652490616	0.416199505
H	4.004234314	8.213837624	3.159818172
H	2.887756109	8.297898293	-0.988410175
H	6.362985611	8.585394859	2.533753633
H	5.245878220	8.669502258	-1.616812587
H	7.000932693	8.815961838	0.140257299
C	0.701740146	7.494690418	-0.016195737
C	0.911215901	8.275605202	-1.165379524
C	-0.121771201	6.361582756	-0.126771763
C	0.319060773	7.936140060	-2.380673409
C	-0.714203954	6.021603107	-1.341786623
C	-0.496574730	6.807372093	-2.474667072
H	1.543824673	9.155615807	-1.103315830
H	-0.297655344	5.743535995	0.748184025

H	0.495376825	8.554920197	-3.255905151
H	-1.347140908	5.140917778	-1.403361797
H	-0.958052218	6.542681694	-3.421437502
C	1.303492665	6.637603760	2.911794424
C	0.491455853	6.852029324	4.038270950
C	1.968989849	5.405121803	2.800287008
C	0.349906385	5.870214462	5.017468452
C	1.827837467	4.422918797	3.779151917
C	1.017514825	4.650826931	4.892547131
H	-0.032259796	7.796876907	4.145268917
H	2.601702929	5.217512131	1.938346624
H	-0.283337861	6.058442593	5.879778862
H	2.352109194	3.477624178	3.671612501
H	0.907383263	3.885708332	5.655345440

==============================

4Ctol

==============================

Si	13.251944542	8.510235786	8.064699173
C	12.630499840	10.793341637	5.407455921
C	12.933759689	11.376744270	6.649231434
C	13.272357941	9.590800285	5.066510201
C	13.845367432	10.779767990	7.518377304
C	14.184057236	8.993344307	5.935231209
C	14.474934578	9.585068703	7.165461063
H	12.449050903	12.301161766	6.926559448
H	13.050405502	9.129311562	4.115634441
H	14.063879013	11.245256424	8.468099594
H	14.666080475	8.068941116	5.653217316
H	15.248146057	9.174890518	7.798152447
C	11.918025017	11.620679855	4.321412086
H	11.886797905	11.056090355	3.401268721
H	10.910861969	11.843494415	4.641375065
H	12.456745148	12.542695045	4.159955502
C	13.297221184	6.791706562	7.354623318
C	14.493937492	6.245114326	6.861229420
C	12.135680199	6.003189564	7.296365261
C	14.528184891	4.957310677	6.329051971
C	12.169292450	4.715321064	6.764304638
C	13.365867615	4.186187267	6.278052807
H	15.403730392	6.836320877	6.896105766
H	11.199723244	6.405048370	7.671811581
H	15.465005875	4.555654049	5.953491688
H	11.258629799	4.124137878	6.729634762
H	13.392303467	3.182787895	5.863459587
C	13.692700386	8.446692467	9.870605469
C	13.627490044	9.599544525	10.671236038
C	14.100330353	7.244472980	10.472966194
C	13.957721710	9.552242279	12.024578094
C	14.430688858	7.196537971	11.826254845
C	14.360945702	8.350351334	12.608599663
H	13.315102577	10.539531708	10.227048874
H	14.158023834	6.341205120	9.873597145
H	13.899826050	10.456380844	12.623901367
H	14.743221283	6.255688667	12.270251274
H	14.618290901	8.313250542	13.663016319
C	11.542920113	9.217475891	7.868106842
C	10.679830551	8.752233505	6.861649036
C	11.078285217	10.232166290	8.721833229
C	9.399063110	9.282028198	6.714076042
C	9.797625542	10.762358665	8.574760437
C	8.951811790	10.289746284	7.570046425
H	11.017685890	7.968739033	6.190490723
H	11.728001595	10.606976509	9.506594658
H	8.749312401	8.906626701	5.928643703
H	9.460027695	11.546351433	9.246616364
H	7.953961849	10.702683449	7.455261707

==============================

5CAn1 12A‘ 19B‘

==============================

Br	0.447798073	9.890656471	2.635499716
Si	1.492115736	7.948027134	1.605344415
C	0.338636935	7.544765949	0.232229784
H	0.731317997	6.837091923	-0.323402643
H	0.193260923	8.344858170	-0.314461410
H	-0.512525201	7.237038612	0.601374090
C	1.436171651	6.862796307	3.055098057
H	1.306696415	7.453362942	3.937915802
Br	-1.753007174	11.000905037	-0.333748877
Br	-1.792595506	14.877610207	-0.315483272
Br	0.443890989	16.011547089	2.667049408
Br	1.833397985	12.874362946	4.359876156
Br	-1.778671145	12.869117737	2.993659019
B	2.465290546	12.083365440	1.282183528
H	3.240713596	11.589176178	1.610453606
B	1.458559275	11.534980774	-0.076880664
H	1.611377358	10.680480003	-0.536714375
B	0.800659001	12.964942932	-0.871371150
B	1.403711677	14.412940025	-0.015569500
H	1.554517031	15.253087997	-0.492008328
B	2.441200972	13.887371063	1.314116359
H	3.226031542	14.394173622	1.636000037
B	0.808675587	11.566850662	1.595125914
B	-0.239158675	12.082560539	0.251389533
B	-0.243348554	13.824429512	0.296095580
B	0.724242508	14.448039055	1.657714367
B	1.419417620	12.963745117	2.479028463
B	-0.234340504	12.918137550	1.827597380
C	2.370114088	12.984553337	-0.179065943
H	3.158750296	13.025039673	-0.761521995
C	3.298810005	8.317811966	1.003085852
H	3.263489246	8.868680954	0.086463772
H	3.811499119	8.893121719	1.745425940
H	3.817916632	7.395586967	0.845194638
H	0.600741684	12.971549988	-2.034293890
H	0.617202401	6.180944920	2.958825350
H	2.351577282	6.313175201	3.124735117

==============================

5Ctol

==============================

Si	13.403890610	8.347096443	8.438190460
C	14.303126335	6.735026360	8.209065437
H	13.704222679	5.936411858	8.665029526
H	15.283724785	6.724080563	8.695558548
H	14.428503990	6.479257107	7.150798321
C	12.799219131	10.823840141	5.320407867
C	13.025666237	11.456334114	6.558573723
C	13.482626915	9.632782936	5.023150921
C	13.900789261	10.917656898	7.473487377
C	14.365496635	9.083914757	5.929168224
C	14.538528442	9.667096138	7.216969013
H	12.518650055	12.394841194	6.773072243
H	13.330727577	9.161337852	4.055030823
H	14.090991020	11.426779747	8.416316986
H	14.913366318	8.177858353	5.677587509
H	15.464352608	9.443844795	7.759763241
C	11.868755341	11.445382118	4.336516857
H	11.677954674	10.770236015	3.465192080
H	10.887453079	11.710326195	4.835471630
H	12.317235947	12.415586472	3.961038589
C	11.735942841	8.374575615	7.614628315
H	11.807680130	8.272651672	6.525555611
H	11.147205353	7.523461342	7.978077412
H	11.174618721	9.285517693	7.846964836
C	13.414294243	8.996092796	10.176874161
H	13.072614670	10.034241676	10.242539406

H	12.716441154	8.389973640	10.768288612
H	14.399612427	8.913768768	10.646001816

==============================
6CAn1
==============================

Br	0.447798073	9.890656471	2.635499716
Si	1.492115736	7.948027134	1.605344415
Br	-1.753007174	11.000905037	-0.333748877
Br	-1.792595506	14.877610207	-0.315483272
Br	0.443890989	16.011547089	2.667049408
Br	1.833397985	12.874362946	4.359876156
Br	-1.778671145	12.869117737	2.993659019
B	2.465290546	12.083365440	1.282183528
H	3.240713596	11.589176178	1.610453606
B	1.458559275	11.534980774	-0.076880664
H	1.611377358	10.680480003	-0.536714375
B	0.800659001	12.964942932	-0.871371150
B	1.403711677	14.412940025	-0.015569500
H	1.554517031	15.253087997	-0.492008328
B	2.441200972	13.887371063	1.314116359
H	3.226031542	14.394173622	1.636000037
B	0.808675587	11.566850662	1.595125914
B	-0.239158675	12.082560539	0.251389533
B	-0.243348554	13.824429512	0.296095580
B	0.724242508	14.448039055	1.657714367
B	1.419417620	12.963745117	2.479028463
B	-0.234340504	12.918137550	1.827597380
C	2.370114088	12.984553337	-0.179065943
H	3.158750296	13.025039673	-0.761521995
H	0.600741684	12.971549988	-2.034293890
C	2.765805006	8.386877060	0.322875679
C	2.599582434	8.007667542	-1.019845843
C	3.921545029	9.107027054	0.669258833
C	3.553885698	8.336386681	-1.981132865
C	4.876203537	9.436041832	-0.291574299
C	4.696887970	9.052233696	-1.621518135
H	1.714243531	7.450650215	-1.310313344
H	4.070872784	9.410450935	1.700810909
H	3.403988123	8.032528877	-3.013222218
H	5.761945724	9.993433952	-0.000401095
H	5.440558434	9.308465004	-2.370314598
C	0.278599620	6.917146683	0.643957973
C	-0.226367921	7.359217167	-0.590510726
C	-0.159202501	5.674194336	1.131534219
C	-1.135765195	6.586919308	-1.311188698
C	-1.068581700	4.901443958	0.411319047
C	-1.561252832	5.354194164	-0.813632250
H	0.097967558	8.316480637	-0.986638963
H	0.217701659	5.312633038	2.083207130
H	-1.512614489	6.949167728	-2.263432026
H	-1.392812848	3.943627834	0.808143973
H	-2.269789934	4.752292633	-1.374958038
H	2.078914404	7.238317490	2.763955593

==============================
6Ctol
==============================

Si	13.585218430	8.447195053	8.337317467
C	12.630499840	10.793341637	5.407455921
C	12.933759689	11.376744270	6.649231434
C	13.272357941	9.590800285	5.066510201
C	13.845367432	10.779767990	7.518377304
C	14.184057236	8.993344307	5.935231209
C	14.474934578	9.585068703	7.165461063
H	12.449050903	12.301161766	6.926559448
H	13.050405502	9.129311562	4.115634441
H	14.063879013	11.245256424	8.468099594
H	14.666080475	8.068941116	5.653217316

H	15.248146057	9.174890518	7.798152447
C	11.918025017	11.620679855	4.321412086
H	11.886797905	11.056090355	3.401268721
H	10.910861969	11.843494415	4.641375065
H	12.456745148	12.542695045	4.159955502
C	14.280906677	8.653497696	10.049935341
C	14.064527512	9.834216118	10.780286789
C	15.037759781	7.633052826	10.650099754
C	14.585733414	9.989111900	12.063710213
C	15.559227943	7.787356377	11.933487892
C	15.335662842	8.966279984	12.646491051
H	13.482661247	10.636007309	10.336409569
H	15.217617035	6.712048531	10.104328156
H	14.405472755	10.910912514	12.609416962
H	16.141407013	6.984742165	12.377204895
H	15.741855621	9.086733818	13.646438599
H	13.766011238	7.045858860	7.896896362
C	11.772601128	8.864156723	8.350060463
C	10.982177734	8.686103821	7.202080250
C	11.154819489	9.366143227	9.507940292
C	9.623809814	8.998476028	7.211319447
C	9.796498299	9.678698540	9.517799377
C	9.024432182	9.496326447	8.369379997
H	11.438333511	8.299506187	6.296029091
H	11.746097565	9.511795998	10.406627655
H	9.032587051	8.852546692	6.311782360
H	9.340524673	10.065519333	10.424697876
H	7.966097355	9.739778519	8.376819611

==============================

7CAn1

==============================

Br	0.447798073	9.890656471	2.635499716
Si	1.492115736	7.948027134	1.605344415
C	1.436171651	6.862796307	3.055098057
H	1.306696415	7.453362942	3.937915802
Br	-1.753007174	11.000905037	-0.333748877
Br	-1.792595506	14.877610207	-0.315483272
Br	0.443890989	16.011547089	2.667049408
Br	1.833397985	12.874362946	4.359876156
Br	-1.778671145	12.869117737	2.993659019
B	2.465290546	12.083365440	1.282183528
H	3.240713596	11.589176178	1.610453606
B	1.458559275	11.534980774	-0.076880664
H	1.611377358	10.680480003	-0.536714375
B	0.800659001	12.964942932	-0.871371150
B	1.403711677	14.412940025	-0.015569500
H	1.554517031	15.253087997	-0.492008328
B	2.441200972	13.887371063	1.314116359
H	3.226031542	14.394173622	1.636000037
B	0.808675587	11.566850662	1.595125914
B	-0.239158675	12.082560539	0.251389533
B	-0.243348554	13.824429512	0.296095580
B	0.724242508	14.448039055	1.657714367
B	1.419417620	12.963745117	2.479028463
B	-0.234340504	12.918137550	1.827597380
C	2.370114088	12.984553337	-0.179065943
H	3.158750296	13.025039673	-0.761521995
C	3.298810005	8.317811966	1.003085852
H	3.263489246	8.868680954	0.086463772
H	3.811499119	8.893121719	1.745425940
H	3.817916632	7.395586967	0.845194638
H	0.600741684	12.971549988	-2.034293890
H	0.617202401	6.180944920	2.958825350
H	2.351577282	6.313175201	3.124735117
H	0.857206762	7.423197269	0.375773638

==============================
7Ctol
==============================

Si	13.382635117	8.339340210	8.394604683
C	13.896575928	8.843928337	10.107615471
H	13.153370857	8.487014771	10.829840660
H	13.959135056	9.931962013	10.218427658
H	14.861706734	8.405991554	10.383521080
C	12.794980049	10.897164345	5.367139339
C	13.112977982	11.477169037	6.611252785
C	13.379517555	9.668327332	5.010582447
C	13.986824989	10.856393814	7.473126411
C	14.260676384	9.036635399	5.860652924
C	14.531075478	9.574521065	7.154073715
H	12.676814079	12.438920975	6.873305321
H	13.154644012	9.236567497	4.038202286
H	14.245438576	11.318746567	8.423905373
H	14.735354424	8.103379250	5.563562393
H	15.468533516	9.284544945	7.642163277
C	11.863044739	11.600569725	4.444270611
H	11.695895195	11.040204048	3.513461590
H	10.886981964	11.773561478	4.935857296
H	12.265930176	12.599362373	4.190233231
H	12.044725418	8.832950592	8.012146950
C	13.671905518	6.557625294	7.971364975
H	14.719235420	6.266115665	8.100814819
H	13.078145027	5.946375847	8.662269592
H	13.353043556	6.306467056	6.955970287

==============================
1CAn2 6A“
==============================

C	8.021757126	10.761763573	-3.281394482
C	8.004054070	11.218891144	-4.605505466
C	7.906239986	9.384282112	-3.041123629
C	7.874057770	10.324755669	-5.659748554
C	7.779691219	8.489935875	-4.093709469
C	7.764840603	8.962154388	-5.403104305
H	8.105237961	12.281694412	-4.819608688
H	7.938719273	9.000131607	-2.021218300
H	7.867685318	10.690756798	-6.683256149
H	7.702628613	7.423951149	-3.895260334
H	7.671892166	8.261642456	-6.229796410
C	7.053943634	11.441577911	-0.420897424
H	7.159897804	10.388776779	-0.139521912
H	7.265349865	12.058264732	0.459463507
H	6.007342339	11.606694221	-0.707205832
Si	8.136898994	11.921060562	-1.858521342
C	8.193369865	13.750068665	-2.165752172
H	7.609805107	14.258999825	-1.390450001
H	9.214311600	14.147714615	-2.147037983
H	7.761391163	13.993015289	-3.143105507
B	13.893969536	11.556344032	-3.638193130
B	13.982066154	13.152444839	-2.866436243
B	14.563460350	12.927792549	-1.208629012
B	14.833851814	11.193738937	-0.955391586
B	14.420634270	10.347216606	-2.457519770
B	12.693355560	10.580378532	-2.777303219
B	12.426569939	12.329428673	-3.029991627
B	12.838431358	13.184480667	-1.517638683
B	13.373180389	11.965435028	-0.323549777
B	13.283541679	10.353273392	-1.103753209
H	15.288613319	13.722634315	-0.718674004
H	15.740745544	10.823599815	-0.293773741
H	15.051695824	9.410327911	-2.806298018
H	14.163883209	11.437695503	-4.783178806
H	14.310076714	14.098199844	-3.495772600
Br	11.596141815	9.216946602	-3.702463865
Br	11.064015388	13.020344734	-4.301564693

Br	11.900723457	14.847253799	-0.978316426
Br	13.082820892	12.207082748	1.617670178
Br	12.886568069	8.712489128	-0.073393174
C	15.061839104	11.913794518	-2.468579531
B	12.081492424	11.593901634	-1.470553994
H	16.085836411	12.024348259	-2.813990355
Br	10.217988968	11.318320274	-0.625089169

==============================
2CAn2
==============================

C	8.250500463	13.734453313	-2.483378451
C	8.126250047	14.621028225	-1.403061017
C	8.348464455	14.250783800	-3.781795577
C	8.112877399	15.991719279	-1.615891903
C	8.329794270	15.622324499	-3.994304749
C	8.217965381	16.489546013	-2.911993126
H	8.055261782	14.236826626	-0.384669169
H	8.449347859	13.578118429	-4.632358574
H	8.027746129	16.672845544	-0.773047201
H	8.411652720	16.016391786	-5.003931045
H	8.213771547	17.564205377	-3.079343150
C	8.229177266	10.712196233	-3.542935579
H	9.081380385	10.854193005	-4.215505491
H	8.268749495	9.691479462	-3.148955837
H	7.300144542	10.829012423	-4.114898127
Si	8.245037725	11.931798330	-2.155042211
B	14.086149531	11.197698691	-3.465553526
B	14.129347056	12.899670019	-2.960335308
B	14.576223356	12.952059344	-1.248869259
B	14.809526303	11.283769260	-0.694556961
B	14.506352123	10.199787447	-2.065195931
B	12.811834774	10.375360348	-2.553976731
B	12.584581934	12.057125715	-3.109968249
B	12.881613832	13.152925441	-1.729593589
B	13.311635951	12.145340273	-0.314091621
B	13.268295602	10.425696954	-0.824172313
H	15.270301648	13.817728389	-0.840983593
H	15.657712732	11.029776070	0.088500572
H	15.152202881	9.219497898	-2.204093287
H	14.441912780	10.893621709	-4.551269446
H	14.514134028	13.728524022	-3.710528383
Br	11.760785954	8.882660545	-3.326843413
Br	11.352490669	12.535008051	-4.595492123
Br	11.915983539	14.871775594	-1.548121335
Br	12.871537399	12.700708586	1.531714973
Br	12.777776125	8.975938706	0.427258966
C	15.161864399	11.745370266	-2.282152574
B	12.112258734	11.586491641	-1.483535388
H	16.210694887	11.800957599	-2.559588880
Br	10.189210493	11.395029967	-0.743597344
H	7.363064173	11.582500274	-1.022566836

==============================
3CAn2 6B“ 13A“
==============================

C	7.957245157	10.577114817	-3.473219923
C	8.426352907	10.907684960	-4.753654493
C	7.456517166	9.284651546	-3.251767505
C	8.372524137	9.980704201	-5.785532226
C	7.416678298	8.354088378	-4.280493047
C	7.871325227	8.705180590	-5.548104051
H	8.868778139	11.885834045	-4.937212938
H	7.098341930	8.997076262	-2.263593953
H	8.745974594	10.247023850	-6.771194813
H	7.033775958	7.353673634	-4.094711860
H	7.842663568	7.975366861	-6.354209139
C	6.958381210	11.271817611	-0.604210604
H	7.218338158	10.284978361	-0.206957133

H	7.065854642	12.007344338	0.200771138
H	5.904126037	11.255529027	-0.907514163
Si	7.991816050	11.766495061	-2.072382222
B	13.934516770	11.903675563	-3.505385219
B	14.015522621	13.376243225	-2.519482599
B	14.514252792	12.913220738	-0.884089578
B	14.741073990	11.156076228	-0.857625176
B	14.385620372	10.533058975	-2.477759273
B	12.680630316	10.847994755	-2.837633125
B	12.451705059	12.623580852	-2.862311637
B	12.812065202	13.253686759	-1.228363367
B	13.264055864	11.868806174	-0.192565019
B	13.184969820	10.381553813	-1.187133865
H	15.228537420	13.617622539	-0.258469533
H	15.609158151	10.678495237	-0.212740418
H	15.014253280	9.637007211	-2.924640934
H	14.258127022	11.932767028	-4.642426862
H	14.391940342	14.390280533	-2.997223228
Br	11.622187866	9.626465448	-3.981890529
Br	11.146736038	13.522938081	-4.061863654
Br	11.919062771	14.862023014	-0.487913696
Br	12.888168979	11.848871822	1.749696151
Br	12.721016491	8.624990575	-0.405936148
C	15.053562358	12.069459188	-2.247425105
B	12.019565016	11.689494020	-1.437760652
H	16.094509482	12.201327763	-2.528527990
Br	10.104513626	11.369178664	-0.741382823
C	7.941850534	13.562443028	-2.475624495
C	8.386038024	14.531794448	-1.566356481
C	7.395537335	13.980339299	-3.698625867
C	8.315080084	15.881200706	-1.883296871
C	7.316455463	15.331852670	-4.010011376
C	7.784735040	16.280371231	-3.106188509
H	8.834903058	14.234571341	-0.617900214
H	7.037715558	13.245609772	-4.419088095
H	8.691463184	16.620854066	-1.180667169
H	6.898574760	15.644424608	-4.964098573
H	7.739270567	17.337541972	-3.358151525

==============================

4CAn2 13B“ 20B“

==============================

C	8.210325241	13.834952354	-2.808259964
C	8.015945435	14.829474449	-1.835089803
C	8.594464302	14.224206924	-4.099782944
C	8.164151192	16.171545029	-2.153834581
C	8.735403061	15.568127632	-4.419736862
C	8.514811516	16.540369034	-3.449746847
H	7.763094425	14.550800323	-0.811899304
H	8.809329033	13.470597267	-4.855725765
H	8.017395020	16.930551529	-1.389235854
H	9.035779953	15.856209755	-5.424169540
H	8.633686066	17.592338562	-3.699714661
Si	8.008200645	12.065782547	-2.340574741
B	14.066254616	10.755687714	-3.008857489
B	14.060495377	12.527195930	-2.948365927
B	14.364192963	13.017896652	-1.273634791
B	14.553152084	11.552500725	-0.297771066
B	14.372723579	10.154800415	-1.370605826
B	12.728135109	10.156119347	-2.021674156
B	12.530056953	11.637539864	-3.005222082
B	12.717980385	13.051329613	-1.923017383
B	13.024377823	12.443010330	-0.270945460
B	13.028823853	10.653846741	-0.332251638
H	15.015724182	13.977846146	-1.045218229
H	15.333417892	11.523726463	0.589971542
H	15.028280258	9.184511185	-1.206495881
H	14.516440392	10.193513870	-3.946876287
H	14.507068634	13.153895378	-3.846378326

Br	11.832486153	8.439877510	-2.457650900
Br	11.378562927	11.685427666	-4.624229908
Br	11.791244507	14.769332886	-2.243823290
Br	12.435462952	13.434268951	1.336494803
Br	12.440845490	9.553266525	1.204112291
C	15.038346291	11.608381271	-1.918376803
B	11.922747612	11.581838608	-1.357124567
H	16.106733322	11.618045807	-2.115029812
Br	9.934174538	11.528909683	-0.794010222
C	6.725944519	11.765705109	-1.028436184
C	5.682075024	12.688148499	-0.856715679
C	6.696147442	10.575531006	-0.284062117
C	4.651615143	12.436032295	0.041848075
C	5.673049450	10.329940796	0.620927453
C	4.650714874	11.261359215	0.785316169
H	5.669595242	13.612904549	-1.432605028
H	7.483139515	9.829927444	-0.403696328
H	3.850159883	13.161884308	0.163488328
H	5.672913551	9.408884048	1.199457884
H	3.849066734	11.067758560	1.494365692
C	8.027348518	10.799176216	-3.684463024
C	8.530107498	9.505068779	-3.509073973
C	7.432032108	11.130061150	-4.912550449
C	8.474762917	8.578899384	-4.541574478
C	7.372216225	10.199464798	-5.942886353
C	7.901453972	8.925921440	-5.760993481
H	9.018089294	9.221171379	-2.577092171
H	7.013738632	12.123599052	-5.073700905
H	8.898847580	7.587995529	-4.395305157
H	6.917505264	10.473418236	-6.892204285
H	7.868637085	8.202820778	-6.572475433

==============================

5CAn2 12A“ 19B“

==============================

Si	8.368066796	10.401358520	-0.597744282
C	7.856900153	10.544311562	-2.370244595
H	6.926498214	9.981093895	-2.519302991
H	7.667828240	11.592553432	-2.627155452
H	8.616205838	10.152040116	-3.053983663
B	13.512941403	10.900454659	-3.680056648
B	13.831059746	12.601412634	-3.308622736
B	14.626763497	12.674912756	-1.725763006
B	14.800113446	11.017871836	-1.118932602
B	14.112289583	9.921040251	-2.325805844
B	12.386391669	10.265200784	-2.477076297
B	12.203746508	11.934194166	-3.087638820
B	12.902849363	13.044666162	-1.871298712
B	13.508294844	12.055296355	-0.503203167
B	13.181356396	10.336604507	-0.878705597
H	15.471882306	13.475335576	-1.520897642
H	15.762213050	10.703168399	-0.508562974
H	14.602920393	8.865271332	-2.531434567
H	13.604583559	10.497090993	-4.787554930
H	14.142534351	13.350203145	-4.168592414
Br	11.145097084	8.795220345	-2.989021967
Br	10.678209046	12.458732106	-4.238726670
Br	12.208133850	14.876937899	-1.610594680
Br	13.519298134	12.732633598	1.353906343
Br	12.791037957	9.005851600	0.536883536
C	14.860894592	11.369128503	-2.773652194
B	12.040670920	11.587010906	-1.367980322
H	15.829857934	11.291651050	-3.258595564
Br	10.279342246	11.934838753	-0.336526275
C	8.945437855	8.777673327	0.075813397
H	8.102255927	8.074938948	0.086377289
H	9.752705925	8.344037897	-0.522749971
H	9.303308395	8.899775176	1.104183489
C	7.265288555	11.341142854	0.571731865

```
H   6.294980567  10.832806156   0.638508703
H   7.689019295  11.384870906   1.580791376
H   7.083171758  12.363544961   0.223715164
```

==============================
6CAn2
==============================

```
C   7.402680428  10.284839944  -1.875122560
C   7.366303578   9.441629575  -2.994649827
C   6.794916657   9.860630974  -0.682896533
C   6.722735978   8.211837791  -2.928573099
C   6.159095715   8.629122380  -0.616780270
C   6.121660580   7.807742720  -1.741158996
H   7.853838126   9.745901063  -3.920848770
H   6.827970538  10.495179273   0.204026850
H   6.699665574   7.563502441  -3.801145035
H   5.696883263   8.304126742   0.312365716
H   5.626209051   6.840949334  -1.687264444
Si  8.286700952  11.893612830  -1.993766285
B  13.725893934  11.944952897  -3.697351054
B  14.175055195  13.174561168  -2.500925068
B  14.772398497  12.341989678  -1.055835574
B  14.697188360  10.596266857  -1.359312873
B  14.052971626  10.351407492  -2.991077617
B  12.398610380  10.978944075  -3.038776828
B  12.475416934  12.740869624  -2.733114129
B  13.126225629  12.989914582  -1.088903424
B  13.452136974  11.379672087  -0.375880947
B  13.003239440  10.134968163  -1.583591683
H  15.665723591  12.800029669  -0.431850113
H  15.540557705   9.882474871  -0.939187784
H  14.458676278   9.473929886  -3.671630785
H  13.911518179  12.137351846  -4.848939133
H  14.661066314  14.193436169  -2.852046621
Br 11.036716196  10.151583838  -4.227334139
Br 11.215206785  14.040528125  -3.558683735
Br 12.582953115  14.539885928   0.017664726
Br 13.295224962  11.049194622   1.567258352
Br 12.321273136   8.356797442  -1.055866244
C  14.996163248  11.700916521  -2.606232451
B  12.063142992  11.623256854  -1.437079448
H  16.003772397  11.727735644  -3.011186252
Br 10.229460324  11.533135460  -0.498145441
H   8.938571640  12.124633304  -3.286773247
C   7.492834059  13.327525257  -1.143982636
C   6.127976819  13.265287239  -0.822575224
C   8.203679076  14.506478695  -0.867510256
C   5.489418037  14.353725546  -0.240144658
C   7.562489137  15.589897903  -0.283396241
C   6.206944999  15.514151548   0.029904500
H   5.558223174  12.359856090  -1.031244872
H   9.266879979  14.578976459  -1.103198088
H   4.431316942  14.293829907   0.004921955
H   8.124044303  16.495788491  -0.068650896
H   5.708903202  16.364476049   0.490065981
```

==============================
7CAn2
==============================

```
Si  8.136898994  11.921060562  -1.858521342
B  13.893969536  11.556344032  -3.638193130
B  13.982066154  13.152444839  -2.866436243
B  14.563460350  12.927792549  -1.208629012
B  14.833851814  11.193738937  -0.955391586
B  14.420634270  10.347216606  -2.457519770
B  12.693355560  10.580378532  -2.777303219
B  12.426569939  12.329428673  -3.029991627
B  12.838431358  13.184480667  -1.517638683
B  13.373180389  11.965435028  -0.323549777
```

B	13.283541679	10.353273392	-1.103753209
H	15.288613319	13.722634315	-0.718674004
H	15.740745544	10.823599815	-0.293773741
H	15.051695824	9.410327911	-2.806298018
H	14.163883209	11.437695503	-4.783178806
H	14.310076714	14.098199844	-3.495772600
Br	11.596141815	9.216946602	-3.702463865
Br	11.064015388	13.020344734	-4.301564693
Br	11.900723457	14.847253799	-0.978316426
Br	13.082820892	12.207082748	1.617670178
Br	12.886568069	8.712489128	-0.073393174
C	15.061839104	11.913794518	-2.468579531
B	12.081492424	11.593901634	-1.470553994
H	16.085836411	12.024348259	-2.813990355
Br	10.217988968	11.318320274	-0.625089169
H	8.150233269	12.491481781	-3.224113703
C	7.510198116	13.337146759	-0.828206778
H	6.500187874	13.635159492	-1.131812334
H	7.470396042	13.070710182	0.234118626
H	8.156678200	14.216935158	-0.923836648
C	7.234705448	10.310049057	-2.082768440
H	7.294856548	9.959991455	-3.119566679
H	7.655459404	9.524866104	-1.444363594
H	6.173164368	10.408473015	-1.828696847

==============================

HB

==============================

H	-1.276532650	0.281050414	-1.913004279
C	-0.235942662	-1.936623931	-3.004189730
H	0.461550206	-2.021986485	-2.164046288
H	-0.431268960	-2.950150967	-3.374453306
H	0.249904290	-1.376176000	-3.811054707
Si	-1.839155912	-1.139718771	-2.515994072
C	-2.935887337	-0.568426788	-3.898745537
H	-2.346779346	-0.168973044	-4.732090473
H	-3.527877808	-1.412610173	-4.272422314
H	-3.636975050	0.208475769	-3.571310759
C	-0.571075320	2.014848232	-3.917828083
C	0.446768403	1.457890868	-4.708979607
C	-1.604345441	2.722030640	-4.551843643
C	0.420226187	1.585317135	-6.091850758
C	-1.626990199	2.856836557	-5.934386253
C	-0.619424462	2.281410217	-6.703754425
H	1.276080966	0.924924552	-4.242466927
H	-2.402148008	3.174044847	-3.962534428
H	1.213231206	1.149941921	-6.694078922
H	-2.429880619	3.410635471	-6.413714409
H	-0.639690936	2.382577896	-7.785757065
C	1.034819961	1.443572998	-1.275003433
H	0.899970591	1.114492416	-0.239167020
H	1.623669267	0.686107695	-1.803402781
H	1.624911308	2.368054867	-1.258699775
Si	-0.599355221	1.759700298	-2.094911337
C	-1.857131958	2.750435829	-1.157088161
H	-2.869163752	2.585499525	-1.544637680
H	-1.853272319	2.485917807	-0.095086440
H	-1.638222933	3.821675301	-1.240389228
C	-2.826726198	-2.333072662	-1.486328483
H	-3.800701141	-1.912171245	-1.211877465
H	-3.011810064	-3.268276215	-2.026926279
H	-2.303248167	-2.586789608	-0.557508230

9.7 Vibrational Frequencies (cm^{-1}) of the Calculated Structures

```
==============================
00-Carborate
==============================
   68.47    68.69    75.66    75.76    79.72    79.84
   88.02    88.26   100.73   193.75   193.83   194.42
  239.92   239.96   253.78   302.92   329.55   329.75
  541.13   541.26   582.92   583.07   597.45   609.62
  609.75   637.80   638.09   645.67   712.62   712.77
  740.69   740.76   764.59   792.15   792.32   812.16
  812.22   816.03   816.12   821.89   824.62   824.77
  846.80   847.03   859.50   859.62   903.58   905.49
  905.70   905.76   940.47   940.62   947.46   947.82
  956.72   982.34   982.38  1058.72  1119.37  1119.42
 2711.00  2711.35  2716.75  2716.89  2723.44  3204.82
```

```
==============================
1A
==============================
   29.05    83.74   131.57   145.02   152.73   177.02
  230.75   287.20   380.94   398.96   466.82   602.96
  624.44   648.02   693.84   705.08   710.71   728.17
  737.73   778.27   852.55   864.06   899.77   901.39
  924.73   973.17   997.55  1006.30  1056.48  1098.69
 1139.87  1162.53  1195.40  1275.82  1280.78  1316.18
 1364.74  1434.03  1436.75  1443.45  1447.96  1461.09
 1517.15  1641.94  1665.60  2253.18  3036.55  3037.69
 3128.31  3129.49  3134.41  3143.43  3144.23  3151.49
 3168.38  3178.74  3190.75
```

```
==============================
2A
==============================
   56.42    56.94    74.70    76.86    77.73   113.44
  232.98   235.59   246.04   287.09   324.64   325.14
  408.20   408.86   428.15   429.01   471.87   472.74
  612.54   613.20   613.67   626.85   627.72   665.69
  690.98   691.56   711.44   718.12   776.48   776.78
  813.50   854.04   854.87   857.09   932.26   933.48
  962.58   967.18   968.12   991.44   992.19   993.36
 1004.36  1004.44  1005.19  1021.35  1021.61  1022.27
 1054.33  1055.41  1055.46  1115.52  1119.60  1120.43
 1173.75  1173.84  1174.81  1193.70  1194.20  1197.50
 1224.79  1314.63  1321.14  1322.13  1380.68  1382.27
 1395.03  1410.66  1411.23  1472.05  1482.51  1483.15
 1514.45  1523.24  1523.52  1621.05  1624.18  1624.55
 1656.07  1656.78  1670.97  3190.22  3190.41  3190.56
 3194.87  3195.93  3196.49  3198.50  3198.57  3199.68
 3208.43  3208.69  3209.00  3212.95  3213.11  3213.50
```

```
==============================
3A
==============================
    6.71    21.97    34.28    38.87    45.48    52.21
   61.71    69.03    71.43    79.56    80.65    85.58
  108.46   122.55   130.48   140.05   168.84   186.53
  235.52   237.00   245.51   249.51   276.48   285.66
  322.60   324.63   373.92   400.25   407.68   410.78
  427.51   430.75   458.63   471.59   474.29   607.16
  609.41   611.15   612.35   623.90   624.82   625.95
  647.20   665.05   689.84   691.72   696.89   701.69
  705.95   708.69   710.51   719.08   769.65   774.69
  775.50   776.67   811.74   849.45   851.19   852.95
  856.64   867.91   901.61   909.04   926.68   928.54
  931.07   959.93   964.45   969.47   974.50   989.33
  989.77   994.35   998.05  1002.26  1002.89  1003.30
 1007.53  1017.68  1019.09  1019.72  1051.82  1053.97
```

```
 1054.65  1055.70  1098.90  1114.58  1118.37  1120.71
 1136.33  1164.50  1170.78  1171.75  1172.86  1192.79
 1193.57  1195.41  1196.59  1221.55  1281.42  1287.79
 1314.20  1314.44  1320.76  1321.06  1363.19  1377.77
 1381.55  1394.44  1408.65  1410.78  1432.40  1435.75
 1442.75  1446.62  1458.56  1469.97  1480.94  1481.71
 1513.09  1514.37  1521.53  1522.65  1620.25  1624.27
 1625.41  1637.19  1656.51  1657.73  1660.65  1670.75
 2199.72  3038.62  3042.67  3132.16  3135.81  3137.31
 3139.82  3142.08  3157.81  3172.92  3180.77  3187.64
 3188.60  3190.62  3192.96  3194.51  3195.07  3197.90
 3199.36  3200.18  3201.24  3205.76  3206.25  3209.48
 3212.12  3212.35  3213.48
```

==============================

3A-TS

==============================

```
 -425.05    11.46    29.52    32.46    50.48    58.71
   65.47    69.57    79.26    84.07    87.02   114.58
  120.99   131.20   135.98   147.72   161.02   193.95
  220.77   244.69   248.46   258.90   278.83   294.25
  318.73   321.60   322.01   388.64   395.97   408.18
  408.99   427.76   456.76   487.81   487.98   496.00
  614.21   614.79   619.61   620.42   627.19   628.78
  647.99   677.57   693.09   699.18   701.23   703.25
  708.52   718.04   726.06   744.86   755.61   767.88
  769.41   796.12   813.25   849.88   852.86   857.13
  857.47   863.55   914.02   915.34   935.60   943.45
  947.76   951.22   980.22   981.82   982.79   986.20
  998.24  1001.07  1003.37  1007.15  1008.55  1012.45
 1012.83  1014.06  1015.25  1018.67  1054.88  1055.65
 1058.15  1059.16  1103.81  1106.17  1114.23  1115.79
 1121.51  1141.82  1169.16  1169.61  1169.90  1170.21
 1190.21  1190.56  1196.27  1197.45  1280.96  1282.57
 1298.44  1304.27  1306.60  1315.13  1340.28  1343.51
 1354.16  1370.99  1389.73  1391.77  1411.20  1417.81
 1420.92  1429.12  1434.17  1461.46  1474.09  1483.46
 1484.24  1511.87  1520.83  1521.63  1534.55  1634.61
 1639.01  1642.30  1642.70  1658.06  1664.01  1664.23
 1679.79  3034.91  3039.82  3129.93  3134.22  3139.36
 3141.95  3143.49  3154.76  3162.45  3166.75  3170.07
 3180.89  3187.79  3188.36  3188.77  3190.53  3196.45
 3196.92  3197.15  3200.18  3203.26  3204.48  3204.66
 3209.79  3211.73  3212.02
```

==============================

4A

==============================

```
    6.11    10.16    16.05    37.50    49.86    53.29
   63.91    68.23    69.92    81.97    96.31   108.31
  125.96   141.62   142.75   157.10   166.22   202.50
  228.73   238.16   244.12   245.79   273.30   293.69
  305.21   313.96   375.39   393.49   407.45   408.60
  418.06   443.11   464.81   491.62   495.55   610.75
  611.86   616.16   619.99   624.61   627.63   641.95
  672.02   692.59   700.74   704.64   707.16   708.66
  728.51   734.53   739.48   749.14   760.44   762.82
  791.20   818.85   845.32   852.64   855.92   858.04
  860.66   869.18   887.66   890.10   926.58   937.59
  941.16   945.72   971.78   980.10   982.76   984.15
 1003.11  1005.33  1006.23  1007.21  1008.67  1009.62
 1011.68  1020.51  1054.98  1055.69  1057.22  1058.40
 1090.94  1095.72  1108.87  1110.00  1115.48  1116.31
 1139.24  1166.41  1167.56  1168.07  1173.59  1184.61
 1189.55  1191.00  1196.17  1199.62  1271.59  1274.82
 1281.09  1286.30  1318.13  1318.33  1329.90  1332.88
 1373.80  1378.87  1379.53  1383.24  1408.32  1416.39
 1417.85  1423.92  1462.26  1474.13  1482.80  1483.42
 1509.09  1521.95  1525.57  1526.70  1632.22  1644.94
```

1652.03 1654.35 1654.59 1668.11 1671.64 1673.71
2174.53 3029.78 3032.68 3121.84 3122.04 3140.45
3142.23 3147.98 3150.74 3156.44 3160.38 3167.11
3174.15 3179.44 3183.45 3183.95 3184.88 3188.78
3189.45 3192.62 3195.68 3197.39 3200.59 3200.84
3203.20 3205.66 3207.41

==============================
5A
==============================
21.99 25.45 50.30 59.91 60.26 78.62
218.42 234.25 234.80 270.70 299.69 300.47
408.26 408.68 413.27 474.09 499.78 501.06
609.21 609.98 620.77 623.03 626.54 663.84
707.68 708.30 710.10 740.52 763.22 763.91
833.34 852.02 852.55 854.73 879.18 880.30
924.46 925.21 926.68 967.04 967.63 968.34
990.92 991.39 991.84 1008.76 1009.34 1009.57
1058.44 1060.09 1060.38 1102.72 1102.93 1105.84
1160.89 1161.21 1161.35 1187.13 1187.53 1188.89
1199.06 1212.85 1213.66 1289.56 1290.06 1322.70
1341.99 1342.02 1372.26 1383.33 1383.60 1479.77
1486.18 1486.29 1530.04 1530.79 1530.80 1655.40
1659.93 1660.18 1677.86 1677.89 1683.32 3002.76
3153.70 3153.82 3154.10 3167.67 3167.76 3168.50
3175.71 3175.99 3177.18 3183.30 3183.56 3184.58
3194.20 3194.35 3194.83
==============================
6A
==============================
81.77 109.88 132.62 229.86 279.93 306.08
348.19 388.18 391.50 428.38 515.00 612.19
648.87 679.04 751.83 753.60 768.89 784.71
853.41 862.27 884.12 944.80 954.94 991.08
1001.92 1029.87 1051.92 1115.00 1150.42 1177.72
1203.59 1310.00 1324.00 1352.78 1390.20 1420.22
1425.67 1445.34 1445.98 1463.65 1503.85 1621.80
1651.79 2720.91 2829.99 2885.01 2904.70 2995.17
3046.45 3172.12 3176.98 3193.31 3208.10 3212.54

==============================
7A
==============================
14.75 21.52 33.91 46.49 51.69 59.65
112.62 115.21 126.06 131.33 137.44 147.21
151.34 153.68 160.59 188.90 199.18 205.96
235.06 279.88 294.26 306.66 372.45 390.41
396.47 398.29 449.30 462.05 614.37 619.25
620.22 644.85 649.46 698.01 701.03 703.95
720.78 723.98 726.15 743.69 747.23 752.52
769.19 804.64 809.10 833.05 859.47 863.14
864.39 864.65 875.48 939.60 940.76 969.37
984.69 984.80 1003.10 1005.13 1018.99 1019.91
1053.51 1055.55 1106.23 1108.23 1138.87 1141.62
1171.75 1172.16 1198.29 1199.71 1283.49 1287.39
1288.93 1294.54 1317.21 1317.61 1369.43 1371.90
1414.87 1417.49 1419.10 1422.25 1426.37 1427.72
1431.36 1434.16 1461.65 1462.71 1511.24 1512.88
1632.34 1636.21 1655.94 1657.49 1851.96 3039.59
3041.51 3043.21 3043.72 3135.78 3137.78 3138.05
3138.45 3143.08 3145.13 3146.73 3148.96 3151.18
3152.63 3152.85 3159.91 3181.23 3186.79 3188.76
3197.99 3200.81 3206.82

==============================

7A-TS

==============================

-122.95	14.74	28.80	47.14	66.82	72.16
79.85	95.33	114.88	124.83	140.72	149.89
154.47	161.05	167.55	178.01	189.32	217.62
235.31	252.26	285.01	293.48	367.58	379.17
393.49	399.95	439.47	450.24	615.76	621.48
621.67	642.53	649.76	675.94	691.60	697.48
705.35	714.78	722.82	729.47	751.31	753.56
759.98	787.15	807.29	824.71	854.85	859.32
871.40	875.72	906.79	916.13	941.37	948.51
987.19	988.83	1002.62	1004.44	1015.76	1024.37
1052.84	1053.22	1101.17	1111.29	1132.66	1140.41
1169.14	1174.47	1196.60	1200.54	1277.74	1283.66
1285.39	1290.10	1314.79	1317.84	1365.88	1378.17
1398.46	1408.10	1410.86	1422.46	1425.76	1428.82
1436.42	1437.67	1458.71	1462.72	1507.54	1510.66
1628.38	1629.76	1651.66	1655.02	2129.16	3026.05
3032.11	3035.48	3043.27	3125.32	3126.23	3127.53
3129.49	3140.73	3141.67	3148.81	3153.73	3157.70
3159.92	3160.57	3161.54	3179.31	3186.16	3187.04
3196.23	3198.43	3204.36			

==============================

8A

==============================

19.41	28.38	39.60	42.56	63.49	110.16
123.91	135.22	141.15	155.55	164.91	171.99
173.58	186.11	193.19	203.67	216.31	223.82
251.27	264.30	304.74	308.18	340.06	386.74
396.26	397.85	466.87	480.81	607.01	615.54
620.62	635.68	655.80	673.65	688.48	700.62
703.22	713.11	715.82	737.38	745.37	749.76
776.03	783.79	802.07	814.78	853.75	860.21
860.78	875.12	889.23	908.87	934.33	978.39
994.55	999.59	1004.26	1012.53	1013.81	1022.86
1037.39	1055.99	1056.27	1104.46	1106.65	1137.09
1169.85	1177.22	1197.99	1199.12	1282.92	1286.08
1291.76	1292.93	1300.92	1317.20	1369.34	1380.77
1421.86	1423.17	1426.05	1428.88	1432.40	1435.85
1440.73	1441.86	1460.91	1463.67	1484.55	1515.30
1608.79	1639.82	1644.55	1660.81	2304.88	3038.42
3040.35	3044.43	3045.65	3130.54	3133.08	3135.37
3135.51	3148.64	3150.02	3152.16	3153.63	3155.89
3157.46	3158.23	3165.10	3184.46	3190.12	3193.96
3203.39	3206.18	3210.28			

==============================

8A-TS

==============================

-169.80	19.53	37.24	38.62	45.65	105.61
118.44	131.21	133.06	139.21	145.01	170.33
174.12	188.56	199.19	202.14	210.26	214.08
222.15	233.10	291.63	320.08	342.85	381.33
396.80	403.82	451.45	480.57	612.08	621.93
623.45	638.55	643.21	659.88	677.17	688.75
689.59	706.83	714.21	737.78	742.87	748.60
759.78	790.32	797.30	821.46	849.07	857.54
863.42	886.64	893.49	911.12	931.12	975.54
978.32	995.32	1003.62	1009.41	1012.56	1029.82
1044.30	1055.40	1083.06	1101.83	1105.64	1138.52
1169.26	1174.06	1196.22	1198.69	1284.38	1285.25
1291.39	1296.06	1302.54	1316.96	1365.12	1377.27
1416.29	1419.49	1424.59	1428.78	1432.53	1434.95
1438.82	1442.99	1444.72	1461.92	1490.96	1514.68
1604.45	1636.17	1641.54	1660.37	2326.48	3035.55
3039.40	3042.47	3045.70	3128.79	3131.66	3134.25
3134.48	3135.19	3138.69	3140.70	3144.62	3155.22

3158.23 3170.75 3175.38 3177.42 3183.30 3190.46
3195.57 3198.67 3207.75

==============================
9A
==============================
12.33 22.70 39.30 62.04 86.39 95.33
107.66 129.93 135.46 148.41 160.97 170.13
178.27 186.13 189.32 194.99 205.90 216.14
233.14 252.75 302.60 325.70 350.59 395.16
407.13 441.16 463.57 488.91 612.93 622.37
628.38 642.55 662.39 674.53 689.34 694.83
706.66 709.44 711.38 715.22 740.78 753.16
766.83 788.17 795.32 821.77 845.89 854.32
861.76 875.16 911.19 915.14 929.77 952.54
976.19 993.79 1004.56 1011.62 1014.33 1035.24
1046.86 1057.18 1073.43 1102.64 1104.19 1139.38
1169.27 1173.59 1190.41 1197.65 1281.04 1289.34
1289.85 1294.05 1302.95 1317.27 1368.57 1391.16
1418.44 1422.16 1428.17 1430.61 1432.08 1433.57
1436.15 1440.61 1441.99 1461.85 1491.24 1516.15
1590.10 1638.54 1640.35 1662.07 2333.21 3037.26
3037.75 3043.62 3048.64 3087.07 3131.16 3131.71
3137.30 3137.68 3137.88 3139.74 3140.00 3149.65
3157.84 3163.34 3165.44 3182.35 3186.21 3189.41
3191.69 3201.81 3212.36

==============================
9A-TS
==============================
-105.48 25.81 35.31 47.87 56.33 62.28
71.23 103.80 108.74 128.70 136.17 148.95
162.14 169.83 174.18 187.25 208.36 214.12
243.06 248.14 270.26 359.00 396.58 401.89
421.20 445.66 467.11 477.10 527.68 608.09
622.85 623.50 630.98 634.72 674.83 682.93
685.39 701.72 708.37 712.84 735.29 741.12
756.21 766.38 793.13 809.32 831.80 851.21
861.45 871.82 878.84 917.07 927.76 941.20
977.14 988.26 1004.06 1004.31 1012.08 1015.37
1055.63 1058.45 1100.99 1105.51 1133.67 1143.20
1168.44 1169.65 1197.01 1199.55 1204.06 1286.03
1287.27 1294.37 1312.88 1317.96 1365.19 1367.73
1396.57 1400.80 1407.79 1413.71 1423.30 1428.46
1430.30 1457.92 1461.39 1510.24 1513.39 1519.34
1636.15 1639.50 1658.77 1662.96 2392.05 2798.27
3029.11 3038.32 3040.59 3064.55 3122.79 3123.62
3131.27 3137.07 3139.86 3141.73 3143.54 3145.58
3152.37 3154.62 3161.22 3182.70 3183.31 3190.89
3192.13 3202.01 3203.16

==============================
10A
==============================
10.32 14.53 26.51 32.21 44.69 55.96
83.95 109.25 135.86 144.00 148.89 155.15
163.29 170.15 199.50 205.79 218.59 223.56
240.08 263.87 271.48 305.24 366.82 393.17
398.32 446.90 459.03 473.92 528.04 617.86
619.75 620.87 656.51 673.85 691.86 701.49
705.03 706.61 717.12 739.92 746.45 752.62
766.08 777.80 808.19 814.33 858.45 859.92
864.71 885.53 905.72 934.70 942.46 980.79
985.56 997.79 1003.37 1004.01 1017.63 1018.59
1023.47 1056.41 1057.23 1106.03 1108.82 1135.07
1147.52 1172.03 1172.13 1198.78 1200.99 1293.18
1294.59 1299.33 1314.87 1320.20 1350.04 1368.35
1371.10 1373.07 1420.80 1424.54 1427.31 1427.92
1432.88 1438.33 1460.31 1463.52 1512.85 1515.25

1637.26 1639.24 1659.09 1660.31 2329.94 3018.27
3040.95 3043.65 3044.96 3130.36 3137.34 3139.05
3140.97 3144.36 3145.64 3148.42 3148.88 3163.08
3166.60 3187.51 3187.71 3197.27 3197.97 3200.33
3206.13 3206.29 3209.17

==============================
10A-TS
==============================
-68.24 26.86 31.87 37.30 43.73 51.21
58.55 72.77 82.68 93.53 115.08 140.82
149.90 156.61 161.61 169.20 190.30 196.23
219.37 224.58 249.96 275.23 366.08 390.59
401.29 441.14 446.04 461.84 562.67 607.42
615.72 616.90 618.21 660.75 670.86 690.33
696.89 697.44 723.01 726.59 740.60 751.65
755.20 786.97 820.11 832.02 840.73 858.32
860.96 866.58 920.82 924.64 947.84 956.93
988.22 995.20 1001.74 1003.85 1024.06 1025.26
1053.95 1055.01 1107.50 1113.86 1134.85 1147.63
1174.70 1175.14 1200.35 1203.83 1253.85 1284.72
1285.57 1291.49 1312.54 1322.70 1378.57 1380.89
1403.45 1416.54 1428.27 1430.27 1432.97 1441.21
1447.48 1451.80 1459.66 1464.30 1506.14 1508.02
1627.55 1630.04 1651.92 1655.16 2231.28 3005.11
3034.65 3039.71 3043.21 3090.83 3104.91 3126.59
3131.58 3139.21 3142.95 3143.95 3161.14 3166.90
3170.24 3170.74 3180.76 3191.48 3192.02 3203.71
3203.97 3209.84 3210.02

==============================
11A
==============================
8.00 19.72 36.15 45.95 57.70 67.50
72.92 110.20 119.24 134.55 138.95 140.86
143.96 163.49 167.43 181.14 194.91 202.71
215.13 233.32 245.85 320.81 367.37 395.02
402.08 453.69 456.69 466.10 611.68 619.53
620.43 642.39 671.73 673.20 683.57 701.58
706.16 719.22 732.39 747.70 749.80 755.79
758.37 776.04 812.09 839.51 856.21 863.76
869.37 877.20 896.28 940.01 947.70 984.51
990.74 991.91 1003.39 1004.80 1018.53 1020.88
1055.37 1055.76 1103.59 1107.48 1134.32 1147.52
1172.15 1172.46 1196.99 1199.48 1285.26 1286.06
1287.12 1293.35 1313.75 1320.96 1371.77 1373.76
1412.57 1415.26 1418.52 1421.87 1423.47 1427.02
1429.68 1437.10 1459.69 1463.96 1511.74 1512.84
1635.37 1637.89 1656.94 1658.45 1886.24 3039.41
3040.87 3042.54 3043.20 3133.78 3135.81 3136.75
3137.16 3140.20 3143.46 3148.84 3150.51 3153.32
3157.01 3162.93 3166.94 3187.82 3188.14 3197.79
3198.52 3206.20 3206.42

==============================
12A Me3Si+
==============================
29.95 35.06 66.20 198.48 199.16 211.54
606.76 632.77 635.35 699.57 757.78 759.49
835.87 902.00 902.54 1283.50 1283.75 1288.76
1391.78 1391.88 1393.79 1394.58 1400.83 1401.85
3028.54 3028.98 3032.11 3110.78 3111.84 3112.05
3166.21 3166.77 3167.63

==============================
12A Ph2MeSiH
==============================

17.45 34.08 51.56 94.28 124.50 144.25
170.18 209.17 236.21 285.58 386.32 398.09
401.06 422.28 460.23 481.06 623.92 624.59
656.25 691.09 702.49 707.13 709.73 729.14
735.50 751.35 773.95 847.88 864.17 868.88
896.65 924.86 930.26 973.56 978.28 998.18
1000.04 1004.18 1005.91 1055.00 1056.38 1098.31
1100.05 1132.84 1141.10 1162.65 1162.98 1195.57
1197.50 1278.88 1314.49 1315.94 1360.57 1363.44
1437.92 1439.56 1459.68 1461.28 1514.68 1516.46
1636.05 1640.55 1662.93 1664.51 2248.15 3038.62
3128.43 3133.18 3138.06 3141.39 3145.53 3151.30
3161.98 3168.92 3169.65 3178.88 3186.81 3191.92

==============================
13A Me3SiH
==============================

137.73 147.51 148.11 179.14 179.58 213.57
595.97 596.23 622.66 659.45 707.50 707.88
841.41 841.96 869.11 918.57 918.84 1275.97
1276.09 1283.60 1434.51 1436.73 1436.90 1448.04
1448.13 1454.70 2245.47 3035.41 3037.06 3037.22
3129.34 3129.91 3129.98 3138.36 3138.42 3138.85

==============================
13A Ph2MeSi+
==============================

32.95 44.26 74.10 78.91 92.89 147.75
148.72 223.30 249.92 277.34 366.57 389.61
399.51 435.47 441.37 464.21 614.51 615.60
672.15 686.28 692.93 727.28 739.77 752.39
754.48 787.30 824.59 857.41 864.30 949.99
958.93 990.15 996.43 1001.08 1004.55 1026.84
1027.92 1053.57 1054.23 1108.45 1115.27 1137.57
1149.62 1176.02 1176.26 1201.54 1204.92 1287.23
1311.48 1322.51 1382.21 1385.07 1408.69 1414.22
1460.24 1464.98 1503.72 1506.75 1624.18 1627.00
1650.47 1655.27 3044.09 3131.79 3168.68 3169.30
3173.16 3177.82 3184.24 3193.07 3193.50 3205.88
3206.07 3211.29 3211.55

==============================
1B
==============================

25.85 35.32 50.86 96.95 127.21 159.32
174.12 207.59 242.73 291.03 380.63 398.75
401.72 419.37 458.44 483.90 621.62 623.57
654.44 688.40 701.61 709.29 710.40 730.25
731.55 757.65 767.64 850.71 865.95 866.61
892.93 926.50 929.50 975.18 977.26 998.69
999.58 1002.32 1005.96 1050.16 1055.82 1098.73
1099.73 1130.80 1140.08 1162.91 1163.04 1195.30
1197.06 1276.14 1314.25 1317.48 1362.06 1363.88
1436.92 1442.45 1459.34 1461.71 1515.20 1516.21
1637.68 1640.33 1663.40 1664.38 2248.91 3037.52
3131.96 3134.72 3140.13 3141.85 3145.90 3148.00
3164.49 3166.56 3172.10 3175.70 3187.63 3188.85

==============================
2B
==============================

56.42 56.94 74.70 76.86 77.73 113.44
232.98 235.59 246.04 287.09 324.64 325.14
408.20 408.86 428.15 429.01 471.87 472.74
612.54 613.20 613.67 626.85 627.72 665.69
690.98 691.56 711.44 718.12 776.48 776.78

813.50 854.04 854.87 857.09 932.26 933.48
962.58 967.18 968.12 991.44 992.19 993.36
1004.36 1004.44 1005.19 1021.35 1021.61 1022.27
1054.33 1055.41 1055.46 1115.52 1119.60 1120.43
1173.75 1173.84 1174.81 1193.70 1194.20 1197.50
1224.79 1314.63 1321.14 1322.13 1380.68 1382.27
1395.03 1410.66 1411.23 1472.05 1482.51 1483.15
1514.45 1523.24 1523.52 1621.05 1624.18 1624.55
1656.07 1656.78 1670.97 3190.22 3190.41 3190.56
3194.87 3195.93 3196.49 3198.50 3198.57 3199.68
3208.43 3208.69 3209.00 3212.95 3213.11 3213.50

==============================
3B
==============================
3.89 10.45 24.87 31.27 35.06 53.89
59.21 64.59 69.36 72.41 77.53 83.86
85.42 92.79 102.18 121.46 129.87 154.60
177.28 212.01 235.82 236.81 241.04 247.54
275.01 288.84 324.34 326.32 388.78 397.89
404.49 408.22 410.68 425.27 427.75 430.34
460.63 470.94 471.86 474.20 613.24 613.84
614.89 622.76 624.52 627.64 627.90 658.58
663.81 685.83 687.72 691.26 701.24 705.79
708.17 714.98 718.52 730.87 735.40 763.36
770.92 772.86 774.39 810.13 845.46 851.92
854.28 856.30 865.31 872.13 900.90 925.83
932.26 932.84 936.74 956.13 961.94 968.52
975.35 982.21 984.88 989.28 995.37 999.77
1002.46 1004.74 1005.22 1005.64 1008.27 1009.33
1012.39 1015.45 1019.12 1053.78 1054.43 1054.70
1055.97 1056.50 1096.41 1099.66 1113.92 1117.43
1121.23 1130.41 1138.30 1164.42 1164.86 1171.09
1171.83 1172.60 1191.02 1192.28 1193.06 1195.13
1198.79 1223.16 1281.88 1309.27 1313.32 1314.59
1319.65 1322.66 1360.91 1363.17 1378.47 1380.82
1394.06 1409.04 1410.87 1432.38 1436.29 1457.06
1461.26 1471.07 1481.57 1482.90 1512.51 1513.04
1513.82 1521.43 1522.72 1620.52 1623.47 1626.57
1633.69 1635.99 1655.51 1656.93 1659.47 1661.32
1670.71 2202.18 3039.22 3123.25 3137.49 3139.27
3143.81 3144.16 3151.86 3170.46 3170.91 3177.89
3180.04 3185.48 3187.05 3188.31 3191.23 3193.43
3193.53 3194.65 3195.61 3197.41 3200.17 3200.81
3206.56 3207.98 3209.20 3210.61 3211.08 3213.08

==============================
3B-TS
==============================
-392.24 17.60 24.80 28.42 41.27 41.71
50.89 63.35 66.42 68.18 78.77 85.66
85.97 95.27 122.76 132.50 146.84 165.70
182.64 212.64 223.66 236.53 245.31 249.44
268.47 300.75 320.18 322.80 336.61 379.08
391.92 399.25 406.25 408.36 426.27 444.90
456.67 473.35 486.35 488.60 504.09 614.10
615.15 618.68 619.89 621.32 626.41 629.10
669.39 691.51 696.50 697.50 700.38 703.92
709.33 710.88 732.43 742.25 751.73 754.98
767.34 768.90 777.69 800.68 848.48 850.11
852.62 857.78 863.06 907.35 916.50 928.41
934.48 942.65 944.50 947.35 973.71 975.80
978.92 981.25 981.54 983.08 986.09 1003.00
1003.24 1007.32 1008.71 1008.91 1009.43 1010.73
1011.23 1011.87 1013.05 1054.63 1055.33 1056.46
1058.25 1058.91 1100.25 1104.57 1108.80 1114.59
1116.04 1126.26 1139.87 1145.87 1167.65 1168.04
1169.09 1169.31 1169.74 1189.88 1190.49 1194.71
1197.01 1198.21 1280.01 1301.30 1307.16 1310.85

1311.42 1317.02 1345.07 1347.25 1365.20 1369.07
1371.24 1391.91 1393.62 1424.00 1430.69 1444.97
1459.66 1462.71 1473.83 1482.59 1483.86 1511.06
1512.01 1519.35 1521.05 1545.38 1633.61 1635.50
1636.15 1639.08 1641.75 1658.20 1659.55 1662.68
1663.97 1683.28 3039.94 3135.53 3136.58 3144.55
3149.47 3159.97 3165.31 3167.90 3172.47 3180.95
3181.31 3182.27 3187.88 3189.19 3189.52 3191.03
3191.20 3196.24 3198.13 3199.06 3200.80 3201.69
3203.61 3205.29 3206.55 3209.43 3211.89 3214.11

==============================
4B
==============================
2.90 25.23 26.84 31.51 38.06 45.91
54.06 55.68 57.47 68.59 73.09 76.60
81.21 94.08 105.55 128.06 132.33 156.81
162.86 221.08 227.85 236.61 245.89 246.23
267.25 273.97 294.40 314.25 362.80 389.40
398.07 408.85 411.28 417.64 439.75 445.77
464.15 466.13 490.40 503.25 607.76 610.43
614.18 614.93 621.16 624.99 627.15 664.17
669.19 689.52 695.12 706.02 709.34 709.99
725.43 737.79 742.02 749.08 757.71 759.85
765.67 785.29 822.05 845.26 850.29 854.09
855.02 857.30 864.77 877.30 883.03 925.82
931.13 934.90 940.09 951.23 972.84 973.83
976.19 982.07 991.17 1000.08 1000.56 1002.51
1003.01 1004.77 1005.16 1009.38 1010.58 1017.28
1022.84 1053.40 1054.09 1057.38 1059.91 1060.44
1102.56 1105.02 1107.09 1108.77 1111.81 1132.91
1146.97 1149.39 1159.24 1164.69 1165.18 1166.74
1173.53 1173.96 1186.88 1188.85 1191.30 1196.90
1199.15 1201.84 1264.38 1277.15 1285.37 1313.33
1320.89 1321.81 1328.87 1333.13 1374.57 1377.97
1380.07 1380.68 1382.28 1408.27 1414.74 1460.01
1464.01 1476.25 1482.17 1484.19 1506.64 1507.30
1526.00 1527.05 1527.87 1628.87 1630.91 1650.49
1652.66 1654.77 1655.37 1657.31 1672.38 1673.54
1677.64 2589.29 3010.28 3112.58 3137.95 3142.90
3158.90 3159.28 3162.93 3164.15 3165.55 3172.08
3172.53 3177.84 3180.99 3181.99 3184.73 3187.35
3188.73 3189.54 3189.81 3192.49 3194.98 3200.88
3201.13 3201.68 3202.09 3204.06 3207.57 3208.28

==============================
5B
==============================
21.99 25.45 50.30 59.91 60.26 78.62
218.42 234.25 234.80 270.70 299.69 300.47
408.26 408.68 413.27 474.09 499.78 501.06
609.21 609.98 620.77 623.03 626.54 663.84
707.68 708.30 710.10 740.52 763.22 763.91
833.34 852.02 852.55 854.73 879.18 880.30
924.46 925.21 926.68 967.04 967.63 968.34
990.92 991.39 991.84 1008.76 1009.34 1009.57
1058.44 1060.09 1060.38 1102.72 1102.93 1105.84
1160.89 1161.21 1161.35 1187.13 1187.53 1188.89
1199.06 1212.85 1213.66 1289.56 1290.06 1322.70
1341.99 1342.02 1372.26 1383.33 1383.60 1479.77
1486.18 1486.29 1530.04 1530.79 1530.80 1655.40
1659.93 1660.18 1677.86 1677.89 1683.32 3002.76
3153.70 3153.82 3154.10 3167.67 3167.76 3168.50
3175.71 3175.99 3177.18 3183.30 3183.56 3184.58
3194.20 3194.35 3194.83

==============================
6B
==============================

```
    32.95    44.26    74.10    78.91    92.89   147.75
   148.72   223.30   249.92   277.34   366.57   389.61
   399.51   435.47   441.37   464.21   614.51   615.60
   672.15   686.28   692.93   727.28   739.77   752.39
   754.48   787.30   824.59   857.41   864.30   949.99
   958.93   990.15   996.43  1001.08  1004.55  1026.84
  1027.92  1053.57  1054.23  1108.45  1115.27  1137.57
  1149.62  1176.02  1176.26  1201.54  1204.92  1287.23
  1311.48  1322.51  1382.21  1385.07  1408.69  1414.22
  1460.24  1464.98  1503.72  1506.75  1624.18  1627.00
  1650.47  1655.27  3044.09  3131.79  3168.68  3169.30
  3173.16  3177.82  3184.24  3193.07  3193.50  3205.88
  3206.07  3211.29  3211.55
```

==============================
7B
==============================

```
     2.51    19.37    31.80    35.50    40.88    44.29
    52.95    57.18    67.57    76.23    80.51   108.25
   127.68   139.40   143.66   150.99   171.01   177.67
   180.87   190.16   215.70   221.24   242.46   243.22
   282.69   331.87   359.20   375.37   395.70   396.00
   400.85   406.47   445.20   450.82   458.67   459.68
   469.10   478.13   619.79   619.98   620.55   620.66
   670.46   671.21   691.00   702.94   704.94   705.97
   710.68   718.31   719.34   732.03   748.93   750.66
   751.11   757.32   766.85   770.74   810.16   818.03
   858.85   863.49   865.79   866.41   870.85   938.14
   939.37   941.17   946.56   962.54   981.85   984.44
   985.02   989.62  1002.95  1003.05  1003.66  1003.82
  1013.93  1016.54  1017.28  1017.83  1054.20  1054.86
  1055.17  1055.76  1102.90  1103.29  1105.54  1107.23
  1130.79  1135.01  1146.01  1147.46  1169.48  1170.70
  1171.20  1171.21  1196.68  1197.34  1198.85  1200.26
  1283.11  1285.81  1314.13  1315.34  1320.39  1321.88
  1368.29  1368.58  1369.95  1370.87  1422.01  1424.32
  1426.05  1426.57  1458.03  1458.60  1461.82  1462.79
  1511.00  1511.46  1512.73  1512.94  1633.96  1634.47
  1636.25  1637.31  1655.56  1656.25  1657.50  1657.83
  1860.65  3032.07  3039.41  3126.55  3131.33  3134.73
  3136.30  3138.72  3144.59  3148.27  3155.32  3158.24
  3160.42  3163.80  3165.31  3179.12  3179.93  3182.68
  3183.47  3191.47  3192.23  3192.60  3193.11  3198.66
  3200.04  3201.38  3202.20
```

==============================
7B-TS
==============================

```
   -94.45     2.99    18.51    34.77    38.12    47.76
    54.01    59.65    63.15    76.61    78.90    91.90
    98.29   110.92   130.37   140.18   158.74   171.59
   175.44   193.36   208.92   225.22   244.24   245.41
   273.23   294.09   360.80   373.00   391.46   397.20
   399.97   400.93   428.63   441.09   452.88   456.80
   462.21   473.54   614.99   617.62   620.78   623.01
   658.48   670.13   676.50   689.76   700.29   705.48
   707.41   710.94   723.99   736.84   739.55   748.82
   749.35   756.53   764.66   779.14   782.21   824.42
   855.78   863.92   864.69   867.50   870.12   903.13
   932.74   934.37   943.75   949.83   977.41   978.99
   985.29   990.82  1001.32  1001.57  1003.59  1004.90
  1008.44  1009.61  1019.64  1021.18  1053.80  1054.39
  1054.65  1055.90  1099.68  1102.29  1105.44  1112.40
  1130.04  1132.46  1141.97  1144.74  1167.31  1167.67
  1173.17  1173.38  1194.31  1196.85  1199.20  1203.35
  1281.08  1283.62  1312.08  1313.06  1317.80  1322.64
```

1366.57 1367.90 1373.21 1378.55 1411.58 1419.27
1429.38 1434.34 1457.99 1458.48 1462.35 1463.43
1506.57 1508.77 1511.21 1515.16 1628.23 1631.20
1633.47 1639.72 1652.96 1654.96 1657.86 1663.21
2132.75 3038.99 3040.34 3129.86 3133.20 3133.36
3140.98 3145.53 3147.17 3148.31 3159.44 3160.22
3163.38 3167.43 3174.16 3177.27 3182.60 3183.28
3185.66 3187.97 3189.29 3193.76 3197.20 3197.70
3200.05 3205.65 3206.11

==============================
8B
==============================
8.09 25.27 29.51 39.86 49.94 58.99
61.89 81.96 85.79 93.51 107.83 129.15
143.41 162.24 168.44 173.34 182.39 191.19
193.35 213.06 228.49 233.34 245.70 256.15
290.84 330.93 361.68 377.83 394.49 399.48
402.83 406.30 421.25 443.47 466.01 473.48
477.00 486.49 607.70 619.57 620.32 621.58
639.62 673.07 676.62 701.28 702.34 704.15
707.16 712.75 719.34 738.00 738.77 745.18
751.97 754.07 756.65 780.83 791.67 812.31
839.96 858.86 862.55 868.53 879.69 893.33
931.97 934.75 941.84 976.26 978.88 987.59
997.41 999.13 1001.56 1003.58 1004.02 1009.78
1010.33 1012.87 1015.87 1018.67 1038.84 1052.30
1054.88 1055.87 1057.79 1101.11 1104.47 1105.26
1109.43 1127.68 1137.13 1140.78 1169.05 1169.74
1169.85 1177.21 1195.87 1198.06 1200.17 1203.36
1287.10 1288.25 1295.64 1312.21 1318.55 1319.65
1363.66 1367.74 1369.56 1378.25 1428.72 1430.95
1434.16 1435.41 1458.41 1460.53 1461.85 1462.28
1481.97 1512.85 1513.68 1514.88 1607.19 1632.18
1636.65 1638.97 1641.11 1658.41 1659.93 1660.97
2306.74 3037.25 3039.15 3129.04 3130.53 3136.12
3137.12 3146.29 3149.49 3152.71 3153.45 3161.13
3162.52 3163.93 3169.56 3174.87 3179.86 3180.74
3182.39 3183.24 3188.95 3192.45 3197.26 3199.29
3201.49 3201.67 3206.55

==============================
8B-TS
==============================
-114.91 16.14 27.55 35.76 41.83 44.22
49.48 55.55 63.70 68.92 102.89 121.09
136.88 141.37 143.19 155.68 165.40 177.96
183.60 212.86 215.47 221.38 236.20 244.26
252.64 276.87 356.85 382.82 395.94 397.23
399.57 403.75 413.20 429.22 453.58 463.00
483.84 494.21 615.19 618.85 622.53 622.66
638.81 659.11 678.71 687.11 697.77 705.32
706.82 710.61 714.20 732.05 742.96 747.86
750.66 751.72 755.52 762.06 792.49 802.87
812.78 863.45 864.21 874.48 886.67 893.14
930.40 941.20 943.83 961.76 976.68 983.14
986.99 993.50 1002.38 1003.61 1004.05 1006.58
1009.80 1012.78 1017.99 1020.92 1048.44 1053.99
1054.09 1054.69 1086.15 1099.75 1104.51 1106.14
1111.95 1130.92 1137.39 1141.23 1167.51 1168.60
1171.24 1171.63 1195.04 1196.48 1197.48 1200.53
1281.08 1285.72 1302.29 1313.85 1316.25 1318.30
1361.07 1362.88 1372.68 1378.52 1414.39 1420.38
1431.59 1439.53 1445.80 1458.15 1459.82 1461.64
1496.71 1510.93 1511.85 1513.69 1609.80 1632.89
1633.53 1634.46 1643.48 1655.75 1656.89 1658.53
2356.53 3035.21 3035.92 3129.26 3129.51 3133.11
3133.43 3137.17 3143.12 3148.38 3149.18 3150.82
3152.25 3156.91 3168.32 3174.69 3180.19 3181.57

3187.24 3188.12 3190.31 3193.45 3196.98 3198.17
3200.97 3205.81 3206.97

==============================
9B
==============================
2.95 14.07 25.79 36.09 39.59 49.60
60.49 66.69 74.25 83.84 92.19 125.52
141.41 153.25 157.36 170.25 175.44 180.73
187.93 216.00 229.47 234.03 245.18 254.70
292.45 317.62 362.44 395.63 397.71 399.17
403.28 407.00 435.02 449.41 460.99 466.90
493.43 498.42 615.70 619.68 622.73 623.50
656.32 664.16 680.94 694.74 703.77 706.85
707.77 712.58 717.78 740.41 745.45 750.71
752.09 752.62 758.21 771.60 792.94 802.90
830.97 864.43 865.30 873.99 890.01 908.15
933.56 938.83 940.47 949.66 979.87 984.64
987.70 992.61 1002.68 1003.29 1004.07 1011.21
1012.87 1017.90 1020.57 1032.38 1050.47 1054.31
1056.59 1057.49 1075.13 1099.57 1101.32 1105.39
1105.96 1132.06 1139.15 1141.32 1168.33 1168.81
1171.03 1171.89 1189.85 1197.49 1198.13 1201.51
1282.19 1289.94 1299.24 1313.27 1319.24 1319.42
1364.09 1366.08 1367.74 1389.19 1424.15 1428.18
1434.07 1436.87 1439.04 1457.60 1461.16 1461.39
1490.41 1512.81 1514.63 1515.71 1594.20 1635.29
1635.95 1637.78 1639.22 1654.77 1659.59 1660.12
2344.60 3038.00 3041.99 3060.74 3125.10 3130.14
3133.18 3135.53 3139.96 3144.66 3146.36 3150.11
3158.06 3163.48 3164.99 3178.73 3181.56 3181.65
3182.94 3187.65 3189.60 3189.87 3191.11 3198.59
3200.21 3201.30 3209.22

==============================
9B-TS
==============================
-74.47 4.90 19.55 27.06 32.22 38.85
41.11 48.55 54.57 68.13 82.44 86.97
103.73 120.94 134.52 136.29 144.09 154.91
170.11 208.24 216.74 220.64 232.80 245.13
248.53 284.96 344.75 387.84 396.19 397.56
400.58 402.27 431.86 434.83 445.62 477.87
484.39 489.16 554.12 612.00 620.50 623.08
624.01 660.54 677.42 685.50 691.92 706.04
707.47 708.10 710.55 713.84 735.89 736.83
746.15 750.16 750.70 765.07 768.45 792.41
823.60 850.69 862.28 864.65 870.92 898.45
928.52 932.12 936.82 944.70 976.89 978.03
981.87 983.76 1000.49 1000.90 1004.12 1004.48
1008.00 1008.56 1008.88 1022.87 1049.49 1052.88
1055.92 1056.61 1097.73 1100.63 1101.76 1110.74
1125.28 1135.03 1140.59 1146.08 1164.50 1166.62
1167.01 1174.58 1194.67 1195.39 1197.16 1200.28
1219.80 1288.44 1310.15 1314.22 1316.20 1317.57
1363.17 1364.12 1364.65 1383.57 1404.71 1413.05
1435.05 1443.66 1455.69 1459.58 1459.68 1460.36
1501.64 1508.20 1513.67 1514.87 1619.24 1626.79
1637.05 1638.29 1648.40 1654.03 1660.63 1661.11
2375.50 2983.99 3040.26 3080.32 3115.12 3122.69
3133.64 3134.00 3140.24 3157.89 3160.21 3163.23
3170.57 3171.94 3172.24 3172.59 3178.61 3179.21
3183.09 3188.21 3188.59 3188.76 3192.59 3199.25
3199.77 3201.44 3207.25

==============================
10B
==============================

9.34	22.62	28.53	36.23	38.25	41.10
44.39	51.93	55.73	61.70	76.56	108.94
117.61	136.55	139.34	166.17	171.72	183.03
202.76	215.66	234.51	243.98	249.00	250.92
287.33	307.24	322.62	393.45	395.58	396.25
400.37	402.52	442.79	446.31	455.44	466.15
503.28	504.85	543.29	619.00	620.14	620.81
621.78	661.78	681.35	692.16	701.63	704.18
705.36	707.40	721.77	730.91	734.24	741.84
747.07	749.82	751.21	753.23	780.19	833.34
860.64	861.65	863.23	868.28	889.09	929.71
936.42	939.44	944.32	973.00	977.76	982.84
984.35	987.68	1001.94	1002.90	1003.23	1004.17
1010.85	1013.07	1017.22	1017.80	1047.44	1054.50
1054.92	1056.52	1058.03	1103.56	1104.48	1107.38
1107.85	1126.03	1140.69	1144.39	1146.05	1168.95
1170.24	1171.47	1171.58	1196.89	1198.71	1198.81
1201.44	1294.97	1312.99	1317.31	1318.41	1319.28
1332.20	1368.14	1370.34	1371.47	1372.04	1374.01
1425.33	1431.51	1458.41	1461.53	1462.65	1463.41
1512.21	1513.62	1513.92	1514.81	1636.11	1638.20
1638.57	1639.49	1659.35	1659.58	1660.05	1661.16
2325.94	3015.98	3042.32	3117.96	3122.79	3135.90
3137.16	3144.30	3149.49	3157.60	3165.89	3167.18
3169.52	3180.02	3183.60	3186.05	3186.40	3190.68
3194.04	3195.68	3196.18	3196.63	3199.69	3202.81
3204.95	3205.19	3218.64			

==============================
10B-TS
==============================

-52.52	4.78	7.20	14.29	26.85	32.47
40.05	48.04	53.50	62.97	65.58	78.12
96.52	97.26	138.61	150.69	159.22	159.64
167.08	179.51	185.66	234.08	243.70	251.13
253.44	287.65	307.74	383.19	394.73	397.26
397.98	407.76	430.55	432.38	462.25	464.28
498.15	505.69	581.92	615.63	617.46	618.24
623.89	641.33	682.55	688.15	691.93	697.75
700.10	705.62	711.09	721.36	744.12	744.91
745.77	749.76	753.71	758.53	774.77	843.29
862.68	863.90	864.25	871.24	886.16	900.45
927.89	948.39	957.94	960.66	974.75	990.69
992.27	996.52	1001.42	1001.69	1004.10	1005.55
1006.91	1022.80	1023.56	1024.19	1053.60	1054.29
1055.30	1056.84	1100.76	1103.62	1110.97	1111.52
1123.26	1142.95	1144.67	1146.39	1166.88	1173.90
1174.09	1174.23	1195.79	1199.98	1201.09	1205.29
1260.74	1282.53	1309.50	1316.19	1319.71	1320.78
1365.10	1375.77	1377.91	1379.41	1425.99	1434.23
1437.65	1443.34	1457.24	1461.56	1462.66	1463.59
1504.83	1506.20	1507.67	1516.48	1625.65	1628.50
1630.10	1640.65	1651.85	1653.27	1656.06	1663.81
2257.25	3013.60	3032.62	3105.55	3109.15	3123.41
3133.08	3135.92	3144.65	3167.71	3169.47	3173.82
3174.63	3175.24	3177.20	3182.34	3183.91	3190.07
3190.58	3190.75	3195.58	3201.29	3202.08	3202.37
3207.66	3208.66	3208.86			

==============================
11B
==============================

8.00	19.72	36.15	45.95	57.70	67.50
72.92	110.20	119.24	134.55	138.95	140.86
143.96	163.49	167.43	181.14	194.91	202.71
215.13	233.32	245.85	320.81	367.37	395.02

402.08	453.69	456.69	466.10	611.68	619.53
620.43	642.39	671.73	673.20	683.57	701.58
706.16	719.22	732.39	747.70	749.80	755.79
758.37	776.04	812.09	839.51	856.21	863.76
869.37	877.20	896.28	940.01	947.70	984.51
990.74	991.91	1003.39	1004.80	1018.53	1020.88
1055.37	1055.76	1103.59	1107.48	1134.32	1147.52
1172.15	1172.46	1196.99	1199.48	1285.26	1286.06
1287.12	1293.35	1313.75	1320.96	1371.77	1373.76
1412.57	1415.26	1418.52	1421.87	1423.47	1427.02
1429.68	1437.10	1459.69	1463.96	1511.74	1512.84
1635.37	1637.89	1656.94	1658.45	1886.24	3039.41
3040.87	3042.54	3043.20	3133.78	3135.81	3136.75
3137.16	3140.20	3143.46	3148.84	3150.51	3153.32
3157.01	3162.93	3166.94	3187.82	3188.14	3197.79
3198.52	3206.20	3206.42			

===============================

12B Me2PhSi+

===============================

81.77	109.88	132.62	229.86	279.93	306.08
348.19	388.18	391.50	428.38	515.00	612.19
648.87	679.04	751.83	753.60	768.89	784.71
853.41	862.27	884.12	944.80	954.94	991.08
1001.92	1029.87	1051.92	1115.00	1150.42	1177.72
1203.59	1310.00	1324.00	1352.78	1390.20	1420.22
1425.67	1445.34	1445.98	1463.65	1503.85	1621.80
1651.79	2720.91	2829.99	2885.01	2904.70	2995.17
3046.45	3172.12	3176.98	3193.31	3208.10	3212.54

===============================

12B Ph3SiH

===============================

13.09	31.67	35.61	45.99	57.49	58.06
151.74	158.40	176.32	219.34	235.12	251.43
394.13	395.99	400.63	402.21	421.11	430.71
478.61	491.98	501.22	623.62	624.15	625.18
686.88	702.58	706.78	709.69	711.37	711.66
741.54	743.06	754.38	813.81	829.89	863.37
867.11	867.96	923.99	928.92	933.46	973.23
976.69	979.93	998.14	998.92	1000.01	1004.87
1005.00	1005.54	1055.45	1055.80	1056.76	1097.41
1099.81	1101.06	1131.27	1138.58	1140.56	1163.11
1163.25	1163.34	1194.43	1195.70	1198.14	1312.56
1317.11	1317.67	1361.50	1364.29	1364.77	1458.91
1461.90	1462.90	1514.59	1515.12	1516.39	1637.99
1640.22	1640.40	1663.61	1664.02	1664.55	2249.71
3136.36	3140.80	3146.44	3146.89	3149.27	3151.30
3166.55	3166.93	3168.09	3174.57	3175.23	3177.26
3189.05	3189.34	3189.86			

===============================

13B Ph3Si+

===============================

23.91	30.26	43.33	51.72	59.30	96.29
142.55	147.18	161.86	236.90	243.79	251.46
327.51	392.48	392.94	402.06	428.24	435.88
463.48	497.51	506.72	613.85	616.38	617.81
681.14	686.89	693.41	704.51	741.66	741.94
752.39	754.81	755.24	858.75	863.12	869.83
943.83	955.50	962.76	989.31	992.31	996.89
1000.56	1001.93	1005.58	1021.66	1025.53	1026.63
1052.93	1055.29	1055.95	1101.94	1109.96	1118.75
1122.88	1142.14	1145.64	1174.39	1174.97	1175.78
1201.25	1201.42	1209.94	1306.66	1319.42	1323.17
1369.06	1380.73	1384.41	1455.73	1461.72	1464.78
1502.39	1506.99	1509.75	1623.18	1627.19	1632.57
1650.60	1654.14	1656.47	3158.71	3159.82	3179.64
3180.90	3187.98	3191.69	3191.83	3194.12	3199.93

3201.85 3205.70 3207.29 3209.41 3211.41 3212.87

==============================
13B PhMe2SiH
==============================

29.05 83.74 131.57 145.02 152.73 177.02
230.75 287.20 380.94 398.96 466.82 602.96
624.44 648.02 693.84 705.08 710.71 728.17
737.73 778.27 852.55 864.06 899.77 901.39
924.73 973.17 997.55 1006.30 1056.48 1098.69
1139.87 1162.53 1195.40 1275.82 1280.78 1316.18
1364.74 1434.03 1436.75 1443.45 1447.96 1461.09
1517.15 1641.94 1665.60 2253.18 3036.55 3037.69
3128.31 3129.49 3134.41 3143.43 3144.23 3151.49
3168.38 3178.74 3190.75

==============================
14B
==============================

10.62 20.24 36.76 43.20 46.19 55.85
59.23 75.67 82.04 113.56 132.73 137.12
146.21 149.69 158.90 167.19 174.51 178.57
190.71 217.84 240.56 262.32 285.55 337.83
361.69 384.39 392.82 395.54 398.51 450.34
454.17 459.66 467.53 619.26 619.80 619.94
640.35 643.76 672.64 698.88 699.59 701.71
716.21 721.51 722.34 732.13 740.82 744.39
753.50 764.00 772.24 799.73 814.99 837.17
858.48 861.14 866.99 873.47 922.05 934.55
937.04 943.41 977.56 979.04 986.59 1003.44
1003.75 1004.14 1012.66 1013.68 1017.87 1054.48
1054.58 1056.25 1104.35 1105.29 1106.67 1136.76
1142.48 1152.37 1170.23 1170.82 1171.73 1196.25
1197.09 1198.33 1284.93 1288.24 1294.16 1315.74
1317.54 1320.54 1370.16 1373.62 1375.82 1414.72
1420.74 1421.49 1425.74 1427.79 1431.73 1460.84
1461.52 1464.40 1512.60 1513.07 1514.38 1636.80
1637.87 1639.69 1657.75 1659.89 1660.60 1916.66
3029.95 3036.79 3038.17 3118.89 3127.42 3130.58
3135.51 3137.75 3140.58 3140.89 3144.91 3153.44
3160.97 3162.04 3163.59 3182.08 3185.86 3186.85
3194.36 3196.31 3197.30 3201.73 3204.75 3205.64

==============================
14B-TS
==============================

-108.64 23.01 29.17 41.33 43.36 54.08
82.17 90.09 94.33 108.85 119.24 128.35
153.06 159.85 176.36 180.64 188.06 204.18
207.75 238.10 242.38 250.47 270.44 280.02
359.59 367.59 389.79 395.03 403.11 440.70
443.50 464.77 472.11 609.29 617.18 617.62
620.08 647.85 669.04 677.79 685.56 697.76
704.87 706.74 717.16 735.90 740.83 749.90
751.55 758.32 780.54 791.75 820.62 855.44
858.33 867.38 883.93 898.86 903.37 938.40
942.72 949.60 982.72 989.60 991.43 1001.54
1003.85 1007.08 1017.79 1019.64 1021.36 1049.64
1053.60 1056.11 1089.26 1104.93 1111.98 1113.31
1127.77 1139.25 1171.14 1171.67 1172.47 1196.55
1198.72 1203.88 1280.53 1283.97 1291.79 1307.61
1312.76 1322.49 1370.14 1371.81 1373.91 1416.34
1427.48 1430.42 1432.45 1438.67 1443.18 1446.47
1458.88 1463.37 1500.79 1510.64 1512.34 1613.06
1633.79 1636.56 1645.06 1656.00 1657.30 2266.83
3038.54 3041.43 3045.59 3136.34 3138.09 3138.75
3143.53 3144.37 3153.28 3164.60 3167.41 3169.41
3170.42 3171.66 3182.09 3186.99 3189.07 3189.47
3191.75 3198.52 3199.31 3206.22 3206.68 3207.50

==============================
15B
==============================

18.35	22.54	39.01	49.54	54.28	66.35
74.02	87.63	120.63	138.16	153.14	158.97
166.77	179.58	180.27	198.92	208.71	224.16
232.93	238.10	245.70	263.19	269.58	328.30
353.28	383.97	396.39	398.07	400.10	438.13
474.78	481.21	492.40	606.90	621.90	622.55
623.70	636.56	669.43	678.11	699.41	704.19
706.71	709.56	714.40	723.48	738.52	747.26
751.26	775.64	785.13	790.13	810.98	859.08
860.94	864.16	875.85	878.71	912.04	933.20
939.12	978.85	981.93	995.56	999.22	1003.36
1003.98	1010.31	1012.00	1013.54	1013.71	1037.72
1054.05	1057.80	1058.68	1102.70	1104.18	1107.73
1131.55	1138.15	1169.85	1170.08	1176.04	1198.28
1198.96	1201.64	1285.22	1287.44	1293.59	1299.84
1314.42	1319.58	1367.52	1369.07	1377.26	1421.72
1425.23	1431.66	1436.09	1438.82	1445.36	1457.65
1459.76	1462.36	1481.72	1514.08	1516.00	1604.97
1637.61	1639.58	1641.05	1659.60	1661.41	2322.25
3040.77	3043.34	3045.67	3128.58	3133.29	3136.16
3149.37	3149.60	3152.05	3154.26	3155.79	3160.29
3161.82	3166.72	3167.62	3183.53	3184.09	3189.48
3192.69	3193.69	3202.99	3203.23	3205.08	3209.90

==============================
15B-TS
==============================

-164.07	26.38	32.29	45.90	49.05	60.86
65.29	76.10	115.96	129.74	139.99	141.46
181.84	186.41	199.04	200.03	211.52	215.22
217.29	227.50	232.82	245.39	248.90	289.84
346.53	390.95	396.39	403.17	412.04	427.86
465.21	476.87	497.30	613.06	622.17	622.77
628.70	652.87	661.81	674.35	679.34	698.72
703.10	710.07	713.17	717.03	737.65	746.20
750.81	758.21	785.31	787.83	803.30	861.37
864.07	870.66	878.21	886.70	906.74	933.57
939.55	969.90	980.44	985.21	997.18	1003.06
1003.49	1005.42	1011.19	1014.43	1029.70	1044.76
1056.35	1056.61	1082.92	1098.85	1101.45	1104.19
1132.22	1140.22	1168.49	1169.25	1175.06	1195.41
1197.46	1199.90	1280.94	1284.19	1287.93	1300.83
1313.25	1318.70	1364.89	1366.91	1382.47	1413.67
1417.24	1426.80	1433.34	1434.65	1435.81	1443.98
1458.31	1462.25	1490.41	1514.11	1514.77	1607.81
1636.79	1638.62	1647.51	1660.23	1660.87	2361.42
3036.27	3037.52	3039.49	3122.32	3131.54	3132.26
3132.29	3133.98	3137.97	3148.16	3154.13	3157.65
3159.01	3161.35	3174.14	3182.56	3183.36	3190.30
3191.54	3192.17	3198.30	3202.51	3202.67	3207.99

==============================
16B
==============================

10.27	14.93	37.52	43.14	52.52	70.49
78.40	92.96	119.08	125.08	147.93	161.25
163.74	167.29	181.67	188.03	204.91	216.84
229.16	232.25	238.76	246.55	254.90	324.69
364.25	395.97	401.58	404.49	437.52	447.30
467.34	494.16	500.74	613.59	616.58	622.50
623.32	658.06	664.69	681.45	694.15	704.74
706.79	708.45	716.92	720.26	742.14	749.97
751.39	775.07	780.76	793.15	800.85	860.03
863.12	867.33	875.74	900.04	912.49	933.10
938.34	952.98	978.83	987.91	994.73	1003.53
1003.91	1011.33	1013.17	1020.69	1033.78	1049.59

1056.83 1058.29 1078.22 1099.28 1101.21 1106.78
1132.67 1141.84 1169.07 1169.34 1175.39 1191.76
1197.50 1201.13 1280.71 1290.04 1295.15 1303.41
1313.97 1319.38 1365.95 1368.65 1385.75 1406.39
1415.33 1430.08 1432.46 1433.95 1435.83 1437.99
1459.35 1462.29 1491.31 1514.94 1516.08 1593.58
1637.37 1638.08 1639.95 1661.00 1661.78 2334.84
3041.03 3041.52 3042.35 3081.17 3128.02 3130.82
3134.12 3135.36 3141.36 3151.53 3153.60 3159.73
3159.84 3168.21 3180.25 3183.40 3183.67 3189.39
3192.22 3192.38 3192.97 3202.96 3203.36 3213.94

==============================

16B-TS

==============================

-100.67 8.89 12.59 31.81 40.80 41.50
47.47 62.75 68.44 72.84 75.28 95.44
109.30 140.58 148.69 162.73 167.65 190.57
203.21 216.05 223.60 237.78 240.67 248.79
358.85 395.19 398.78 401.46 422.65 445.66
469.38 485.06 497.84 512.51 621.69 622.42
622.67 628.56 654.82 662.71 671.93 678.73
706.84 707.43 710.42 711.92 716.05 736.38
747.38 756.77 766.77 790.72 815.92 831.80
864.45 869.79 871.78 877.79 921.88 931.17
932.55 946.95 978.06 984.30 990.12 1001.27
1002.62 1004.58 1010.10 1013.86 1015.97 1051.58
1053.29 1056.89 1098.23 1102.86 1106.18 1124.99
1135.76 1143.94 1167.87 1168.60 1168.76 1194.11
1195.81 1198.76 1204.18 1278.37 1289.83 1310.79
1312.78 1317.73 1356.25 1365.74 1367.63 1382.87
1386.88 1403.65 1411.95 1418.79 1438.91 1456.42
1460.22 1461.99 1508.25 1511.72 1516.11 1624.70
1628.84 1639.83 1654.00 1658.12 1663.30 2383.81
2942.53 3017.36 3037.24 3063.90 3086.36 3120.50
3125.86 3130.69 3133.18 3138.41 3146.59 3147.45
3147.73 3156.27 3159.10 3169.87 3171.63 3180.07
3182.66 3188.35 3193.27 3193.91 3199.25 3199.86

==============================

17B

==============================

12.88 19.38 23.06 30.71 31.94 41.27
49.74 53.00 61.04 102.19 106.10 129.97
143.33 154.10 167.56 183.89 191.58 201.47
218.24 235.81 246.77 250.49 266.94 272.13
308.92 394.40 394.56 401.36 443.46 445.17
458.72 504.02 505.51 561.32 615.92 619.98
620.99 621.10 658.89 683.14 688.19 701.71
704.35 704.90 707.08 733.51 735.53 748.12
750.71 751.84 765.10 804.68 858.83 861.67
863.02 867.43 885.55 900.36 937.58 939.73
944.21 982.23 983.61 984.29 987.46 1002.63
1003.04 1003.26 1017.20 1017.68 1017.97 1018.68
1055.22 1056.59 1056.85 1103.86 1107.26 1107.93
1126.56 1145.85 1146.44 1171.74 1171.81 1171.96
1198.62 1198.68 1201.21 1293.61 1298.32 1313.03
1318.37 1319.41 1359.07 1370.51 1372.39 1372.84
1380.49 1421.97 1429.00 1433.74 1439.42 1458.81
1462.98 1463.54 1511.89 1513.29 1513.77 1636.03
1638.16 1638.34 1658.99 1659.23 1659.52 2326.42
3017.45 3040.84 3042.19 3132.14 3135.55 3136.49
3136.67 3146.99 3147.09 3148.45 3165.49 3167.00
3169.76 3186.52 3187.10 3187.22 3193.59 3196.80
3197.00 3197.05 3205.11 3205.77 3205.94 3209.55

==============================
17B-TS
==============================

```
    -44.04     5.44    23.83    37.86    39.77    42.38
     51.67    55.66    60.52    72.73    79.93    95.85
    156.92   159.33   160.62   192.03   194.99   201.45
    209.41   225.30   234.87   242.54   252.06   252.96
    309.12   394.81   395.50   406.57   429.24   430.91
    462.76   502.04   503.62   591.33   614.14   615.60
    617.25   617.49   619.98   670.40   683.87   691.15
    697.46   697.90   698.38   716.20   746.42   746.84
    748.93   755.34   756.60   844.38   848.17   861.41
    862.62   870.13   871.88   923.83   927.29   946.89
    955.23   959.54   990.50   990.70   995.96  1001.77
   1002.01  1003.51  1021.99  1023.28  1023.73  1054.31
   1054.73  1055.49  1103.88  1109.96  1112.31  1125.83
   1148.36  1148.65  1173.66  1173.90  1174.25  1199.48
   1200.46  1204.48  1274.25  1287.18  1295.39  1310.74
   1319.70  1321.74  1376.90  1379.36  1383.39  1429.06
   1433.10  1435.48  1446.36  1449.42  1451.17  1457.93
   1463.13  1464.29  1505.90  1506.24  1506.86  1626.62
   1629.17  1630.46  1652.67  1654.16  1657.57  2209.24
   3005.45  3006.71  3018.26  3090.18  3093.06  3104.64
   3108.45  3121.27  3131.73  3161.01  3165.35  3167.34
   3173.18  3173.56  3178.06  3190.39  3193.80  3194.35
   3201.94  3203.17  3206.66  3209.87  3212.11  3216.35
```

==============================
18B
==============================

```
     11.30    24.13    29.86    36.00    38.43    47.48
     55.26    64.01    78.77   116.43   123.66   136.06
    146.48   153.56   159.63   175.26   180.42   183.90
    197.34   208.00   224.58   237.80   245.77   255.20
    362.40   392.75   397.10   405.40   433.21   443.48
    479.91   499.29   503.94   612.75   619.48   621.05
    621.67   655.64   679.36   682.61   686.38   702.02
    703.59   708.10   733.56   734.58   747.62   749.11
    752.68   753.88   757.25   841.96   855.70   860.13
    863.74   874.78   877.58   897.10   934.06   939.65
    955.91   980.49   984.94   996.15  1001.89  1003.03
   1003.56  1014.47  1015.44  1017.57  1021.13  1055.64
   1055.92  1057.45  1101.60  1106.76  1108.34  1125.57
   1145.03  1146.75  1171.74  1171.86  1172.11  1196.93
   1198.88  1201.72  1285.55  1286.44  1292.39  1311.81
   1317.76  1321.08  1369.12  1372.23  1374.06  1412.99
   1416.21  1418.79  1426.89  1429.71  1437.19  1458.03
   1461.18  1464.37  1510.43  1512.55  1513.92  1634.15
   1637.99  1638.15  1656.73  1658.60  1659.88  1890.28
   3036.94  3040.45  3041.15  3133.93  3135.49  3136.74
   3136.96  3142.02  3144.96  3148.26  3149.65  3152.78
   3160.63  3166.02  3169.39  3187.01  3187.11  3187.26
   3196.70  3197.16  3197.37  3205.35  3205.65  3205.96
```

==============================
19B Me3Si+
==============================

```
     29.95    35.06    66.20   198.48   199.16   211.54
    606.76   632.77   635.35   699.57   757.78   759.49
    835.87   902.00   902.54  1283.50  1283.75  1288.76
   1391.78  1391.88  1393.79  1394.58  1400.83  1401.85
   3028.54  3028.98  3032.11  3110.78  3111.84  3112.05
   3166.21  3166.77  3167.63
```

==============================
19B Ph3SiH
==============================

```
  13.09    31.67    35.61    45.99    57.49    58.06
 151.74   158.40   176.32   219.34   235.12   251.43
 394.13   395.99   400.63   402.21   421.11   430.71
 478.61   491.98   501.22   623.62   624.15   625.18
 686.88   702.58   706.78   709.69   711.37   711.66
 741.54   743.06   754.38   813.81   829.89   863.37
 867.11   867.96   923.99   928.92   933.46   973.23
 976.69   979.93   998.14   998.92  1000.01  1004.87
1005.00  1005.54  1055.45  1055.80  1056.76  1097.41
1099.81  1101.06  1131.27  1138.58  1140.56  1163.11
1163.25  1163.34  1194.43  1195.70  1198.14  1312.56
1317.11  1317.67  1361.50  1364.29  1364.77  1458.91
1461.90  1462.90  1514.59  1515.12  1516.39  1637.99
1640.22  1640.40  1663.61  1664.02  1664.55  2249.71
3136.36  3140.80  3146.44  3146.89  3149.27  3151.30
3166.55  3166.93  3168.09  3174.57  3175.23  3177.26
3189.05  3189.34  3189.86
```

==============================
20B Ph3Si+
==============================

```
  23.91    30.26    43.33    51.72    59.30    96.29
 142.55   147.18   161.86   236.90   243.79   251.46
 327.51   392.48   392.94   402.06   428.24   435.88
 463.48   497.51   506.72   613.85   616.38   617.81
 681.14   686.89   693.41   704.51   741.66   741.94
 752.39   754.81   755.24   858.75   863.12   869.83
 943.83   955.50   962.76   989.31   992.31   996.89
1000.56  1001.93  1005.58  1021.66  1025.53  1026.63
1052.93  1055.29  1055.95  1101.94  1109.96  1118.75
1122.88  1142.14  1145.64  1174.39  1174.97  1175.78
1201.25  1201.42  1209.94  1306.66  1319.42  1323.17
1369.06  1380.73  1384.41  1455.73  1461.72  1464.78
1502.39  1506.99  1509.75  1623.18  1627.19  1632.57
1650.60  1654.14  1656.47  3158.71  3159.82  3179.64
3180.90  3187.98  3191.69  3191.83  3194.12  3199.93
3201.85  3205.70  3207.29  3209.41  3211.41  3212.87
```

==============================
20B Me3SiH
==============================

```
 137.73   147.51   148.11   179.14   179.58   213.57
 595.97   596.23   622.66   659.45   707.50   707.88
 841.41   841.96   869.11   918.57   918.84  1275.97
1276.09  1283.60  1434.51  1436.73  1436.90  1448.04
1448.13  1454.70  2245.47  3035.41  3037.06  3037.22
3129.34  3129.91  3129.98  3138.36  3138.42  3138.85
```

==============================
00-CH3+
==============================

```
1354.14  1354.50  1373.54  2982.55  3201.71  3201.91
```

==============================
00-CH4
==============================

```
1318.85  1319.08  1320.24  1541.39  1541.46  3038.86
3176.60  3177.37  3179.67
```

==============================
00-toluene
==============================

```
  40.16   209.42   343.11   410.78   468.02   524.42
 627.52   704.29   735.76   804.42   851.40   900.93
 963.70   988.84   993.51  1008.54  1046.96  1058.59
1107.28  1160.64  1186.44  1244.19  1326.82  1374.63
```

1400.91 1457.06 1465.30 1495.19 1535.92 1660.59
1684.21 3029.24 3104.91 3127.60 3156.72 3157.26
3172.16 3181.51 3193.53

==============================
1C
==============================
53.45 60.86 69.93 102.18 118.24 196.56
239.47 292.97 381.40 387.98 424.54 612.52
647.02 671.90 679.29 725.02 741.98 752.31
789.99 833.11 853.62 874.74 955.12 991.05
1001.65 1029.78 1051.75 1114.63 1149.90 1177.44
1203.55 1285.19 1290.89 1318.09 1390.77 1400.96
1406.22 1409.47 1409.98 1463.34 1504.00 1622.15
1651.80 3039.48 3041.29 3124.28 3128.97 3162.39
3168.61 3174.16 3175.38 3193.92 3208.31 3212.86

==============================
1CH
==============================
29.05 83.74 131.57 145.02 152.73 177.02
230.75 287.20 380.94 398.96 466.82 602.96
624.44 648.02 693.84 705.08 710.71 728.17
737.73 778.27 852.55 864.06 899.77 901.39
924.73 973.17 997.55 1006.30 1056.48 1098.69
1139.87 1162.53 1195.40 1275.82 1280.78 1316.18
1364.74 1434.03 1436.75 1443.45 1447.96 1461.09
1517.15 1641.94 1665.60 2253.18 3036.55 3037.69
3128.31 3129.49 3134.41 3143.43 3144.23 3151.49
3168.38 3178.74 3190.75

==============================
2C
==============================
65.81 81.78 120.16 151.75 283.47 380.20
404.92 410.59 518.11 610.15 669.80 673.51
722.24 752.06 763.26 796.79 853.57 910.29
960.07 994.32 1000.89 1031.82 1049.09 1115.23
1154.34 1178.71 1204.47 1287.44 1317.92 1393.92
1395.72 1403.13 1464.28 1499.97 1615.23 1650.64
2386.68 3038.63 3123.51 3168.77 3172.47 3179.46
3194.49 3210.49 3214.36

==============================
2CH
==============================
14.94 100.44 140.24 184.59 246.02 381.29
398.29 442.20 485.62 624.53 672.87 683.90
707.98 710.48 733.47 770.48 862.90 884.40
913.64 926.03 964.84 972.70 998.25 1006.64
1057.11 1098.93 1138.83 1163.15 1195.54 1278.88
1316.40 1364.77 1441.02 1442.63 1461.64 1517.25
1643.07 1665.46 2260.53 2267.91 3046.58 3141.52
3144.45 3150.63 3153.18 3169.45 3179.99 3191.52

==============================
3C
==============================
32.95 44.26 74.10 78.91 92.89 147.75
148.72 223.30 249.92 277.34 366.57 389.61
399.51 435.47 441.37 464.21 614.51 615.60
672.15 686.28 692.93 727.28 739.77 752.39
754.48 787.30 824.59 857.41 864.30 949.99
958.93 990.15 996.43 1001.08 1004.55 1026.84
1027.92 1053.57 1054.23 1108.45 1115.27 1137.57
1149.62 1176.02 1176.26 1201.54 1204.92 1287.23
1311.48 1322.51 1382.21 1385.07 1408.69 1414.22
1460.24 1464.98 1503.72 1506.75 1624.18 1627.00
1650.47 1655.27 3044.09 3131.79 3168.68 3169.30

3173.16 3177.82 3184.24 3193.07 3193.50 3205.88
3206.07 3211.29 3211.55

==============================
3CH
==============================
17.45 34.08 51.56 94.28 124.50 144.25
170.18 209.17 236.21 285.58 386.32 398.09
401.06 422.28 460.23 481.06 623.92 624.59
656.25 691.09 702.49 707.13 709.73 729.14
735.50 751.35 773.95 847.88 864.17 868.88
896.65 924.86 930.26 973.56 978.28 998.18
1000.04 1004.18 1005.91 1055.00 1056.38 1098.31
1100.05 1132.84 1141.10 1162.65 1162.98 1195.57
1197.50 1278.88 1314.49 1315.94 1360.57 1363.44
1437.92 1439.56 1459.68 1461.28 1514.68 1516.46
1636.05 1640.55 1662.93 1664.51 2248.15 3038.62
3128.43 3133.18 3138.06 3141.39 3145.53 3151.30
3161.98 3168.92 3169.65 3178.88 3186.81 3191.92

==============================
4C
==============================
23.91 30.26 43.33 51.72 59.30 96.29
142.55 147.18 161.86 236.90 243.79 251.46
327.51 392.48 392.94 402.06 428.24 435.88
463.48 497.51 506.72 613.85 616.38 617.81
681.14 686.89 693.41 704.51 741.66 741.94
752.39 754.81 755.24 858.75 863.12 869.83
943.83 955.50 962.76 989.31 992.31 996.89
1000.56 1001.93 1005.58 1021.66 1025.53 1026.63
1052.93 1055.29 1055.95 1101.94 1109.96 1118.75
1122.88 1142.14 1145.64 1174.39 1174.97 1175.78
1201.25 1201.42 1209.94 1306.66 1319.42 1323.17
1369.06 1380.73 1384.41 1455.73 1461.72 1464.78
1502.39 1506.99 1509.75 1623.18 1627.19 1632.57
1650.60 1654.14 1656.47 3158.71 3159.82 3179.64
3180.90 3187.98 3191.69 3191.83 3194.12 3199.93
3201.85 3205.70 3207.29 3209.41 3211.41 3212.87

==============================
4CH
==============================
29.05 83.74 131.57 145.02 152.73 177.02
230.75 287.20 380.94 398.96 466.82 602.96
624.44 648.02 693.84 705.08 710.71 728.17
737.73 778.27 852.55 864.06 899.77 901.39
924.73 973.17 997.55 1006.30 1056.48 1098.69
1139.87 1162.53 1195.40 1275.82 1280.78 1316.18
1364.74 1434.03 1436.75 1443.45 1447.96 1461.09
1517.15 1641.94 1665.60 2253.18 3036.55 3037.69
3128.31 3129.49 3134.41 3143.43 3144.23 3151.49
3168.38 3178.74 3190.75

==============================
5C
==============================
29.95 35.06 66.20 198.48 199.16 211.54
606.76 632.77 635.35 699.57 757.78 759.49
835.87 902.00 902.54 1283.50 1283.75 1288.76
1391.78 1391.88 1393.79 1394.58 1400.83 1401.85
3028.54 3028.98 3032.11 3110.78 3111.84 3112.05
3166.21 3166.77 3167.63

==============================
5CH
==============================
137.73 147.51 148.11 179.14 179.58 213.57
595.97 596.23 622.66 659.45 707.50 707.88

```
  841.41   841.96   869.11   918.57   918.84  1275.97
 1276.09  1283.60  1434.51  1436.73  1436.90  1448.04
 1448.13  1454.70  2245.47  3035.41  3037.06  3037.22
 3129.34  3129.91  3129.98  3138.36  3138.42  3138.85
```

===============================

6C

===============================

```
   27.82    37.73    77.02   132.21   160.78   198.66
  244.56   382.55   392.02   411.14   420.96   442.23
  464.45   572.34   612.69   614.64   682.30   684.47
  703.06   737.95   751.91   752.60   840.53   856.22
  859.31   952.67   957.32   991.08   993.23  1000.54
 1003.18  1028.22  1028.49  1051.11  1052.14  1108.84
 1115.44  1137.50  1154.03  1176.65  1177.14  1201.85
 1205.42  1311.94  1321.56  1385.24  1388.62  1461.18
 1465.72  1500.66  1504.71  1619.99  1623.43  1648.06
 1655.03  2377.40  3167.96  3168.00  3180.04  3185.20
 3194.02  3194.36  3207.66  3207.82  3212.60  3212.77
```

===============================

6CH

===============================

```
    3.88    25.74    49.84   149.26   157.02   194.18
  239.53   385.60   397.47   400.92   421.00   423.22
  477.63   602.82   624.10   624.98   691.92   704.86
  707.08   708.86   717.09   748.88   770.68   861.95
  864.88   868.99   928.30   928.95   966.17   974.97
  975.73   999.07   999.11  1005.95  1006.39  1055.84
 1056.83  1098.73  1099.62  1134.86  1140.26  1163.26
 1163.38  1194.69  1196.32  1314.76  1318.10  1364.55
 1365.81  1460.63  1463.28  1515.97  1516.64  1641.61
 1642.92  1665.08  1665.22  2259.83  2262.86  3151.07
 3152.15  3152.55  3153.91  3170.24  3170.51  3181.36
 3181.71  3192.24  3192.60
```

===============================

7C

===============================

```
   34.17    51.67   212.35   448.03   600.13   622.48
  661.77   811.24   839.50   877.26   946.68  1278.48
 1284.36  1378.59  1380.99  1392.11  1394.32  2394.62
 3020.73  3023.94  3101.48  3103.63  3169.30  3170.19
```

===============================

7CH

===============================

```
  145.99   156.47   189.09   447.48   566.22   625.17
  659.57   726.11   854.06   882.22   887.41   933.37
  975.64  1281.12  1286.16  1438.14  1441.87  1448.73
 1451.45  2259.19  2264.51  3042.39  3043.78  3136.63
 3137.02  3143.60  3144.12
```

===============================

1CAn1 6A‘

===============================

```
   12.29    25.87    39.20    46.39    52.62    68.32
   74.66    75.94    78.50    79.38    84.78    84.97
   89.57    99.16   117.32   146.71   160.07   168.06
  173.10   189.29   195.95   196.11   196.75   206.41
  240.77   242.99   253.01   278.00   302.77   310.26
  323.97   330.30   385.07   394.34   457.83   539.06
  541.34   582.60   582.64   597.21   599.42   613.59
  620.22   631.99   639.03   645.81   652.12   697.62
  701.23   710.07   714.56   724.72   739.24   741.53
  745.07   754.18   763.77   781.00   794.68   803.33
  810.82   814.16   819.77   821.75   822.20   823.34
  829.19   837.34   839.32   854.49   859.68   866.51
  868.06   870.62   902.66   905.46   905.81   916.01
```

```
  933.53   938.15   940.89   945.06   954.90   962.65
  974.46   983.74   987.42  1004.18  1009.80  1056.20
 1059.21  1106.67  1121.66  1125.15  1142.16  1169.01
 1198.84  1287.68  1294.99  1319.38  1370.86  1414.20
 1417.63  1429.67  1432.33  1462.75  1514.86  1639.49
 1661.41  2696.07  2708.59  2719.50  2724.60  2735.32
 3036.15  3036.85  3132.16  3135.15  3144.52  3146.98
 3154.45  3159.31  3181.39  3193.53  3201.67  3202.47
```

==============================

1Ctol

==============================

```
   26.99    46.38    53.15    78.79    87.48   122.24
  131.54   150.70   165.17   175.63   183.29   195.60
  196.60   258.67   295.11   310.89   349.28   382.72
  395.98   402.41   462.22   477.58   522.47   611.58
  620.45   654.29   690.77   693.73   702.06   724.53
  745.58   751.87   784.11   803.36   805.99   812.88
  852.86   859.85   864.16   938.19   941.97   982.75
  987.02   993.62  1000.79  1004.73  1016.56  1017.77
 1027.38  1037.52  1055.86  1097.54  1106.79  1139.98
 1158.08  1170.74  1191.14  1199.39  1251.59  1287.53
 1296.07  1318.32  1324.00  1371.20  1386.45  1404.36
 1419.66  1423.44  1430.53  1436.38  1444.01  1458.42
 1460.82  1492.26  1504.84  1513.70  1594.57  1636.47
 1659.05  1663.23  3023.25  3041.78  3043.68  3109.08
 3131.21  3134.99  3136.01  3149.49  3152.36  3156.72
 3157.51  3158.42  3181.16  3185.53  3185.93  3195.92
 3196.48  3198.68  3204.34
```

==============================

2CAn1

==============================

```
    3.47     6.67    24.88    41.65    58.50    69.24
   69.57    76.04    77.29    78.54    81.44    86.73
   90.73    98.98   125.89   149.90   161.85   177.62
  196.65   197.25   201.07   238.98   240.94   253.44
  282.52   301.77   311.01   324.68   330.80   393.48
  401.11   455.80   538.77   540.79   582.37   582.56
  597.02   600.18   613.46   619.97   631.47   638.47
  645.56   656.89   698.15   699.53   709.38   712.97
  727.30   738.38   741.60   750.43   760.68   764.18
  782.94   791.56   802.66   808.50   818.03   821.09
  821.87   824.23   829.06   836.99   838.65   852.69
  861.67   864.71   868.24   888.34   899.18   902.74
  907.78   912.30   934.77   936.73   940.13   944.59
  953.49   964.90   975.88   983.76   986.80  1004.92
 1011.93  1055.85  1057.56  1106.26  1120.17  1124.00
 1141.61  1169.46  1197.87  1291.06  1319.52  1371.71
 1416.02  1418.68  1463.62  1514.49  1640.41  1661.16
 2354.63  2703.79  2714.70  2734.55  2738.16  2743.68
 3039.53  3141.07  3147.04  3151.58  3161.40  3181.90
 3194.08  3201.96  3207.16
```

==============================

2Ctol

==============================

```
   25.48    42.73    59.70    74.03    81.39   129.92
  135.90   156.57   174.19   190.84   244.44   291.03
  331.77   349.24   396.21   397.23   399.60   460.45
  481.36   522.40   610.07   619.93   667.90   693.11
  701.40   703.22   731.21   749.78   759.55   783.08
  803.92   814.31   849.93   865.81   898.03   942.25
  944.22   984.34   985.73   993.85  1000.59  1004.52
 1017.67  1017.83  1025.39  1036.00  1054.75  1095.43
 1105.98  1141.28  1158.32  1171.15  1191.47  1198.99
 1251.82  1292.16  1318.94  1323.60  1371.06  1384.06
 1405.57  1424.73  1426.62  1443.05  1457.81  1461.11
 1494.43  1502.36  1512.82  1589.57  1635.70  1658.00
```

1662.95 2328.40 3023.97 3046.68 3109.79 3124.13
3144.13 3151.50 3152.18 3155.47 3160.44 3182.64
3184.72 3186.29 3194.84 3197.33 3197.65 3204.72

==============================
3CAn1 6B‘ 13A‘
==============================
6.20 20.06 24.94 34.10 39.63 52.56
61.61 68.37 69.45 74.36 76.98 79.29
79.68 86.04 88.92 90.96 98.61 143.49
150.56 159.97 174.31 193.45 196.12 196.43
208.57 223.73 240.99 243.39 248.13 252.19
302.45 323.26 326.19 330.68 371.47 393.58
399.58 448.54 465.47 472.23 538.82 541.64
581.56 582.74 596.34 597.57 613.35 619.66
621.36 631.55 636.70 644.59 673.85 702.10
705.29 709.31 713.54 717.13 738.93 739.97
743.97 747.16 760.20 762.98 782.55 791.60
793.35 802.36 810.41 814.30 817.39 820.99
822.54 829.02 833.28 838.16 852.74 861.78
864.80 867.52 868.91 898.34 900.79 902.05
914.57 934.47 934.64 939.75 941.00 943.95
953.52 959.73 976.20 981.35 982.10 985.70
1002.93 1003.89 1009.32 1011.42 1054.63 1057.08
1057.49 1104.22 1106.97 1119.77 1124.26 1133.70
1145.38 1168.46 1169.15 1199.04 1200.78 1287.59
1316.40 1321.48 1368.37 1370.06 1415.72 1420.31
1459.76 1463.44 1514.15 1514.87 1637.19 1639.77
1659.87 1661.54 2712.68 2725.12 2731.41 2734.13
2739.63 3040.23 3137.78 3150.77 3152.04 3154.17
3157.88 3164.15 3179.55 3181.37 3190.89 3192.54
3199.41 3201.26 3207.14

==============================
3Ctol1
==============================
21.85 28.81 41.42 60.52 62.85 77.69
92.77 97.36 135.83 149.59 161.54 179.63
195.72 202.48 219.75 246.62 270.79 315.31
350.18 378.42 394.09 401.32 403.81 450.60
467.71 472.08 477.73 524.30 611.21 620.49
621.87 672.38 693.41 701.25 707.02 715.19
738.51 751.77 757.40 771.64 793.67 810.12
811.48 850.36 860.06 868.89 920.90 935.19
945.94 981.91 986.54 988.99 992.08 1000.73
1003.03 1004.36 1015.77 1016.55 1017.40 1026.57
1035.34 1055.82 1057.91 1095.28 1103.85 1109.51
1131.63 1142.65 1156.14 1170.91 1171.79 1191.36
1198.30 1202.48 1251.77 1288.56 1313.11 1321.21
1323.35 1368.32 1370.20 1387.05 1401.90 1424.89
1428.93 1444.49 1458.82 1460.31 1463.11 1490.96
1504.96 1513.12 1513.70 1594.03 1635.76 1638.20
1658.20 1659.47 1664.95 3023.61 3042.64 3112.69
3125.41 3134.86 3139.09 3153.27 3158.55 3161.35
3162.42 3174.56 3184.28 3184.70 3184.89 3187.69
3195.51 3196.88 3197.04 3198.74 3203.55 3206.00

==============================
4CAn1 13B‘ 20B‘
==============================
7.94 18.56 25.74 32.07 40.99 45.10
49.67 54.69 60.54 64.76 69.18 74.47
76.63 80.88 81.24 82.19 86.81 89.73
92.85 98.30 166.21 168.54 189.14 194.49
195.75 196.11 207.33 234.51 241.40 243.82
247.87 250.49 252.62 301.99 322.77 329.91
357.56 395.75 397.83 404.52 441.67 448.94
483.79 498.03 503.46 538.69 540.65 581.38
582.20 595.68 599.58 611.03 618.59 620.23

620.74	630.79	636.40	642.94	683.22	701.27
703.54	706.64	708.59	712.99	730.75	734.52
738.34	739.07	748.84	751.54	752.55	764.58
781.43	790.81	800.96	810.10	817.93	819.93
823.50	827.58	830.59	837.82	851.59	860.82
861.71	865.33	868.48	869.95	898.20	900.35
901.32	914.63	933.51	936.67	937.58	939.90
942.76	944.17	953.28	958.74	977.05	980.24
980.75	983.81	983.98	1001.63	1002.88	1003.78
1009.05	1009.95	1010.93	1052.31	1055.00	1055.88
1057.23	1101.69	1105.33	1105.78	1119.85	1122.46
1123.76	1139.68	1143.49	1167.21	1168.14	1168.84
1198.36	1199.08	1201.38	1313.78	1318.59	1320.44
1366.87	1368.14	1368.87	1457.16	1461.00	1462.39
1511.35	1512.71	1513.53	1634.88	1636.57	1638.10
1658.68	1659.37	1659.89	2716.39	2731.81	2735.51
2740.75	2746.23	3154.37	3154.46	3157.35	3163.24
3165.79	3177.58	3180.14	3180.44	3183.13	3191.24
3191.30	3191.76	3199.48	3200.19	3200.45	3209.44

==============================

4Ctol

==============================

18.60	27.01	29.51	39.09	50.52	61.65
66.79	75.07	87.31	91.55	109.23	139.03
169.51	187.01	200.04	204.96	237.94	245.91
253.03	272.21	345.79	349.97	394.96	398.70
404.14	411.83	443.66	452.78	473.48	487.45
496.34	504.00	524.13	612.74	619.68	621.96
622.58	681.17	694.66	703.12	707.11	710.11
726.87	729.12	748.72	751.71	753.39	788.34
809.76	849.80	861.25	867.98	873.60	916.06
935.67	945.61	952.42	983.34	985.16	988.53
990.32	994.38	1001.91	1002.45	1003.74	1004.18
1015.50	1015.87	1016.31	1019.66	1026.34	1035.77
1056.86	1057.12	1058.08	1094.92	1102.17	1108.28
1109.81	1123.04	1137.55	1140.34	1154.97	1170.07
1171.28	1171.34	1190.53	1199.08	1200.99	1204.91
1251.02	1310.98	1317.62	1321.41	1323.34	1367.86
1369.36	1370.71	1386.99	1402.86	1445.13	1456.93
1459.16	1462.09	1462.71	1489.10	1505.70	1511.50
1512.81	1513.91	1596.61	1633.86	1636.59	1637.72
1657.69	1659.28	1659.66	1664.82	3028.60	3112.16
3128.00	3143.30	3154.78	3157.46	3162.50	3172.40
3173.42	3179.52	3183.84	3185.12	3185.99	3186.97
3187.16	3195.70	3196.41	3196.46	3197.36	3203.01
3203.80	3205.50	3205.69			

==============================

5CAn1 13A‘ 19B‘

==============================

36.46	44.96	48.94	68.96	71.08	75.55
77.93	78.75	84.10	84.38	92.11	99.05
129.43	137.70	151.50	160.65	163.62	177.96
196.68	197.21	198.25	199.00	210.29	240.92
246.15	255.26	289.79	303.46	324.20	330.86
538.14	540.58	581.94	582.29	596.32	598.28
612.97	617.26	631.78	638.83	644.22	684.48
693.91	694.42	709.64	712.39	737.70	741.13
762.37	768.89	774.24	781.71	791.10	804.37
808.46	818.61	821.55	821.90	827.95	832.07
837.74	851.40	853.76	862.73	866.08	879.20
885.17	898.39	904.00	906.50	910.22	936.19
939.57	943.34	952.53	962.26	982.67	986.84
1056.14	1119.51	1123.75	1283.94	1287.21	1294.03
1408.32	1409.26	1418.84	1423.40	1429.85	1437.15
2704.59	2720.98	2735.26	2741.26	2746.73	3040.48
3041.12	3042.16	3135.91	3136.48	3140.49	3152.87
3157.76	3158.09	3208.68			

===============================
5Ctol
===============================

```
  34.63    50.94    70.34   114.45   133.25   152.20
 170.47   173.03   181.55   193.57   210.56   215.36
 237.32   336.06   351.00   401.76   488.84   529.17
 610.99   621.39   684.84   689.36   696.78   706.15
 762.10   769.63   800.48   808.76   837.23   855.95
 871.54   875.94   937.31   986.13   995.54  1005.23
1020.01  1030.10  1039.48  1094.48  1157.69  1193.79
1251.61  1288.83  1290.50  1298.87  1323.94  1386.83
1406.59  1416.24  1420.22  1421.52  1429.56  1434.39
1440.25  1445.10  1458.78  1490.71  1503.39  1590.14
1664.56  3036.89  3039.36  3040.09  3045.33  3112.98
3116.56  3130.63  3131.92  3135.99  3148.94  3149.81
3156.67  3158.53  3183.73  3187.05  3196.49  3199.60
```

===============================
6CAn1
===============================

```
   4.20    17.71    29.39    36.92    45.34    52.42
  57.23    66.15    69.37    75.15    76.79    80.46
  83.23    84.87    86.70    90.19    98.94   167.06
 172.53   186.33   196.33   196.86   199.67   237.54
 240.78   244.31   251.85   302.52   323.12   329.74
 332.61   391.62   397.02   407.81   448.33   461.82
 470.78   538.38   541.02   581.11   582.59   595.74
 598.79   613.93   619.62   621.09   631.39   636.95
 644.70   697.01   699.80   701.41   708.28   712.68
 723.58   735.11   738.62   742.35   746.91   760.36
 766.47   785.22   790.33   803.59   808.16   818.74
 820.42   821.61   822.10   827.58   832.55   836.81
 850.30   860.07   862.15   863.80   868.57   897.62
 899.56   906.93   912.09   932.76   934.99   938.00
 938.69   942.63   953.11   962.38   974.79   978.37
 980.26   985.02  1003.39  1003.98  1010.05  1011.05
1054.69  1055.65  1057.05  1105.25  1107.09  1117.69
1122.13  1132.92  1148.70  1168.80  1169.43  1197.83
1200.42  1317.29  1320.62  1370.23  1372.13  1461.04
1464.41  1512.78  1513.17  1636.95  1639.62  1659.78
1660.97  2334.24  2719.07  2732.23  2740.73  2743.37
2748.30  3151.49  3157.97  3160.04  3171.19  3181.12
3181.84  3193.59  3193.63  3201.42  3201.72  3212.96
```

===============================
6Ctol
===============================

```
  21.72    30.70    34.90    46.62    67.87    72.77
  81.53    99.08   134.51   175.49   183.03   193.32
 232.36   269.32   335.25   348.90   392.87   394.57
 400.70   421.76   443.78   467.50   471.28   476.53
 521.60   611.64   618.82   621.34   690.04   694.69
 700.25   704.69   720.58   740.03   747.77   758.85
 791.01   807.88   827.82   849.32   863.58   864.34
 936.67   941.28   941.78   982.63   983.90   987.97
 991.21  1001.17  1002.33  1004.42  1016.01  1016.62
1019.02  1025.66  1037.01  1055.14  1055.75  1096.64
1102.59  1109.05  1131.29  1143.54  1156.68  1170.97
1172.39  1189.84  1196.23  1202.22  1250.70  1312.80
1320.59  1323.29  1370.01  1372.35  1386.80  1405.51
1444.28  1458.36  1459.61  1463.48  1491.66  1504.03
1511.11  1513.25  1594.46  1635.05  1638.11  1657.17
1658.68  1662.35  2325.34  3025.18  3109.21  3138.25
3151.31  3152.60  3158.60  3162.66  3170.15  3182.40
3185.21  3186.10  3188.27  3196.72  3197.13  3198.59
3198.94  3204.44  3207.00
```

==============================
7CAn1
==============================

```
  15.34   44.36   50.51   68.19   69.86   75.37
  77.67   78.67   82.45   84.88   90.59   98.61
 131.81  142.32  144.86  164.64  188.80  196.83
 197.33  210.94  239.08  240.94  252.36  302.24
 309.10  323.69  331.41  537.41  540.55  581.60
 582.19  596.12  598.14  613.29  625.25  629.97
 634.94  638.59  644.19  679.27  704.88  708.45
 712.75  737.49  740.21  760.14  779.74  788.09
 790.05  801.29  807.66  818.07  820.45  821.89
 828.71  831.53  837.04  851.52  855.98  865.64
 867.63  870.92  897.76  901.29  904.23  909.55
 914.25  935.60  939.39  943.49  953.54  963.57
 982.97  987.56 1055.88 1120.02 1124.08 1287.99
1293.40 1417.91 1419.88 1427.68 1434.14 2366.66
2706.48 2720.60 2737.10 2743.57 2749.26 3041.62
3045.66 3138.90 3140.79 3154.92 3158.72 3207.73
```

==============================
7Ctol
==============================

```
  45.42   51.09   75.28  118.63  142.34  159.17
 189.81  193.07  217.30  218.68  348.98  362.10
 402.82  493.18  525.59  610.05  627.89  659.39
 697.05  702.79  710.46  782.94  798.94  811.67
 858.09  859.77  864.45  906.87  933.86  984.73
 994.76 1007.82 1018.11 1028.82 1039.24 1092.28
1158.27 1193.80 1251.44 1291.32 1297.87 1324.02
1385.23 1407.67 1418.79 1420.80 1429.92 1435.52
1443.64 1456.99 1491.99 1501.81 1586.94 1661.85
2341.80 3037.09 3042.61 3043.74 3111.70 3118.51
3132.54 3134.29 3157.27 3158.35 3160.31 3185.15
3188.69 3197.44 3201.56
```

==============================
1CAn2 6A“
==============================

```
  29.84   41.93   54.80   58.79   65.98   69.71
  74.92   81.39   82.57   84.71   85.79   93.06
  94.51  103.75  138.58  153.99  167.16  171.58
 189.38  196.42  197.23  213.28  216.44  237.34
 240.06  248.42  254.68  278.80  288.36  306.66
 324.22  330.47  385.57  398.48  463.00  532.18
 534.01  577.61  578.65  586.35  603.43  605.49
 621.08  629.74  643.74  644.59  648.78  703.35
 705.44  707.28  718.54  729.67  734.74  735.79
 754.79  758.71  762.04  788.12  791.01  792.34
 804.19  805.57  808.94  813.45  814.89  826.19
 828.47  830.78  839.61  843.27  863.25  872.66
 875.52  877.25  903.92  911.67  912.01  913.40
 939.86  942.03  946.76  948.56  950.97  951.76
 993.98  995.01  998.97 1004.07 1028.16 1040.61
1059.60 1098.97 1130.49 1138.95 1139.02 1167.53
1199.11 1282.93 1289.85 1295.53 1343.85 1426.13
1429.63 1433.01 1439.17 1451.51 1505.37 1600.48
1621.48 2675.92 2677.06 2681.12 2681.60 2687.38
2986.79 2989.38 3066.08 3074.59 3076.21 3082.18
3092.03 3104.11 3114.27 3127.02 3134.01 3134.53
```

==============================
2CAn2
==============================

```
  16.59   32.28   48.14   57.12   58.05   70.08
  73.73   76.50   81.43   84.94   85.33   86.56
  95.68   98.55  115.97  137.51  159.72  175.19
 194.95  197.15  198.12  237.52  241.87  251.61
 278.39  284.72  294.89  326.17  331.83  391.64
```

400.30	452.02	536.95	538.96	583.78	584.67
595.86	605.28	606.84	619.25	629.23	643.53
645.96	663.92	695.04	701.17	709.84	712.82
737.57	742.39	743.46	754.22	755.73	767.31
792.09	792.92	794.64	809.44	810.88	818.02
820.52	828.67	829.40	838.66	841.66	842.66
855.87	869.33	872.34	900.51	901.67	902.57
904.02	904.86	933.00	936.58	939.16	944.12
948.12	950.67	971.46	988.32	990.75	1004.34
1008.58	1055.18	1055.74	1107.45	1122.50	1122.62
1141.74	1168.99	1198.96	1284.75	1321.12	1373.73
1413.59	1422.35	1463.87	1514.23	1640.39	1661.74
2325.76	2733.42	2734.62	2738.63	2738.67	2744.15
3041.03	3142.08	3152.81	3159.11	3170.77	3181.18
3194.88	3202.02	3206.32			

==============================

3CAn2 6B“ 13A“

==============================

20.58	27.69	37.42	44.85	48.41	54.64
65.15	71.13	74.12	76.55	81.81	82.53
85.76	86.75	92.92	95.07	98.74	142.42
154.74	167.77	189.30	196.29	196.88	197.98
212.16	221.85	238.57	240.55	249.34	251.74
287.66	292.89	327.22	329.85	380.60	393.23
401.95	453.28	460.91	464.23	537.53	538.24
583.11	584.39	595.66	605.46	606.18	619.94
621.05	630.80	642.87	644.75	671.83	696.31
702.15	710.89	711.68	721.27	737.24	741.76
742.41	747.34	755.32	759.07	784.66	791.80
792.60	804.91	809.70	810.14	818.80	819.55
822.52	828.52	828.62	839.60	841.19	856.84
862.84	868.27	870.24	901.24	901.44	902.38
903.67	932.18	936.85	938.49	939.80	944.58
946.45	949.27	973.04	979.74	987.48	988.69
1002.28	1003.50	1008.22	1010.46	1055.19	1057.31
1057.77	1105.05	1110.08	1122.22	1122.48	1131.66
1145.31	1168.32	1169.24	1199.36	1203.03	1287.53
1315.67	1323.37	1368.34	1372.76	1425.20	1432.24
1459.55	1464.20	1513.65	1514.02	1637.49	1639.73
1660.86	1661.43	2731.68	2733.29	2737.05	2737.38
2742.81	3040.46	3137.69	3151.55	3157.11	3163.09
3165.68	3174.97	3178.27	3180.77	3189.66	3192.78
3198.16	3200.07	3206.57			

==============================

4CAn2 13B“ 20B“

==============================

0.85	19.63	32.84	35.15	38.92	45.05
49.63	55.79	58.50	71.50	72.21	76.50
80.15	82.94	84.59	85.59	85.91	95.39
102.97	109.74	155.54	168.33	187.41	194.02
196.02	197.46	207.37	230.27	239.00	240.23
245.55	251.81	255.21	285.85	328.04	329.13
350.56	391.26	402.04	406.07	431.70	455.10
475.03	500.19	503.90	537.71	538.37	583.14
584.53	595.73	605.44	606.78	619.35	621.74
622.10	632.84	641.59	645.09	682.20	698.94
702.73	709.38	711.09	711.81	725.29	736.19
741.44	742.36	747.62	748.81	753.09	758.62
791.40	792.50	796.56	809.28	810.32	818.43
820.12	827.89	828.23	840.30	841.63	857.58
865.73	867.16	868.10	870.14	900.17	901.72
903.18	904.10	933.09	936.77	937.29	938.88
944.38	946.23	948.23	948.40	975.04	981.74
984.54	987.55	988.19	1001.52	1002.48	1002.74
1007.08	1008.47	1010.55	1055.11	1056.63	1056.76
1057.62	1101.43	1108.52	1110.45	1122.15	1122.42
1122.49	1137.47	1142.37	1166.98	1168.62	1168.77

```
   1199.50  1201.98  1206.42  1311.55  1321.07  1322.89
   1363.78  1367.23  1370.86  1456.67  1461.86  1464.13
   1512.60  1513.23  1514.73  1634.23  1637.26  1638.30
   1660.24  1660.87  1661.46  2730.85  2732.28  2736.29
   2736.33  2741.93  3154.14  3158.97  3160.31  3166.46
   3168.54  3175.06  3177.52  3178.89  3181.22  3185.78
   3187.85  3192.02  3196.66  3198.42  3199.50  3205.40
```

===============================

5CAn2 13A" 19B"

===============================

```
     33.46    57.48    57.74    70.56    72.22    77.32
     81.06    85.28    86.24    87.64    89.72    97.98
    118.73   127.24   145.69   158.17   163.97   169.69
    196.03   197.28   205.77   209.04   226.85   237.64
    241.40   252.07   276.49   288.95   326.59   333.22
    537.09   539.06   583.92   585.15   596.30   605.77
    607.09   614.12   632.12   644.65   645.92   690.67
    691.74   697.61   709.83   713.11   741.90   743.62
    756.78   765.53   781.77   791.91   792.83   794.33
    809.42   810.56   818.34   820.36   828.88   829.04
    838.79   842.06   854.96   869.35   871.30   883.89
    888.60   900.48   901.85   904.18   905.64   936.72
    939.39   943.81   948.25   950.47   988.42   990.05
   1055.07  1122.71  1122.78  1284.23  1285.77  1293.37
   1405.64  1413.56  1417.07  1420.67  1430.37  1437.94
   2734.15  2735.39  2739.35  2739.45  2744.88  3036.36
   3037.09  3039.23  3133.65  3133.91  3134.47  3151.38
   3158.71  3159.08  3206.61
```

===============================

6CAn2

===============================

```
      7.59    13.27    30.77    32.36    43.01    52.80
     61.11    64.36    70.10    76.51    76.98    81.40
     82.78    85.46    88.78    93.22   101.38   160.26
    169.29   186.47   195.51   197.19   205.80   238.78
    239.61   245.24   251.70   287.33   311.82   327.40
    334.54   393.93   399.87   403.44   447.60   458.70
    475.09   537.68   538.77   583.75   585.05   596.00
    605.77   606.41   619.13   620.00   630.30   644.09
    645.61   691.52   700.04   700.70   705.61   710.49
    712.59   718.01   742.65   742.85   748.93   751.47
    759.19   792.25   792.69   798.64   800.16   809.59
    810.71   818.23   820.06   828.81   828.97   838.51
    841.43   863.69   867.60   869.05   871.73   900.76
    901.84   902.60   903.92   936.84   936.99   938.21
    943.33   944.83   947.26   951.12   978.51   982.77
    988.21   989.40  1002.22  1004.00  1010.39  1011.07
   1054.79  1054.99  1055.74  1104.43  1107.80  1122.54
   1122.91  1129.70  1144.90  1168.25  1169.09  1198.18
   1202.98  1317.28  1321.44  1367.65  1370.09  1459.93
   1464.12  1512.51  1513.78  1635.32  1638.76  1659.26
   1660.74  2400.69  2733.82  2734.62  2738.71  2738.84
   2744.19  3149.11  3151.38  3161.12  3161.95  3178.51
   3178.71  3189.24  3190.41  3198.79  3198.99  3206.74
```

===============================

7CAn2

===============================

```
     14.65    55.53    60.78    69.08    71.22    79.43
     79.96    82.82    85.52    86.68    87.89    99.25
    119.70   148.87   164.42   165.71   194.48   196.46
    197.37   218.73   237.69   240.85   251.33   284.49
    295.36   326.44   333.39   537.22   538.70   583.73
    585.07   595.84   605.01   606.47   622.57   629.51
    629.87   645.07   646.79   675.91   703.70   709.87
    712.78   742.44   743.57   756.43   786.48   792.03
    792.89   794.29   809.27   810.80   817.94   820.42
```

829.01	829.42	838.19	841.58	855.66	870.21
871.73	873.04	900.15	901.37	903.35	904.44
907.80	936.79	938.61	944.21	948.09	951.17
989.19	990.89	1054.94	1122.66	1122.86	1287.75
1293.11	1415.22	1418.52	1427.93	1434.35	2391.34
2734.79	2735.78	2739.75	2739.90	2745.22	3040.09
3042.82	3138.11	3138.88	3154.85	3156.06	3206.71

==============================
HB
==============================

17.72	31.32	48.96	56.96	98.36	111.18
124.16	133.88	137.35	140.70	146.64	150.98
152.84	180.97	192.55	195.89	203.77	219.77
287.56	302.87	377.30	397.15	456.42	607.40
614.19	619.55	647.33	674.88	684.23	697.92
702.69	722.44	733.64	746.60	758.15	760.63
783.99	810.58	844.83	857.86	863.82	865.20
880.63	897.44	941.29	985.22	996.21	1006.30
1020.41	1055.47	1107.44	1141.80	1172.78	1198.61
1286.03	1287.35	1288.57	1292.04	1296.32	1317.51
1373.40	1411.67	1413.48	1415.15	1419.18	1420.58
1425.48	1427.91	1429.29	1431.76	1437.48	1462.22
1512.47	1636.61	1656.97	1873.03	3039.72	3040.55
3044.14	3045.95	3046.83	3135.16	3135.63	3137.31
3139.99	3141.01	3146.42	3147.28	3153.37	3155.62
3156.13	3157.53	3161.72	3189.15	3199.36	3207.32

10 References

[S1] A. Schäfer, M. Reißmann, A. Schäfer, W. Saak, D. Haase and T. Müller, *Angew. Chem. Int. Ed.*, 2011, **50**, 12636–12638.

[S2] H. R. Wiltse, A. N. Johnson, R. J. Durand, W. Brennessel and R. M. Chin, *Organometallics*, 2016, **35**, 1079–1085.

[S3] J. Suffert, *Org. Chem.*, 1989, **54**, 509–510.

[S4] L. Omann and M. Oestreich, *Organometallics*, 2017, **36**, 767–776.

[S5] (*a*) C. Wang, G. Erker, G. Kehr, K. Wedeking and R. Fröhlich, *Organometallics*, 2005, **24**, 4760–4773; (*b*) J. B. Lambert, L. Lin and S. Keinan, *Org. Biomol. Chem.*, 2003, **1**, 2559–2565.

[S6] R. K. Harris, E. D. Becker, S. M. Cabral de Menezes, R. Goodfellow and P. Granger, *Pure Appl. Chem.*, 2001, **73**, 1795–1818.

[S7] C. A. Reed, *Acc. Chem. Res.*, 2010, **43**, 121–128.

[S8] Z. Xie, T. Jelínek, R. Bau and C. A. Reed, *J. Am. Chem. Soc.*, 1994, **116**, 1907–1913.

[S9] C. B. Caputo, L. J. Hounjet, R. Dobrovetsky and D. W. Stephan, *Science*, 2013, **341**, 1374–1377.

[S10] *Agilent CrysAlis PRO*, 2012, Agilent Technologies, Yarnton, UK.

[S11] G. M. Sheldrick, *Acta Crystallogr., Sect. A.*, 1990, **46**, 467–473.

[S12] G. M. Sheldrick, *Acta Crystallogr., Sect. A.*, 2008, **64**, 112–122.

[S13] Mercury 3.9; Cambrige Crystallographic Data Center: https://www.ccdc.cam.ac.uk/solutions/csd-system/components/mercury/.

[S14] A. D. Bochevarov, E. Harder, T. F. Hughes, J. R. Greenwood, D. A. Braden, D. M. Philipp, D. Rinaldo, M. D. Halls, J. Zhang and R. A. Friesner, *Int. J. Quantum Chem.*, 2013, **113**, 2110–2142.
